# Diversity of cultivable protease-producing bacteria and their extracellular proteases associated to scleractinian corals

**DOI:** 10.7717/peerj.9055

**Published:** 2020-05-06

**Authors:** Hongfei Su, Zhenlun Xiao, Kefu Yu, Qinyu Huang, Guanghua Wang, Yinghui Wang, Jiayuan Liang, Wen Huang, Xueyong Huang, Fen Wei, Biao Chen

**Affiliations:** 1 Coral Reef Research Center of China, Guangxi University, Nanning, Guangxi, China; 2 Guangxi Laboratory on the Study of Coral Reefs in the South China Sea, Guangxi University, Nanning, Guangxi, China; 3 School of Marine Sciences, Guangxi University, Nanning, Guangxi, China

**Keywords:** Diversity, Cultivable, Protease-producing bacteria, Scleractinian corals, Extracellular protease diversity, Hydrolytic ability, Serine proteases, Metalloprotease, Firmicutes, Proteobacteria

## Abstract

Protease-producing bacteria play a vital role in degrading organic nitrogen in marine environments. However, the diversity of the bacteria and extracellular proteases has seldom been addressed, especially in communities of coral reefs. In this study, 136 extracellular protease-producing bacterial strains were isolated from seven genera of scleractinian corals from Luhuitou fringing reef, and their protease types were characterized. The massive coral had more cultivable protease-producing bacteria than branching or foliose corals. The abundance of cultivable protease-producing bacteria reached 10^6^ CFU g^−1^ of coral. Phylogenetic analysis of 16S rRNA gene sequences revealed that the isolates were assigned to 24 genera, from which 20 corresponded to the phyla *Firmicutes* and *Proteobacteria. Bacillus* and *Fictibacillus* were retrieved from all coral samples. Moreover, *Vibrio* and *Pseudovibrio* were most prevalent in massive or foliose coral *Platygyra* and *Montipora.* In contrast, 11 genera were each identified in only one isolate. Nearly all the extracellular proteases from the bacteria were serine proteases or metalloproteases; 45.83% of isolates also released cysteine or aspartic proteases. These proteases had different hydrolytic ability against different substrates. This study represents a novel insight on the diversity of cultivable protease-producing bacteria and their extracellular proteases in scleractinian corals.

## Introduction

In coral reef ecosystems, nitrogen is as essential as carbon and other nutrients, but it is present at very low concentrations in the water surrounding coral reefs (Suzuki & Casareto, 2011). Abundant particulate organic material (including proteins, amino acids and various biomolecules) and detritus that carry organic nitrogen (OrgN), are the main nitrogen sources in coral reefs (Atkinson, Falter & Hearn, 2001). The imbalance of consumption and production between inorganic and organic nitrogen suggests that OrgN is produced in dissolved forms (dissolved organic nitrogen, DON), which are important components of coral reef metabolism and biogeochemical cycles (Larkum, Kennedy & Muller, 1988; Rädecker et al., 2015). In this cycle, particulate OrgN must be first decomposed into dissolved OrgN and then undergo ammonification, nitrification, and denitrification resulting in formation of nitrogen gas, which will be released into the atmosphere. These processes are mainly performed by bacteria through degradation enzymes (Hunter, Mills & Kostka, 2006; Olson & Lesser, 2013).

A large amount of the dry tissue weight (about 50%) of corals is composed of proteins that exist in many forms and have dozens of functions, such as comprising muscle protein and scaffolds for cytoskeletons, acting as biological catalysts, and giving corals wondrous colorations (Fitzgerald & Szmant, 1997). Organisms absorb organic matter from the environment to assemble tissue. Protease-producing bacteria are considered the main degraders of OrgN in the marine environment because they secrete extracellular proteases that hydrolyze the OrgN into peptides and amino acids that are easily taken up by organisms for subsequent catabolism (Chen et al., 2003; Hui-Lin et al., 2012). Protease-producing bacteria not only enhance protein digestibility and improve the growth of the host but also reduce organic waste in aquaculture (Amin, 2018; Shi et al., 2016). Some bacteria are potential dietary probiotics that promote growth and improve immunity and disease resistance (Amin, 2018; Selim & Reda, 2015).

The diversity of protease-producing bacteria and extracellular proteases from coastal and ocean sediments has been studied (Li et al., 2017; Ming-Yang et al., 2013; Zhou et al., 2009). Protease-producing bacteria have been found into four major phyla, *Proteobacteria, Firmicutes, Actinobacteria*, and *Bacteroidetes*, and are dominated by *Pseudoalteromonas, Pseudomonas,* and *Bacillus* genera. Most extracellular proteases secreted by these bacteria are serine proteases and/or metalloproteases isolated from the marine sediments. Many of these proteases are in a relatively low proportion of cysteine proteases (Ming-Yang et al., 2013; Zhang et al., 2015). It is well known that corals harbor abundant, highly biodiverse, and multifunctional prokaryotic communities that play significant roles in nutrient procurement and material transformation to maintain the health of coral reef ecosystems (Blackall, Bryan & Van Oppen, 2015), which population structure is distinct from that of the contiguous sediment (Melissa & Farooq, 2010) and seawater (Shinichi, Woodley & Mónica, 2010). However, there are few reports on bacterial metalloproteases from corals and on coral extracellular proteases that has significant physiological effect on coral symbionts (Anithajothi et al., 2014; Meir et al., 2009; Santos et al., 2011).

A previous study by Zhao et al. (2008) showed that the Luhuitou reef consists of 69 species of hermatypic corals that belong to 24 genera and 13 families (Zhao et al., 2008). It covers an area that has a remarkable amount of biological diversity, including typical coral reefs, many valuable fisheries, and countless microorganisms. However, the functional roles of the microbial symbionts of corals are still poorly understood, and the diversity and functional redundancy of protease-producing bacteria and their extracellular proteases has seldom been addressed. In this paper, seven genera of scleractinian corals from Luhuitou fringing reef were sampled, representing several different skeletal morphology of coral. After isolation and screening, 136 cultivable protease-producing bacterial strains were obtained from these corals, and their diversity was investigated by phylogenetic analysis of 16S rRNA gene sequences. The diversity of bacterial extracellular proteases secreted by these strains was studied using different substrates and inhibitors tests. This study aimed to explore the diversity of protease-producing bacterial communities in corals and characterize their extracellular proteases.

## Materials and methods

### Sampling and coral identification

Coral samples were collected from the Luhuitou coral reef (109°28′E, 18°13′N), located in the south of Hainan Island, east of Sanya Bay, and west of Luhuitou Peninsula in China. In September 2017, coral fragments (approximately 10 × 10 cm) were collected from seven healthy corals using a hammer and punch at a depth of 2–10 m (Table 1). The collected samples were gently washed with sterile seawater and placed in sterile plastic bags. All samples were stored at low temperatures (0–4 °C) to reduce mucus secreting and immediately transported to the laboratory for isolation of protease-producing bacteria. After removing tissue using an airbrush, the coral skeleton was ready for immediate species identification. All coral samples were identified through ecological and morphological characteristics, according to the book by Veron (2009).

**Table 1:** Cumulative list of cultivable protease-producing bacteria in corals.

**CORAL GENERA**									
		**A**	**L**	**B**	**C**	**F**	**D**	**Q**	**Total and** **Rate (%)**
**GENERA DISTRIBUTION**									
***Fimicutes*(5)**	*Bacillus*	5	2	1		5	3	6	22 (16.18%)
	*Lysinibacillus*			2					2 (1.47%)
	*Paenisporosarcina*		1						1 (0.74%)
	*Fictibacillus*	3	1	3	1	5	2	2	17 (12.50%)
	*Exiguobacterium*			1		1	2		4 (2.94%)
***Proteobacteria*(15)**									
	*Sphingomonas*					1			1 (0.74%)
	*Paracoccus*	1							1 (0.74%)
	*Ruegeria*						1		1 (0.74%)
	*Roseibacterium*				1	1			2 (1.47%)
	*Oceanicola*				1				1 (0.74%)
	*Pseudovibrio*		4		15	1			20 (14.71%)
	*Stenotrophomonas*							1	1 (0.74%)
	*Pseudomonas*			1			1	1	3 (2.21%)
	*Erwinia*				1				1 (0.74%)
	*Shewanella*		1						1 (0.74%)
	*Pseudoalteromonas*		1						1 (0.74%)
	*Idiomarina*		1						1 (0.74%)
	*Alteromonas*		1				1		2 (1.47%)
	*Microbulbifer*		3		3	1	2		9 (6.62%)
	*Vibrio*		6	2	9	2	1	14	34 (25.00%)
***Actinobacteria*(3)**									
	*Microbacterium*	2		2		1	1		6 (4.41%)
	*Micrococcus*					1			1 (0.74%)
	*Brevibacterium*					1			1 (0.74%)
***Bacteroidetes*(1)**	*Aquimarina*				2	1			3 (2.21%)
**Total strain** **number (136)**		11	21	12	33	21	14	24	136

**Notes.**

*Pocillopora*, *Porites*, *Platygyra*, *Turbinaria*, *Faviia*, *Acropora*, *Montipora* were represented by A, B, C, D, F, L, Q.

### Cultivation and screening of protease-producing bacteria

Protease-producing bacteria were cultivated and screened according to a previous study (Li et al., 2017). Briefly, 1 g fresh weight of corals, including tissue, mucus and skeleton (triplicate samples collected from one species were weighed equally before mixing), was diluted in 10 mL sterile sea water and homogenized by vortexing with sterile three mm glass beads for 10 min at a speed setting of 6.0. A portion of the resulting homogenates was pooled and filtered at 0.22 µm to remove bacteria and simulate coral environment for fostering more bacteria on plate. 100 mL eluate was then added to media cooled to 50 °C, which was composed of 1/10 2216E (tenfold dilution of 2216E) and 1.5% (w/v) agar powder in 1 L seawater at pH 8.0, and supplemented with 1% (w/v) casein. The preparation and addition of coral tissue homogenates was performed according to Rebecca et al. with minor modifications (Certner & Vollmer, 2015). Aliquots of 100 µL of the homogenates were serially diluted (10^−1^–10^−6^) and separately spread on screening plates with three replicates. All plates for bacterial screening were incubated at 25 °C until a clear hydrolytic zone formed around the colonies. Positive colonies were selected and purified by repeated streaking until uniform or pure colonies could be detected on the same plate. Morphological characteristics (e.g., colony color and zone, hydrolytic zone) were analyzed. Pure cultures were preserved in 25% (v/v) glycerol at −80 °C for further use.

### Molecular identification of the bacterial strains

After incubation in liquid screening medium without agar, genomic DNA was extracted from the bacterial strains using the Hipure bacterial DNA kit (Magen, China). The 16S rRNA gene was amplified using the universal primer pair 27F (5′-AGAGTTTGATCMTGGCTCAG-3′) and 1492R (5′-TACGGYTACCTTGTTACGACTT-3′). PCR amplifications were performed in a Mastercycler pro (Eppendorf, Germany) in a final volume of 30 µL, containing 1.2 µL (10 µM) of each primer, 0.1 µL of template DNA (approximately 60 ng), and 25 µL Easy Taq super Mix (Transgen Biotech, Beijing). The PCR conditions were as follows: 95 °C for 4 min; 30 cycles at 94 °C for 45 s, 54 °C for 45 s, 72 °C for 120 s; followed by 72 °C for 10 min. PCR products were sequenced by Sangon Biotech (Shanghai, China). Identification of bacterial strains was carried out by comparison with available 16S rRNA gene sequences in GenBank using BLASTn approach to determine their closest relatives and approximate phylogenetic affiliation (Tomova et al., 2013). Samples with the same 16S rRNA gene sequence (or with only one base difference) were categorized as the same strain. A phylogenetic gene tree was generated from 16S rRNA using the neighbor-joining method with MEGA package version 7.0 (Kumar, Stecher & Tamura, 2016). The 16S rRNA gene sequences of this study were deposited in GenBank database under accession numbers MK617631 –MK617766.

### Hydrolysis of casein, elastin, and gelatin by bacterial extracellular proteases

Three solid basic media (2216E) were prepared, and supplemented with 1% (w/v) casein, 0.5% (w/v) gelatin, or 0.5% (w/v) elastin powder, respectively (Li et al., 2017). Protease-producing isolates were inoculated with sterilized toothpicks on plates and then incubated at 25 °C for 3 days. The ratio of the hydrolytic zone diameter to the colony diameter (hydrolytic zone/colony, H/C) was calculated to indicate the enzymatic activity for each substrate.

### Effect of protease inhibitors on protease activity

The inhibitor assay was performed as described in a previous paper (Li et al., 2017). Each protease-producing strain was cultivated in the screening medium without agar at 25 °C with shaking (170 rpm) for 3 d. The culture supernatant was obtained by centrifugation at 14,000×g at 4 °C for 5 min. The supernatant was diluted to maintain OD_660_ between 0.4 and 0.8 in 20 mM Tris–HCl (pH 8.0), and incubated at 15 °C for 20 min in the presence of different inhibitors including phenylmethylsulfonyl fluoride (1.0 mM, PMSF, Aladdin), 1,10-Phenanthroline (1.0 mM, OP, Aladdin), E-64 (0.1 mM, Sigma), and pepstatin A (0.1 mM, Aladdin). The residual activity of each sample was measured in triplicates using samples without inhibitors as controls (100%), and the relative activity (%) of samples was calculated.

## Results

### Sample collection and coral identification

The 17 scleractinian coral samples were classified into seven genera, corresponding to branching (*Pocillopora* and *Acropora*), massive (*Porites*, *Platygyra*, and *Favia*), and foliose (*Turbinaria* and *Montipora*) corals according to skeletal morphology, and used for incubation and screening of protease-producing bacteria.

### Isolation and quantification of protease-producing bacteria from corals

After incubation at 25 °C for 12–15 days, approximately 20–100 colonies of various color, size, and morphology, appeared in samples diluted to 10^−3^–10^−4^. Manual counts indicated that the most abundant bacteria reached 10^7^CFU g^−1^ in *Platygyra* coral samples. Nearly 20% of the colonies had hydrolytic zones. The massive coral had more cultivable protease-producing bacteria than branching or foliose corals. Approximately 200 colonies were obtained for further identification and characterization.

### Phylogenetic Diversity of Protease-Producing Bacteria Isolated from Corals

The 16S rRNA gene of the 136 isolates, which contained nearly 1,400 bp, were amplified and partially sequenced. Samples with the same 16S rRNA gene sequence (or with only one base difference) were categorized as the same strain, resulting in a total of 76 different strains. Phylogenetic affiliation was assigned based on the sequences of the 16S rRNA genes. As shown in Table 1, the 136 strains were assigned to 24 genera. Most of the bacterial isolates were assigned to 20 genera within the phyla *Firmicutes* and *Proteobacteria*, except for isolates (sC2, sC26, sJ58) from *Platygyra* and *Faviidae* belonging to *Aquimarina* in the phylum *Bacteroidetes*, and eight isolates belonging to *Microbacterium* (sA9, sA61, sD33, sF16b, yB24b, yB18), *Micrococcus* (sF19) and *Brevibacterium* (yF15) in the phylum *Actinobacteria*. The *Firmicutes* included *Bacillus*, *Lysinibacillus*, *Paenisporosarcina*, *Fictibacillus*, and *Exiguobacterium*. The *Proteobacteria* were mainly affiliated with the phyla *Alphaproteobacteria* and *Gammaproteobacteria*, including *Sphingomonas*, *Paracoccus*, *Ruegeria*, *Roseibacterium*, *Oceanicola*, *Enterobacteriaceae*, *Pseudovibrio*, and *Stenotrophomonas*, *Pseudomonas*, *Erwinia*, *Shewanella*, *Pseudoalteromonas*, *Idiomarina*, *Alteromonas*, *Microbulbifer*, *Vibrio*, and *Lucibacterium*. Four of the most predominant genera were *Bacillus* (16.18%) and *Fictibacillus* (12.50%) in the phylum of *Firmicutes* that were present in all coral samples, *Pseudovibrio* (14.71%) and *Vibrio* (25.00%) in the phylum of *Proteobacteria* that hat were present in almost all samples. The largest number of protease-producing bacteria was in *Platygyra* and *Montipora* which corresponded massive or foliose corals. *Sphingomonas*, *Paracoccus*, *Ruegeria*, *Stenotrophomonas*, *Oceanicola*, *Shewanella*, *Pseudoalteromonas*, *Idiomarina*, *Alteromonas*, *Micrococcus* and *Brevibacterium* were represented only by one isolate each, 11 isolates in total, corresponding to 8.1% of the total. The rest of genera constituted 1.4% to 7.3% of all strains. These results revealed that the predominant bacteria in corals of various genera were different. In massive coral (*Platygyra*), *Pseudovibrio* (42.80%) and *Vibrio* (25.00%) were the predominant protease-producing bacteria. In foliose coral (*Montipora*), *Bacillus* (24.00%) and *Vibrio* (56.00%) were the predominant protease-producing bacteria.

**Figure 1:**
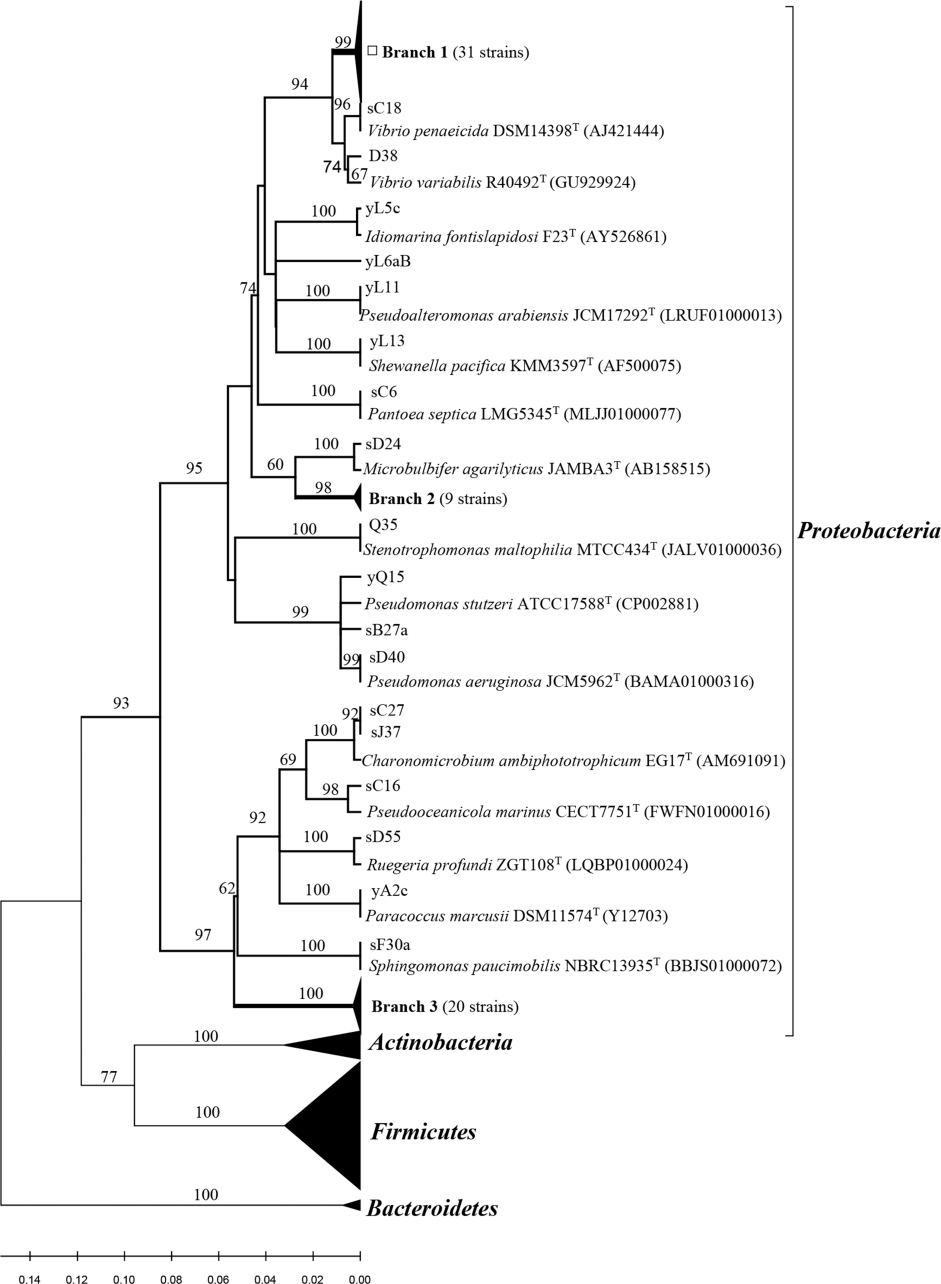
Phylogenetic tree of the protease-producing *Proteobacteria* isolated from Scleractinian corals based on 16S rRNA gene sequences. Phylogenetic tree of the protease-producing Proteobacteria isolated from Scleractinian corals based on 16S rRNA gene sequences. The tree was constructed by neighbor-joining method using MEGA package version 7.0. Only bootstrap values greater than 50% are presented in the nodes. The scale bar represents 2% nucleotide substitution. Branch 1 indicates 31* Vibrio* strains similar to *Vibrio owensii* LMG 25443^T^ (JPRD01000038). Branch 2 indicates nine *Microbulbifer* strains similar to *Microbulbifer variabilis* Ni2088^T^ (AB167354). Branch 3 indicates 20 *Pseudovibrio* strains similar to *Pseudovibrio denitrificans* DSM17465^T^ (jgi.1107980).

**Figure 2:**
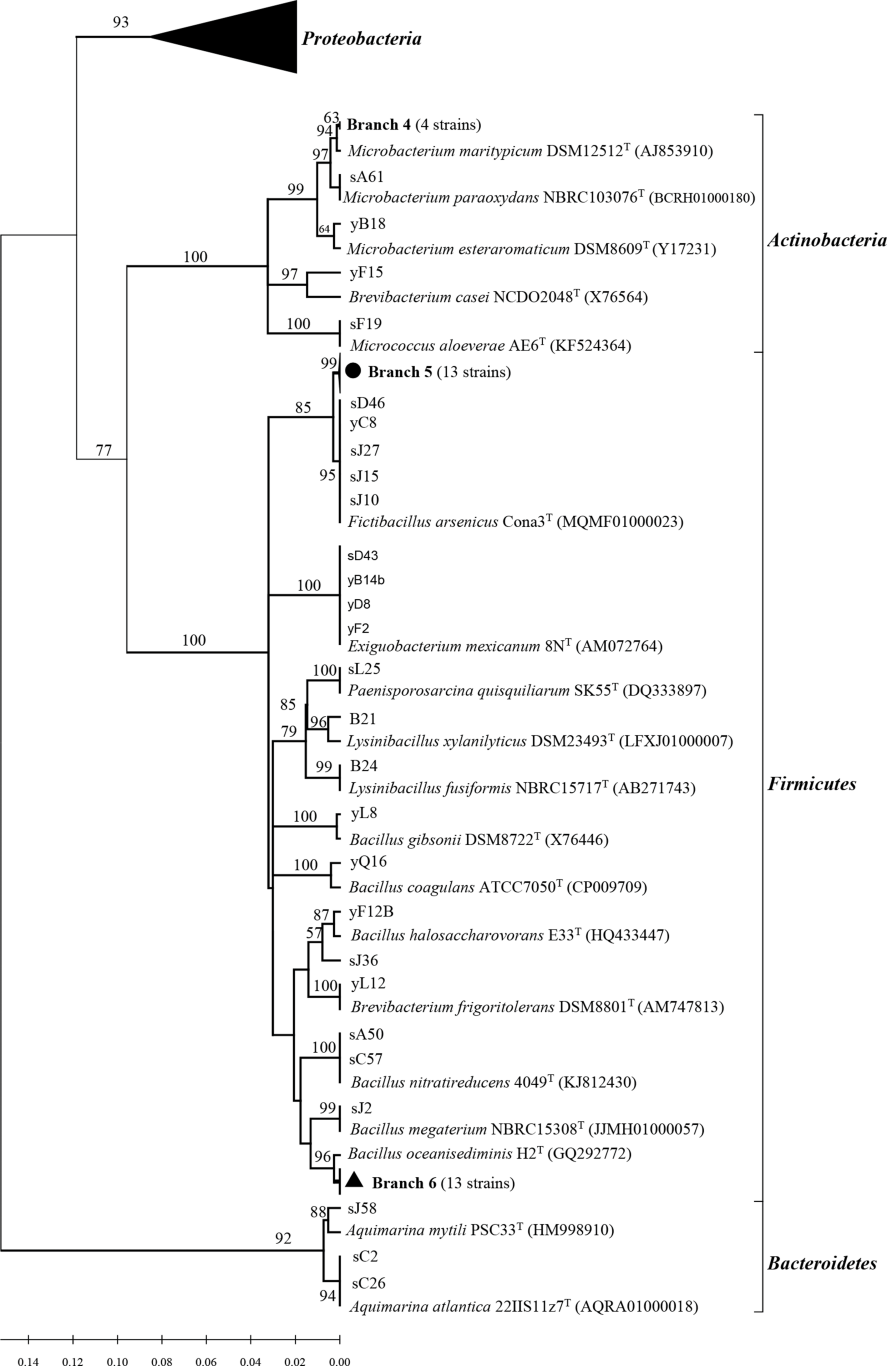
Phylogenetic tree of the protease-producing *Actinobacteria*, *Firmicutes* and *Bacteroidetes* isolated from Scleractinian corals based on 16S rRNA gene sequences. The tree was constructed by neighbor-joining method using MEGA package version 7.0. Only bootstrap values greater than 50% are presented in the nodes. The scale bar represents 2% nucleotide substitution. Branch 4 indicates four *Micrococcus* strains similar to *Micrococcus aloeverae* AE-6 T (KF524364). Branch 5 indicates 13 *Fictibacillus* strains similar to *Fictibacillus nanhaiensis* JSM082006 T (GU477780). Branch 6 indicates 13 *Bacillus* strains similar to *Bacillus megaterium* NBRC 15308 T (JJMH01000057).

All related sequences from this study and reference sequences from the GenBank database were used to construct a neighbor-joining tree (Fig. 1 and 2). Approximately thirty one strains from *Poritidae*, *Platygyra*, and *Montipora* formed Branch 1 in Fig. 1, with 99% sequence similarity with the type strain *Vibrio parahaemolyticus* NBRC 12711. Nineteen *Pseudovibrio* strains (Branch 3 from three corals) (Fig. 1) clustered with *Pseudovibrio denitrificans* DSM 17465 with 99–100% similarity. Thirteen *Bacillus* strains (Branch 6 from four corals) were closest to *Bacillus oceanisediminis* H2^T^ (99.2–100%). The phylogenetic relationships of the other strains are shown in Fig. 1 and 2. The results suggest that the diversity and composition of protease-producing bacterial communities was various in different genera of coral, and the highest diversity and evenness in community were present in *Acroporidae, Faviidae* and *Platygyra* (Table 1).

### Diversity of the extracellular proteases produced by the bacteria

The diversity of the bacterial extracellular proteases in corals was investigated using substrate specificity assays that measured the H/C ratio on plates containing casein, gelatin, or elastin (Table 2). Of the 76 isolates, 48 produced enough protease for enzymatic assays. The extracellular proteases generated by these strains produced obvious hydrolytic zones on casein and gelatin media. These isolates were screened from medium containing casein and gelatin; however, they showed very different hydrolytic abilities against casein and gelatin. The extracellular proteases from isolates sC18 and sL3 belonging to *Vibrio*, isolates sC1, sD13, and sD19 belonging to *Microbulbifer*, isolate yL5c belonging to *Idiomarina*, and isolates sC2 and yF15 belonging to *Brevibacterium* had high caseinolytic activity with H/C ratios over 4.0. The extracellular proteases from *Vibrio* (sL3), *Paracoccus* (yA2c), *Brevibacterium* (sC2), *Bacillus* (yF12B, sA10, sJ2 and yQ16) showed high gelatinolytic activity with H/C ratios over 6.0. However, only 24 isolates (50.0% of the total isolates) were capable of hydrolyzing elastin. All elastinolytic isolates produced proteases with various levels of activity against all three proteins, for example, bacillus (yQ16). Although the isolates belong to the same genus, they showed different levels of degradation of elastin. Different substrate specificities reflect that these extracellular proteases may belong to different kinds of metalloproteases or serine proteases.

In addition, the diversity of the bacterial extracellular proteases in corals was investigated using protease inhibitors such as PMSF (serine protease inhibitor), OP (metalloprotease inhibitor), E-64 (cysteine protease inhibitor), and pepstatin A (aspartic protease inhibitor). Among the 78 strains, only 48 secreted enough extracellular proteases for inhibition assays. The activities of most proteases were inhibited by PMSF ranging from 23.10% to 98.56%, indicating that these extracellular proteases may be serine proteases in different proportions. The protease activities of 14 strains were inhibited more than 90% by PMSF, which indicated that they were serine proteases. The protease activities of 41 isolates were inhibited by OP with an efficiency ranging from 24.58% to 92.47%. There was slight inhibition (2.48%–12.15%) in eight isolates, while no inhibitory effect was observed in seven isolates. This indicates that most isolates tested in corals produced metalloproteases. Forty-eight (83.33%) isolates were inhibited by both PMSF and OP at different levels, suggesting that the majority of isolates produced serine proteases or metalloproteases. E-64 showed less than 30% inhibitory effect on the proteolytic activities of all proteases, and pepstatin A also showed light inhibition (1.36%–27.77%) on 35 isolates, except on sA10 and sC26. These results indicate that 45.83% of isolates produced cysteine and/or aspartic proteases. Different inhibition levels indicate that nearly all the extracellular proteases from the bacteria associated to corals are serine proteases and/or metalloproteases, and half of the isolates produced cysteine proteases or/and aspartic proteases.

**Table 2:** Summary of the diversiy analysis of the extracellular proteases of the screened strains isolated from Scleractinian corals.

**Genera**	**Strains**	**H/C ratio**^a^			**Inhaibition ratio**^b^**(%)**			
		**Casein**	**Gelatin**	**Elastin**	**PMSF** **(1 mM)**	**OP** **(1 mM)**	**E64** **(0.1 mM)**	**P-A** **(0.1 mM)**
*Vibrio*	sC18	4.20	5.16	0	54.57	49.26	26.43	19.43
	sC57	2.30	2.41	4.00	81.86	6.60	27.74	18.21
	D38	3.81	4.10	0	ND	64.20	ND	ND
	sL1	3.90	1.23	0	97.68	86.24	29.02	0
	sL3	5.10	6.09	0	59.55	60.42	4.82	3.88
	sQ3	1.88	3.56	1.41	91.09	87.88	2.67	−3.66
	sQ48	2.16	3.18	1.53	98.56	81.12	10.25	16.02
*Idiomarina*	yL5c	5.13	6.17	0	84.56	46.45	0	0
*Pseudoalteromonas*	yL11	4.73	4.02	1.67	61.22	16.42	0	0
*Shwanella*	yL13	2.86	2.55	1.54	88.03	−1.92	5.76	8.23
*Pantoea*	sC6	2.16	2.02	0	65.2	92.47	6.12	−4.00
*Microbulbifer*	sC1	4.1	4.22	0	93.01	67.83	10.53	23.32
	sC9	3.78	2.16	0	91.78	75.26	9.25	−2.973
	sC10	3.66	2.45	0	86.5	0	9.32	2.70
	sD13	4.52	3.21	0	92.87	2.78	14.38	15.88
	sD19	4.62	3.20	0	80.04	0	0	1.72
	sL30	3.71	4.11	0	74.35	−3.52	6.33	8.76
*Strenotrophomonas*	Q35	3.62	2.75	1.63	92.52	83.72	0.90	20.68
*Pseudomonas*	sB27a	2.47	2.04	3.75	66.11	30.12	−6.12	0
	sD40	3.20	2.81	2.36	91.30	82.74	0	−15.86
	yQ15	4.00	3.42	1.26	92.50	78.14	1.20	6.46
*Oceanicola*	sC16	3.60	3.25	1.58	63.14	51.51	17.27	27.77
*Ruegeria*	sD55	3.02	3.56	2.36	53.03	57.04	12.23	24.30
*Paracoccus*	yA2c	3.65	7.50	3.24	40.32	25.52	1.21	1.36
*Sphingomonas*	sF30a	3.26	3.12	2.77	62.79	56.68	14.31	14.73
*Pseudovibrio*	sC3	2.30	2.35	0	84.73	73.26	13.03	9.81
	sC13	2.36	3.20	0	52.80	62.76	14.56	5.89
	sC15	2.56	2.31	0	65.01	47.92	15.76	3.58
	sL47	2.56	1.28	0	54.42	37.34	19.95	2.48
	yF15	4.00	3.65	0	82.75	37.91	2.23	4.30
*Micrococcus*	sF19	1.76	2.15	0	88.26	8.24	16.75	17.67
*Fictibacillus*	sD46	1.62	2.81	2.45	90.23	56.42	28.56	8.48
	sJ27	2.34	1.74	0	87.12	50.14	12.42	16.42
	yQ18	1.88	2.01	2.01	84.12	40.13	8.45	3.88
*Lysinibacillus*	B21	3.21	4.21	2.54	41.60	45.25	2.47	8.29
	B24	3.02	4.05	3.21	65.21	42.00	−12.10	8.14
*Bacillus*	sA10	1.64	5.21	0	92.85	2.48	15.88	33.29
	sA50	2.32	4.00	2.65	85.30	76.40	24.59	25.39
	yF12B	2.32	5.52	2.45	23.10	65.01	−1.25	6.24
	sJ2	2.15	5.22	0	85.09	44.68	15.51	12.44
	sJ36	1.33	1.42	0	90.20	−5.84	0	5.05
	yL8	2.17	4.32	3.65	89.32	5.03	13.02	0
	yL12	2.36	2.30	1.96	86.20	44.52	12.34	0
	yQ16	3.02	5.25	5.63	56.56	43.22	0	0
*Aquimarina*	sC2	3.60	4.32	0	90.00	12.15	12.34	35.02
	sC26	2.46	3.30	2.26	82.07	0	5.76	8.23
	sJ58	3.01	3.65	0	77.62	10.31	23.72	11.44

**Notes.**

a H/C ratio is the ratio of the hydrolytic zone diameter vs. the colony diameter of a colony on the plate.

a Inhibition ratio (%) was calculated by using control activity minus the relative activity of a sample with an inhibitor and the activity of a sample without any inhibitor was taken as a control (%).

PMSF phenylmethylsulfonyl fluoride OP 1 10-phenanthroline P-A pepstatin A

*Pocillopora*, *Porites*, *Platygyra*, *Turbinaria*, *Faviia*, *Acropora*, *Montipora* were represented by A, B, C, D, F, L, Q.

## Discussion

In a previous study, the numbers of bacteria in mucus and tissue samples were detected by SYBR gold staining, and the cultivable population on Marine Agar was between 10^5^ and 10^6^/cm^2^ of coral surface (Koren & Rosenberg, 2006). Our report focused on protease-producing bacteria in corals. The highest abundance of cultured bacteria reached 10^7^ CFU g^−1^ in some coral samples, which is higher than that in previous reports. Only 20% of the colonies had detectable protease activity, which corresponds to the community of protease-producing bacteria isolated from the South China Sea (10^6^ CFU g^−1^) (Zhou et al., 2009), this is more than in sub-Antarctic sediments (10^5^ CFU g^−1^) in Jiaozhou Bay (Zhang et al., 2015), and in Laizhou Bay (10^4^ CFU g-1) (Li et al., 2017). And protease-producing bacteria can be found in all coral samples, this result indicates that a sizable population of protease-producing bacteria exist in corals.

Most reports estimate that important sources of DON come from bacteria and other organisms associated with corals (Ferrier, 1991; Grover et al., 2003; Renaud et al., 2008). Coral reefs are considered “oligotrophic areas” based on their low nitrate concentrations, which means that the degradation rate of organic matter in dissolved and particulate forms must be fast enough (Christian et al., 2004; Muscatine & Porter, 1977). It is not known how much DON is produced by bacteria, and few reports refer to the degradation of DON, which results in a failure to characterize microbial and to address the adaptive physiology and functional attributes of bacteria. In this study, we investigated the protease-producing bacterial communities and the diversity of extracellular proteases to reveal physiology and functional attributes of bacteria in scleractinian corals.

The bacterial phyla associated with corals were identified with the development of culture-independent high-throughput DNA sequencing methods. Overall, *Proteobacteria*, *Bacteroidetes*, *Firmicutes*, *Cyanobacteria*, and *Chloroflexi* were the most abundant population with an abundance of over 90%, which is similar to other coral species (Jie et al., 2013; Liang et al., 2017). It have been found that the core microbiomes of different corals were reliably dominated by the phylum *Proteobacteria*, specifically *Gammaproteobacteria* and *Alphaproteobacteria*, and secondarily by *Bacteroidetes* and *Actinobacteria* (Chu & Vollmer, 2016). Similarly, in our study, *Proteobacteria* was the predominant cultivable protease-producing bacterial phyla (58.1%), represented by the genera *Stenotrophomonas*, *Pseudomonas*, *Erwinia*, *Shewanella*, *Pseudoalteromonas*, *Idiomarina*, *Alteromonas*, *Microbulbifer*, *Vibrio*, and *Lucibacterium*, followed by *Gammaproteobacteria* with an abundance of 39.0% (Table 1). *Vibrio* was the predominant genus, accounting for 23.5% of the total strains. This is different from the predominant protease-producing bacteria isolated from the sediments of the South China Sea, which belong to *Alteromonas* and *Pseudoalteromonas* (Zhou et al., 2009). *Firmicutes* was the second most abundant phylum (33.8%), with *Bacillus* as the dominant genus that was found in all coral samples (Table 1). This was consistent with a paper that described protease-producing bacteria and their extracellular proteases in the sediments of Laizhou Bay by Li et al. (2017) which illustrates that *Firmicutes* or *Bacillus* might play important roles in biodegradation in corals. Unlike these studies, ours is the first study reporting that numerous isolates belonging to *Fictibacillus* and *Pseudovibrio* possess protease-producing abilities.

There were differences in the diversity of protease-producing bacteria between species, although only a small portion of bacteria was screened. For example, the most abundant and diverse protease-producing bacteria was found in *Platygyra* sp. and *Faviidae* sp*.*, both belonging to massive corals. This result is consistent with previous findings suggesting that abundant bacterial species may help corals to respond to abnormal changes of temperature (Liang et al., 2017). Although only 20 protease-producing isolates were obtained from *Acroporidae* sp. (a fast growing branching species) (Edwards & Clark, 1999), they have the second highest diversity.

Because of their broad biochemical diversity, microbes are an excellent source of proteases. Some proteolytic bacteria have been reported to be associated not only with fresh water but marine fish processing waste as well. The protease produced by *Bacillus proteolyticus* CFR3001 lyses the cells of pathogenic bacteria such as *Escherichia coli*, *Listeria monocytogenes*, *Bacillus cereus*, and *Yersinia enterocolitica* (Bhaskar et al., 2007). The protease-producing *Bacillus subtilis* E20 also shows great potential to increase the growth performance of *Litopenaeus vannamei* and increases disease resistance against the pathogen *Vibrio alginolyticus* (Liu et al., 2009; Tseng et al., 2009). To date, no study had demonstrated protease production by probiotic bacteria in corals; however, in the present study, we found that many *Bacillus* isolates from corals that displayed high proteolytic activity against casein, gelatin and/or elastin were widespread in all coral samples, and may play an important role in coral health. The analysis of protease-producing bacterial diversity in this study suggests that bacterial species may benefit coral growth, transplant, and regeneration.

Most isolates produced serine and/or metalloproteases, which was similar to protease-producing bacteria isolated from coastal sediments (Li et al., 2017; Ming-Yang et al., 2013; Zhou et al., 2009). Numerous studies have demonstrated that the zinc-metalloproteases are present in *Vibrio* pathogenic strains, for example, the zinc-metalloprotease of *Vibrio coralliilyticus* causes rapid photosystem II inactivation of *Symbiodinium* endosymbionts, leading to *Pocillopora damicornis* bleach (Yael, Maya & Eugene, 2003). *V. coralliilyticus* strain P1 has a diverse zinc-metalloproteases that possibly enables it to be an efficient coral pathogen (De et al., 2011). In this study, 32 isolates belonging to *Vibrionaceae*, but none belonging to *V. coralliilyticus* were found, suggesting that metalloproteases are widespread in protease-producing bacteria associated with corals. However, nearly 45.83% of isolates were also inhibited by E-64, which was different from the protease-producing bacteria isolated from coastal sediments (Ming-Yang et al., 2013; Zhou et al., 2009). Twenty-five isolates showed different levels of degradation activity against elastin, which is the most insoluble and difficult to degrade of the three protein substrates tested due to its high molecular weight protein polymer. Furthermore, the *Bacillus* isolates from corals displayed higher proteolytic activities against casein, gelatin and/or elastin than those in South China Sea (Zhou et al., 2009) or than those in the sediments of Laizhou Bay (Li et al., 2017). Strains with multiple enzyme types and different substrates specificities were observed in corals, which may allow the bacterial community to more effectively and rapidly hydrolyze complex OrgN sources for coral reefs.

## Conclusion

This paper is the first systematic study to investigate the phylogenetic diversity of cultivable protease-producing bacteria associated with scleractinian corals and their extracellular protease types. *Fictibacillus*, *Bacillus*, *Vibrio*, and *Pseudovibrio* were the most retrieved genera, and *Fictibacillus* and *Pseudovibrio* that possess proteolytic activity were isolated for a first time from Luhuitou fringing coral reefs. In addition, some bacterial genera (*Bacillus*, *Fictibacillus* and *Vibrio*) were found in all samples but were most dominant in *Platygyra* and *Montipora*, which are massive or foliose coral. Forty-eight isolates showed different levels of degradation activities against casein and gelatin. Twenty-five of them simultaneously displayed hydrolytic activity against casein, gelatin, and elastin. Most protease-producing bacteria associated to scleractinian corals produced metalloproteases and/or serine proteases, and 22 isolates also released cysteine or aspartic proteases.

##  Supplemental Information

10.7717/peerj.9055/supp-1 Figure S1 Bacteral culture on CFCF plate A large number of coral symbiotic bacteria with different colors grown on CFCF plates

10.7717/peerj.9055/supp-2 Figure S2 Screening of protase-producing bacteria on plate Cultivable protease-producing bacteria displayed a clear hydrolytic zone around the colonies.

10.7717/peerj.9055/supp-3 Supplemental Information 1 The 16S rRNA of 136 isolates

10.7717/peerj.9055/supp-4 Supplemental Information 2 GenBank flat file

## Additional Information and Declarations

### Data Availability

The following information was supplied regarding data availability: The raw data is available at GenBank: MK617631 –MK617766.

## References

[ref-1] Amin M (2018). Marine protease-producing bacterium and its potential use as an abalone probiont. Aquaculture Reports.

[ref-2] Anithajothi R, Nagarani N, Umagowsalya G, Duraikannu K, Ramakritinan CM (2014). Screening, isolation and characterization of protease producing moderately halophilic microorganism *Halomonas meridiana* associated with coral mucus. Toxicological & Environmental Chemistry.

[ref-3] Atkinson M, Falter J, Hearn C (2001). Nutrient dynamics in the Biosphere 2 coral reef mesocosm: water velocity controls NH_4_ and PO_4_ uptake. Coral Reefs.

[ref-4] Bhaskar N, Sudeepa ES, Rashmi HN, Tamil Selvi A (2007). Partial purification and characterization of protease of *Bacillus proteolyticus* CFR3001 isolated from fish processing waste and its antibacterial activities. Bioresource Technology.

[ref-5] Blackall LL, Bryan W, Van Oppen MJH (2015). Coral-the world’s most diverse symbiotic ecosystem. Molecular Ecology.

[ref-6] Certner RH, Vollmer SV (2015). Evidence for autoinduction and quorum sensing in white band disease-causing microbes on Acropora cervicornis. Scientific Reports.

[ref-7] Chen XL, Zhang YZ, Gao PJ, Luan XW (2003). Two different proteases produced by a deep-sea psychrotrophic bacterial strain, Pseudoaltermonas sp. SM9913. Marine Biology.

[ref-8] Christian W, Markus H, Anke K, Kremb SG, Rasheed MYM, Jorgensen BB (2004). Coral mucus functions as an energy carrier and particle trap in the reef ecosystem. Nature.

[ref-9] Chu ND, Vollmer SV (2016). Caribbean corals house shared and host-specific microbial symbionts over time and space. Environmental Microbiology Reports.

[ref-10] De OSE, Jr AN, Dias GM, Mazotto AM, Vermelho A, Vora GJ, Wilson B, Beltran VH, Bourne DG, Le RF (2011). Genomic and proteomic analyses of the coral pathogen *Vibrio coralliilyticus* reveal a diverse virulence repertoire. Isme Journal.

[ref-11] Edwards AJ, Clark S (1999). Coral transplantation: a useful management tool or misguided meddling?. Marine Pollution Bulletin.

[ref-12] Ferrier MD (1991). Net uptake of dissolved free amino acids by four scleractinian corals. Coral Reefs.

[ref-13] Fitzgerald LM, Szmant AM (1997). Biosynthesis of ’essential’ amino acids by scleractinian corals. Biochemical Journal.

[ref-14] Grover R, Maguer JFO, Allemand D, Ferrier-Pagès C (2003). Nitrate uptake in the scleractinian coral Stylophora pistillata. Limnology and Oceanography.

[ref-15] Hui-Lin Z, Xiu-Lan C, Bin-Bin X, Ming-Yang Z, Xiang G, Xi-Ying Z, Bai-Cheng Z, Weiss AS, Yu-Zhong Z (2012). Elastolytic mechanism of a novel M23 metalloprotease pseudoalterin from deep-sea Pseudoalteromonas sp. CF6-2: cleaving not only glycyl bonds in the hydrophobic regions but also peptide bonds in the hydrophilic regions involved in cross-linking. Journal of Biological Chemistry.

[ref-16] Hunter EM, Mills HJ, Kostka JE (2006). Microbial community diversity associated with carbon and nitrogen cycling in permeable shelf sediments. Applied and Environmental Microbiology.

[ref-17] Jie L, Qi C, Si Z, Hui H, Jian Y, Xin-Peng T, Li-Juan L (2013). Highly heterogeneous bacterial communities associated with the South China Sea reef corals Porites lutea, Galaxea fascicularis and Acropora millepora. PLOS ONE.

[ref-18] Koren O, Rosenberg E (2006). Bacteria associated with mucus and tissues of the coral Oculina patagonica in summer and winter. Applied and Environmental Microbiology.

[ref-19] Kumar S, Stecher G, Tamura K (2016). MEGA7: molecular Evolutionary Genetics Analysis version 7.0 for bigger datasets. Molecular Biology and Evolution.

[ref-20] Larkum AWD, Kennedy IR, Muller WJ (1988). Nitrogen fixation on a coral reef. Marine Biology.

[ref-21] Li Y, Wu C, Zhou M, Wang ET, Zhang Z, Liu W, Ning J, Xie Z (2017). Diversity of cultivable protease-producing bacteria in Laizhou bay sediments, Bohai Sea, China. Frontiers in Microbiology.

[ref-22] Liang J, Yu K, Wang Y, Huang X, Huang W, Qin Z, Pan Z, Yao Q, Wang W, Wu Z (2017). Distinct bacterial communities associated with massive and branching scleractinian corals and potential linkages to coral susceptibility to thermal or cold stress. Frontiers in Microbiology.

[ref-23] Liu CH,  Chiu CS, Ho PL, Wang SW (2009). Improvement in the growth performance of white shrimp, Litopenaeus vannamei, by a protease-producing probiotic, *Bacillus subtilis* E20, from natto. Journal of Applied Microbiology.

[ref-24] Meir S, Mieog JC, Jason D, Steven V, Willis BL, Bourne DG (2009). *Vibrio* zinc-metalloprotease causes photoinactivation of coral endosymbionts and coral tissue lesions. Plos One.

[ref-25] Melissa G, Farooq A (2010). New method for counting bacteria associated with coral mucus. Applied and Environmental Microbiology.

[ref-26] Ming-Yang Z, Guang-Long W, Dan L, Dian-Li Z, Qi-Long Q, Xiu-Lan C, Bo C, Bai-Cheng Z, Xi-Ying Z, Yu-Zhong Z (2013). Diversity of both the cultivable protease-producing bacteria and bacterial extracellular proteases in the coastal sediments of king george island, antarctica. PLOS ONE.

[ref-27] Muscatine L, Porter JW (1977). Reef corals: mutualistic symbioses adapted to nutrient-poor environments. Bioscience.

[ref-28] Olson ND, Lesser MP (2013). Diazotrophic diversity in the Caribbean coral, Montastraea cavernosa. Archives of Microbiology.

[ref-29] Rädecker N, Pogoreutz C, Voolstra CR, Wiedenmann J, Wild C (2015). Nitrogen cycling in corals: the key to understanding holobiont functioning?. Trends in Microbiology.

[ref-30] Renaud G, Jean-FrançOis M, Denis A, Christine FP (2008). Uptake of dissolved free amino acids by the scleractinian coral Stylophora pistillata. Journal of Experimental Biology.

[ref-31] Santos EDO, Alves Jr AN, Dias GM, Mazotto AM, Vermelho A, Vora GJ, Wilson B, Beltran VH, Bourne DG, Le RF (2011). Genomic and proteomic analyses of the coral pathogen Vibrio coralliilyticus reveal a diverse virulence repertoire. ISME Journal.

[ref-32] Selim KM, Reda RM (2015). Improvement of immunity and disease resistance in the Nile tilapia, Oreochromis niloticus, by dietary supplementation with Bacillus amyloliquefaciens. Fish and Shellfish Immunology.

[ref-33] Shi Z, Li X-Q, Chowdhury MAK, Chen J-N, Leng X-J (2016). Effects of protease supplementation in low fish meal pelleted and extruded diets on growth, nutrient retention and digestibility of gibel carp, Carassius auratus gibelio. Aquaculture.

[ref-34] Shinichi S, Woodley CM, Mónica M (2010). Threatened corals provide underexplored microbial habitats. PLOS ONE.

[ref-35] Suzuki Y, Casareto BE, Dubinsky Z, Stambler N (2011). The role of dissolved organic nitrogen (don) in coral biology and reef ecology. Coral reefs: an ecosystem in transition.

[ref-36] Tomova I, Lazarkevich I, Tomova A, Kambourova M, Vasileva-Tonkova E (2013). Diversity and biosynthetic potential of culturable aerobic heterotrophic bacteria isolated from Magura Cave, Bulgaria. International Journal of Speleology.

[ref-37] Tseng DY, Ho PL, Huang SY, Cheng SC, Shiu YL, Chiu CS, Liu CH (2009). Enhancement of immunity and disease resistance in the white shrimp, Litopenaeus vannamei, by the probiotic, *Bacillus subtilis* E20. Fish & Shellfish Immunology.

[ref-38] Veron JEN, Gornitz V (2009). Corals and coral reefs. Encyclopedia of paleoclimatology and ancient environments.

[ref-39] Yael BH, Maya ZK, Eugene R (2003). Temperature-regulated bleaching and lysis of the coral Pocillopora damicornis by the novel pathogen Vibrio coralliilyticus. Applied and Environmental Microbiology.

[ref-40] Zhang XY, Han XX, Chen XL, Dang HY, Xie BB, Qin QL, Shi M, Zhou BC, Zhang YZ (2015). Diversity of cultivable protease-producing bacteria in sediments of Jiaozhou Bay, China. Frontiers in Microbiology.

[ref-41] Zhao M, Yu K, Zhang Q, Qi S (2008). Spatial pattern of coral diversity in Luhuitou fringing reef, Sanya, China. Acta Ecologica Sinica.

[ref-42] Zhou MY, Chen XL, Zhao HL, Dang HY, Luan XW, Zhang XY, He HL, Zhou BC, Zhang YZ (2009). Diversity of both the cultivable protease-producing bacteria and their extracellular proteases in the sediments of the South China Sea. Microbial Ecology.

