# Supplementary figures and images for: Diversity of cultivable protease-producing bacteria and their extracellular proteases associated to scleractinian corals
Source: PeerJ. 2020 May 6;8:e9055. doi: 10.7717/peerj.9055 (PMC7210813; doi:10.7717/peerj.9055)

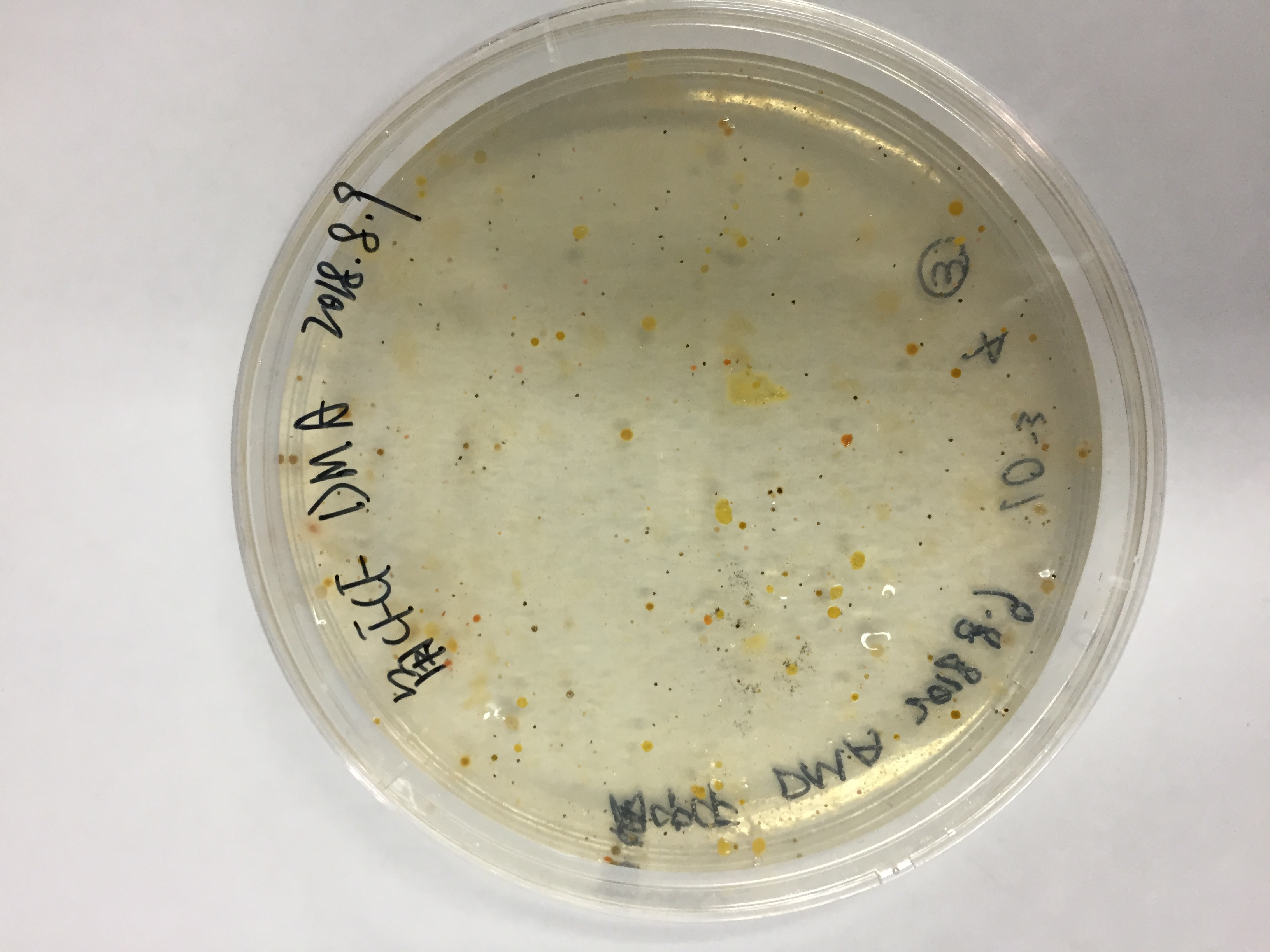

Supplement: Figure S1 — A large number of coral symbiotic bacteria with different colors grown on CFCF plates [file peerj-08-9055-s001.jpg]

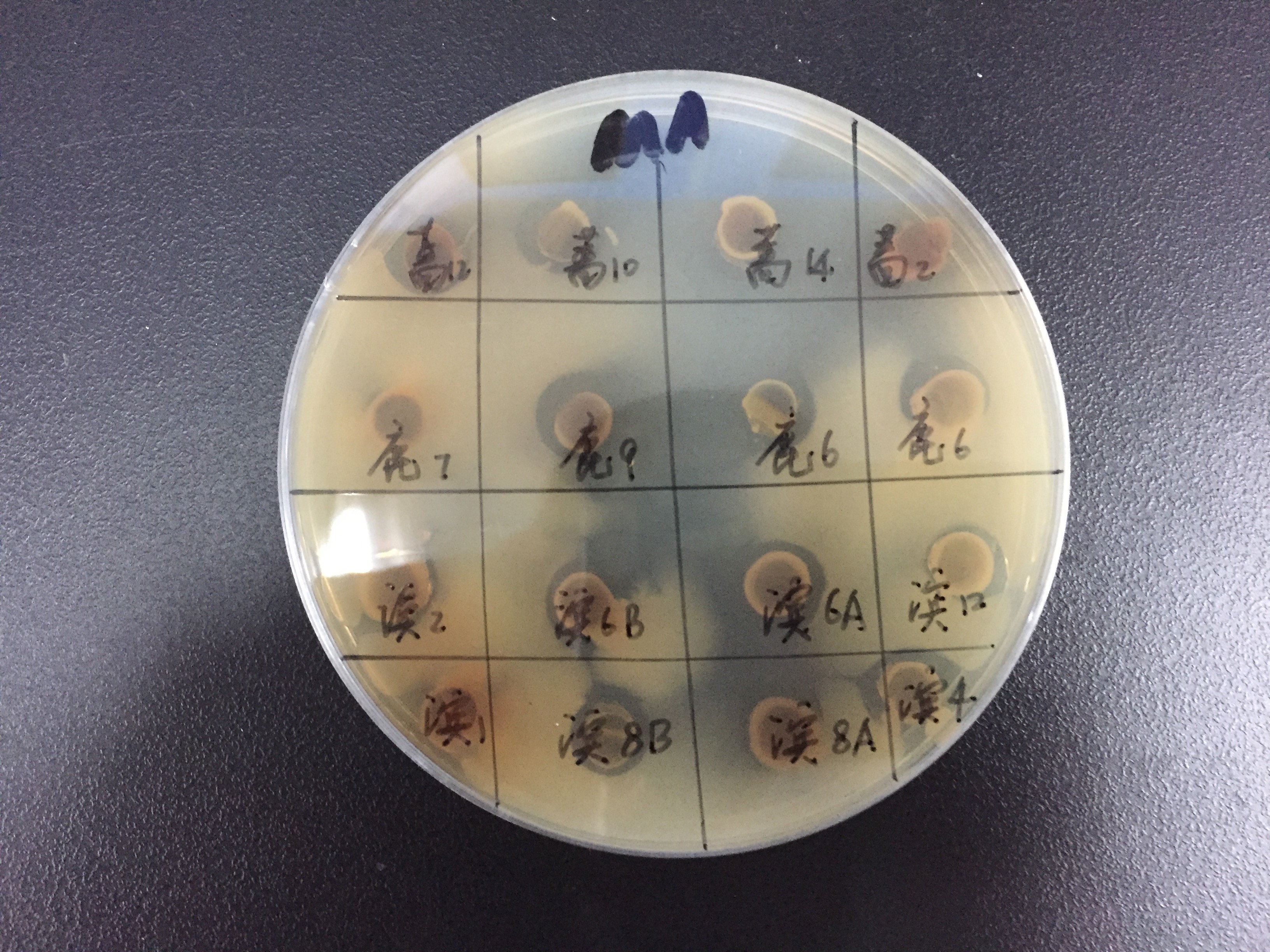

Supplement: Figure S2 — Cultivable protease-producing bacteria displayed a clear hydrolytic zone around the colonies. [file peerj-08-9055-s002.jpg]
