# Supplementary material for: Diversity of cultivable protease-producing bacteria and their extracellular proteases associated to scleractinian corals
Source: PeerJ. 2020 May 6;8:e9055. doi: 10.7717/peerj.9055 (PMC7210813; doi:10.7717/peerj.9055)
Supplement: Supplemental Information 2 [file peerj-08-9055-s004.docx]

GenBank flat file:

LOCUS MK617631 640 bp DNA linear BCT 30-MAY-2019

DEFINITION Microbacterium sp. strain sA9 16S ribosomal RNA gene, partial

sequence.

ACCESSION MK617631

VERSION MK617631

KEYWORDS .

SOURCE Microbacterium sp.

ORGANISM Microbacterium sp.

Bacteria; Actinobacteria; Micrococcales; Microbacteriaceae;

Microbacterium.

REFERENCE 1 (bases 1 to 640)

AUTHORS Su,H., Huang,Q. and Yu,K.

TITLE Diversity of Cultivable Protease-Producing Bacteria

JOURNAL Unpublished

REFERENCE 2 (bases 1 to 640)

AUTHORS Su,H., Huang,Q. and Yu,K.

TITLE Direct Submission

JOURNAL Submitted (11-MAR-2019) Coral Reef Research Center of China, School

of Marine Sciences, No. 100 Daxue Road, Xixiangtang District,

Nanning, Guangxi Zhuang Autonomous Region 530004, China

COMMENT ##Assembly-Data-START##

Sequencing Technology :: Sanger dideoxy sequencing

##Assembly-Data-END##

FEATURES Location/Qualifiers

source 1..640

/organism="Microbacterium sp."

/mol_type="genomic DNA"

/strain="sA9"

/isolation_source="coral"

/db_xref="taxon:51671"

rRNA <1..>640

/product="16S ribosomal RNA"

ORIGIN

1 acggtgaaca cggagcttgc tctgtgggat cagtggcgaa cgggtgagta acacgtgagc

61 aacctgcccc tgactctggg ataagcgctg gaaacggcgt ctaatactgg atatgtgacg

121 tgatcgcatg gtctgcgtct ggaaagaatt tcggttgggg atgggctcgc ggcctatcag

181 cttgttggtg aggtaatggc tcaccaaggc gtcgacgggt agccggcctg agagggtgac

241 cggccacact gggactgaga cacggcccag actcctacgg gaggcagcag tggggaatat

301 tgcacaatgg gcgcaagcct gatgcagcaa cgccgcgtga gggatgacgg ccttcgggtt

361 gtaaacctct tttagcaggg aagaagcgaa agtgacggta cctgcagaaa aagcgccggc

421 taactacgtg ccagcagccg cggtaatacg tagggcgcaa gcgttatccg gaattattgg

481 gcgtaaagag ctcgtaggcg gtttgtcgcg tctgctgtga aatccggagg ctcaacctcc

541 ggcctgcagt gggtacgggc agactagagt gcggtagggg agattggaat tcctggtgta

601 gcggtggaat gcgcagatat caggaggaac accgatggcg

//

LOCUS MK617632 640 bp DNA linear BCT 30-MAY-2019

DEFINITION Bacillus sp. (in: Bacteria) strain sA10 16S ribosomal RNA gene,

partial sequence.

ACCESSION MK617632

VERSION MK617632

KEYWORDS .

SOURCE Bacillus sp. (in: Bacteria)

ORGANISM Bacillus sp. (in: Bacteria)

Bacteria; Firmicutes; Bacilli; Bacillales; Bacillaceae; Bacillus.

REFERENCE 1 (bases 1 to 640)

AUTHORS Su,H., Huang,Q. and Yu,K.

TITLE Diversity of Cultivable Protease-Producing Bacteria

JOURNAL Unpublished

REFERENCE 2 (bases 1 to 640)

AUTHORS Su,H., Huang,Q. and Yu,K.

TITLE Direct Submission

JOURNAL Submitted (11-MAR-2019) Coral Reef Research Center of China, School

of Marine Sciences, No. 100 Daxue Road, Xixiangtang District,

Nanning, Guangxi Zhuang Autonomous Region 530004, China

COMMENT ##Assembly-Data-START##

Sequencing Technology :: Sanger dideoxy sequencing

##Assembly-Data-END##

FEATURES Location/Qualifiers

source 1..640

/organism="Bacillus sp. (in: Bacteria)"

/mol_type="genomic DNA"

/strain="sA10"

/isolation_source="coral"

/db_xref="taxon:1409"

rRNA <1..>640

/product="16S ribosomal RNA"

ORIGIN

1 ggacagatgg gagcttgctc cctgaagtca gcggcggacg ggtgagtaac acgtgggcaa

61 cctgcctgta agactgggat aactccggga aaccggggct aataccggat aattctttcc

121 ctcacatgag ggaaagctga aagatggttt cggctatcac ttacagatgg gcccgcggcg

181 cattagctag ttggtgaggt aacggctcac caaggcaacg atgcgtagcc gacctgagag

241 ggtgatcggc cacactggga ctgagacacg gcccagactc ctacgggagg cagcagtagg

301 gaatcttccg caatggacga aagtctgacg gagcaacgcc gcgtgagtga tgaaggtttt

361 cggatcgtaa aactctgttg ttagggaaga acaagtaccg gagtaactgc cggtaccttg

421 acggtaccta accagaaagc cacggctaac tacgtgccag cagccgcggt aatacgtagg

481 tggcaagcgt tgtccggaat tattgggcgt aaagcgcgcg caggcggttc cttaagtctg

541 atgtgaaagc ccccggctca accggggagg gtcattggaa actggggaac ttgagtgcag

601 aagagaagag tggaattcca cgtgtagcgg tgaaatgcgt

//

LOCUS MK617633 640 bp DNA linear BCT 30-MAY-2019

DEFINITION Bacillus sp. (in: Bacteria) strain sA11 16S ribosomal RNA gene,

partial sequence.

ACCESSION MK617633

VERSION MK617633

KEYWORDS .

SOURCE Bacillus sp. (in: Bacteria)

ORGANISM Bacillus sp. (in: Bacteria)

Bacteria; Firmicutes; Bacilli; Bacillales; Bacillaceae; Bacillus.

REFERENCE 1 (bases 1 to 640)

AUTHORS Su,H., Huang,Q. and Yu,K.

TITLE Diversity of Cultivable Protease-Producing Bacteria

JOURNAL Unpublished

REFERENCE 2 (bases 1 to 640)

AUTHORS Su,H., Huang,Q. and Yu,K.

TITLE Direct Submission

JOURNAL Submitted (11-MAR-2019) Coral Reef Research Center of China, School

of Marine Sciences, No. 100 Daxue Road, Xixiangtang District,

Nanning, Guangxi Zhuang Autonomous Region 530004, China

COMMENT ##Assembly-Data-START##

Sequencing Technology :: Sanger dideoxy sequencing

##Assembly-Data-END##

FEATURES Location/Qualifiers

source 1..640

/organism="Bacillus sp. (in: Bacteria)"

/mol_type="genomic DNA"

/strain="sA11"

/isolation_source="coral"

/db_xref="taxon:1409"

rRNA <1..>640

/product="16S ribosomal RNA"

ORIGIN

1 gacagatggg agcttgctcc ctgaagtcag cggcggacgg gtgagtaaca cgtgggcaac

61 ctgcctgtaa gactgggata actccgggaa accggggcta ataccggata attctttccc

121 tcacatgagg gaaagctgaa agatggtttc ggctatcact tacagatggg cccgcggcgc

181 attagctagt tggtgaggta acggctcacc aaggcaacga tgcgtagccg acctgagagg

241 gtgatcggcc acactgggac tgagacacgg cccagactcc tacgggaggc agcagtaggg

301 aatcttccgc aatggacgaa agtctgacgg agcaacgccg cgtgagtgat gaaggttttc

361 ggatcgtaaa actctgttgt tagggaagaa caagtaccgg agtaactgcc ggtaccttga

421 cggtacctaa ccagaaagcc acggctaact acgtgccagc agccgcggta atacgtaggt

481 ggcaagcgtt gtccggaatt attgggcgta aagcgcgcgc aggcggttcc ttaagtctga

541 tgtgaaagcc cccggctcaa ccggggaggg tcattggaaa ctggggaact tgagtgcaga

601 agagaagagt ggaattccac gtgtagcggt gaaatgcgta

//

LOCUS MK617634 640 bp DNA linear BCT 30-MAY-2019

DEFINITION Bacillus sp. (in: Bacteria) strain sA27 16S ribosomal RNA gene,

partial sequence.

ACCESSION MK617634

VERSION MK617634

KEYWORDS .

SOURCE Bacillus sp. (in: Bacteria)

ORGANISM Bacillus sp. (in: Bacteria)

Bacteria; Firmicutes; Bacilli; Bacillales; Bacillaceae; Bacillus.

REFERENCE 1 (bases 1 to 640)

AUTHORS Su,H., Huang,Q. and Yu,K.

TITLE Diversity of Cultivable Protease-Producing Bacteria

JOURNAL Unpublished

REFERENCE 2 (bases 1 to 640)

AUTHORS Su,H., Huang,Q. and Yu,K.

TITLE Direct Submission

JOURNAL Submitted (11-MAR-2019) Coral Reef Research Center of China, School

of Marine Sciences, No. 100 Daxue Road, Xixiangtang District,

Nanning, Guangxi Zhuang Autonomous Region 530004, China

COMMENT ##Assembly-Data-START##

Sequencing Technology :: Sanger dideoxy sequencing

##Assembly-Data-END##

FEATURES Location/Qualifiers

source 1..640

/organism="Bacillus sp. (in: Bacteria)"

/mol_type="genomic DNA"

/strain="sA27"

/isolation_source="coral"

/db_xref="taxon:1409"

rRNA <1..>640

/product="16S ribosomal RNA"

ORIGIN

1 gcggacagat gggagcttgc tccctgaagt cagcggcgga cgggtgagta acacgtgggc

61 aacctgcctg taagactggg ataactccgg gaaaccgggg ctaataccgg ataattcttt

121 ccctcacatg agggaaagct gaaagatggt ttcggctatc acttacagat gggcccgcgg

181 cgcattagct agttggtgag gtaacggctc accaaggcaa cgatgcgtag ccgacctgag

241 agggtgatcg gccacactgg gactgagaca cggcccagac tcctacggga ggcagcagta

301 gggaatcttc cgcaatggac gaaagtctga cggagcaacg ccgcgtgagt gatgaaggtt

361 ttcggatcgt aaaactctgt tgttagggaa gaacaagtac cggagtaact gccggtacct

421 tgacggtacc taaccagaaa gccacggcta actacgtgcc agcagccgcg gtaatacgta

481 ggtggcaagc gttgtccgga attattgggc gtaaagcgcg cgcaggcggt tccttaagtc

541 tgatgtgaaa gcccccggct caaccgggga gggtcattgg aaactgggga acttgagtgc

601 agaagagaag agtggaattc cacgtgtagc ggtgaaatgc

//

LOCUS MK617635 640 bp DNA linear BCT 30-MAY-2019

DEFINITION Bacillus sp. (in: Bacteria) strain sA50 16S ribosomal RNA gene,

partial sequence.

ACCESSION MK617635

VERSION MK617635

KEYWORDS .

SOURCE Bacillus sp. (in: Bacteria)

ORGANISM Bacillus sp. (in: Bacteria)

Bacteria; Firmicutes; Bacilli; Bacillales; Bacillaceae; Bacillus.

REFERENCE 1 (bases 1 to 640)

AUTHORS Su,H., Huang,Q. and Yu,K.

TITLE Diversity of Cultivable Protease-Producing Bacteria

JOURNAL Unpublished

REFERENCE 2 (bases 1 to 640)

AUTHORS Su,H., Huang,Q. and Yu,K.

TITLE Direct Submission

JOURNAL Submitted (11-MAR-2019) Coral Reef Research Center of China, School

of Marine Sciences, No. 100 Daxue Road, Xixiangtang District,

Nanning, Guangxi Zhuang Autonomous Region 530004, China

COMMENT ##Assembly-Data-START##

Sequencing Technology :: Sanger dideoxy sequencing

##Assembly-Data-END##

FEATURES Location/Qualifiers

source 1..640

/organism="Bacillus sp. (in: Bacteria)"

/mol_type="genomic DNA"

/strain="sA50"

/isolation_source="coral"

/db_xref="taxon:1409"

rRNA <1..>640

/product="16S ribosomal RNA"

ORIGIN

1 aatggattaa gagcttgctc ttatgaagtt agcggcggac gggtgagtaa cacgtgggta

61 acctgcccat aagactggga taactccggg aaaccggggc taataccgga taacattttg

121 aaccgcatgg ttcgaaattg aaaggcggct tcggctgtca cttatggatg gacccgcgtc

181 gcattagcta gttggtgagg taacggctca ccaaggcaac gatgcgtagc cgacctgaga

241 gggtgatcgg ccacactggg actgagacac ggcccagact cctacgggag gcagcagtag

301 ggaatcttcc gcaatggacg aaagtctgac ggagcaacgc cgcgtgagtg atgaaggctt

361 tcgggtcgta aaactctgtt gttagggaag aacaagtgct agttgaataa gctggcacct

421 tgacggtacc taaccagaaa gccacggcta actacgtgcc agcagccgcg gtaatacgta

481 ggtggcaagc gttatccgga attattgggc gtaaagcgcg cgcaggtggt ttcttaagtc

541 tgatgtgaaa gcccacggct caaccgtgga gggtcattgg aaactgggag acttgagtgc

601 agaagaggaa agtggaattc catgtgtagc ggtgaaatgc

//

LOCUS MK617636 640 bp DNA linear BCT 30-MAY-2019

DEFINITION Fictibacillus sp. strain sA51 16S ribosomal RNA gene, partial

sequence.

ACCESSION MK617636

VERSION MK617636

KEYWORDS .

SOURCE Fictibacillus sp.

ORGANISM Fictibacillus sp.

Bacteria; Firmicutes; Bacilli; Bacillales; Bacillaceae;

Fictibacillus.

REFERENCE 1 (bases 1 to 640)

AUTHORS Su,H., Huang,Q. and Yu,K.

TITLE Diversity of Cultivable Protease-Producing Bacteria

JOURNAL Unpublished

REFERENCE 2 (bases 1 to 640)

AUTHORS Su,H., Huang,Q. and Yu,K.

TITLE Direct Submission

JOURNAL Submitted (11-MAR-2019) Coral Reef Research Center of China, School

of Marine Sciences, No. 100 Daxue Road, Xixiangtang District,

Nanning, Guangxi Zhuang Autonomous Region 530004, China

COMMENT ##Assembly-Data-START##

Sequencing Technology :: Sanger dideoxy sequencing

##Assembly-Data-END##

FEATURES Location/Qualifiers

source 1..640

/organism="Fictibacillus sp."

/mol_type="genomic DNA"

/strain="sA51"

/isolation_source="coral"

/db_xref="taxon:1871617"

rRNA <1..>640

/product="16S ribosomal RNA"

ORIGIN

1 atgatgagga gcttgctcct ctgatttagc ggcggacggg tgagtaacac gtgggtaatc

61 tgcctgtaag acggggataa ctccgggaaa ccggggctaa taccggataa taagagaaga

121 agcatttctt ctttttgaaa gtcggtttcg gctgacactt acagatgagc ccgcggcgca

181 ttagctagtt ggtgaggtaa cggctcacca aggcgacgat gcgtagccga cctgagaggg

241 tgatcggcca cactgggact gagacacggc ccagactcct acgggaggca gcagtaggga

301 atcttcggca atgggcgaaa gcctgaccga gcaacgccgc gtgagcgatg aaggccttcg

361 ggtcgtaaag ctctgttgtt agagaagaac aagtacgaga gtaactgctc gtaccttgac

421 ggtacctaac cagaaagcca cggctaacta cgtgccagca gccgcggtaa tacgtaggtg

481 gcaagcgtta tccggaatta ttgggcgtaa agcgcgcgca ggcggtctct taagtctgat

541 gtgaaagccc acggctcaac cgtggagggt cattggaaac tgggagactt gagtgcagga

601 gagaaaagtg gaattccacg tgtagcggtg aaatgcgtag

//

LOCUS MK617637 640 bp DNA linear BCT 30-MAY-2019

DEFINITION Fictibacillus sp. strain sA57 16S ribosomal RNA gene, partial

sequence.

ACCESSION MK617637

VERSION MK617637

KEYWORDS .

SOURCE Fictibacillus sp.

ORGANISM Fictibacillus sp.

Bacteria; Firmicutes; Bacilli; Bacillales; Bacillaceae;

Fictibacillus.

REFERENCE 1 (bases 1 to 640)

AUTHORS Su,H., Huang,Q. and Yu,K.

TITLE Diversity of Cultivable Protease-Producing Bacteria

JOURNAL Unpublished

REFERENCE 2 (bases 1 to 640)

AUTHORS Su,H., Huang,Q. and Yu,K.

TITLE Direct Submission

JOURNAL Submitted (11-MAR-2019) Coral Reef Research Center of China, School

of Marine Sciences, No. 100 Daxue Road, Xixiangtang District,

Nanning, Guangxi Zhuang Autonomous Region 530004, China

COMMENT ##Assembly-Data-START##

Sequencing Technology :: Sanger dideoxy sequencing

##Assembly-Data-END##

FEATURES Location/Qualifiers

source 1..640

/organism="Fictibacillus sp."

/mol_type="genomic DNA"

/strain="sA57"

/isolation_source="coral"

/db_xref="taxon:1871617"

rRNA <1..>640

/product="16S ribosomal RNA"

ORIGIN

1 cgaatgatga ggagcttgct cctctgattt agcggcggac gggtgagtaa cacgtgggta

61 atctgcctgt aagacgggga taactccggg aaaccggggc taataccgga taataagaga

121 agaagcattt cttctttttg aaagtcggtt tcggctgaca cttacagatg agcccgcggc

181 gcattagcta gttggtgagg taacggctca ccaaggcgac gatgcgtagc cgacctgaga

241 gggtgatcgg ccacactggg actgagacac ggcccagact cctacgggag gcagcagtag

301 ggaatcttcg gcaatgggcg aaagcctgac cgagcaacgc cgcgtgagcg atgaaggcct

361 tcgggtcgta aagctctgtt gttagagaag aacaagtacg agagtaactg ctcgtacctt

421 gacggtacct aaccagaaag ccacggctaa ctacgtgcca gcagccgcgg taatacgtag

481 gtggcaagcg ttatccggaa ttattgggcg taaagcgcgc gcaggcggtc tcttaagtct

541 gatgtgaaag cccacggctc aaccgtggag ggtcattgga aactgggaga cttgagtgca

601 ggagagaaaa gtggaattcc acgtgtagcg gtgaaatgcg

//

LOCUS MK617638 640 bp DNA linear BCT 30-MAY-2019

DEFINITION Microbacterium sp. strain sA61 16S ribosomal RNA gene, partial

sequence.

ACCESSION MK617638

VERSION MK617638

KEYWORDS .

SOURCE Microbacterium sp.

ORGANISM Microbacterium sp.

Bacteria; Actinobacteria; Micrococcales; Microbacteriaceae;

Microbacterium.

REFERENCE 1 (bases 1 to 640)

AUTHORS Su,H., Huang,Q. and Yu,K.

TITLE Diversity of Cultivable Protease-Producing Bacteria

JOURNAL Unpublished

REFERENCE 2 (bases 1 to 640)

AUTHORS Su,H., Huang,Q. and Yu,K.

TITLE Direct Submission

JOURNAL Submitted (11-MAR-2019) Coral Reef Research Center of China, School

of Marine Sciences, No. 100 Daxue Road, Xixiangtang District,

Nanning, Guangxi Zhuang Autonomous Region 530004, China

COMMENT ##Assembly-Data-START##

Sequencing Technology :: Sanger dideoxy sequencing

##Assembly-Data-END##

FEATURES Location/Qualifiers

source 1..640

/organism="Microbacterium sp."

/mol_type="genomic DNA"

/strain="sA61"

/isolation_source="coral"

/db_xref="taxon:51671"

rRNA <1..>640

/product="16S ribosomal RNA"

ORIGIN

1 gtgaagcagg agcttgctct tgtggatcag tggcgaacgg gtgagtaaca cgtgagcaac

61 ctgcccctga ctctgggata agcgctggaa acggcgtcta atactggata tgtgacgtga

121 ccgcatggtc tgcgtttgga aagatttttc ggttggggat gggctcgcgg cctatcagct

181 tgttggtgag gtaatggctc accaaggcgt cgacgggtag ccggcctgag agggtgaccg

241 gccacactgg gactgagaca cggcccagac tcctacggga ggcagcagtg gggaatattg

301 cacaatgggc gaaagcctga tgcagcaacg ccgcgtgagg gatgacggcc ttcgggttgt

361 aaacctcttt tagcagggaa gaagcgaaag tgacggtacc tgcagaaaaa gcgccggcta

421 actacgtgcc agcagccgcg gtaatacgta gggcgcaagc gttatccgga attattgggc

481 gtaaagagct cgtaggcggt ttgtcgcgtc tgctgtgaaa tcccgaggct caacctcggg

541 cctgcagtgg gtacgggcag actagagtgc ggtaggggag attggaattc ctggtgtagc

601 ggtggaatgc gcagatatca ggaggaacac cgatggcgaa

//

LOCUS MK617639 640 bp DNA linear BCT 30-MAY-2019

DEFINITION Vibrio sp. strain sB2a 16S ribosomal RNA gene, partial sequence.

ACCESSION MK617639

VERSION MK617639

KEYWORDS .

SOURCE Vibrio sp.

ORGANISM Vibrio sp.

Bacteria; Proteobacteria; Gammaproteobacteria; Vibrionales;

Vibrionaceae; Vibrio.

REFERENCE 1 (bases 1 to 640)

AUTHORS Su,H., Huang,Q. and Yu,K.

TITLE Diversity of Cultivable Protease-Producing Bacteria

JOURNAL Unpublished

REFERENCE 2 (bases 1 to 640)

AUTHORS Su,H., Huang,Q. and Yu,K.

TITLE Direct Submission

JOURNAL Submitted (11-MAR-2019) Coral Reef Research Center of China, School

of Marine Sciences, No. 100 Daxue Road, Xixiangtang District,

Nanning, Guangxi Zhuang Autonomous Region 530004, China

COMMENT ##Assembly-Data-START##

Sequencing Technology :: Sanger dideoxy sequencing

##Assembly-Data-END##

FEATURES Location/Qualifiers

source 1..640

/organism="Vibrio sp."

/mol_type="genomic DNA"

/strain="sB2a"

/isolation_source="coral"

/db_xref="taxon:678"

rRNA <1..>640

/product="16S ribosomal RNA"

ORIGIN

1 cgagttatct gaaccttcgg ggaacgataa cggcgtcgag cggcggacgg gtgagtaatg

61 cctaggaaat tgccctgatg tgggggataa ccattggaaa cgatggctaa taccgcatga

121 tgcctacggg ccaaagaggg ggaccttcgg gcctctcgcg tcaggatatg cctaggtggg

181 attagctagt tggtgaggta agggctcacc aaggcgacga tccctagctg gtctgagagg

241 atgatcagcc acactggaac tgagacacgg tccagactcc tacgggaggc agcagtgggg

301 aatattgcac aatgggcgca agcctgatgc agccatgccg cgtgtgtgaa gaaggccttc

361 gggttgtaaa gcactttcag tcgtgaggaa ggtggtgtag ttaatagctg cattatttga

421 cgttagcgac agaagaagca ccggctaact ccgtgccagc agccgcggta atacggaggg

481 tgcgagcgtt aatcggaatt actgggcgta aagcgcatgc aggtggtttg ttaagtcaga

541 tgtgaaagcc cggggctcaa cctcggaata gcatttgaaa ctggcagact agagtactgt

601 agaggggggt agaatttcag gtgtagcggt gaaatgcgta

//

LOCUS MK617640 640 bp DNA linear BCT 30-MAY-2019

DEFINITION Fictibacillus sp. strain sB10B 16S ribosomal RNA gene, partial

sequence.

ACCESSION MK617640

VERSION MK617640

KEYWORDS .

SOURCE Fictibacillus sp.

ORGANISM Fictibacillus sp.

Bacteria; Firmicutes; Bacilli; Bacillales; Bacillaceae;

Fictibacillus.

REFERENCE 1 (bases 1 to 640)

AUTHORS Su,H., Huang,Q. and Yu,K.

TITLE Diversity of Cultivable Protease-Producing Bacteria

JOURNAL Unpublished

REFERENCE 2 (bases 1 to 640)

AUTHORS Su,H., Huang,Q. and Yu,K.

TITLE Direct Submission

JOURNAL Submitted (11-MAR-2019) Coral Reef Research Center of China, School

of Marine Sciences, No. 100 Daxue Road, Xixiangtang District,

Nanning, Guangxi Zhuang Autonomous Region 530004, China

COMMENT ##Assembly-Data-START##

Sequencing Technology :: Sanger dideoxy sequencing

##Assembly-Data-END##

FEATURES Location/Qualifiers

source 1..640

/organism="Fictibacillus sp."

/mol_type="genomic DNA"

/strain="sB10B"

/isolation_source="coral"

/db_xref="taxon:1871617"

rRNA <1..>640

/product="16S ribosomal RNA"

ORIGIN

1 gatgaggagc ttgctcctct gatttagcgg cggacgggtg agtaacacgt gggtaatctg

61 cctgtaagac ggggataact ccgggaaacc ggggctaata ccggataata agagaagaag

121 catttcttct ttttgaaagt cggtttcggc tgacacttac agatgagccc gcggcgcatt

181 agctagttgg tgaggtaacg gctcaccaag gcgacgatgc gtagccgacc tgagagggtg

241 atcggccaca ctgggactga gacacggccc agactcctac gggaggcagc agtagggaat

301 cttcggcaat gggcgaaagc ctgaccgagc aacgccgcgt gagcgatgaa ggccttcggg

361 tcgtaaagct ctgttgttag agaagaacaa gtacgagagt aactgctcgt accttgacgg

421 tacctaacca gaaagccacg gctaactacg tgccagcagc cgcggtaata cgtaggtggc

481 aagcgttatc cggaattatt gggcgtaaag cgcgcgcagg cggtctctta agtctgatgt

541 gaaagcccac ggctcaaccg tggagggtca ttggaaactg ggagacttga gtgcaggaga

601 gaaaagtgga attccacgtg tagcggtgaa atgcgtagag

//

LOCUS MK617641 640 bp DNA linear BCT 30-MAY-2019

DEFINITION Vibrio sp. strain sB12 16S ribosomal RNA gene, partial sequence.

ACCESSION MK617641

VERSION MK617641

KEYWORDS .

SOURCE Vibrio sp.

ORGANISM Vibrio sp.

Bacteria; Proteobacteria; Gammaproteobacteria; Vibrionales;

Vibrionaceae; Vibrio.

REFERENCE 1 (bases 1 to 640)

AUTHORS Su,H., Huang,Q. and Yu,K.

TITLE Diversity of Cultivable Protease-Producing Bacteria

JOURNAL Unpublished

REFERENCE 2 (bases 1 to 640)

AUTHORS Su,H., Huang,Q. and Yu,K.

TITLE Direct Submission

JOURNAL Submitted (11-MAR-2019) Coral Reef Research Center of China, School

of Marine Sciences, No. 100 Daxue Road, Xixiangtang District,

Nanning, Guangxi Zhuang Autonomous Region 530004, China

COMMENT ##Assembly-Data-START##

Sequencing Technology :: Sanger dideoxy sequencing

##Assembly-Data-END##

FEATURES Location/Qualifiers

source 1..640

/organism="Vibrio sp."

/mol_type="genomic DNA"

/strain="sB12"

/isolation_source="coral"

/db_xref="taxon:678"

rRNA <1..>640

/product="16S ribosomal RNA"

ORIGIN

1 acgagttatc tgaaccttcg gggaacgata acggcgtcga gcggcggacg ggtgagtaat

61 gcctaggaaa ttgccctgat gtgggggata accattggaa acgatggcta ataccgcatg

121 atgcctacgg gccaaagagg gggaccttcg ggcctctcgc gtcaggatat gcctaggtgg

181 gattagctag ttggtgaggt aagggctcac caaggcgacg atccctagct ggtctgagag

241 gatgatcagc cacactggaa ctgagacacg gtccagactc ctacgggagg cagcagtggg

301 gaatattgca caatgggcgc aagcctgatg cagccatgcc gcgtgtgtga agaaggcctt

361 cgggttgtaa agcactttca gtcgtgagga aggtggtgta gttaatagct gcattatttg

421 acgttagcga cagaagaagc accggctaac tccgtgccag cagccgcggt aatacggagg

481 gtgcgagcgt taatcggaat tactgggcgt aaagcgcatg caggtggttt gttaagtcag

541 atgtgaaagc ccggggctca acctcggaat agcatttgaa actggcagac tagagtactg

601 tagagggggg tagaatttca ggtgtagcgg tgaaatgcgt

//

LOCUS MK617642 640 bp DNA linear BCT 30-MAY-2019

DEFINITION Bacillus sp. (in: Bacteria) strain sB17 16S ribosomal RNA gene,

partial sequence.

ACCESSION MK617642

VERSION MK617642

KEYWORDS .

SOURCE Bacillus sp. (in: Bacteria)

ORGANISM Bacillus sp. (in: Bacteria)

Bacteria; Firmicutes; Bacilli; Bacillales; Bacillaceae; Bacillus.

REFERENCE 1 (bases 1 to 640)

AUTHORS Su,H., Huang,Q. and Yu,K.

TITLE Diversity of Cultivable Protease-Producing Bacteria

JOURNAL Unpublished

REFERENCE 2 (bases 1 to 640)

AUTHORS Su,H., Huang,Q. and Yu,K.

TITLE Direct Submission

JOURNAL Submitted (11-MAR-2019) Coral Reef Research Center of China, School

of Marine Sciences, No. 100 Daxue Road, Xixiangtang District,

Nanning, Guangxi Zhuang Autonomous Region 530004, China

COMMENT ##Assembly-Data-START##

Sequencing Technology :: Sanger dideoxy sequencing

##Assembly-Data-END##

FEATURES Location/Qualifiers

source 1..640

/organism="Bacillus sp. (in: Bacteria)"

/mol_type="genomic DNA"

/strain="sB17"

/isolation_source="coral"

/db_xref="taxon:1409"

rRNA <1..>640

/product="16S ribosomal RNA"

ORIGIN

1 gacagatggg agcttgctcc ctgaagtcag cggcggacgg gtgagtaaca cgtgggcaac

61 ctgcctgtaa gactgggata actccgggaa accggggcta ataccggata attctttccc

121 tcacatgagg gaaagctgaa agatggtttc ggctatcact tacagatggg cccgcggcgc

181 attagctagt tggtgaggta acggctcacc aaggcaacga tgcgtagccg acctgagagg

241 gtgatcggcc acactgggac tgagacacgg cccagactcc tacgggaggc agcagtaggg

301 aatcttccgc aatggacgaa agtctgacgg agcaacgccg cgtgagtgat gaaggttttc

361 ggatcgtaaa actctgttgt tagggaagaa caagtaccgg agtaactgcc ggtaccttga

421 cggtacctaa ccagaaagcc acggctaact acgtgccagc agccgcggta atacgtaggt

481 ggcaagcgtt gtccggaatt attgggcgta aagcgcgcgc aggcggttcc ttaagtctga

541 tgtgaaagcc cccggctcaa ccggggaggg tcattggaaa ctggggaact tgagtgcaga

601 agagaagagt ggaattccac gtgtagcggt gaaatgcgta

//

LOCUS MK617643 640 bp DNA linear BCT 30-MAY-2019

DEFINITION Fictibacillus sp. strain sB33b 16S ribosomal RNA gene, partial

sequence.

ACCESSION MK617643

VERSION MK617643

KEYWORDS .

SOURCE Fictibacillus sp.

ORGANISM Fictibacillus sp.

Bacteria; Firmicutes; Bacilli; Bacillales; Bacillaceae;

Fictibacillus.

REFERENCE 1 (bases 1 to 640)

AUTHORS Su,H., Huang,Q. and Yu,K.

TITLE Diversity of Cultivable Protease-Producing Bacteria

JOURNAL Unpublished

REFERENCE 2 (bases 1 to 640)

AUTHORS Su,H., Huang,Q. and Yu,K.

TITLE Direct Submission

JOURNAL Submitted (11-MAR-2019) Coral Reef Research Center of China, School

of Marine Sciences, No. 100 Daxue Road, Xixiangtang District,

Nanning, Guangxi Zhuang Autonomous Region 530004, China

COMMENT ##Assembly-Data-START##

Sequencing Technology :: Sanger dideoxy sequencing

##Assembly-Data-END##

FEATURES Location/Qualifiers

source 1..640

/organism="Fictibacillus sp."

/mol_type="genomic DNA"

/strain="sB33b"

/isolation_source="coral"

/db_xref="taxon:1871617"

rRNA <1..>640

/product="16S ribosomal RNA"

ORIGIN

1 aatgatgagg agcttgctcc tctgatttag cggcggacgg gtgagtaaca cgtgggtaat

61 ctgcctgtaa gacggggata actccgggaa accggggcta ataccggata ataagagaag

121 aagcatttct tctttttgaa agtcggtttc ggctgacact tacagatgag cccgcggcgc

181 attagctagt tggtgaggta acggctcacc aaggcgacga tgcgtagccg acctgagagg

241 gtgatcggcc acactgggac tgagacacgg cccagactcc tacgggaggc agcagtaggg

301 aatcttcggc aatgggcgaa agcctgaccg agcaacgccg cgtgagcgat gaaggccttc

361 gggtcgtaaa gctctgttgt tagagaagaa caagtacgag agtaactgct cgtaccttga

421 cggtacctaa ccagaaagcc acggctaact acgtgccagc agccgcggta atacgtaggt

481 ggcaagcgtt atccggaatt attgggcgta aagcgcgcgc aggcggtctc ttaagtctga

541 tgtgaaagcc cacggctcaa ccgtggaggg tcattggaaa ctgggagact tgagtgcagg

601 agagaaaagt ggaattccac gtgtagcggt gaaatgcgta

//

LOCUS MK617644 640 bp DNA linear BCT 30-MAY-2019

DEFINITION Fictibacillus sp. strain sB66 16S ribosomal RNA gene, partial

sequence.

ACCESSION MK617644

VERSION MK617644

KEYWORDS .

SOURCE Fictibacillus sp.

ORGANISM Fictibacillus sp.

Bacteria; Firmicutes; Bacilli; Bacillales; Bacillaceae;

Fictibacillus.

REFERENCE 1 (bases 1 to 640)

AUTHORS Su,H., Huang,Q. and Yu,K.

TITLE Diversity of Cultivable Protease-Producing Bacteria

JOURNAL Unpublished

REFERENCE 2 (bases 1 to 640)

AUTHORS Su,H., Huang,Q. and Yu,K.

TITLE Direct Submission

JOURNAL Submitted (11-MAR-2019) Coral Reef Research Center of China, School

of Marine Sciences, No. 100 Daxue Road, Xixiangtang District,

Nanning, Guangxi Zhuang Autonomous Region 530004, China

COMMENT ##Assembly-Data-START##

Sequencing Technology :: Sanger dideoxy sequencing

##Assembly-Data-END##

FEATURES Location/Qualifiers

source 1..640

/organism="Fictibacillus sp."

/mol_type="genomic DNA"

/strain="sB66"

/isolation_source="coral"

/db_xref="taxon:1871617"

rRNA <1..>640

/product="16S ribosomal RNA"

ORIGIN

1 cgaatgatga ggagcttgct cctctgattt agcggcggac gggtgagtaa cacgtgggta

61 atctgcctgt aagacgggga taactccggg aaaccggggc taataccgga taataagaga

121 agaagcattt cttctttttg aaagtcggtt tcggctgaca cttacagatg agcccgcggc

181 gcattagcta gttggtgagg taacggctca ccaaggcgac gatgcgtagc cgacctgaga

241 gggtgatcgg ccacactggg actgagacac ggcccagact cctacgggag gcagcagtag

301 ggaatcttcg gcaatgggcg aaagcctgac cgagcaacgc cgcgtgagcg atgaaggcct

361 tcgggtcgta aagctctgtt gttagagaag aacaagtacg agagtaactg ctcgtacctt

421 gacggtacct aaccagaaag ccacggctaa ctacgtgcca gcagccgcgg taatacgtag

481 gtggcaagcg ttatccggaa ttattgggcg taaagcgcgc gcaggcggtc tcttaagtct

541 gatgtgaaag cccacggctc aaccgtggag ggtcattgga aactgggaga cttgagtgca

601 ggagagaaaa gtggaattcc acgtgtagcg gtgaaatgcg

//

LOCUS MK617645 640 bp DNA linear BCT 30-MAY-2019

DEFINITION Microbulbifer sp. strain sC1 16S ribosomal RNA gene, partial

sequence.

ACCESSION MK617645

VERSION MK617645

KEYWORDS .

SOURCE Microbulbifer sp.

ORGANISM Microbulbifer sp.

Bacteria; Proteobacteria; Gammaproteobacteria; Cellvibrionales;

Microbulbiferaceae; Microbulbifer.

REFERENCE 1 (bases 1 to 640)

AUTHORS Su,H., Huang,Q. and Yu,K.

TITLE Diversity of Cultivable Protease-Producing Bacteria

JOURNAL Unpublished

REFERENCE 2 (bases 1 to 640)

AUTHORS Su,H., Huang,Q. and Yu,K.

TITLE Direct Submission

JOURNAL Submitted (11-MAR-2019) Coral Reef Research Center of China, School

of Marine Sciences, No. 100 Daxue Road, Xixiangtang District,

Nanning, Guangxi Zhuang Autonomous Region 530004, China

COMMENT ##Assembly-Data-START##

Sequencing Technology :: Sanger dideoxy sequencing

##Assembly-Data-END##

FEATURES Location/Qualifiers

source 1..640

/organism="Microbulbifer sp."

/mol_type="genomic DNA"

/strain="sC1"

/isolation_source="coral"

/db_xref="taxon:1908541"

rRNA <1..>640

/product="16S ribosomal RNA"

ORIGIN

1 aaagttcttc ggaacgagta aagcggcgga cgggtgagta acgcgtggga aattgcccag

61 tagtggggga caacattctg aaacggatgc taataccgca tacgccctac gggggaaagc

121 aggggatctt ccgaccttgc gctattggat atgcccgcgt cggattaact agttggtgaa

181 gtaatggctc accaaggcaa cgatccgtac ctggtctgag aggatgatca gccacactgg

241 gactgagaca cggcccggac tcctacggga ggcggcagtg gggaatattg gacaatgggc

301 ggaagcctga tccagccatg ccgcgtgtgt gaagaaggcc ctagggttgt aaagcacttt

361 cattagggag gaaggcctta aagttaatac ctttgaggat tgacgttacc tacagaacaa

421 gcaccggcta actccgtgcc agcagccgcg gtaatacgga gggtgcaagc gttaatcgga

481 attactgggc gtaaagcgcg cgtaggcggt tagttaagct ggatgtgaaa gccccgggct

541 caacctggga actgcattca gaactggctg gctagagtac gagagagggt agtggaattt

601 cctgtgtagc ggtgactgcg tagatatagg aaggaacatc

//

LOCUS MK617646 640 bp DNA linear BCT 30-MAY-2019

DEFINITION Aquimarina sp. strain sC2 16S ribosomal RNA gene, partial sequence.

ACCESSION MK617646

VERSION MK617646

KEYWORDS .

SOURCE Aquimarina sp.

ORGANISM Aquimarina sp.

Bacteria; Bacteroidetes; Flavobacteriia; Flavobacteriales;

Flavobacteriaceae; Aquimarina.

REFERENCE 1 (bases 1 to 640)

AUTHORS Su,H., Huang,Q. and Yu,K.

TITLE Diversity of Cultivable Protease-Producing Bacteria

JOURNAL Unpublished

REFERENCE 2 (bases 1 to 640)

AUTHORS Su,H., Huang,Q. and Yu,K.

TITLE Direct Submission

JOURNAL Submitted (11-MAR-2019) Coral Reef Research Center of China, School

of Marine Sciences, No. 100 Daxue Road, Xixiangtang District,

Nanning, Guangxi Zhuang Autonomous Region 530004, China

COMMENT ##Assembly-Data-START##

Sequencing Technology :: Sanger dideoxy sequencing

##Assembly-Data-END##

FEATURES Location/Qualifiers

source 1..640

/organism="Aquimarina sp."

/mol_type="genomic DNA"

/strain="sC2"

/isolation_source="coral"

/db_xref="taxon:1872586"

rRNA <1..>640

/product="16S ribosomal RNA"

ORIGIN

1 ggtaacatag ttgcttgcaa ctgatgacga ccggcgcacg ggtgcgtaac gcgtatagaa

61 cctaccttat agtaagggat agcccagaga aatttggatt aataccttat agtatcgttt

121 agatgcatat ttaaatgatt aaagatttat cgctataaga tggctatgcg ttctattagc

181 tagttggtat ggtaacggca taccaaggct acgatagata ggggtcctga gagggagatc

241 ccccacactg gtactgagac acggaccaga ctcctacggg aggcagcagt gaggaatatt

301 ggacaatgga ggcaactctg atccagccat gccgcgtgta ggaagactgc cctatgggtt

361 gtaaactact tttatagagg aagaaaccat tccacgtgtg gaatgctgac ggtactctac

421 gaataaggat cggctaactc cgtgccagca gccgcggtaa tacggaggat ccaagcgtta

481 tccggaatca ttgggtttaa agggtccgta ggcggtttag taagtcagtg gtgaaagttt

541 tcggctcaac cggaaaattg ccattgatac tgcaagactt gaattattgt gaagtggtta

601 gaatgtgtag tgtagcggtg aaatgcatag atattacaca

//

LOCUS MK617647 640 bp DNA linear BCT 30-MAY-2019

DEFINITION Pseudovibrio sp. strain sC3 16S ribosomal RNA gene, partial

sequence.

ACCESSION MK617647

VERSION MK617647

KEYWORDS .

SOURCE Pseudovibrio sp.

ORGANISM Pseudovibrio sp.

Bacteria; Proteobacteria; Alphaproteobacteria; Rhodobacterales;

Rhodobacteraceae; Pseudovibrio.

REFERENCE 1 (bases 1 to 640)

AUTHORS Su,H., Huang,Q. and Yu,K.

TITLE Diversity of Cultivable Protease-Producing Bacteria

JOURNAL Unpublished

REFERENCE 2 (bases 1 to 640)

AUTHORS Su,H., Huang,Q. and Yu,K.

TITLE Direct Submission

JOURNAL Submitted (11-MAR-2019) Coral Reef Research Center of China, School

of Marine Sciences, No. 100 Daxue Road, Xixiangtang District,

Nanning, Guangxi Zhuang Autonomous Region 530004, China

COMMENT ##Assembly-Data-START##

Sequencing Technology :: Sanger dideoxy sequencing

##Assembly-Data-END##

FEATURES Location/Qualifiers

source 1..640

/organism="Pseudovibrio sp."

/mol_type="genomic DNA"

/strain="sC3"

/isolation_source="coral"

/db_xref="taxon:1909297"

rRNA <1..>640

/product="16S ribosomal RNA"

ORIGIN

1 gatccttcgg gattagtggc agacgggtga gtaacgcgtg ggaagctacc ttgtggtagg

61 gaacaacagt tggaaacgac tgctaatacc ctatgagccc tatgggggaa agatttatcg

121 ccatgagatg tgcccgcgtt agattagcta gttggtaagg taatggctta ccaaggcgac

181 gatctatagc tggtctgaga ggatgatcag ccacactggg actgagacac ggcccagact

241 cctacgggag gcagcagtgg ggaatattgg acaatggggg caaccctgat ccagccatgc

301 cgcgtgagtg atgacggcct tagggttgta aagctctttc agcagtgaag ataatgacat

361 taactgcaga agaagccccg gctaacttcg tgccagcagc cgcggtaata cgaagggggc

421 tagcgttgtt cggaatcact gggcgtaaag cgtacgtagg cggactgatc agtcaggggt

481 gaaatcccgg ggctcaaccc cggaactgcc tttgatactg tcagtcttga gatcgagaga

541 ggtgagtgga actccgagtg tagaggtgaa attcgtagat attcggaaga acaccagtgg

601 cgaaggcggc tcactggctc gatactgacg ctgaggtacg

//

LOCUS MK617648 640 bp DNA linear BCT 30-MAY-2019

DEFINITION Pseudovibrio sp. strain sC4 16S ribosomal RNA gene, partial

sequence.

ACCESSION MK617648

VERSION MK617648

KEYWORDS .

SOURCE Pseudovibrio sp.

ORGANISM Pseudovibrio sp.

Bacteria; Proteobacteria; Alphaproteobacteria; Rhodobacterales;

Rhodobacteraceae; Pseudovibrio.

REFERENCE 1 (bases 1 to 640)

AUTHORS Su,H., Huang,Q. and Yu,K.

TITLE Diversity of Cultivable Protease-Producing Bacteria

JOURNAL Unpublished

REFERENCE 2 (bases 1 to 640)

AUTHORS Su,H., Huang,Q. and Yu,K.

TITLE Direct Submission

JOURNAL Submitted (11-MAR-2019) Coral Reef Research Center of China, School

of Marine Sciences, No. 100 Daxue Road, Xixiangtang District,

Nanning, Guangxi Zhuang Autonomous Region 530004, China

COMMENT ##Assembly-Data-START##

Sequencing Technology :: Sanger dideoxy sequencing

##Assembly-Data-END##

FEATURES Location/Qualifiers

source 1..640

/organism="Pseudovibrio sp."

/mol_type="genomic DNA"

/strain="sC4"

/isolation_source="coral"

/db_xref="taxon:1909297"

rRNA <1..>640

/product="16S ribosomal RNA"

ORIGIN

1 attcttcgga attagtggca gacgggtgag taacgcgtgg gaagctacct tgtggtaggg

61 aacaacagtt ggaaacgact gctaataccc tatgagccct atgggggaaa gatttatcgc

121 catgagatgt gcccgcgtta gattagctag ttggtaaggt aatggcttac caaggcgacg

181 atctatagct ggtctgagag gatgatcagc cacactggga ctgagacacg gcccagactc

241 ctacgggagg cagcagtggg gaatattgga caatgggggc aaccctgatc cagccatgcc

301 gcgtgagtga tgacggcctt agggttgtaa agctctttca gcagtgaaga taatgacatt

361 aactgcagaa gaagccccgg ctaacttcgt gccagcagcc gcggtaatac gaagggggct

421 agcgttgttc ggaatcactg ggcgtaaagc gtacgtaggc ggactgatca gtcaggggtg

481 aaatcccggg gctcaacccc ggaactgcct ttgatactgt cagtcttgag atcgagagag

541 gtgagtggaa ctccgagtgt agaggtgaaa ttcgtagata ttcggaagaa caccagtggc

601 gaaggcggct cactggctcg atactgacgc tgaggtacga

//

LOCUS MK617649 640 bp DNA linear BCT 30-MAY-2019

DEFINITION Pseudovibrio sp. strain sC5 16S ribosomal RNA gene, partial

sequence.

ACCESSION MK617649

VERSION MK617649

KEYWORDS .

SOURCE Pseudovibrio sp.

ORGANISM Pseudovibrio sp.

Bacteria; Proteobacteria; Alphaproteobacteria; Rhodobacterales;

Rhodobacteraceae; Pseudovibrio.

REFERENCE 1 (bases 1 to 640)

AUTHORS Su,H., Huang,Q. and Yu,K.

TITLE Diversity of Cultivable Protease-Producing Bacteria

JOURNAL Unpublished

REFERENCE 2 (bases 1 to 640)

AUTHORS Su,H., Huang,Q. and Yu,K.

TITLE Direct Submission

JOURNAL Submitted (11-MAR-2019) Coral Reef Research Center of China, School

of Marine Sciences, No. 100 Daxue Road, Xixiangtang District,

Nanning, Guangxi Zhuang Autonomous Region 530004, China

COMMENT ##Assembly-Data-START##

Sequencing Technology :: Sanger dideoxy sequencing

##Assembly-Data-END##

FEATURES Location/Qualifiers

source 1..640

/organism="Pseudovibrio sp."

/mol_type="genomic DNA"

/strain="sC5"

/isolation_source="coral"

/db_xref="taxon:1909297"

rRNA <1..>640

/product="16S ribosomal RNA"

ORIGIN

1 atccttcggg attagtggca gacgggtgag taacgcgtgg gaagctacct tgtggtaggg

61 aacaacagtt ggaaacgact gctaataccc tatgagccct atgggggaaa gatttatcgc

121 catgagatgt gcccgcgtta gattagctag ttggtaaggt aatggcttac caaggcgacg

181 atctatagct ggtctgagag gatgatcagc cacactggga ctgagacacg gcccagactc

241 ctacgggagg cagcagtggg gaatattgga caatgggggc aaccctgatc cagccatgcc

301 gcgtgagtga tgacggcctt agggttgtaa agctctttca gcagtgaaga taatgacatt

361 aactgcagaa gaagccccgg ctaacttcgt gccagcagcc gcggtaatac gaagggggct

421 agcgttgttc ggaatcactg ggcgtaaagc gtacgtaggc ggactgatca gtcaggggtg

481 aaatcccggg gctcaacccc ggaactgcct ttgatactgt cagtcttgag atcgagagag

541 gtgagtggaa ctccgagtgt agaggtgaaa ttcgtagata ttcggaagaa caccagtggc

601 gaaggcggct cactggctcg atactgacgc tgaggtacga

//

LOCUS MK617650 640 bp DNA linear BCT 30-MAY-2019

DEFINITION Pantoea sp. strain sC6 16S ribosomal RNA gene, partial sequence.

ACCESSION MK617650

VERSION MK617650

KEYWORDS .

SOURCE Pantoea sp.

ORGANISM Pantoea sp.

Bacteria; Proteobacteria; Gammaproteobacteria; Enterobacterales;

Erwiniaceae; Pantoea.

REFERENCE 1 (bases 1 to 640)

AUTHORS Su,H., Huang,Q. and Yu,K.

TITLE Diversity of Cultivable Protease-Producing Bacteria

JOURNAL Unpublished

REFERENCE 2 (bases 1 to 640)

AUTHORS Su,H., Huang,Q. and Yu,K.

TITLE Direct Submission

JOURNAL Submitted (11-MAR-2019) Coral Reef Research Center of China, School

of Marine Sciences, No. 100 Daxue Road, Xixiangtang District,

Nanning, Guangxi Zhuang Autonomous Region 530004, China

COMMENT ##Assembly-Data-START##

Sequencing Technology :: Sanger dideoxy sequencing

##Assembly-Data-END##

FEATURES Location/Qualifiers

source 1..640

/organism="Pantoea sp."

/mol_type="genomic DNA"

/strain="sC6"

/isolation_source="coral"

/db_xref="taxon:69393"

rRNA <1..>640

/product="16S ribosomal RNA"

ORIGIN

1 ggcagcacag aagagcttgc tctttgggtg gcgagtggcg gacgggtgag taatgtctgg

61 gaaactgccc gatggagggg gataactact ggaaacggta gctaataccg cataacgtcg

121 caagaccaaa gtgggggacc ttcgggcctc acaccatcgg atgtgcccag atgggattag

181 ctagtaggtg gggtaacggc tcacctaggc gacgatccct agctggtctg agaggatgac

241 cagccacact ggaactgaga cacggtccag actcctacgg gaggcagcag tggggaatat

301 tgcacaatgg gcgcaagcct gatgcagcca tgccgcgtgt atgaagaagg ccttcgggtt

361 gtaaagtact ttcagcgggg aggaaggcga cgcggttaat aaccgcgtcg attgacgtta

421 cccgcagaag aagcaccggc taactccgtg ccagcagccg cggtaatacg gagggtgcaa

481 gcgttaatcg gaattactgg gcgtaaagcg cacgcaggcg gtctgtcaag tcggatgtga

541 aatccccggg cttaacctgg gaactgcatt cgaaactggc aggctagagt cttgtagagg

601 ggggtagaat tccaggtgta gcggtgaaat gcgtagagat

//

LOCUS MK617651 640 bp DNA linear BCT 30-MAY-2019

DEFINITION Pseudovibrio sp. strain sC7 16S ribosomal RNA gene, partial

sequence.

ACCESSION MK617651

VERSION MK617651

KEYWORDS .

SOURCE Pseudovibrio sp.

ORGANISM Pseudovibrio sp.

Bacteria; Proteobacteria; Alphaproteobacteria; Rhodobacterales;

Rhodobacteraceae; Pseudovibrio.

REFERENCE 1 (bases 1 to 640)

AUTHORS Su,H., Huang,Q. and Yu,K.

TITLE Diversity of Cultivable Protease-Producing Bacteria

JOURNAL Unpublished

REFERENCE 2 (bases 1 to 640)

AUTHORS Su,H., Huang,Q. and Yu,K.

TITLE Direct Submission

JOURNAL Submitted (11-MAR-2019) Coral Reef Research Center of China, School

of Marine Sciences, No. 100 Daxue Road, Xixiangtang District,

Nanning, Guangxi Zhuang Autonomous Region 530004, China

COMMENT ##Assembly-Data-START##

Sequencing Technology :: Sanger dideoxy sequencing

##Assembly-Data-END##

FEATURES Location/Qualifiers

source 1..640

/organism="Pseudovibrio sp."

/mol_type="genomic DNA"

/strain="sC7"

/isolation_source="coral"

/db_xref="taxon:1909297"

rRNA <1..>640

/product="16S ribosomal RNA"

ORIGIN

1 gatccttcgg gattagtggc agacgggtga gtaacgcgtg ggaagctacc ttgtggtagg

61 gaacaacagt tggaaacgac tgctaatacc ctatgagccc tatgggggaa agatttatcg

121 ccatgagatg tgcccgcgtt agattagcta gttggtaagg taatggctta ccaaggcgac

181 gatctatagc tggtctgaga ggatgatcag ccacactggg actgagacac ggcccagact

241 cctacgggag gcagcagtgg ggaatattgg acaatggggg caaccctgat ccagccatgc

301 cgcgtgagtg atgacggcct tagggttgta aagctctttc agcagtgaag ataatgacat

361 taactgcaga agaagccccg gctaacttcg tgccagcagc cgcggtaata cgaagggggc

421 tagcgttgtt cggaatcact gggcgtaaag cgtacgtagg cggactgatc agtcaggggt

481 gaaatcccgg ggctcaaccc cggaactgcc tttgatactg tcagtcttga gatcgagaga

541 ggtgagtgga actccgagtg tagaggtgaa attcgtagat attcggaaga acaccagtgg

601 cgaaggcggc tcactggctc gatactgacg ctgaggtacg

//

LOCUS MK617652 640 bp DNA linear BCT 30-MAY-2019

DEFINITION Pseudovibrio sp. strain sC8 16S ribosomal RNA gene, partial

sequence.

ACCESSION MK617652

VERSION MK617652

KEYWORDS .

SOURCE Pseudovibrio sp.

ORGANISM Pseudovibrio sp.

Bacteria; Proteobacteria; Alphaproteobacteria; Rhodobacterales;

Rhodobacteraceae; Pseudovibrio.

REFERENCE 1 (bases 1 to 640)

AUTHORS Su,H., Huang,Q. and Yu,K.

TITLE Diversity of Cultivable Protease-Producing Bacteria

JOURNAL Unpublished

REFERENCE 2 (bases 1 to 640)

AUTHORS Su,H., Huang,Q. and Yu,K.

TITLE Direct Submission

JOURNAL Submitted (11-MAR-2019) Coral Reef Research Center of China, School

of Marine Sciences, No. 100 Daxue Road, Xixiangtang District,

Nanning, Guangxi Zhuang Autonomous Region 530004, China

COMMENT ##Assembly-Data-START##

Sequencing Technology :: Sanger dideoxy sequencing

##Assembly-Data-END##

FEATURES Location/Qualifiers

source 1..640

/organism="Pseudovibrio sp."

/mol_type="genomic DNA"

/strain="sC8"

/isolation_source="coral"

/db_xref="taxon:1909297"

rRNA <1..>640

/product="16S ribosomal RNA"

ORIGIN

1 tccttcggga ttagtggcag acgggtgagt aacgcgtggg aagctacctt gtggtaggga

61 acaacagttg gaaacgactg ctaataccct atgagcccta tgggggaaag atttatcgcc

121 atgagatgtg cccgcgttag attagctagt tggtaaggta atggcttacc aaggcgacga

181 tctatagctg gtctgagagg atgatcagcc acactgggac tgagacacgg cccagactcc

241 tacgggaggc agcagtgggg aatattggac aatgggggca accctgatcc agccatgccg

301 cgtgagtgat gacggcctta gggttgtaaa gctctttcag cagtgaagat aatgacatta

361 actgcagaag aagccccggc taacttcgtg ccagcagccg cggtaatacg aagggggcta

421 gcgttgttcg gaatcactgg gcgtaaagcg tacgtaggcg gactgatcag tcaggggtga

481 aatcccgggg ctcaaccccg gaactgcctt tgatactgtc agtcttgaaa tcgagagagg

541 tgagtggaac tccgagtgta gaggtgaaat tcgtagatat tcggaagaac accagtggcg

601 aaggcggctc actggctcga tactgacgct gaggtacgaa

//

LOCUS MK617653 640 bp DNA linear BCT 30-MAY-2019

DEFINITION Microbulbifer sp. strain sC9 16S ribosomal RNA gene, partial

sequence.

ACCESSION MK617653

VERSION MK617653

KEYWORDS .

SOURCE Microbulbifer sp.

ORGANISM Microbulbifer sp.

Bacteria; Proteobacteria; Gammaproteobacteria; Cellvibrionales;

Microbulbiferaceae; Microbulbifer.

REFERENCE 1 (bases 1 to 640)

AUTHORS Su,H., Huang,Q. and Yu,K.

TITLE Diversity of Cultivable Protease-Producing Bacteria

JOURNAL Unpublished

REFERENCE 2 (bases 1 to 640)

AUTHORS Su,H., Huang,Q. and Yu,K.

TITLE Direct Submission

JOURNAL Submitted (11-MAR-2019) Coral Reef Research Center of China, School

of Marine Sciences, No. 100 Daxue Road, Xixiangtang District,

Nanning, Guangxi Zhuang Autonomous Region 530004, China

COMMENT ##Assembly-Data-START##

Sequencing Technology :: Sanger dideoxy sequencing

##Assembly-Data-END##

FEATURES Location/Qualifiers

source 1..640

/organism="Microbulbifer sp."

/mol_type="genomic DNA"

/strain="sC9"

/isolation_source="coral"

/db_xref="taxon:1908541"

rRNA <1..>640

/product="16S ribosomal RNA"

ORIGIN

1 aagttcttcg gaacgagtag agcggcggac gggtgagtaa cgcgtgggaa attgcccagt

61 agtgggggac aacattcgga aacggatgct aataccgcat acgccctacg ggggaaagca

121 ggggatcttc ggaccttgcg ctattggata tgcccgcgtc ggattagcta gttggtgagg

181 taatggctca ccaaggcaac gatccgtagc tggtctgaga ggatgatcag ccacactggg

241 actgagacac ggcccagact cctacgggag gcagcagtgg ggaatattgg acaatgggcg

301 gaagcctgat ccagccatgc cgcgtgtgtg aagaaggccc tagggttgta aagcactttc

361 agtagggagg aaggccttaa agttaatacc tttgaggatt gacgttacct acagaagaag

421 caccggctaa ctccgtgcca gcagccgcgg taatacggag ggtgcaagcg ttaatcggaa

481 ttactgggcg taaagcgcgc gtaggcggtt agttaagctg gatgtgaaag ccccgggctc

541 aacctgggaa ctgcattcag aactggctgg ctagagtacg agagagggta gtggaatttc

601 ctgtgtagcg gtgaaatgcg tagatatagg aaggaacatc

//

LOCUS MK617654 640 bp DNA linear BCT 30-MAY-2019

DEFINITION Microbulbifer sp. strain sC10 16S ribosomal RNA gene, partial

sequence.

ACCESSION MK617654

VERSION MK617654

KEYWORDS .

SOURCE Microbulbifer sp.

ORGANISM Microbulbifer sp.

Bacteria; Proteobacteria; Gammaproteobacteria; Cellvibrionales;

Microbulbiferaceae; Microbulbifer.

REFERENCE 1 (bases 1 to 640)

AUTHORS Su,H., Huang,Q. and Yu,K.

TITLE Diversity of Cultivable Protease-Producing Bacteria

JOURNAL Unpublished

REFERENCE 2 (bases 1 to 640)

AUTHORS Su,H., Huang,Q. and Yu,K.

TITLE Direct Submission

JOURNAL Submitted (11-MAR-2019) Coral Reef Research Center of China, School

of Marine Sciences, No. 100 Daxue Road, Xixiangtang District,

Nanning, Guangxi Zhuang Autonomous Region 530004, China

COMMENT ##Assembly-Data-START##

Sequencing Technology :: Sanger dideoxy sequencing

##Assembly-Data-END##

FEATURES Location/Qualifiers

source 1..640

/organism="Microbulbifer sp."

/mol_type="genomic DNA"

/strain="sC10"

/isolation_source="coral"

/db_xref="taxon:1908541"

rRNA <1..>640

/product="16S ribosomal RNA"

ORIGIN

1 tcttcggaac gagtagagcg gcggacgggt gagtaacgcg tgggaaattg cccagtagtg

61 ggggacaaca ttcggaaacg gatgctaata ccgcatacgc cctacggggg aaagcagggg

121 atcttcggac cttgcgctat tggatatgcc cgcgtcggat tagctagttg gtgaggtaat

181 ggctcaccaa ggcaacgatc cgtagctggt ctgagaggat gatcagccac actgggactg

241 agacacggcc cagactccta cgggaggcag cagtggggaa tattggacaa tgggcggaag

301 cctgatccag ccatgccgcg tgtgtgaaga aggccctagg gttgtaaagc actttcagta

361 gggaggaagg ccttaaagtt aatacctttg aggattgacg ttacctacag aagaagcacc

421 ggctaactcc gtgccagcag ccgcggtaat acggagggtg caagcgttaa tcggaattac

481 tgggcgtaaa gcgcgcgtag gcggttagtt aagctggatg tgaaagcccc gggctcaacc

541 tgggaactgc attcagaact ggctggctag agtacgagag agggtagtgg aatttcctgt

601 gtagcggtga aatgcgtaga tataggaagg aacatcagtg

//

LOCUS MK617655 640 bp DNA linear BCT 30-MAY-2019

DEFINITION Pseudovibrio sp. strain sC11 16S ribosomal RNA gene, partial

sequence.

ACCESSION MK617655

VERSION MK617655

KEYWORDS .

SOURCE Pseudovibrio sp.

ORGANISM Pseudovibrio sp.

Bacteria; Proteobacteria; Alphaproteobacteria; Rhodobacterales;

Rhodobacteraceae; Pseudovibrio.

REFERENCE 1 (bases 1 to 640)

AUTHORS Su,H., Huang,Q. and Yu,K.

TITLE Diversity of Cultivable Protease-Producing Bacteria

JOURNAL Unpublished

REFERENCE 2 (bases 1 to 640)

AUTHORS Su,H., Huang,Q. and Yu,K.

TITLE Direct Submission

JOURNAL Submitted (11-MAR-2019) Coral Reef Research Center of China, School

of Marine Sciences, No. 100 Daxue Road, Xixiangtang District,

Nanning, Guangxi Zhuang Autonomous Region 530004, China

COMMENT ##Assembly-Data-START##

Sequencing Technology :: Sanger dideoxy sequencing

##Assembly-Data-END##

FEATURES Location/Qualifiers

source 1..640

/organism="Pseudovibrio sp."

/mol_type="genomic DNA"

/strain="sC11"

/isolation_source="coral"

/db_xref="taxon:1909297"

rRNA <1..>640

/product="16S ribosomal RNA"

ORIGIN

1 ccttcgggat tagtggcaga cgggtgagta acgcgtggga agctaccttg tggtagggaa

61 caacagttgg aaacgactgc taatacccta tgagccctat gggggaaaga tttatcgcca

121 tgagatgtgc ccgcgttaga ttagctagtt ggtaaggtaa tggcttacca aggcgacgat

181 ctatagctgg tctgagagga tgatcagcca cactgggact gagacacggc ccagactcct

241 acgggaggca gcagtgggga atattggaca atgggggcaa ccctgatcca gccatgccgc

301 gtgagtgatg acggccttag ggttgtaaag ctctttcagc agtgaagata atgacattaa

361 ctgcagaaga agccccggct aacttcgtgc cagcagccgc ggtaatacga agggggctag

421 cgttgttcgg aatcactggg cgtaaagcgt acgtaggcgg actgatcagt caggggtgaa

481 atcccggggc tcaaccccgg aactgccttt gatactgtca gtcttgagat cgagagaggt

541 gagtggaact ccgagtgtag aggtgaaatt cgtagatatt cggaagaaca ccagtggcga

601 aggcggctca ctggctcgat actgacgctg aggtacgaaa

//

LOCUS MK617656 640 bp DNA linear BCT 30-MAY-2019

DEFINITION Pseudovibrio sp. strain sC13 16S ribosomal RNA gene, partial

sequence.

ACCESSION MK617656

VERSION MK617656

KEYWORDS .

SOURCE Pseudovibrio sp.

ORGANISM Pseudovibrio sp.

Bacteria; Proteobacteria; Alphaproteobacteria; Rhodobacterales;

Rhodobacteraceae; Pseudovibrio.

REFERENCE 1 (bases 1 to 640)

AUTHORS Su,H., Huang,Q. and Yu,K.

TITLE Diversity of Cultivable Protease-Producing Bacteria

JOURNAL Unpublished

REFERENCE 2 (bases 1 to 640)

AUTHORS Su,H., Huang,Q. and Yu,K.

TITLE Direct Submission

JOURNAL Submitted (11-MAR-2019) Coral Reef Research Center of China, School

of Marine Sciences, No. 100 Daxue Road, Xixiangtang District,

Nanning, Guangxi Zhuang Autonomous Region 530004, China

COMMENT ##Assembly-Data-START##

Sequencing Technology :: Sanger dideoxy sequencing

##Assembly-Data-END##

FEATURES Location/Qualifiers

source 1..640

/organism="Pseudovibrio sp."

/mol_type="genomic DNA"

/strain="sC13"

/isolation_source="coral"

/db_xref="taxon:1909297"

rRNA <1..>640

/product="16S ribosomal RNA"

ORIGIN

1 gatccttcgg gattagtggc agacgggtga gtaacgcgtg ggaagctacc ttgtggtagg

61 gaacaacagt tggaaacgac tgctaatacc ctatgagccc tatgggggaa agatttatcg

121 ccatgagatg tgcccgcgtt agattagcta gttggtaagg taatggctta ccaaggcgac

181 gatctatagc tggtctgaga ggatgatcag ccacactggg actgagacac ggcccagact

241 cctacgggag gcagcagtgg ggaatattgg acaatggggg caaccctgat ccagccatgc

301 cgcgtgtgtg atgacggcct tagggttgta aagcactttc agcagtgaag ataatgacat

361 taactgcaga agaagccccg gctaacttcg tgccagcagc cgcggtaata cgaagggggc

421 tagcgttgtt cggaatcact gggcgtaaag cgtacgtagg cggactgatc agtcaggggt

481 gaaatcccgg ggctcaaccc cggaactgcc tttgatactg tcagtcttga gatcgagaga

541 ggtgagtgga actccgagtg tagaggtgaa attcgtagat attcggaaga acaccagtgg

601 cgaaggcggc tcactggctc gatactgacg ctgaggtacg

//

LOCUS MK617657 640 bp DNA linear BCT 30-MAY-2019

DEFINITION Pseudovibrio sp. strain sC14 16S ribosomal RNA gene, partial

sequence.

ACCESSION MK617657

VERSION MK617657

KEYWORDS .

SOURCE Pseudovibrio sp.

ORGANISM Pseudovibrio sp.

Bacteria; Proteobacteria; Alphaproteobacteria; Rhodobacterales;

Rhodobacteraceae; Pseudovibrio.

REFERENCE 1 (bases 1 to 640)

AUTHORS Su,H., Huang,Q. and Yu,K.

TITLE Diversity of Cultivable Protease-Producing Bacteria

JOURNAL Unpublished

REFERENCE 2 (bases 1 to 640)

AUTHORS Su,H., Huang,Q. and Yu,K.

TITLE Direct Submission

JOURNAL Submitted (11-MAR-2019) Coral Reef Research Center of China, School

of Marine Sciences, No. 100 Daxue Road, Xixiangtang District,

Nanning, Guangxi Zhuang Autonomous Region 530004, China

COMMENT ##Assembly-Data-START##

Sequencing Technology :: Sanger dideoxy sequencing

##Assembly-Data-END##

FEATURES Location/Qualifiers

source 1..640

/organism="Pseudovibrio sp."

/mol_type="genomic DNA"

/strain="sC14"

/isolation_source="coral"

/db_xref="taxon:1909297"

rRNA <1..>640

/product="16S ribosomal RNA"

ORIGIN

1 tccttcggga ttagtggcag acgggtgagt aacgcgtggg aagctacctt gtggtaggga

61 acaacagttg gaaacgactg ctaataccct atgagcccta tgggggaaag atttatcgcc

121 atgagatgtg cccgcgttag attagctagt tggtaaggta atggcttacc aaggcgacga

181 tctatagctg gtctgagagg atgatcagcc acactgggac tgagacacgg cccagactcc

241 tacgggaggc agcagtgggg aatattggac aatgggggca accctgatcc agccatgccg

301 cgtgagtgat gacggcctta gggttgtaaa gctctttcag cagtgaagat aatgacatta

361 actgcagaag aagccccggc taacttcgtg ccagcagccg cggtaatacg aagggggcta

421 gcgttgttcg gaatcactgg gcgtaaagcg tacgtaggcg gactgatcag tcaggggtga

481 aatcccgggg ctcaaccccg gaactgcctt tgatactgtc agtcttgaga tcgagagagg

541 tgagtggaac tccgagtgta gaggtgaaat tcgtagatat tcggaagaac accagtggcg

601 aaggcggctc actggctcga tactgacgct gaggtacgaa

//

LOCUS MK617658 640 bp DNA linear BCT 30-MAY-2019

DEFINITION Pseudovibrio sp. strain sC15 16S ribosomal RNA gene, partial

sequence.

ACCESSION MK617658

VERSION MK617658

KEYWORDS .

SOURCE Pseudovibrio sp.

ORGANISM Pseudovibrio sp.

Bacteria; Proteobacteria; Alphaproteobacteria; Rhodobacterales;

Rhodobacteraceae; Pseudovibrio.

REFERENCE 1 (bases 1 to 640)

AUTHORS Su,H., Huang,Q. and Yu,K.

TITLE Diversity of Cultivable Protease-Producing Bacteria

JOURNAL Unpublished

REFERENCE 2 (bases 1 to 640)

AUTHORS Su,H., Huang,Q. and Yu,K.

TITLE Direct Submission

JOURNAL Submitted (11-MAR-2019) Coral Reef Research Center of China, School

of Marine Sciences, No. 100 Daxue Road, Xixiangtang District,

Nanning, Guangxi Zhuang Autonomous Region 530004, China

COMMENT ##Assembly-Data-START##

Sequencing Technology :: Sanger dideoxy sequencing

##Assembly-Data-END##

FEATURES Location/Qualifiers

source 1..640

/organism="Pseudovibrio sp."

/mol_type="genomic DNA"

/strain="sC15"

/isolation_source="coral"

/db_xref="taxon:1909297"

rRNA <1..>640

/product="16S ribosomal RNA"

ORIGIN

1 ttcttcggaa ttagtggcag acgggtgagt aacgcgtggg aagctacctt gtggtaggga

61 acaacagttg gaaacgactg ctaataccct atgagcccta tgggggaaag atttatcgcc

121 atgagatgtg cccgcgttag attagctggt tggtaaggta atggcttacc aaggcgacga

181 tctatagctg gtctgagagg atgatcagcc acactgggac tgagacacgg cccagactcc

241 tacgggaggc agcagtgggg aatattggac aatgggggca accctgatcc agccatgccg

301 cgtgagtgat gacggcctta gggttgtaaa gctctttcag cagtgaagat aatgacatta

361 actgcagaag aagccccggc taacttcgtg ccagcagccg cggtaatacg aagggggcta

421 gcgttgttcg gaatcactgg gcgtaaagcg tacgtaggcg gactgatcag tcaggggtga

481 aatcccgggg ctcaaccccg gaactgcctt tgatactgtc agtcttgaga tcgagagagg

541 tgagtggaac tccgagtgta gaggtgaaat tcgtagatat tcggaagaac accagtggcg

601 aaggcggctc actggctcga tactgacgct gaggtacgaa

//

LOCUS MK617659 640 bp DNA linear BCT 30-MAY-2019

DEFINITION Oceanicola sp. strain sC16 16S ribosomal RNA gene, partial

sequence.

ACCESSION MK617659

VERSION MK617659

KEYWORDS .

SOURCE Oceanicola sp.

ORGANISM Oceanicola sp.

Bacteria; Proteobacteria; Alphaproteobacteria; Rhodobacterales;

Rhodobacteraceae; Oceanicola.

REFERENCE 1 (bases 1 to 640)

AUTHORS Su,H., Huang,Q. and Yu,K.

TITLE Diversity of Cultivable Protease-Producing Bacteria

JOURNAL Unpublished

REFERENCE 2 (bases 1 to 640)

AUTHORS Su,H., Huang,Q. and Yu,K.

TITLE Direct Submission

JOURNAL Submitted (11-MAR-2019) Coral Reef Research Center of China, School

of Marine Sciences, No. 100 Daxue Road, Xixiangtang District,

Nanning, Guangxi Zhuang Autonomous Region 530004, China

COMMENT ##Assembly-Data-START##

Sequencing Technology :: Sanger dideoxy sequencing

##Assembly-Data-END##

FEATURES Location/Qualifiers

source 1..640

/organism="Oceanicola sp."

/mol_type="genomic DNA"

/strain="sC16"

/isolation_source="coral"

/db_xref="taxon:1967289"

rRNA <1..>640

/product="16S ribosomal RNA"

ORIGIN

1 gcaccttcgg gtgagcggcg gacgggttag taacgcgtgg gaacgtgccc agatctgcgg

61 aatagccact ggaaacggtg agtaataccg catacgccct tcgggggaaa gatttatcgg

121 atttggatcg gcccgcgtta gattagatag ttggtggggt aatggcctac caagtctacg

181 atctatagct ggttttagag gatgatcagc aacactggga ctgagacacg gcccagactc

241 ctacgggagg cagcagtggg gaatcttgga caatgggcgc aagcctgatc cagccatgcc

301 gcgtgagtga tgaaggccct agggtcgtaa agctctttcg ccagggatga taatgacagt

361 acctggtaaa gaaaccccgg ctaactccgt gccagcagcc gcggtaatac ggagggggtt

421 agcgttgttc ggaattactg ggcgtaaagc gcgcgtaggc ggactattaa gtcaggggtg

481 aaatcccggg gctcaacccc ggaactgccc ttgatactgg tagtcttgag gtcgagagag

541 gtgagtggaa ttccgagtgt agaggtgaaa ttcgtagata ttcggaggaa caccagtggc

601 gaaggcggct cactggctcg atactgacgc tgaggtgcga

//

LOCUS MK617660 640 bp DNA linear BCT 30-MAY-2019

DEFINITION Vibrio sp. strain sC18 16S ribosomal RNA gene, partial sequence.

ACCESSION MK617660

VERSION MK617660

KEYWORDS .

SOURCE Vibrio sp.

ORGANISM Vibrio sp.

Bacteria; Proteobacteria; Gammaproteobacteria; Vibrionales;

Vibrionaceae; Vibrio.

REFERENCE 1 (bases 1 to 640)

AUTHORS Su,H., Huang,Q. and Yu,K.

TITLE Diversity of Cultivable Protease-Producing Bacteria

JOURNAL Unpublished

REFERENCE 2 (bases 1 to 640)

AUTHORS Su,H., Huang,Q. and Yu,K.

TITLE Direct Submission

JOURNAL Submitted (11-MAR-2019) Coral Reef Research Center of China, School

of Marine Sciences, No. 100 Daxue Road, Xixiangtang District,

Nanning, Guangxi Zhuang Autonomous Region 530004, China

COMMENT ##Assembly-Data-START##

Sequencing Technology :: Sanger dideoxy sequencing

##Assembly-Data-END##

FEATURES Location/Qualifiers

source 1..640

/organism="Vibrio sp."

/mol_type="genomic DNA"

/strain="sC18"

/isolation_source="coral"

/db_xref="taxon:678"

rRNA <1..>640

/product="16S ribosomal RNA"

ORIGIN

1 acgagaatag cttgctattc ggcgtcgagc ggcggacggg tgagtaatgc ctgggaaatt

61 gccctgatgt gggggataac cattggaaac gatggctaat accgcataat gccttcgggc

121 caaagagggg gaccttcggg cctctcgcgt caggatatgc ccaggtggga ttagctagtt

181 ggtgaggtaa aggctcacca aggcgacgat ccctagctgg tctgagagga tgatcagcca

241 cactggaact gagacacggt ccagactcct acgggaggca gcagtgggga atattgcaca

301 atgggcgcaa gcctgatgca gccatgccgc gtgtatgaag aaggccttcg ggttgtaaag

361 tactttcagc agtgaggaag ggtgtgaagt taatagcttc atatcttgac gttagctgca

421 gaagaagcac cggctaactc cgtgccagca gccgcggtaa tacggagggt gcgagcgtta

481 atcggaatta ctgggcgtaa agcgcatgca ggtggtttgt taagtcagat gtgaaagccc

541 ggggctcaac ctcggaaccg catttgaaac tggcaggcta gagtactgta gaggggggta

601 gaatttcagg tgtagcggtg aaatgcgtag agatctgaag

//

LOCUS MK617661 640 bp DNA linear BCT 30-MAY-2019

DEFINITION Pseudovibrio sp. strain sC21 16S ribosomal RNA gene, partial

sequence.

ACCESSION MK617661

VERSION MK617661

KEYWORDS .

SOURCE Pseudovibrio sp.

ORGANISM Pseudovibrio sp.

Bacteria; Proteobacteria; Alphaproteobacteria; Rhodobacterales;

Rhodobacteraceae; Pseudovibrio.

REFERENCE 1 (bases 1 to 640)

AUTHORS Su,H., Huang,Q. and Yu,K.

TITLE Diversity of Cultivable Protease-Producing Bacteria

JOURNAL Unpublished

REFERENCE 2 (bases 1 to 640)

AUTHORS Su,H., Huang,Q. and Yu,K.

TITLE Direct Submission

JOURNAL Submitted (11-MAR-2019) Coral Reef Research Center of China, School

of Marine Sciences, No. 100 Daxue Road, Xixiangtang District,

Nanning, Guangxi Zhuang Autonomous Region 530004, China

COMMENT ##Assembly-Data-START##

Sequencing Technology :: Sanger dideoxy sequencing

##Assembly-Data-END##

FEATURES Location/Qualifiers

source 1..640

/organism="Pseudovibrio sp."

/mol_type="genomic DNA"

/strain="sC21"

/isolation_source="coral"

/db_xref="taxon:1909297"

rRNA <1..>640

/product="16S ribosomal RNA"

ORIGIN

1 acggatcctt cgggattagt ggcagacggg tgagtaacgc gtgggaagct accttgtggt

61 agggaacaac agttggaaac gactgctaat accctatgag ccctatgggg gaaagattta

121 tcgccatgag atgtgcccgc gttagattag ctagttggta aggtaatggc ttaccaaggc

181 gacgatctat agctggtctg agaggatgat cagccacact gggactgaga cacggcccag

241 actcctacgg gaggcagcag tggggaatat tggacaatgg gggcaaccct gatccagcca

301 tgccgcgtga gtgatgacgg ccttagggtt gtaaagctct ttcagcagtg aagataatga

361 cattaactgc agaagaagcc ccggctaact tcgtgccagc agccgcggta atacgaaggg

421 ggctagcgtt gttcggaatc actgggcgta aagcgtacgt aggcggactg atcagtcagg

481 ggtgaaatcc cggggctcaa ccccggaact gcctttgata ctgtcagtct tgagatcgag

541 agaggtgagt ggaactccga gtgtagaggt gaaattcgta gatattcgga agaacaccag

601 tggcgaaggc ggctcactgg ctcgatactg acgctgaggt

//

LOCUS MK617662 640 bp DNA linear BCT 30-MAY-2019

DEFINITION Pseudovibrio sp. strain sC23 16S ribosomal RNA gene, partial

sequence.

ACCESSION MK617662

VERSION MK617662

KEYWORDS .

SOURCE Pseudovibrio sp.

ORGANISM Pseudovibrio sp.

Bacteria; Proteobacteria; Alphaproteobacteria; Rhodobacterales;

Rhodobacteraceae; Pseudovibrio.

REFERENCE 1 (bases 1 to 640)

AUTHORS Su,H., Huang,Q. and Yu,K.

TITLE Diversity of Cultivable Protease-Producing Bacteria

JOURNAL Unpublished

REFERENCE 2 (bases 1 to 640)

AUTHORS Su,H., Huang,Q. and Yu,K.

TITLE Direct Submission

JOURNAL Submitted (11-MAR-2019) Coral Reef Research Center of China, School

of Marine Sciences, No. 100 Daxue Road, Xixiangtang District,

Nanning, Guangxi Zhuang Autonomous Region 530004, China

COMMENT ##Assembly-Data-START##

Sequencing Technology :: Sanger dideoxy sequencing

##Assembly-Data-END##

FEATURES Location/Qualifiers

source 1..640

/organism="Pseudovibrio sp."

/mol_type="genomic DNA"

/strain="sC23"

/isolation_source="coral"

/db_xref="taxon:1909297"

rRNA <1..>640

/product="16S ribosomal RNA"

ORIGIN

1 ggatccttcg ggattagtgg cagacgggtg agtaacgcgt gggaagctac cttgtggtag

61 ggaacaacag ttggaaacga ctgctaatac cctatgagcc ctatggggga aagatttatc

121 gccatgagat gtgcccgcgt tagattagct agttggtaag gtaatggctt accaaggcga

181 cgatctatag ctggtctgag aggatgatca gccacactgg gactgagaca cggcccagac

241 tcctacggga ggcagcagtg gggaatattg gacaatgggg gcaaccctga tccagccatg

301 ccgcgtgagt gatgacggcc ttagggttgt aaagctcttt cagcagtgaa gataatgaca

361 ttaactgcag aagaagcccc ggctaacttc gtgccagcag ccgcggtaat acgaaggggg

421 ctagcgttgt tcggaatcac tgggcgtaaa gcgtacgtag gcggactgat cagtcagggg

481 tgaaatcccg gggctcaacc ccggaactgc ctttgatact gtcagtcttg agatcgagag

541 aggtgagtgg aactccgagt gtagaggtga aattcgtaga tattcggaag aacaccagtg

601 gcgaaggcgg ctcactggct cgatactgac gctgaggtac

//

LOCUS MK617663 640 bp DNA linear BCT 30-MAY-2019

DEFINITION Pseudovibrio sp. strain sC25 16S ribosomal RNA gene, partial

sequence.

ACCESSION MK617663

VERSION MK617663

KEYWORDS .

SOURCE Pseudovibrio sp.

ORGANISM Pseudovibrio sp.

Bacteria; Proteobacteria; Alphaproteobacteria; Rhodobacterales;

Rhodobacteraceae; Pseudovibrio.

REFERENCE 1 (bases 1 to 640)

AUTHORS Su,H., Huang,Q. and Yu,K.

TITLE Diversity of Cultivable Protease-Producing Bacteria

JOURNAL Unpublished

REFERENCE 2 (bases 1 to 640)

AUTHORS Su,H., Huang,Q. and Yu,K.

TITLE Direct Submission

JOURNAL Submitted (11-MAR-2019) Coral Reef Research Center of China, School

of Marine Sciences, No. 100 Daxue Road, Xixiangtang District,

Nanning, Guangxi Zhuang Autonomous Region 530004, China

COMMENT ##Assembly-Data-START##

Sequencing Technology :: Sanger dideoxy sequencing

##Assembly-Data-END##

FEATURES Location/Qualifiers

source 1..640

/organism="Pseudovibrio sp."

/mol_type="genomic DNA"

/strain="sC25"

/isolation_source="coral"

/db_xref="taxon:1909297"

rRNA <1..>640

/product="16S ribosomal RNA"

ORIGIN

1 gatccttcgg gattagtggc agacgggtga gtaacgcgtg ggaagctacc ttgtggtagg

61 gaacaacagt tggaaacgac tgctaatacc ctatgagccc tatgggggaa agatttatcg

121 ccatgagatg tgcccgcgtt agattagcta gttggtaagg taatggctta ccaaggcgac

181 gatctatagc tggtctgaga ggatgatcag ccacactggg actgagacac ggcccagact

241 cctacgggag gcagcagtgg ggaatattgg acaatggggg caaccctgat ccagccatgc

301 cgcgtgagtg atgacggcct tagggttgta aagctctttc agcagtgaag ataatgacat

361 taactgcaga agaagccccg gctaacttcg tgccagcagc cgcggtaata cgaagggggc

421 tagcgttgtt cggaatcact gggcgtaaag cgtacgtagg cggactgatc agtcaggggt

481 gaaatcccgg ggctcaaccc cggaactgcc tttgatactg tcagtcttga gatcgagaga

541 ggtgagtgga actccgagtg tagaggtgaa attcgtagat attcggaaga acaccagtgg

601 cgaaggcggc tcactggctc gatactgacg ctgaggtacg

//

LOCUS MK617664 640 bp DNA linear BCT 30-MAY-2019

DEFINITION Aquimarina sp. strain sC26 16S ribosomal RNA gene, partial

sequence.

ACCESSION MK617664

VERSION MK617664

KEYWORDS .

SOURCE Aquimarina sp.

ORGANISM Aquimarina sp.

Bacteria; Bacteroidetes; Flavobacteriia; Flavobacteriales;

Flavobacteriaceae; Aquimarina.

REFERENCE 1 (bases 1 to 640)

AUTHORS Su,H., Huang,Q. and Yu,K.

TITLE Diversity of Cultivable Protease-Producing Bacteria

JOURNAL Unpublished

REFERENCE 2 (bases 1 to 640)

AUTHORS Su,H., Huang,Q. and Yu,K.

TITLE Direct Submission

JOURNAL Submitted (11-MAR-2019) Coral Reef Research Center of China, School

of Marine Sciences, No. 100 Daxue Road, Xixiangtang District,

Nanning, Guangxi Zhuang Autonomous Region 530004, China

COMMENT ##Assembly-Data-START##

Sequencing Technology :: Sanger dideoxy sequencing

##Assembly-Data-END##

FEATURES Location/Qualifiers

source 1..640

/organism="Aquimarina sp."

/mol_type="genomic DNA"

/strain="sC26"

/isolation_source="coral"

/db_xref="taxon:1872586"

rRNA <1..>640

/product="16S ribosomal RNA"

ORIGIN

1 acatagttgc ttgcaactga tgacgaccgg cgcacgggtg cgtaacgcgt atagaaccta

61 ccttatagta agggatagcc cagagaaatt tggattaata ccttatagta tcgtttagat

121 gcatatttaa atgattaaag atttatcgct ataagatggc tatgcgttct attagctagt

181 tggtatggta acggcatacc aaggctacga tagatagggg tcctgagagg gagatccccc

241 acactggtac tgagacacgg accagactcc tacgggaggc agcagtgagg aatattggac

301 aatggaggca actctgatcc agccatgccg cgtgtaggaa gactgcccta tgggttgtaa

361 actactttta tagaggaaga aaccattcca cgtgtggaat gctgacggta ctctacgaat

421 aaggatcggc taactccgtg ccagcagccg cggtaatacg gaggatccaa gcgttatccg

481 gaatcattgg gtttaaaggg tccgtaggcg gtttagtaag tcagtggtga aagttttcgg

541 ctcaaccgga aaattgccat tgatactgca agacttgaat tattgtgaag tggttagaat

601 gtgtagtgta gcggtgaaat gcatagatat tacacagaat

//

LOCUS MK617665 640 bp DNA linear BCT 30-MAY-2019

DEFINITION Roseobacter sp. strain sC27 16S ribosomal RNA gene, partial

sequence.

ACCESSION MK617665

VERSION MK617665

KEYWORDS .

SOURCE Roseobacter sp.

ORGANISM Roseobacter sp.

Bacteria; Proteobacteria; Alphaproteobacteria; Rhodobacterales;

Rhodobacteraceae; Roseobacter.

REFERENCE 1 (bases 1 to 640)

AUTHORS Su,H., Huang,Q. and Yu,K.

TITLE Diversity of Cultivable Protease-Producing Bacteria

JOURNAL Unpublished

REFERENCE 2 (bases 1 to 640)

AUTHORS Su,H., Huang,Q. and Yu,K.

TITLE Direct Submission

JOURNAL Submitted (11-MAR-2019) Coral Reef Research Center of China, School

of Marine Sciences, No. 100 Daxue Road, Xixiangtang District,

Nanning, Guangxi Zhuang Autonomous Region 530004, China

COMMENT ##Assembly-Data-START##

Sequencing Technology :: Sanger dideoxy sequencing

##Assembly-Data-END##

FEATURES Location/Qualifiers

source 1..640

/organism="Roseobacter sp."

/mol_type="genomic DNA"

/strain="sC27"

/isolation_source="coral"

/db_xref="taxon:1907202"

rRNA <1..>640

/product="16S ribosomal RNA"

ORIGIN

1 gcagctacca tgcaagtcga gcgagatctt cggatctagc ggcggacggg tgagtaacgc

61 gtgggaacgt gcccttctct acggaatagt cccgggaaac tgggtttaat accgtatacg

121 cccttcgggg gaaagattta tcggagaagg atcggcccgc gttggattag gtagttggtg

181 gggtaatggc ctaccaagcc gacgatccat agctggtttg agaggatgat cagccacact

241 gggactgaga cacggcccag actcctacgg gaggcagcag tggggaatct tagacaatgg

301 gggcaaccct gatctagcca tgccgcgtga gtgatgaagg ccttagggtc gtaaagctct

361 ttcgccaggg atgataatga cagtacctgg tgaaaccccg gctaactccg tgccagcagc

421 cgcggtaata cggagggggt tagcgttgtt cggaattact gggcgtaaag cgcacgtagg

481 cggattagtc agtcagaggt gaaatcccag ggctcaaccc tggaactgcc tttgatactg

541 ctagtcttga gttcgagaga ggtgagtgga attccgagtg tagaggtgaa attcgtagat

601 attcggagga acaccagtgg cgaaggcggc tcactggctc

//

LOCUS MK617666 640 bp DNA linear BCT 30-MAY-2019

DEFINITION Pseudovibrio sp. strain sC28 16S ribosomal RNA gene, partial

sequence.

ACCESSION MK617666

VERSION MK617666

KEYWORDS .

SOURCE Pseudovibrio sp.

ORGANISM Pseudovibrio sp.

Bacteria; Proteobacteria; Alphaproteobacteria; Rhodobacterales;

Rhodobacteraceae; Pseudovibrio.

REFERENCE 1 (bases 1 to 640)

AUTHORS Su,H., Huang,Q. and Yu,K.

TITLE Diversity of Cultivable Protease-Producing Bacteria

JOURNAL Unpublished

REFERENCE 2 (bases 1 to 640)

AUTHORS Su,H., Huang,Q. and Yu,K.

TITLE Direct Submission

JOURNAL Submitted (11-MAR-2019) Coral Reef Research Center of China, School

of Marine Sciences, No. 100 Daxue Road, Xixiangtang District,

Nanning, Guangxi Zhuang Autonomous Region 530004, China

COMMENT ##Assembly-Data-START##

Sequencing Technology :: Sanger dideoxy sequencing

##Assembly-Data-END##

FEATURES Location/Qualifiers

source 1..640

/organism="Pseudovibrio sp."

/mol_type="genomic DNA"

/strain="sC28"

/isolation_source="coral"

/db_xref="taxon:1909297"

rRNA <1..>640

/product="16S ribosomal RNA"

ORIGIN

1 attcttcgga attagtggca gacgggtgag taacgcgtgg gaagctacct tgtggtaggg

61 aacaacagtt ggaaacgact gctaataccc tatgagccct atgggggaaa gatttatcgc

121 catgagatgt gcccgcgtta gattagctag ttggtaaggt aatggcttac caaggcgacg

181 atctatagct ggtctgagag gatgatcagc cacactggga ctgagacacg gcccagactc

241 ctacgggagg cagcagtggg gaatattgga caatgggggc aaccctgatc cagccatgcc

301 gcgtgagtga tgacggcctt agggttgtaa agctctttca gcagtgaaga taatgacatt

361 aactgcagaa gaagccccgg ctaacttcgt gccagcagcc gcggtaatac gaagggggct

421 agcgttgttc ggaatcactg ggcgtaaagc gtacgtaggc ggactgatca gtcaggggtg

481 aaatcccggg gctcaacccc ggaactgcct ttgatactgt cagtcttgag atcgagagag

541 gtgagtggaa ctccgagtgt agaggtgaaa ttcgtagata ttcggaagaa caccagtggc

601 gaaggcggct cactggctcg atactgacgc tgaggtacga

//

LOCUS MK617667 640 bp DNA linear BCT 30-MAY-2019

DEFINITION Pseudovibrio sp. strain sC29 16S ribosomal RNA gene, partial

sequence.

ACCESSION MK617667

VERSION MK617667

KEYWORDS .

SOURCE Pseudovibrio sp.

ORGANISM Pseudovibrio sp.

Bacteria; Proteobacteria; Alphaproteobacteria; Rhodobacterales;

Rhodobacteraceae; Pseudovibrio.

REFERENCE 1 (bases 1 to 640)

AUTHORS Su,H., Huang,Q. and Yu,K.

TITLE Diversity of Cultivable Protease-Producing Bacteria

JOURNAL Unpublished

REFERENCE 2 (bases 1 to 640)

AUTHORS Su,H., Huang,Q. and Yu,K.

TITLE Direct Submission

JOURNAL Submitted (11-MAR-2019) Coral Reef Research Center of China, School

of Marine Sciences, No. 100 Daxue Road, Xixiangtang District,

Nanning, Guangxi Zhuang Autonomous Region 530004, China

COMMENT ##Assembly-Data-START##

Sequencing Technology :: Sanger dideoxy sequencing

##Assembly-Data-END##

FEATURES Location/Qualifiers

source 1..640

/organism="Pseudovibrio sp."

/mol_type="genomic DNA"

/strain="sC29"

/isolation_source="coral"

/db_xref="taxon:1909297"

rRNA <1..>640

/product="16S ribosomal RNA"

ORIGIN

1 ggattcttcg gaattagtgg cagacgggtg agtaacgcgt gggaagctac cttgtggtag

61 ggaacaacag ttggaaacga ctgctaatac cctatgagcc ctatggggga aagatttatc

121 gccatgagat gtgcccgcgt tagattagct agttggtaag gtaatggctt accaaggcga

181 cgatctatag ctggtctgag aggatgatca gccacactgg gactgagaca cggcccagac

241 tcctacggga ggcagcagtg gggaatattg gacaatgggg gcaaccctga tccagccatg

301 ccgcgtgagt gatgacggcc ttagggttgt aaagctcttt cagcagtgaa gataatgaca

361 ttaactgcag aagaagcccc ggctaacttc gtgccagcag ccgcggtaat acgaaggggg

421 ctagcgttgt tcggaatcac tgggcgtaaa gcgtacgtag gcggactgat cagtcagggg

481 tgaaatcccg gggctcaacc ccggaactgc ctttgatact gtcagtcttg agatcgagag

541 aggtgagtgg aactccgagt gtagaggtga aattcgtaga tattcggaag aacaccagtg

601 gcgaaggcgg ctcactggct cgatactgac gctgaggtac

//

LOCUS MK617668 640 bp DNA linear BCT 30-MAY-2019

DEFINITION Pseudovibrio sp. strain sC30 16S ribosomal RNA gene, partial

sequence.

ACCESSION MK617668

VERSION MK617668

KEYWORDS .

SOURCE Pseudovibrio sp.

ORGANISM Pseudovibrio sp.

Bacteria; Proteobacteria; Alphaproteobacteria; Rhodobacterales;

Rhodobacteraceae; Pseudovibrio.

REFERENCE 1 (bases 1 to 640)

AUTHORS Su,H., Huang,Q. and Yu,K.

TITLE Diversity of Cultivable Protease-Producing Bacteria

JOURNAL Unpublished

REFERENCE 2 (bases 1 to 640)

AUTHORS Su,H., Huang,Q. and Yu,K.

TITLE Direct Submission

JOURNAL Submitted (11-MAR-2019) Coral Reef Research Center of China, School

of Marine Sciences, No. 100 Daxue Road, Xixiangtang District,

Nanning, Guangxi Zhuang Autonomous Region 530004, China

COMMENT ##Assembly-Data-START##

Sequencing Technology :: Sanger dideoxy sequencing

##Assembly-Data-END##

FEATURES Location/Qualifiers

source 1..640

/organism="Pseudovibrio sp."

/mol_type="genomic DNA"

/strain="sC30"

/isolation_source="coral"

/db_xref="taxon:1909297"

rRNA <1..>640

/product="16S ribosomal RNA"

ORIGIN

1 atccttcggg attagtggca gacgggtgag taacgcgtgg gaagctacct tgtggtaggg

61 aacaacagtt ggaaacgact gctaataccc tatgagccct atgggggaaa gatttatcgc

121 catgagatgt gcccgcgtta gattagctag ttggtaaggt aatggcttac caaggcgacg

181 atctatagct ggtctgagag gatgatcagc cacactggga ctgagacacg gcccagactc

241 ctacgggagg cagcagtggg gaatattgga caatgggggc aaccctgatc cagccatgcc

301 gcgtgagtga tgacggcctt agggttgtaa agctctttca gcagtgaaga taatgacatt

361 aactgcagaa gaagccccgg ctaacttcgt gccagcagcc gcggtaatac gaagggggct

421 agcgttgttc ggaatcactg ggcgtaaagc gtacgtaggc ggactgatca gtcaggggtg

481 aaatcccggg gctcaacccc ggaactgcct ttgatactgt cagtcttgag atcgagagag

541 gtgagtggaa ctccgagtgt agaggtgaaa ttcgtagata ttcggaagaa caccagtggc

601 gaaggcggct cactggctcg atactgacgc tgaggtacga

//

LOCUS MK617669 640 bp DNA linear BCT 30-MAY-2019

DEFINITION Vibrio sp. strain sC33 16S ribosomal RNA gene, partial sequence.

ACCESSION MK617669

VERSION MK617669

KEYWORDS .

SOURCE Vibrio sp.

ORGANISM Vibrio sp.

Bacteria; Proteobacteria; Gammaproteobacteria; Vibrionales;

Vibrionaceae; Vibrio.

REFERENCE 1 (bases 1 to 640)

AUTHORS Su,H., Huang,Q. and Yu,K.

TITLE Diversity of Cultivable Protease-Producing Bacteria

JOURNAL Unpublished

REFERENCE 2 (bases 1 to 640)

AUTHORS Su,H., Huang,Q. and Yu,K.

TITLE Direct Submission

JOURNAL Submitted (11-MAR-2019) Coral Reef Research Center of China, School

of Marine Sciences, No. 100 Daxue Road, Xixiangtang District,

Nanning, Guangxi Zhuang Autonomous Region 530004, China

COMMENT ##Assembly-Data-START##

Sequencing Technology :: Sanger dideoxy sequencing

##Assembly-Data-END##

FEATURES Location/Qualifiers

source 1..640

/organism="Vibrio sp."

/mol_type="genomic DNA"

/strain="sC33"

/isolation_source="coral"

/db_xref="taxon:678"

rRNA <1..>640

/product="16S ribosomal RNA"

ORIGIN

1 acgagttatc tgaaccttcg gggaacgata acggcgtcga gcggcggacg ggtgagtaat

61 gcctaggaaa ttgccctgat gtgggggata accattggaa acgatggcta ataccgcatg

121 atgcctacgg gccaaagagg gggaccttcg ggcctctcgc gtcaggatat gcctaggtgg

181 gattagctag ttggtgaggt aagggctcac caaggcgacg atccctagct ggtctgagag

241 gatgatcagc cacactggaa ctgagacacg gtccagactc ctacgggagg cagcagtggg

301 gaatattgca caatgggcgc aagcctgatg cagccatgcc gcgtgtgtga agaaggcctt

361 cgggttgtaa agcactttca gtcgtgagga aggtggtgta gttaatagct gcattatttg

421 acgttagcga cagaagaagc accggctaac tccgtgccag cagccgcggt aatacggagg

481 gtgcgagcgt taatcggaat tactgggcgt aaagcgcatg caggtggttt gttaagtcag

541 atgtgaaagc ccggggctca acctcggaat agcatttgaa actggcagac tagagtactg

601 tagagggggg tagaatttca ggtgtagcgg tgaaatgcgt

//

LOCUS MK617670 640 bp DNA linear BCT 30-MAY-2019

DEFINITION Vibrio sp. strain sC34 16S ribosomal RNA gene, partial sequence.

ACCESSION MK617670

VERSION MK617670

KEYWORDS .

SOURCE Vibrio sp.

ORGANISM Vibrio sp.

Bacteria; Proteobacteria; Gammaproteobacteria; Vibrionales;

Vibrionaceae; Vibrio.

REFERENCE 1 (bases 1 to 640)

AUTHORS Su,H., Huang,Q. and Yu,K.

TITLE Diversity of Cultivable Protease-Producing Bacteria

JOURNAL Unpublished

REFERENCE 2 (bases 1 to 640)

AUTHORS Su,H., Huang,Q. and Yu,K.

TITLE Direct Submission

JOURNAL Submitted (11-MAR-2019) Coral Reef Research Center of China, School

of Marine Sciences, No. 100 Daxue Road, Xixiangtang District,

Nanning, Guangxi Zhuang Autonomous Region 530004, China

COMMENT ##Assembly-Data-START##

Sequencing Technology :: Sanger dideoxy sequencing

##Assembly-Data-END##

FEATURES Location/Qualifiers

source 1..640

/organism="Vibrio sp."

/mol_type="genomic DNA"

/strain="sC34"

/isolation_source="coral"

/db_xref="taxon:678"

rRNA <1..>640

/product="16S ribosomal RNA"

ORIGIN

1 cgagttatct gaaccttcgg ggaacgataa cggcgtcgag cggcggacgg gtgagtaatg

61 cctaggaaat tgccctgatg tgggggataa ccattggaaa cgatggctaa taccgcatga

121 tgcctacggg ccaaagaggg ggaccttcgg gcctctcgcg tcaggatatg cctaggtggg

181 attagctagt tggtgaggta agggctcacc aaggcgacga tccctagctg gtctgagagg

241 atgatcagcc acactggaac tgagacacgg tccagactcc tacgggaggc agcagtgggg

301 aatattgcac aatgggcgca agcctgatgc agccatgccg cgtgtgtgaa gaaggccttc

361 gggttgtaaa gcactttcag tcgtgaggaa ggtggtgtag ttaatagctg cattatttga

421 cgttagcgac agaagaagca ccggctaact ccgtgccagc agccgcggta atacggaggg

481 tgcgagcgtt aatcggaatt actgggcgta aagcgcatgc aggtggtttg ttaagtcaga

541 tgtgaaagcc cggggctcaa cctcggaata gcatttgaaa ctggcagact agagtactgt

601 agaggggggt agaatttcag gtgtagcggt gaaatgcgta

//

LOCUS MK617671 640 bp DNA linear BCT 30-MAY-2019

DEFINITION Vibrio sp. strain sC42 16S ribosomal RNA gene, partial sequence.

ACCESSION MK617671

VERSION MK617671

KEYWORDS .

SOURCE Vibrio sp.

ORGANISM Vibrio sp.

Bacteria; Proteobacteria; Gammaproteobacteria; Vibrionales;

Vibrionaceae; Vibrio.

REFERENCE 1 (bases 1 to 640)

AUTHORS Su,H., Huang,Q. and Yu,K.

TITLE Diversity of Cultivable Protease-Producing Bacteria

JOURNAL Unpublished

REFERENCE 2 (bases 1 to 640)

AUTHORS Su,H., Huang,Q. and Yu,K.

TITLE Direct Submission

JOURNAL Submitted (11-MAR-2019) Coral Reef Research Center of China, School

of Marine Sciences, No. 100 Daxue Road, Xixiangtang District,

Nanning, Guangxi Zhuang Autonomous Region 530004, China

COMMENT ##Assembly-Data-START##

Sequencing Technology :: Sanger dideoxy sequencing

##Assembly-Data-END##

FEATURES Location/Qualifiers

source 1..640

/organism="Vibrio sp."

/mol_type="genomic DNA"

/strain="sC42"

/isolation_source="coral"

/db_xref="taxon:678"

rRNA <1..>640

/product="16S ribosomal RNA"

ORIGIN

1 cggaacgagt tatctgaacc ttcggggaac gataacggcg tcgagcggcg gacgggtgag

61 taatgcctag gaaattgccc tgatgtgggg gataaccatt ggaaacgatg gctaataccg

121 catgatgcct acgggccaaa gagggggacc ttcgggcctc tcgcgtcagg atatgcctag

181 gtgggattag ctagttggtg aggtaagggc tcaccaaggc gacgatccct agctggtctg

241 agaggatgat cagccacact ggaactgaga cacggtccag actcctacgg gaggcagcag

301 tggggaatat tgcacaatgg gcgcaagcct gatgcagcca tgccgcgtgt gtgaagaagg

361 ccttcgggtt gtaaagcact ttcagtcgtg aggaaggtgg tgtagttaat agctgcatta

421 tttgacgtta gcgacagaag aagcaccggc taactccgtg ccagcagccg cggtaatacg

481 gagggtgcga gcgttaatcg gaattactgg gcgtaaagcg catgcaggtg gtttgttaag

541 tcagatgtga aagcccgggg ctcaacctcg gaatagcatt tgaaactggc agactagagt

601 actgtagagg ggggtagaat ttcaggtgta gcggtgaaat

//

LOCUS MK617672 640 bp DNA linear BCT 30-MAY-2019

DEFINITION Vibrio sp. strain sC47 16S ribosomal RNA gene, partial sequence.

ACCESSION MK617672

VERSION MK617672

KEYWORDS .

SOURCE Vibrio sp.

ORGANISM Vibrio sp.

Bacteria; Proteobacteria; Gammaproteobacteria; Vibrionales;

Vibrionaceae; Vibrio.

REFERENCE 1 (bases 1 to 640)

AUTHORS Su,H., Huang,Q. and Yu,K.

TITLE Diversity of Cultivable Protease-Producing Bacteria

JOURNAL Unpublished

REFERENCE 2 (bases 1 to 640)

AUTHORS Su,H., Huang,Q. and Yu,K.

TITLE Direct Submission

JOURNAL Submitted (11-MAR-2019) Coral Reef Research Center of China, School

of Marine Sciences, No. 100 Daxue Road, Xixiangtang District,

Nanning, Guangxi Zhuang Autonomous Region 530004, China

COMMENT ##Assembly-Data-START##

Sequencing Technology :: Sanger dideoxy sequencing

##Assembly-Data-END##

FEATURES Location/Qualifiers

source 1..640

/organism="Vibrio sp."

/mol_type="genomic DNA"

/strain="sC47"

/isolation_source="coral"

/db_xref="taxon:678"

rRNA <1..>640

/product="16S ribosomal RNA"

ORIGIN

1 gaaacgagtt atctgaacct tcggggaacg ataacggcgt cgagcggcgg acgggtgagt

61 aatgcctagg aaattgccct gatgtggggg ataaccattg gaaacgatgg ctaataccgc

121 atgatgccta cgggccaaag agggggacct tcgggcctct cgcgtcagga tatgcctagg

181 tgggattagc tagttggtga ggtaagggct caccaaggcg acgatcccta gctggtctga

241 gaggatgatc agccacactg gaactgagac acggtccaga ctcctacggg aggcagcagt

301 ggggaatatt gcacaatggg cgcaagcctg atgcagccat gccgcgtgtg tgaagaaggc

361 cttcgggttg taaagcactt tcagtcgtga ggaaggtggt gtagttaata gctgcattat

421 ttgacgttag cgacagaaga agcaccggct aactccgtgc cagcagccgc ggtaatacgg

481 agggtgcgag cgttaatcgg aattactggg cgtaaagcgc atgcaggtgg tttgttaagt

541 cagatgtgaa agcccggggc tcaacctcgg aatagcattt gaaactggca gactagagta

601 ctgtagaggg gggtagaatt tcaggtgtag cggtgaaatg

//

LOCUS MK617673 640 bp DNA linear BCT 30-MAY-2019

DEFINITION Vibrio sp. strain sC48 16S ribosomal RNA gene, partial sequence.

ACCESSION MK617673

VERSION MK617673

KEYWORDS .

SOURCE Vibrio sp.

ORGANISM Vibrio sp.

Bacteria; Proteobacteria; Gammaproteobacteria; Vibrionales;

Vibrionaceae; Vibrio.

REFERENCE 1 (bases 1 to 640)

AUTHORS Su,H., Huang,Q. and Yu,K.

TITLE Diversity of Cultivable Protease-Producing Bacteria

JOURNAL Unpublished

REFERENCE 2 (bases 1 to 640)

AUTHORS Su,H., Huang,Q. and Yu,K.

TITLE Direct Submission

JOURNAL Submitted (11-MAR-2019) Coral Reef Research Center of China, School

of Marine Sciences, No. 100 Daxue Road, Xixiangtang District,

Nanning, Guangxi Zhuang Autonomous Region 530004, China

COMMENT ##Assembly-Data-START##

Sequencing Technology :: Sanger dideoxy sequencing

##Assembly-Data-END##

FEATURES Location/Qualifiers

source 1..640

/organism="Vibrio sp."

/mol_type="genomic DNA"

/strain="sC48"

/isolation_source="coral"

/db_xref="taxon:678"

rRNA <1..>640

/product="16S ribosomal RNA"

ORIGIN

1 gcggaaacga gttatctgaa ccttcgggga acgataacgg cgtcgagcgg cggacgggtg

61 agtaatgcct aggaaattgc cctgatgtgg gggataacca ttggaaacga tggctaatac

121 cgcatgatgc ctacgggcca aagaggggga ccttcgggcc tctcgcgtca ggatatgcct

181 aggtgggatt agctagttgg tgaggtaagg gctcaccaag gcgacgatcc ctagctggtc

241 tgagaggatg atcagccaca ctggaactga gacacggtcc agactcctac gggaggcagc

301 agtggggaat attgcacaat gggcgcaagc ctgatgcagc catgccgcgt gtgtgaagaa

361 ggccttcggg ttgtaaagca ctttcagtcg tgaggaaggt ggtgtagtta atagctgcat

421 tatttgacgt tagcgacaga agaagcaccg gctaactccg tgccagcagc cgcggtaata

481 cggagggtgc gagcgttaat cggaattact gggcgtaaag cgcatgcagg tggtttgtta

541 agtcagatgt gaaagcccgg ggctcaacct cggaatagca tttgaaactg gcagactaga

601 gtactgtaga ggggggtaga atttcaggtg tagcggtgaa

//

LOCUS MK617674 640 bp DNA linear BCT 30-MAY-2019

DEFINITION Vibrio sp. strain sC51 16S ribosomal RNA gene, partial sequence.

ACCESSION MK617674

VERSION MK617674

KEYWORDS .

SOURCE Vibrio sp.

ORGANISM Vibrio sp.

Bacteria; Proteobacteria; Gammaproteobacteria; Vibrionales;

Vibrionaceae; Vibrio.

REFERENCE 1 (bases 1 to 640)

AUTHORS Su,H., Huang,Q. and Yu,K.

TITLE Diversity of Cultivable Protease-Producing Bacteria

JOURNAL Unpublished

REFERENCE 2 (bases 1 to 640)

AUTHORS Su,H., Huang,Q. and Yu,K.

TITLE Direct Submission

JOURNAL Submitted (11-MAR-2019) Coral Reef Research Center of China, School

of Marine Sciences, No. 100 Daxue Road, Xixiangtang District,

Nanning, Guangxi Zhuang Autonomous Region 530004, China

COMMENT ##Assembly-Data-START##

Sequencing Technology :: Sanger dideoxy sequencing

##Assembly-Data-END##

FEATURES Location/Qualifiers

source 1..640

/organism="Vibrio sp."

/mol_type="genomic DNA"

/strain="sC51"

/isolation_source="coral"

/db_xref="taxon:678"

rRNA <1..>640

/product="16S ribosomal RNA"

ORIGIN

1 tcggggaacg ataacggcgt cgagcggcgg acgggtgagt aatgcctagg aaattgccct

61 gatgtggggg ataaccattg gaaacgatgg ctaataccgc atgatgccta cgggccaaag

121 agggggacct tcgggcctct cgcgtcagga tatgcctagg tgggattagc tagttggtga

181 ggtaagggct caccaaggcg acgatcccta gctggtctga gaggatgatc agccacactg

241 gaactgagac acggtccaga ctcctacggg aggcagcagt ggggaatatt gcacaatggg

301 cgcaagcctg atgcagccat gccgcgtgtg tgaagaaggc cttcgggttg taaagcactt

361 tcagtcgtga ggaaggtggt gtagttaata gctgcattat ttgacgttag cgacagaaga

421 agcaccggct aactccgtgc cagcagccgc ggtaatacgg agggtgcgag cgttaatcgg

481 aattactggg cgtaaagcgc atgcaggtgg tttgttaagt cagatgtgaa agcccggggc

541 tcaacctcgg aatagcattt gaaactggca gactagagta ctgtagaggg gggtagaatt

601 tcaggtgtag cggtgaaatg cgtagagatc tgaaggaata

//

LOCUS MK617675 640 bp DNA linear BCT 30-MAY-2019

DEFINITION Vibrio sp. strain sC57 16S ribosomal RNA gene, partial sequence.

ACCESSION MK617675

VERSION MK617675

KEYWORDS .

SOURCE Vibrio sp.

ORGANISM Vibrio sp.

Bacteria; Proteobacteria; Gammaproteobacteria; Vibrionales;

Vibrionaceae; Vibrio.

REFERENCE 1 (bases 1 to 640)

AUTHORS Su,H., Huang,Q. and Yu,K.

TITLE Diversity of Cultivable Protease-Producing Bacteria

JOURNAL Unpublished

REFERENCE 2 (bases 1 to 640)

AUTHORS Su,H., Huang,Q. and Yu,K.

TITLE Direct Submission

JOURNAL Submitted (11-MAR-2019) Coral Reef Research Center of China, School

of Marine Sciences, No. 100 Daxue Road, Xixiangtang District,

Nanning, Guangxi Zhuang Autonomous Region 530004, China

COMMENT ##Assembly-Data-START##

Sequencing Technology :: Sanger dideoxy sequencing

##Assembly-Data-END##

FEATURES Location/Qualifiers

source 1..640

/organism="Vibrio sp."

/mol_type="genomic DNA"

/strain="sC57"

/isolation_source="coral"

/db_xref="taxon:678"

rRNA <1..>640

/product="16S ribosomal RNA"

ORIGIN

1 gtcgagcgga acgagttatc tgaaccttcg gggaacgata acggcgtcga gcggcggacg

61 ggtgagtaat gcctaggaaa ttgccctgat gtgggggata accattggaa acgatggcta

121 ataccgcatg atgcctacgg gccaaagagg gggaccttcg ggcctctcgc gtcaggatat

181 gcctaggtgg gattagctag ttggtgaggt aagggctcac caaggcgacg atccctagct

241 ggtctgagag gatgatcagc cacactggaa ctgagacacg gtccagactc ctacgggagg

301 cagcagtggg gaatattgca caatgggcgc aagcctgatg cagccatgcc gcgtgtgtga

361 agaaggcctt cgggttgtaa agcactttca gtcgtgagga aggtggtgta gttaatagct

421 gcatcatttg acgttagcga cagaagaagc accggctaac tccgtgccag cagccgcggt

481 aatacggagg gtgcgagcgt taatcggaat tactgggcgt aaagcgcatg caggtggttt

541 gttaagtcag atgtgaaagc ccggggctca acctcggaat agcatttgaa actggcagac

601 tagagtactg tagagggggg tagaatttca ggtgtagcgg

//

LOCUS MK617676 640 bp DNA linear BCT 30-MAY-2019

DEFINITION Vibrio sp. strain sC62 16S ribosomal RNA gene, partial sequence.

ACCESSION MK617676

VERSION MK617676

KEYWORDS .

SOURCE Vibrio sp.

ORGANISM Vibrio sp.

Bacteria; Proteobacteria; Gammaproteobacteria; Vibrionales;

Vibrionaceae; Vibrio.

REFERENCE 1 (bases 1 to 640)

AUTHORS Su,H., Huang,Q. and Yu,K.

TITLE Diversity of Cultivable Protease-Producing Bacteria

JOURNAL Unpublished

REFERENCE 2 (bases 1 to 640)

AUTHORS Su,H., Huang,Q. and Yu,K.

TITLE Direct Submission

JOURNAL Submitted (11-MAR-2019) Coral Reef Research Center of China, School

of Marine Sciences, No. 100 Daxue Road, Xixiangtang District,

Nanning, Guangxi Zhuang Autonomous Region 530004, China

COMMENT ##Assembly-Data-START##

Sequencing Technology :: Sanger dideoxy sequencing

##Assembly-Data-END##

FEATURES Location/Qualifiers

source 1..640

/organism="Vibrio sp."

/mol_type="genomic DNA"

/strain="sC62"

/isolation_source="coral"

/db_xref="taxon:678"

rRNA <1..>640

/product="16S ribosomal RNA"

ORIGIN

1 aacgagttat ctgaaccttc ggggaacgat aacggcgtcg agcggcggac gggtgagtaa

61 tgcctaggaa attgccctga tgtgggggat aaccattgga aacgatggct aataccgcat

121 gatgcctacg ggccaaagag ggggaccttc gggcctctcg cgtcaggata tgcctaggtg

181 ggattagcta gttggtgagg taagggctca ccaaggcgac gatccctagc tggtctgaga

241 ggatgatcag ccacactgga actgagacac ggtccagact cctacgggag gcagcagtgg

301 ggaatattgc acaatgggcg caagcctgat gcagccatgc cgcgtgtgtg aagaaggcct

361 tcgggttgta aagcactttc agtcgtgagg aaggtggtgt agttaatagc tgcattattt

421 gacgttagcg acagaagaag caccggctaa ctccgtgcca gcagccgcgg taatacggag

481 ggtgcgagcg ttaatcggaa ttactgggcg taaagcgcat gcaggtggtt tgttaagtca

541 gatgtgaaag cccggggctc aacctcggaa tagcatttga aactggcaga ctagagtact

601 gtagaggggg gtagaatttc aggtgtagcg gtgaaatgcg

//

LOCUS MK617677 640 bp DNA linear BCT 30-MAY-2019

DEFINITION Fictibacillus sp. strain sD11 16S ribosomal RNA gene, partial

sequence.

ACCESSION MK617677

VERSION MK617677

KEYWORDS .

SOURCE Fictibacillus sp.

ORGANISM Fictibacillus sp.

Bacteria; Firmicutes; Bacilli; Bacillales; Bacillaceae;

Fictibacillus.

REFERENCE 1 (bases 1 to 640)

AUTHORS Su,H., Huang,Q. and Yu,K.

TITLE Diversity of Cultivable Protease-Producing Bacteria

JOURNAL Unpublished

REFERENCE 2 (bases 1 to 640)

AUTHORS Su,H., Huang,Q. and Yu,K.

TITLE Direct Submission

JOURNAL Submitted (11-MAR-2019) Coral Reef Research Center of China, School

of Marine Sciences, No. 100 Daxue Road, Xixiangtang District,

Nanning, Guangxi Zhuang Autonomous Region 530004, China

COMMENT ##Assembly-Data-START##

Sequencing Technology :: Sanger dideoxy sequencing

##Assembly-Data-END##

FEATURES Location/Qualifiers

source 1..640

/organism="Fictibacillus sp."

/mol_type="genomic DNA"

/strain="sD11"

/isolation_source="coral"

/db_xref="taxon:1871617"

rRNA <1..>640

/product="16S ribosomal RNA"

ORIGIN

1 aatgatgagg agcttgctcc tctgatttag cggcggacgg gtgagtaaca cgtgggtaat

61 ctgcctgtaa gacggggata actccgggaa accggggcta ataccggata ataagagaag

121 aagcatttct tctttttgaa agtcggtttc ggctgacact tacagatgag cccgcggcgc

181 attagctagt tggtgaggta acggctcacc aaggcgacga tgcgtagccg acctgagagg

241 gtgatcggcc acactgggac tgagacacgg cccagactcc tacgggaggc agcagtaggg

301 aatcttcggc aatgggcgaa agcctgaccg agcaacgccg cgtgagcgat gaaggccttc

361 gggtcgtaaa gctctgttgt tagagaagaa caagtacgag agtaactgct cgtaccttga

421 cggtacctaa ccagaaagcc acggctaact acgtgccagc agccgcggta atacgtaggt

481 ggcaagcgtt atccggaatt attgggcgta aagcgcgcgc aggcggtctc ttaagtctga

541 tgtgaaagcc cacggctcaa ccgtggaggg tcattggaaa ctgggagact tgagtgcagg

601 agagaaaagt ggaattccac gtgtagcggt gaaatgcgta

//

LOCUS MK617678 640 bp DNA linear BCT 30-MAY-2019

DEFINITION Microbulbifer sp. strain sD13 16S ribosomal RNA gene, partial

sequence.

ACCESSION MK617678

VERSION MK617678

KEYWORDS .

SOURCE Microbulbifer sp.

ORGANISM Microbulbifer sp.

Bacteria; Proteobacteria; Gammaproteobacteria; Cellvibrionales;

Microbulbiferaceae; Microbulbifer.

REFERENCE 1 (bases 1 to 640)

AUTHORS Su,H., Huang,Q. and Yu,K.

TITLE Diversity of Cultivable Protease-Producing Bacteria

JOURNAL Unpublished

REFERENCE 2 (bases 1 to 640)

AUTHORS Su,H., Huang,Q. and Yu,K.

TITLE Direct Submission

JOURNAL Submitted (11-MAR-2019) Coral Reef Research Center of China, School

of Marine Sciences, No. 100 Daxue Road, Xixiangtang District,

Nanning, Guangxi Zhuang Autonomous Region 530004, China

COMMENT ##Assembly-Data-START##

Sequencing Technology :: Sanger dideoxy sequencing

##Assembly-Data-END##

FEATURES Location/Qualifiers

source 1..640

/organism="Microbulbifer sp."

/mol_type="genomic DNA"

/strain="sD13"

/isolation_source="coral"

/db_xref="taxon:1908541"

rRNA <1..>640

/product="16S ribosomal RNA"

ORIGIN

1 gcggaacttc ttctgaacga gtagagcggc ggacgggtga gtaacgcgtg ggaaattgcc

61 cagtagtggg ggacaacatt cggaaacgga tgctaatacc gcatacgccc tacgggggaa

121 agcaggggat cttcggacct tgtgctattg gatatgcccg cgtcggatta gctagttggt

181 gaggtaatgg ctcaccaagg caacgatccg tagctggtct gagaggatga tcagccacac

241 tgggactgag acacggccca gactcctacg ggaggcagca gtggggaata ttggacaatg

301 ggcgcaagcc tgatccagcc atgccgcgtg tgtgaagaag gctctagggt tgtaaagcac

361 tttcagtagg gaggaaggcc ttaaagttaa tacctttgag gattgacgtt acctacagaa

421 gaagcaccgg ctaactccgt gccagcagcc gcggtaatac ggagggtgcg agcgttaatc

481 ggaattactg ggcgtaaagc gcgcgtaggc ggttagttaa gctggatgtg aaagccccgg

541 gctcaacctg ggaactgcat tcagaactgg ctggctagag tacgagagag ggtagtggaa

601 tttcctgtgt agcggtgaaa tgcgtagata taggaaggaa

//

LOCUS MK617679 640 bp DNA linear BCT 30-MAY-2019

DEFINITION Microbulbifer sp. strain sD19 16S ribosomal RNA gene, partial

sequence.

ACCESSION MK617679

VERSION MK617679

KEYWORDS .

SOURCE Microbulbifer sp.

ORGANISM Microbulbifer sp.

Bacteria; Proteobacteria; Gammaproteobacteria; Cellvibrionales;

Microbulbiferaceae; Microbulbifer.

REFERENCE 1 (bases 1 to 640)

AUTHORS Su,H., Huang,Q. and Yu,K.

TITLE Diversity of Cultivable Protease-Producing Bacteria

JOURNAL Unpublished

REFERENCE 2 (bases 1 to 640)

AUTHORS Su,H., Huang,Q. and Yu,K.

TITLE Direct Submission

JOURNAL Submitted (11-MAR-2019) Coral Reef Research Center of China, School

of Marine Sciences, No. 100 Daxue Road, Xixiangtang District,

Nanning, Guangxi Zhuang Autonomous Region 530004, China

COMMENT ##Assembly-Data-START##

Sequencing Technology :: Sanger dideoxy sequencing

##Assembly-Data-END##

FEATURES Location/Qualifiers

source 1..640

/organism="Microbulbifer sp."

/mol_type="genomic DNA"

/strain="sD19"

/isolation_source="coral"

/db_xref="taxon:1908541"

rRNA <1..>640

/product="16S ribosomal RNA"

ORIGIN

1 acgttccttc gggaacaagt agagcggcgg acgggtgagt aacgcgtggg aaattgccca

61 gtagtggggg acaacattcg gaaacggatg ctaataccgc atacgcccta cgggggaaag

121 caggggatct tcggaccttg cgctattgga tatgcccgcg tcggattagc tagttggtga

181 ggtaatggct caccaaggca acgatccgta gctggtctga gaggatgatc agccacactg

241 ggactgagac acggcccaga ctcctacggg aggcagcagt ggggaatatt ggacaatggg

301 cgcaagcctg atccagccat gccgcgtgtg tgaagaaggc cctagggttg taaagcactt

361 tcagtaggga ggaaggcctt aaagttaata cctttgagga ttgacgttac ctacagaaga

421 agcaccggct aactccgtgc cagcagccgc ggtaatacgg agggtgcgag cgttaatcgg

481 aattactggg cgtaaagcgc gcgtaggcgg ttagttaagc tggatgtgaa agccccgggc

541 tcaacctggg aactgcattc agaactggct ggctagagta cgagagaggg tagtggaatt

601 tcctgtgtag cggtgaaatg cgtagatata ggaaggaaca

//

LOCUS MK617680 640 bp DNA linear BCT 30-MAY-2019

DEFINITION Microbulbifer sp. strain sD24 16S ribosomal RNA gene, partial

sequence.

ACCESSION MK617680

VERSION MK617680

KEYWORDS .

SOURCE Microbulbifer sp.

ORGANISM Microbulbifer sp.

Bacteria; Proteobacteria; Gammaproteobacteria; Cellvibrionales;

Microbulbiferaceae; Microbulbifer.

REFERENCE 1 (bases 1 to 640)

AUTHORS Su,H., Huang,Q. and Yu,K.

TITLE Diversity of Cultivable Protease-Producing Bacteria

JOURNAL Unpublished

REFERENCE 2 (bases 1 to 640)

AUTHORS Su,H., Huang,Q. and Yu,K.

TITLE Direct Submission

JOURNAL Submitted (11-MAR-2019) Coral Reef Research Center of China, School

of Marine Sciences, No. 100 Daxue Road, Xixiangtang District,

Nanning, Guangxi Zhuang Autonomous Region 530004, China

COMMENT ##Assembly-Data-START##

Sequencing Technology :: Sanger dideoxy sequencing

##Assembly-Data-END##

FEATURES Location/Qualifiers

source 1..640

/organism="Microbulbifer sp."

/mol_type="genomic DNA"

/strain="sD24"

/isolation_source="coral"

/db_xref="taxon:1908541"

rRNA <1..>640

/product="16S ribosomal RNA"

ORIGIN

1 gaaagttctt cggaatgagt agagcggcgg acgggtgagt aatgcatagg aatctgccca

61 gtagtggggg atagcccggg gaaacccgga ttaataccgc atacgtccta cgggagaaag

121 caggggatct tcggaccttg cgctattgga tgagcctatg tcggattagc ttgttggtgg

181 ggtaatggcc caccaaggcg acgatccgta gctggtctga gaggatgatc agccacactg

241 gaactgagac acggtccaga ctcctacggg aggcagcagt ggggaatatt gcacaatggg

301 ggaaaccctg atgcagccat gccgcgtgtg tgaagaaggc cttcgggttg taaagcactt

361 tcagtaggga ggaaggccct aaagttaata cctttaggga ttgacgttac ctacagaaga

421 agcaccggct aactccgtgc cagcagccgc ggtaatacgg agggtgcgag cgttaatcgg

481 aattactggg cgtaaagcgc gcgtaggcgg ttagttaagc tggatgtgaa agccccgggc

541 tcaacctggg aactgcattc agaactggct ggctagagta cgagagaggg tagtggaatt

601 tcctgtgtag cggtgaaatg cgtagatata ggaaggaaca

//

LOCUS MK617681 640 bp DNA linear BCT 30-MAY-2019

DEFINITION Microbacterium sp. strain sD33 16S ribosomal RNA gene, partial

sequence.

ACCESSION MK617681

VERSION MK617681

KEYWORDS .

SOURCE Microbacterium sp.

ORGANISM Microbacterium sp.

Bacteria; Actinobacteria; Micrococcales; Microbacteriaceae;

Microbacterium.

REFERENCE 1 (bases 1 to 640)

AUTHORS Su,H., Huang,Q. and Yu,K.

TITLE Diversity of Cultivable Protease-Producing Bacteria

JOURNAL Unpublished

REFERENCE 2 (bases 1 to 640)

AUTHORS Su,H., Huang,Q. and Yu,K.

TITLE Direct Submission

JOURNAL Submitted (11-MAR-2019) Coral Reef Research Center of China, School

of Marine Sciences, No. 100 Daxue Road, Xixiangtang District,

Nanning, Guangxi Zhuang Autonomous Region 530004, China

COMMENT ##Assembly-Data-START##

Sequencing Technology :: Sanger dideoxy sequencing

##Assembly-Data-END##

FEATURES Location/Qualifiers

source 1..640

/organism="Microbacterium sp."

/mol_type="genomic DNA"

/strain="sD33"

/isolation_source="coral"

/db_xref="taxon:51671"

rRNA <1..>640

/product="16S ribosomal RNA"

ORIGIN

1 gaacacggag cttgctctgt gggatcagtg gcgaacgggt gagtaacacg tgagcaacct

61 gcccctgact ctgggataag cgctggaaac ggcgtctaat actggatatg tgacgtgatc

121 gcatggtctg cgtctggaaa gaatttcggt tggggatggg ctcgcggcct atcagcttgt

181 tggtgaggta atggctcacc aaggcgtcga cgggtagccg gcctgagagg gtgaccggcc

241 acactgggac tgagacacgg cccagactcc tacgggaggc agcagtgggg aatattgcac

301 aatgggcgca agcctgatgc agcaacgccg cgtgagggat gacggccttc gggttgtaaa

361 cctcttttag cagggaagaa gcgaaagtga cggtacctgc agaaaaagcg ccggctaact

421 acgtgccagc agccgcggta atacgtaggg cgcaagcgtt atccggaatt attgggcgta

481 aagagctcgt aggcggtttg tcgcgtctgc tgtgaaatcc ggaggctcaa cctccggcct

541 gcagtgggta cgggcagact agagtgcggt aggggagatt ggaattcctg gtgtagcggt

601 ggaatgcgca gatatcagga ggaacaccga tggcgaaggc

//

LOCUS MK617682 640 bp DNA linear BCT 30-MAY-2019

DEFINITION Pseudomonas sp. strain sD40 16S ribosomal RNA gene, partial

sequence.

ACCESSION MK617682

VERSION MK617682

KEYWORDS .

SOURCE Pseudomonas sp.

ORGANISM Pseudomonas sp.

Bacteria; Proteobacteria; Gammaproteobacteria; Pseudomonadales;

Pseudomonadaceae; Pseudomonas.

REFERENCE 1 (bases 1 to 640)

AUTHORS Su,H., Huang,Q. and Yu,K.

TITLE Diversity of Cultivable Protease-Producing Bacteria

JOURNAL Unpublished

REFERENCE 2 (bases 1 to 640)

AUTHORS Su,H., Huang,Q. and Yu,K.

TITLE Direct Submission

JOURNAL Submitted (11-MAR-2019) Coral Reef Research Center of China, School

of Marine Sciences, No. 100 Daxue Road, Xixiangtang District,

Nanning, Guangxi Zhuang Autonomous Region 530004, China

COMMENT ##Assembly-Data-START##

Sequencing Technology :: Sanger dideoxy sequencing

##Assembly-Data-END##

FEATURES Location/Qualifiers

source 1..640

/organism="Pseudomonas sp."

/mol_type="genomic DNA"

/strain="sD40"

/isolation_source="coral"

/db_xref="taxon:306"

rRNA <1..>640

/product="16S ribosomal RNA"

ORIGIN

1 tgagggagct tgctcctgga ttcagcggcg gacgggtgag taatgcctag gaatctgcct

61 ggtagtgggg gataacgtcc ggaaacgggc gctaataccg catacgtcct gagggagaaa

121 gtgggggatc ttcggacctc acgctatcag atgagcctag gtcggattag ctagttggtg

181 gggtaaaggc ctaccaaggc gacgatccgt aactggtctg agaggatgat cagtcacact

241 ggaactgaga cacggtccag actcctacgg gaggcagcag tggggaatat tggacaatgg

301 gcgaaagcct gatccagcca tgccgcgtgt gtgaagaagg tcttcggatt gtaaagcact

361 ttaagttggg aggaagggca gtaagttaat accttgctgt tttgacgtta ccaacagaat

421 aagcaccggc taacttcgtg ccagcagccg cggtaatacg aagggtgcaa gcgttaatcg

481 gaattactgg gcgtaaagcg cgcgtaggtg gttcagcaag ttggatgtga aatccccggg

541 ctcaacctgg gaactgcatc caaaactact gagctagagt acggtagagg gtggtggaat

601 ttcctgtgta gcggtgaaat gcgtagatat aggaaggaac

//

LOCUS MK617683 640 bp DNA linear BCT 30-MAY-2019

DEFINITION Exiguobacterium sp. strain sD43 16S ribosomal RNA gene, partial

sequence.

ACCESSION MK617683

VERSION MK617683

KEYWORDS .

SOURCE Exiguobacterium sp.

ORGANISM Exiguobacterium sp.

Bacteria; Firmicutes; Bacilli; Bacillales; Bacillales Family XII.

Incertae Sedis; Exiguobacterium.

REFERENCE 1 (bases 1 to 640)

AUTHORS Su,H., Huang,Q. and Yu,K.

TITLE Diversity of Cultivable Protease-Producing Bacteria

JOURNAL Unpublished

REFERENCE 2 (bases 1 to 640)

AUTHORS Su,H., Huang,Q. and Yu,K.

TITLE Direct Submission

JOURNAL Submitted (11-MAR-2019) Coral Reef Research Center of China, School

of Marine Sciences, No. 100 Daxue Road, Xixiangtang District,

Nanning, Guangxi Zhuang Autonomous Region 530004, China

COMMENT ##Assembly-Data-START##

Sequencing Technology :: Sanger dideoxy sequencing

##Assembly-Data-END##

FEATURES Location/Qualifiers

source 1..640

/organism="Exiguobacterium sp."

/mol_type="genomic DNA"

/strain="sD43"

/isolation_source="coral"

/db_xref="taxon:44751"

rRNA <1..>640

/product="16S ribosomal RNA"

ORIGIN

1 agcgcaggaa gtcgacggaa cccttcgggg ggaagtcgac ggaatgagcg gcggacgggt

61 gagtaacacg taaagaacct gccctcaggt ctgggataac cacgagaaat cggggctaat

121 accggatggg tcatcggacc gcatggtccg aggatgaaag gcgctccggc gtcgcctggg

181 gatggctttg cggtgcatta gctagttggt ggggtaatgg cccaccaagg cgacgatgca

241 tagccgacct gagagggtga tcggccacac tgggactgag acacggccca gactcctacg

301 ggaggcagca gtagggaatc ttccacaatg gacgaaagtc tgatggagca acgccgcgtg

361 aacgatgaag gccttcgggt cgtaaagttc tgttgtaagg gaagaacaag tgccgcaggc

421 aatggcggca ccttgacggt accttgcgag aaagccacgg ctaactacgt gccagcagcc

481 gcggtaatac gtaggtggca agcgttgtcc ggaattattg ggcgtaaagc gcgcgcaggc

541 ggcctcttaa gtctgatgtg aaagcccccg gctcaaccgg ggagggccat tggaaactgg

601 gaggcttgag tataggagag aagagtggaa ttccacgtgt

//

LOCUS MK617684 640 bp DNA linear BCT 30-MAY-2019

DEFINITION Fictibacillus sp. strain sD46 16S ribosomal RNA gene, partial

sequence.

ACCESSION MK617684

VERSION MK617684

KEYWORDS .

SOURCE Fictibacillus sp.

ORGANISM Fictibacillus sp.

Bacteria; Firmicutes; Bacilli; Bacillales; Bacillaceae;

Fictibacillus.

REFERENCE 1 (bases 1 to 640)

AUTHORS Su,H., Huang,Q. and Yu,K.

TITLE Diversity of Cultivable Protease-Producing Bacteria

JOURNAL Unpublished

REFERENCE 2 (bases 1 to 640)

AUTHORS Su,H., Huang,Q. and Yu,K.

TITLE Direct Submission

JOURNAL Submitted (11-MAR-2019) Coral Reef Research Center of China, School

of Marine Sciences, No. 100 Daxue Road, Xixiangtang District,

Nanning, Guangxi Zhuang Autonomous Region 530004, China

COMMENT ##Assembly-Data-START##

Sequencing Technology :: Sanger dideoxy sequencing

##Assembly-Data-END##

FEATURES Location/Qualifiers

source 1..640

/organism="Fictibacillus sp."

/mol_type="genomic DNA"

/strain="sD46"

/isolation_source="coral"

/db_xref="taxon:1871617"

rRNA <1..>640

/product="16S ribosomal RNA"

ORIGIN

1 cgaatgaaga ggagcttgct cctctgattt agcggcggac gggtgagtaa cacgtgggta

61 atctgcctgt aagacgggga taactccggg aaaccggggc taataccgga taataagaag

121 aaacgcatgt ttcttttttg aaagtcggtt tcggctgaca cttacagatg agcccgcggc

181 gcattagcta gttggtgagg taacggctca ccaaggcgac gatgcgtagc cgacctgaga

241 gggtgatcgg ccacactggg actgagacac ggcccagact cctacgggag gcagcagtag

301 ggaatcttcg gcaatgggcg aaagcctgac cgagcaacgc cgcgtgagcg atgaaggcct

361 tcgggtcgta aagctctgtt gttagagaag aacaagtacg agagtaactg ctcgtacctt

421 gacggtacct aaccagaaag ccacggctaa ctacgtgcca gcagccgcgg taatacgtag

481 gtggcaagcg ttatccggaa ttattgggcg taaagcgcgc gcaggcggtc tcttaagtct

541 gatgtgaaag cccacggctc aaccgtggag ggtcattgga aactgggaga cttgagtgca

601 ggagagaaaa gtggaattcc acgtgtagcg gtgaaatgcg

//

LOCUS MK617685 640 bp DNA linear BCT 30-MAY-2019

DEFINITION Ruegeria sp. strain sD55 16S ribosomal RNA gene, partial sequence.

ACCESSION MK617685

VERSION MK617685

KEYWORDS .

SOURCE Ruegeria sp.

ORGANISM Ruegeria sp.

Bacteria; Proteobacteria; Alphaproteobacteria; Rhodobacterales;

Rhodobacteraceae; Ruegeria.

REFERENCE 1 (bases 1 to 640)

AUTHORS Su,H., Huang,Q. and Yu,K.

TITLE Diversity of Cultivable Protease-Producing Bacteria

JOURNAL Unpublished

REFERENCE 2 (bases 1 to 640)

AUTHORS Su,H., Huang,Q. and Yu,K.

TITLE Direct Submission

JOURNAL Submitted (11-MAR-2019) Coral Reef Research Center of China, School

of Marine Sciences, No. 100 Daxue Road, Xixiangtang District,

Nanning, Guangxi Zhuang Autonomous Region 530004, China

COMMENT ##Assembly-Data-START##

Sequencing Technology :: Sanger dideoxy sequencing

##Assembly-Data-END##

FEATURES Location/Qualifiers

source 1..640

/organism="Ruegeria sp."

/mol_type="genomic DNA"

/strain="sD55"

/isolation_source="coral"

/db_xref="taxon:1879320"

rRNA <1..>640

/product="16S ribosomal RNA"

ORIGIN

1 cccttcgggg tgagcggcgg acgggttagt aacgcgtggg aatataccct ttggtacgga

61 atagcctctg gaaacggaga gtaataccgt atgtgccctt cgggggaaag atttatcgcc

121 aaaggattag cccgcgttag attaggtagt tggtggggta atggcctacc aagcctacga

181 tctatagctg gttttagagg atgatcagca acactgggac tgagacacgg cccagactcc

241 tacgggaggc agcagtgggg aatcttggac aatgggggca accctgatcc agccatgccg

301 cgtgagtgat gaaggcctta gggtcgtaaa gctctttcgc ctgtgaagat aatgacggta

361 gcaggtaaag aaaccccggc taactccgtg ccagcagccg cggtaatacg gagggggtta

421 gcgttgttcg gaattactgg gcgtaaagcg cacgtaggcg gatcggaaag ttgggggtga

481 aatcccgggg ctcaaccccg gaactgcctc caaaactatc ggtctagagt tcgagagagg

541 tgagtggaat tccgagtgta gaggtgaaat tcgtagatat tcggaggaac accagtggcg

601 aaggcggctc actggctcga tactgacgct gaggtgcgaa

//

LOCUS MK617686 640 bp DNA linear BCT 30-MAY-2019

DEFINITION Microbacterium sp. strain sF16b 16S ribosomal RNA gene, partial

sequence.

ACCESSION MK617686

VERSION MK617686

KEYWORDS .

SOURCE Microbacterium sp.

ORGANISM Microbacterium sp.

Bacteria; Actinobacteria; Micrococcales; Microbacteriaceae;

Microbacterium.

REFERENCE 1 (bases 1 to 640)

AUTHORS Su,H., Huang,Q. and Yu,K.

TITLE Diversity of Cultivable Protease-Producing Bacteria

JOURNAL Unpublished

REFERENCE 2 (bases 1 to 640)

AUTHORS Su,H., Huang,Q. and Yu,K.

TITLE Direct Submission

JOURNAL Submitted (11-MAR-2019) Coral Reef Research Center of China, School

of Marine Sciences, No. 100 Daxue Road, Xixiangtang District,

Nanning, Guangxi Zhuang Autonomous Region 530004, China

COMMENT ##Assembly-Data-START##

Sequencing Technology :: Sanger dideoxy sequencing

##Assembly-Data-END##

FEATURES Location/Qualifiers

source 1..640

/organism="Microbacterium sp."

/mol_type="genomic DNA"

/strain="sF16b"

/isolation_source="coral"

/db_xref="taxon:51671"

rRNA <1..>640

/product="16S ribosomal RNA"

ORIGIN

1 cacggagctt gctctgtggg atcagtggcg aacgggtgag taacacgtga gcaacctgcc

61 cctgactctg ggataagcgc tggaaacggc gtctaatact ggatatgtga cgtgatcgca

121 tggtctgcgt ctggaaagaa tttcggttgg ggatgggctc gcggcctatc agcttgttgg

181 tgaggtaatg gctcaccaag gcgtcgacgg gtagccggcc tgagagggtg accggccaca

241 ctgggactga gacacggccc agactcctac gggaggcagc agtggggaat attgcacaat

301 gggcgcaagc ctgatgcagc aacgccgcgt gagggatgac ggccttcggg ttgtaaacct

361 cttttagcag ggaagaagcg aaagtgacgg tacctgcaga aaaagcgccg gctaactacg

421 tgccagcagc cgcggtaata cgtagggcgc aagcgttatc cggaattatt gggcgtaaag

481 agctcgtagg cggtttgtcg cgtctgctgt gaaatccgga ggctcaacct ccggcctgca

541 gtgggtacgg gcagactaga gtgcggtagg ggagattgga attcctggtg tagcggtgga

601 atgcgcagat atcaggagga acaccgatgg cgaaggcaga

//

LOCUS MK617687 640 bp DNA linear BCT 30-MAY-2019

DEFINITION Micrococcus sp. strain sF19 16S ribosomal RNA gene, partial

sequence.

ACCESSION MK617687

VERSION MK617687

KEYWORDS .

SOURCE Micrococcus sp.

ORGANISM Micrococcus sp.

Bacteria; Actinobacteria; Micrococcales; Micrococcaceae;

Micrococcus.

REFERENCE 1 (bases 1 to 640)

AUTHORS Su,H., Huang,Q. and Yu,K.

TITLE Diversity of Cultivable Protease-Producing Bacteria

JOURNAL Unpublished

REFERENCE 2 (bases 1 to 640)

AUTHORS Su,H., Huang,Q. and Yu,K.

TITLE Direct Submission

JOURNAL Submitted (11-MAR-2019) Coral Reef Research Center of China, School

of Marine Sciences, No. 100 Daxue Road, Xixiangtang District,

Nanning, Guangxi Zhuang Autonomous Region 530004, China

COMMENT ##Assembly-Data-START##

Sequencing Technology :: Sanger dideoxy sequencing

##Assembly-Data-END##

FEATURES Location/Qualifiers

source 1..640

/organism="Micrococcus sp."

/mol_type="genomic DNA"

/strain="sF19"

/isolation_source="coral"

/db_xref="taxon:1271"

rRNA <1..>640

/product="16S ribosomal RNA"

ORIGIN

1 tgaagcccag cttgctgggt ggattagtgg cgaacgggtg agtaacacgt gagtaacctg

61 cccttaactc tgggataagc ctgggaaact gggtctaata ccggatagga gcgcctaccg

121 catggtgggt gttggaaaga tttatcggtt ttggatggac tcgcggccta tcagcttgtt

181 ggtgaggtaa tggctcacca aggcgacgac gggtagccgg cctgagaggg tgaccggcca

241 cactgggact gagacacggc ccagactcct acgggaggca gcagtgggga atattgcaca

301 atgggcgcaa gcctgatgca gcgacgccgc gtgagggatg acggccttcg ggttgtaaac

361 ctctttcagt agggaagaag cgaaagtgac ggtacctgca gaagaagcac cggctaacta

421 cgtgccagca gccgcggtaa tacgtagggt gcgagcgtta tccggaatta ttgggcgtaa

481 agagctcgta ggcggtttgt cgcgtctgtc gtgaaagtcc ggggcttaac cccggatctg

541 cggtgggtac gggcagacta gagtgcagta ggggagactg gaattcctgg tgtagcggtg

601 gaatgcgcag atatcaggag gaacaccgat ggcgaaggca

//

LOCUS MK617688 640 bp DNA linear BCT 30-MAY-2019

DEFINITION Sphingomonas sp. strain sF30a 16S ribosomal RNA gene, partial

sequence.

ACCESSION MK617688

VERSION MK617688

KEYWORDS .

SOURCE Sphingomonas sp.

ORGANISM Sphingomonas sp.

Bacteria; Proteobacteria; Alphaproteobacteria; Sphingomonadales;

Sphingomonadaceae; Sphingomonas.

REFERENCE 1 (bases 1 to 640)

AUTHORS Su,H., Huang,Q. and Yu,K.

TITLE Diversity of Cultivable Protease-Producing Bacteria

JOURNAL Unpublished

REFERENCE 2 (bases 1 to 640)

AUTHORS Su,H., Huang,Q. and Yu,K.

TITLE Direct Submission

JOURNAL Submitted (11-MAR-2019) Coral Reef Research Center of China, School

of Marine Sciences, No. 100 Daxue Road, Xixiangtang District,

Nanning, Guangxi Zhuang Autonomous Region 530004, China

COMMENT ##Assembly-Data-START##

Sequencing Technology :: Sanger dideoxy sequencing

##Assembly-Data-END##

FEATURES Location/Qualifiers

source 1..640

/organism="Sphingomonas sp."

/mol_type="genomic DNA"

/strain="sF30a"

/isolation_source="coral"

/db_xref="taxon:28214"

rRNA <1..>640

/product="16S ribosomal RNA"

ORIGIN

1 tcaaacttga gagtttgatc ctggctcaga acgaacgctg gcggcatgcc taacacatgc

61 aagtcgaacg aaggcttcgg ccttagtgcc gcacgggtgc gtaacgcgtg ggaatctgcc

121 cttaggttcg gaataacagc tggaaacggc tgctaatacc ggatgatatc gcgagatcaa

181 agatttatcg cctgaggatg agcccgcgtt ggattaggta gttggtgggg taatggccta

241 ccaagccgac gatccatagc tggtctgaga ggatgatcag ccacactggg actgagacac

301 ggcccagact cctacgggag gcagcagtgg ggaatattgg acaatgggcg aaagcctgat

361 ccagcaatgc cgcgtgagtg atgaaggccc tagggttgta aagctctttt acccgggaag

421 ataatgactg taccgggaga ataagccccg gctaactccg tgccagcagc cgcggtaata

481 cggagggggc tagcgttgtt cggaattact gggcgtaaag cgcacgtagg cggctttgta

541 agtcagaggt gaaagcctgg agctcaactc cagaactgcc tttgagactg catcgcttga

601 atccaggaga ggtcagtgga attccgagtg tagaggtgaa

//

LOCUS MK617689 640 bp DNA linear BCT 30-MAY-2019

DEFINITION Pseudovibrio sp. strain sF33 16S ribosomal RNA gene, partial

sequence.

ACCESSION MK617689

VERSION MK617689

KEYWORDS .

SOURCE Pseudovibrio sp.

ORGANISM Pseudovibrio sp.

Bacteria; Proteobacteria; Alphaproteobacteria; Rhodobacterales;

Rhodobacteraceae; Pseudovibrio.

REFERENCE 1 (bases 1 to 640)

AUTHORS Su,H., Huang,Q. and Yu,K.

TITLE Diversity of Cultivable Protease-Producing Bacteria

JOURNAL Unpublished

REFERENCE 2 (bases 1 to 640)

AUTHORS Su,H., Huang,Q. and Yu,K.

TITLE Direct Submission

JOURNAL Submitted (11-MAR-2019) Coral Reef Research Center of China, School

of Marine Sciences, No. 100 Daxue Road, Xixiangtang District,

Nanning, Guangxi Zhuang Autonomous Region 530004, China

COMMENT ##Assembly-Data-START##

Sequencing Technology :: Sanger dideoxy sequencing

##Assembly-Data-END##

FEATURES Location/Qualifiers

source 1..640

/organism="Pseudovibrio sp."

/mol_type="genomic DNA"

/strain="sF33"

/isolation_source="coral"

/db_xref="taxon:1909297"

rRNA <1..>640

/product="16S ribosomal RNA"

ORIGIN

1 gatccttcgg gattagtggc agacgggtga gtaacgcgtg ggaagctacc ttgtggtagg

61 gaacaacagt tggaaacgac tgctaatacc ctatgagccc tatgggggaa agatttatcg

121 ccatgagatg tgcccgcgtt agattagcta gttggtaagg taatggctta ccaaggcgac

181 gatctatagc tggtctgaga ggatgatcag ccacactggg actgagacac ggcccagact

241 cctacgggag gcagcagtgg ggaatattgg acaatggggg caaccctgat ccagccatgc

301 cgcgtgagtg atgacggcct tagggttgta aagctctttc agcagtgaag ataatgacat

361 taactgcaga agaagccccg gctaacttcg tgccagcagc cgcggtaata cgaagggggc

421 tagcgttgtt cggaatcact gggcgtaaag cgtacgtagg cggactgatc agtcaggggt

481 gaaatcccgg ggctcaaccc cggaactgcc tttgatactg tcagtcttga gatcgagaga

541 ggtgagtgga actccgagtg tagaggtgaa attcgtagat attcggaaga acaccagtgg

601 cgaaggcggc tcactggctc gatactgacg ctgaggtacg

//

LOCUS MK617690 640 bp DNA linear BCT 30-MAY-2019

DEFINITION Fictibacillus sp. strain sF33a 16S ribosomal RNA gene, partial

sequence.

ACCESSION MK617690

VERSION MK617690

KEYWORDS .

SOURCE Fictibacillus sp.

ORGANISM Fictibacillus sp.

Bacteria; Firmicutes; Bacilli; Bacillales; Bacillaceae;

Fictibacillus.

REFERENCE 1 (bases 1 to 640)

AUTHORS Su,H., Huang,Q. and Yu,K.

TITLE Diversity of Cultivable Protease-Producing Bacteria

JOURNAL Unpublished

REFERENCE 2 (bases 1 to 640)

AUTHORS Su,H., Huang,Q. and Yu,K.

TITLE Direct Submission

JOURNAL Submitted (11-MAR-2019) Coral Reef Research Center of China, School

of Marine Sciences, No. 100 Daxue Road, Xixiangtang District,

Nanning, Guangxi Zhuang Autonomous Region 530004, China

COMMENT ##Assembly-Data-START##

Sequencing Technology :: Sanger dideoxy sequencing

##Assembly-Data-END##

FEATURES Location/Qualifiers

source 1..640

/organism="Fictibacillus sp."

/mol_type="genomic DNA"

/strain="sF33a"

/isolation_source="coral"

/db_xref="taxon:1871617"

rRNA <1..>640

/product="16S ribosomal RNA"

ORIGIN

1 gaatgatgag gagcttgctc ctctgattta gcggcggacg ggtgagtaac acgtgggtaa

61 tctgcctgta agacggggat aactccggga aaccggggct aataccggat aataagagaa

121 gaagcatttc ttctttttga aagtcggttt cggctgacac ttacagatga gcccgcggcg

181 cattagctag ttggtgaggt aacggctcac caaggcgacg atgcgtagcc gacctgagag

241 ggtgatcggc cacactggga ctgagacacg gcccagactc ctacgggagg cagcagtagg

301 gaatcttcgg caatgggcga aagcctgacc gagcaacgcc gcgtgagcga tgaaggcctt

361 cgggtcgtaa agctctgttg ttagagaaga acaagtacga gagtaactgc tcgtaccttg

421 acggtaccta accagaaagc cacggctaac tacgtgccag cagccgcggt aatacgtagg

481 tggcaagcgt tatccggaat tattgggcgt aaagcgcgcg caggcggtct cttaagtctg

541 atgtgaaagc ccacggctca accgtggagg gtcattggaa actgggagac ttgagtgcag

601 gagagaaaag tggaattcca cgtgtagcgg tgaaatgcgt

//

LOCUS MK617691 640 bp DNA linear BCT 30-MAY-2019

DEFINITION Bacillus sp. (in: Bacteria) strain sJ2 16S ribosomal RNA gene,

partial sequence.

ACCESSION MK617691

VERSION MK617691

KEYWORDS .

SOURCE Bacillus sp. (in: Bacteria)

ORGANISM Bacillus sp. (in: Bacteria)

Bacteria; Firmicutes; Bacilli; Bacillales; Bacillaceae; Bacillus.

REFERENCE 1 (bases 1 to 640)

AUTHORS Su,H., Huang,Q. and Yu,K.

TITLE Diversity of Cultivable Protease-Producing Bacteria

JOURNAL Unpublished

REFERENCE 2 (bases 1 to 640)

AUTHORS Su,H., Huang,Q. and Yu,K.

TITLE Direct Submission

JOURNAL Submitted (11-MAR-2019) Coral Reef Research Center of China, School

of Marine Sciences, No. 100 Daxue Road, Xixiangtang District,

Nanning, Guangxi Zhuang Autonomous Region 530004, China

COMMENT ##Assembly-Data-START##

Sequencing Technology :: Sanger dideoxy sequencing

##Assembly-Data-END##

FEATURES Location/Qualifiers

source 1..640

/organism="Bacillus sp. (in: Bacteria)"

/mol_type="genomic DNA"

/strain="sJ2"

/isolation_source="coral"

/db_xref="taxon:1409"

rRNA <1..>640

/product="16S ribosomal RNA"

ORIGIN

1 cgaactgatt agaagcttgc ttctatgacg ttagcggcgg acgggtgagt aacacgtggg

61 caacctgcct gtaagactgg gataacttcg ggaaaccgaa gctaataccg gataggatct

121 tctccttcat gggagatgat tgaaagatgg tttcggctat cacttacaga tgggcccgcg

181 gtgcattagc tagttggtga ggtaacggct caccaaggca acgatgcata gccgacctga

241 gagggtgatc ggccacactg ggactgagac acggcccaga ctcctacggg aggcagcagt

301 agggaatctt ccgcaatgga cgaaagtctg acggagcaac gccgcgtgag tgatgaaggc

361 tttcgggtcg taaaactctg ttgttaggga agaacaagta caagagtaac tgcttgtacc

421 ttgacggtac ctaaccagaa agccacggct aactacgtgc cagcagccgc ggtaatacgt

481 aggtggcaag cgttatccgg aattattggg cgtaaagcgc gcgcaggcgg tttcttaagt

541 ctgatgtgaa agcccacggc tcaaccgtgg agggtcattg gaaactgggg aacttgagtg

601 cagaagagaa aagcggaatt ccacgtgtag cggtgaaatg

//

LOCUS MK617692 640 bp DNA linear BCT 30-MAY-2019

DEFINITION Fictibacillus sp. strain sJ6 16S ribosomal RNA gene, partial

sequence.

ACCESSION MK617692

VERSION MK617692

KEYWORDS .

SOURCE Fictibacillus sp.

ORGANISM Fictibacillus sp.

Bacteria; Firmicutes; Bacilli; Bacillales; Bacillaceae;

Fictibacillus.

REFERENCE 1 (bases 1 to 640)

AUTHORS Su,H., Huang,Q. and Yu,K.

TITLE Diversity of Cultivable Protease-Producing Bacteria

JOURNAL Unpublished

REFERENCE 2 (bases 1 to 640)

AUTHORS Su,H., Huang,Q. and Yu,K.

TITLE Direct Submission

JOURNAL Submitted (11-MAR-2019) Coral Reef Research Center of China, School

of Marine Sciences, No. 100 Daxue Road, Xixiangtang District,

Nanning, Guangxi Zhuang Autonomous Region 530004, China

COMMENT ##Assembly-Data-START##

Sequencing Technology :: Sanger dideoxy sequencing

##Assembly-Data-END##

FEATURES Location/Qualifiers

source 1..640

/organism="Fictibacillus sp."

/mol_type="genomic DNA"

/strain="sJ6"

/isolation_source="coral"

/db_xref="taxon:1871617"

rRNA <1..>640

/product="16S ribosomal RNA"

ORIGIN

1 atgatgagga gcttgctcct ctgatttagc ggcggacggg tgagtaacac gtgggtaatc

61 tgcctgtaag acggggataa ctccgggaaa ccggggctaa taccggataa taagagaaga

121 agcatttctt ctttttgaaa gtcggtttcg gctgacactt acagatgagc ccgcggcgca

181 ttagctagtt ggtgaggtaa cggctcacca aggcgacgat gcgtagccga cctgagaggg

241 tgatcggcca cactgggact gagacacggc ccagactcct acgggaggca gcagtaggga

301 atcttcggca atgggcgaaa gcctgaccga gcaacgccgc gtgagcgatg aaggccttcg

361 ggtcgtaaag ctctgttgtt agagaagaac aagtacgaga gtaactgctc gtaccttgac

421 ggtacctaac cagaaagcca cggctaacta cgtgccagca gccgcggtaa tacgtaggtg

481 gcaagcgtta tccggaatta ttgggcgtaa agcgcgcgca ggcggtctct taagtctgat

541 gtgaaagccc acggctcaac cgtggagggt cattggaaac tgggagactt gagtgcagga

601 gagaaaagtg gaattccacg tgtagcggtg aaatgcgtag

//

LOCUS MK617693 640 bp DNA linear BCT 30-MAY-2019

DEFINITION Fictibacillus sp. strain sJ10 16S ribosomal RNA gene, partial

sequence.

ACCESSION MK617693

VERSION MK617693

KEYWORDS .

SOURCE Fictibacillus sp.

ORGANISM Fictibacillus sp.

Bacteria; Firmicutes; Bacilli; Bacillales; Bacillaceae;

Fictibacillus.

REFERENCE 1 (bases 1 to 640)

AUTHORS Su,H., Huang,Q. and Yu,K.

TITLE Diversity of Cultivable Protease-Producing Bacteria

JOURNAL Unpublished

REFERENCE 2 (bases 1 to 640)

AUTHORS Su,H., Huang,Q. and Yu,K.

TITLE Direct Submission

JOURNAL Submitted (11-MAR-2019) Coral Reef Research Center of China, School

of Marine Sciences, No. 100 Daxue Road, Xixiangtang District,

Nanning, Guangxi Zhuang Autonomous Region 530004, China

COMMENT ##Assembly-Data-START##

Sequencing Technology :: Sanger dideoxy sequencing

##Assembly-Data-END##

FEATURES Location/Qualifiers

source 1..640

/organism="Fictibacillus sp."

/mol_type="genomic DNA"

/strain="sJ10"

/isolation_source="coral"

/db_xref="taxon:1871617"

rRNA <1..>640

/product="16S ribosomal RNA"

ORIGIN

1 tgaagaggag cttgctcctc tgatttagcg gcggacgggt gagtaacacg tgggtaatct

61 gcctgtaaga cggggataac tccgggaaac cggggctaat accggataat aagaagaaac

121 gcatgtttct tttttgaaag tcggtttcgg ctgacactta cagatgagcc cgcggcgcat

181 tagctagttg gtgaggtaac ggctcaccaa ggcgacgatg cgtagccgac ctgagagggt

241 gatcggccac actgggactg agacacggcc cagactccta cgggaggcag cagtagggaa

301 tcttcggcaa tgggcgaaag cctgaccgag caacgccgcg tgagcgatga aggccttcgg

361 gtcgtaaagc tctgttgtta gagaagaaca agtacgagag taactgctcg taccttgacg

421 gtacctaacc agaaagccac ggctaactac gtgccagcag ccgcggtaat acgtaggtgg

481 caagcgttat ccggaattat tgggcgtaaa gcgcgcgcag gcggtctctt aagtctgatg

541 tgaaagccca cggctcaacc gtggagggtc attggaaact gggagacttg agtgcaggag

601 agaaaagtgg aattccacgt gtagcggtga aatgcgtaga

//

LOCUS MK617694 640 bp DNA linear BCT 30-MAY-2019

DEFINITION Fictibacillus sp. strain sJ15 16S ribosomal RNA gene, partial

sequence.

ACCESSION MK617694

VERSION MK617694

KEYWORDS .

SOURCE Fictibacillus sp.

ORGANISM Fictibacillus sp.

Bacteria; Firmicutes; Bacilli; Bacillales; Bacillaceae;

Fictibacillus.

REFERENCE 1 (bases 1 to 640)

AUTHORS Su,H., Huang,Q. and Yu,K.

TITLE Diversity of Cultivable Protease-Producing Bacteria

JOURNAL Unpublished

REFERENCE 2 (bases 1 to 640)

AUTHORS Su,H., Huang,Q. and Yu,K.

TITLE Direct Submission

JOURNAL Submitted (11-MAR-2019) Coral Reef Research Center of China, School

of Marine Sciences, No. 100 Daxue Road, Xixiangtang District,

Nanning, Guangxi Zhuang Autonomous Region 530004, China

COMMENT ##Assembly-Data-START##

Sequencing Technology :: Sanger dideoxy sequencing

##Assembly-Data-END##

FEATURES Location/Qualifiers

source 1..640

/organism="Fictibacillus sp."

/mol_type="genomic DNA"

/strain="sJ15"

/isolation_source="coral"

/db_xref="taxon:1871617"

rRNA <1..>640

/product="16S ribosomal RNA"

ORIGIN

1 atgaagagga gcttgctcct ctgatttagc ggcggacggg tgagtaacac gtgggtaatc

61 tgcctgtaag acggggataa ctccgggaaa ccggggctaa taccggataa taagaagaaa

121 cgcatgtttc ttttttgaaa gtcggtttcg gctgacactt acagatgagc ccgcggcgca

181 ttagctagtt ggtgaggtaa cggctcacca aggcgacgat gcgtagccga cctgagaggg

241 tgatcggcca cactgggact gagacacggc ccagactcct acgggaggca gcagtaggga

301 atcttcggca atgggcgaaa gcctgaccga gcaacgccgc gtgagcgatg aaggccttcg

361 ggtcgtaaag ctctgttgtt agagaagaac aagtacgaga gtaactgctc gtaccttgac

421 ggtacctaac cagaaagcca cggctaacta cgtgccagca gccgcggtaa tacgtaggtg

481 gcaagcgtta tccggaatta ttgggcgtaa agcgcgcgca ggcggtctct taagtctgat

541 gtgaaagccc acggctcaac cgtggagggt cattggaaac tgggagactt gagtgcagga

601 gagaaaagtg gaattccacg tgtagcggtg aaatgcgtag

//

LOCUS MK617695 640 bp DNA linear BCT 30-MAY-2019

DEFINITION Pseudomonas sp. strain sB27a 16S ribosomal RNA gene, partial

sequence.

ACCESSION MK617695

VERSION MK617695

KEYWORDS .

SOURCE Pseudomonas sp.

ORGANISM Pseudomonas sp.

Bacteria; Proteobacteria; Gammaproteobacteria; Pseudomonadales;

Pseudomonadaceae; Pseudomonas.

REFERENCE 1 (bases 1 to 640)

AUTHORS Su,H., Huang,Q. and Yu,K.

TITLE Diversity of Cultivable Protease-Producing Bacteria

JOURNAL Unpublished

REFERENCE 2 (bases 1 to 640)

AUTHORS Su,H., Huang,Q. and Yu,K.

TITLE Direct Submission

JOURNAL Submitted (11-MAR-2019) Coral Reef Research Center of China, School

of Marine Sciences, No. 100 Daxue Road, Xixiangtang District,

Nanning, Guangxi Zhuang Autonomous Region 530004, China

COMMENT ##Assembly-Data-START##

Sequencing Technology :: Sanger dideoxy sequencing

##Assembly-Data-END##

FEATURES Location/Qualifiers

source 1..640

/organism="Pseudomonas sp."

/mol_type="genomic DNA"

/strain="sB27a"

/isolation_source="coral"

/db_xref="taxon:306"

rRNA <1..>640

/product="16S ribosomal RNA"

ORIGIN

1 gaacgctggc ggcaggccta acacatgcaa gtcgagcgga tgaagggagc ttgctctctg

61 attcagcggc ggacgggtga gtaatgccta ggaatctgcc tggtagtggg ggacaacgtt

121 tcgaaaggaa cgctaatacc gcatacgtcc tacgggagaa agtgggggat cttcggacct

181 cacgctatca gatgagccta ggtcggatta gctagtaggt gaggtaatgg ctcacctagg

241 cgacgatccg taactggtct gagaggatga tcagtcacac tggaactgag acacggtcca

301 gactcctacg ggaggcagca gtggggaata ttggacaatg ggcgaaagcc tgatccagcc

361 atgccgcgtg tgtgaagaag gtcttcggat tgtaaagcac tttaagttgg gaggaagggc

421 agtaagttaa taccttgctg ttttgacgtt accaacagaa taagcaccgg ctaacttcgt

481 gccagcagcc gcggtaatac gaagggtgca agcgttaatc ggaattactg ggcgtaaagc

541 gcgcgtaggt ggtttggtaa gatggatgtg aaatccccgg gctcaacctg ggaactgcat

601 ccataactgc ctgactagag tacggtagag ggtggtggaa

//

LOCUS MK617696 640 bp DNA linear BCT 30-MAY-2019

DEFINITION Alteromonas sp. strain yL6aB 16S ribosomal RNA gene, partial

sequence.

ACCESSION MK617696

VERSION MK617696

KEYWORDS .

SOURCE Alteromonas sp.

ORGANISM Alteromonas sp.

Bacteria; Proteobacteria; Gammaproteobacteria; Alteromonadales;

Alteromonadaceae; Alteromonas.

REFERENCE 1 (bases 1 to 640)

AUTHORS Su,H., Huang,Q. and Yu,K.

TITLE Diversity of Cultivable Protease-Producing Bacteria

JOURNAL Unpublished

REFERENCE 2 (bases 1 to 640)

AUTHORS Su,H., Huang,Q. and Yu,K.

TITLE Direct Submission

JOURNAL Submitted (11-MAR-2019) Coral Reef Research Center of China, School

of Marine Sciences, No. 100 Daxue Road, Xixiangtang District,

Nanning, Guangxi Zhuang Autonomous Region 530004, China

COMMENT ##Assembly-Data-START##

Sequencing Technology :: Sanger dideoxy sequencing

##Assembly-Data-END##

FEATURES Location/Qualifiers

source 1..640

/organism="Alteromonas sp."

/mol_type="genomic DNA"

/strain="yL6aB"

/isolation_source="coral"

/db_xref="taxon:232"

rRNA <1..>640

/product="16S ribosomal RNA"

ORIGIN

1 ttgaacgctg gcggcaggcc taacacatgc aagtcgaacg gtaacatttc taccttgcta

61 gaagatgacg agtggcggac gggtgagtaa tggttgggaa cttgcctttg cgagggggat

121 aacagttgga aacgactgct aataacgcat aatgtcttcg gaccaaacgg ggcttcggct

181 ccggctcaaa gagaggccca agtgagatta gctagttggt aaggtaacgg cttaccaagg

241 cgacgatctc tagctgttct gagaggtaga tcagccacac tgggactgag acacggccca

301 gactcctacg ggaggcagca gtggggaata ttgcacaatg ggggaaaccc tgatgcagcc

361 atgccgcgtg tgtgaagaag gccttcgggt tgtaaagtac tttcagttgt gacgaaaagt

421 tagtagttaa tacctgctag ccgtgacgtt aacaacagaa gaagcaccgg ctaactccgt

481 gccagcagcc gcggtaatac ggagggtgcg agcgttaatc ggaattactc ggcgtaaagc

541 gcacgcaggc ggtttgttaa gctagatgtg aaagccccgg gctcaacctg ggatggtcat

601 ttagaactgg cagactagag tcttggagag gggagtggaa

//

LOCUS MK617697 640 bp DNA linear BCT 30-MAY-2019

DEFINITION Microbacterium sp. strain yB24b 16S ribosomal RNA gene, partial

sequence.

ACCESSION MK617697

VERSION MK617697

KEYWORDS .

SOURCE Microbacterium sp.

ORGANISM Microbacterium sp.

Bacteria; Actinobacteria; Micrococcales; Microbacteriaceae;

Microbacterium.

REFERENCE 1 (bases 1 to 640)

AUTHORS Su,H., Huang,Q. and Yu,K.

TITLE Diversity of Cultivable Protease-Producing Bacteria

JOURNAL Unpublished

REFERENCE 2 (bases 1 to 640)

AUTHORS Su,H., Huang,Q. and Yu,K.

TITLE Direct Submission

JOURNAL Submitted (11-MAR-2019) Coral Reef Research Center of China, School

of Marine Sciences, No. 100 Daxue Road, Xixiangtang District,

Nanning, Guangxi Zhuang Autonomous Region 530004, China

COMMENT ##Assembly-Data-START##

Sequencing Technology :: Sanger dideoxy sequencing

##Assembly-Data-END##

FEATURES Location/Qualifiers

source 1..640

/organism="Microbacterium sp."

/mol_type="genomic DNA"

/strain="yB24b"

/isolation_source="coral"

/db_xref="taxon:51671"

rRNA <1..>640

/product="16S ribosomal RNA"

ORIGIN

1 agtcgaacgg tgaacacgga gcttgctctg tgggatcagt ggcgaacggg tgagtaacac

61 gtgagcaacc tgcccctgac tctgggataa gcgctggaaa cggcgtctaa tactggatat

121 gtgacgtgat cgcatggtct gcgtctggaa agaatttcgg ttggggatcg gctcgcggcc

181 tatcagcttg ttggtgaggt aatggctcat caaggcgtcg acgggtagcc ggcctgagag

241 ggtgaccggc cacactggga ctgagacacg gcccagactc ctacgggagg cagcagtggg

301 gaatattgca caatgggcgc aagcctgatg cagcaacgcc gcgtgaggga tgacggcctt

361 cgggttgtaa acctctttta gcagggaaga agcgaaagtg acggtacctg cagaaaaagc

421 gccggctatc tacgtgccag cagccgcggt aatacgtagg gcgcaagcgt tatccggaat

481 tattgggcgt aaagagctcg taggcggttt gtcgcgtctg ctgtgaaatc cggaggctca

541 acctccggcc tgcagtgggt acgggcagac tagagtgcgg taggggagat tggaattcct

601 ggtgtagcgg tggaatgcgc agatatcagg aggaacaccg

//

LOCUS MK617698 640 bp DNA linear BCT 30-MAY-2019

DEFINITION Paracoccus sp. strain yA2c 16S ribosomal RNA gene, partial

sequence.

ACCESSION MK617698

VERSION MK617698

KEYWORDS .

SOURCE Paracoccus sp.

ORGANISM Paracoccus sp.

Bacteria; Proteobacteria; Alphaproteobacteria; Rhodobacterales;

Rhodobacteraceae; Paracoccus.

REFERENCE 1 (bases 1 to 640)

AUTHORS Su,H., Huang,Q. and Yu,K.

TITLE Diversity of Cultivable Protease-Producing Bacteria

JOURNAL Unpublished

REFERENCE 2 (bases 1 to 640)

AUTHORS Su,H., Huang,Q. and Yu,K.

TITLE Direct Submission

JOURNAL Submitted (11-MAR-2019) Coral Reef Research Center of China, School

of Marine Sciences, No. 100 Daxue Road, Xixiangtang District,

Nanning, Guangxi Zhuang Autonomous Region 530004, China

COMMENT ##Assembly-Data-START##

Sequencing Technology :: Sanger dideoxy sequencing

##Assembly-Data-END##

FEATURES Location/Qualifiers

source 1..640

/organism="Paracoccus sp."

/mol_type="genomic DNA"

/strain="yA2c"

/isolation_source="coral"

/db_xref="taxon:267"

rRNA <1..>640

/product="16S ribosomal RNA"

ORIGIN

1 tcgagcgaga ccttcgggtc tagcggcgga cgggtgagta acgcgtggga acgtgccctt

61 ctctacggaa tagccccggg aaactgggag taataccgta tacgcccttt gggggaaaga

121 tttatcggag aaggatcggc ccgcgttgga ttaggtagtt ggtggggtaa tggcccacca

181 agccgacgat ccatagctgg tttgagagga tgatcagcca cactgggact gagacacggc

241 ccagactcct acgggaggca gcagtgggga atcttagaca atgggggcaa ccctgatcta

301 gccatgccgc gtgagtgatg aaggccttag ggttgtaaag ctctttcagc tgggaagata

361 atgacggtac cagcagaaga agccccggct aactccgtgc cagcagccgc ggtaatacgg

421 agggggctag cgttgttcgg aattactggg cgtaaagcgc acgtaggcgg actggaaagt

481 cagaggtgaa atcccagggc tcaaccttgg aactgccttt gaaactatca gtctggagtt

541 cgagagaggt gagtggaatt ccgagtgtag aggtgaaatt cgtagatatt cggaggaaca

601 ccagtggcga aggcggctca ctggctcgat actgacgctg

//

LOCUS MK617699 640 bp DNA linear BCT 30-MAY-2019

DEFINITION Vibrio sp. strain F16 16S ribosomal RNA gene, partial sequence.

ACCESSION MK617699

VERSION MK617699

KEYWORDS .

SOURCE Vibrio sp.

ORGANISM Vibrio sp.

Bacteria; Proteobacteria; Gammaproteobacteria; Vibrionales;

Vibrionaceae; Vibrio.

REFERENCE 1 (bases 1 to 640)

AUTHORS Su,H., Huang,Q. and Yu,K.

TITLE Diversity of Cultivable Protease-Producing Bacteria

JOURNAL Unpublished

REFERENCE 2 (bases 1 to 640)

AUTHORS Su,H., Huang,Q. and Yu,K.

TITLE Direct Submission

JOURNAL Submitted (11-MAR-2019) Coral Reef Research Center of China, School

of Marine Sciences, No. 100 Daxue Road, Xixiangtang District,

Nanning, Guangxi Zhuang Autonomous Region 530004, China

COMMENT ##Assembly-Data-START##

Sequencing Technology :: Sanger dideoxy sequencing

##Assembly-Data-END##

FEATURES Location/Qualifiers

source 1..640

/organism="Vibrio sp."

/mol_type="genomic DNA"

/strain="F16"

/isolation_source="coral"

/db_xref="taxon:678"

rRNA <1..>640

/product="16S ribosomal RNA"

ORIGIN

1 agcggaaacg agttatctga accttcgggg gacgataacg gcgtcgagcg gcggacgggt

61 gagtaatgcc taggaaattg ccctgatgtg ggggataacc attggaaacg atggctaata

121 ccgcataatg cctacgggcc aaagaggggg accttcgggc ctctcgcgtc aggatatgcc

181 taggtgggat tagctagttg gtgaggtaat ggctcaccaa ggcgacgatc cctagctggt

241 ctgagaggat gatcagccac actggaactg agacacggtc cagactccta cgggaggcag

301 cagtggggaa tattgcacaa tgggcgcaag cctgatgcag ccatgccgcg tgtgtgaaga

361 aggccttcgg gttgtaaagc actttcagtc gtgaggaagg tagtgtagtt aatagctgca

421 ttacttgacg ttagcgacag aagaagcacc ggctaactcc gtgccagcag ccgcggtaat

481 acggagggtg cgagcgttaa tcggaattac tgggcgtaaa gcgcatgcag gtggtttgtt

541 aagtcagatg tgaaagcccg gggctcaacc tcggaatagc atttgaaact ggcagactag

601 agtactgtag aggggggtag aatttcaggt gtagcggtga

//

LOCUS MK617700 640 bp DNA linear BCT 30-MAY-2019

DEFINITION Vibrio sp. strain yF30 16S ribosomal RNA gene, partial sequence.

ACCESSION MK617700

VERSION MK617700

KEYWORDS .

SOURCE Vibrio sp.

ORGANISM Vibrio sp.

Bacteria; Proteobacteria; Gammaproteobacteria; Vibrionales;

Vibrionaceae; Vibrio.

REFERENCE 1 (bases 1 to 640)

AUTHORS Su,H., Huang,Q. and Yu,K.

TITLE Diversity of Cultivable Protease-Producing Bacteria

JOURNAL Unpublished

REFERENCE 2 (bases 1 to 640)

AUTHORS Su,H., Huang,Q. and Yu,K.

TITLE Direct Submission

JOURNAL Submitted (11-MAR-2019) Coral Reef Research Center of China, School

of Marine Sciences, No. 100 Daxue Road, Xixiangtang District,

Nanning, Guangxi Zhuang Autonomous Region 530004, China

COMMENT ##Assembly-Data-START##

Sequencing Technology :: Sanger dideoxy sequencing

##Assembly-Data-END##

FEATURES Location/Qualifiers

source 1..640

/organism="Vibrio sp."

/mol_type="genomic DNA"

/strain="yF30"

/isolation_source="coral"

/db_xref="taxon:678"

rRNA <1..>640

/product="16S ribosomal RNA"

ORIGIN

1 agcggaaacg agttatctga accttcgggg gacgataacg gcgtcgagcg gcggacgggt

61 gagtaatgcc taggaaattg ccctgatgtg ggggataacc attggaaacg atggctaata

121 ccgcataatg cctacgggcc aaagaggggg accttcgggc ctctcgcgtc aggatatgcc

181 taggtgggat tagctagttg gtgaggtaat ggctcaccaa ggcgacgatc cctagctggt

241 ctgagaggat gatcagccac actggaactg agacacggtc cagactccta cgggaggcag

301 cagtggggaa tattgcacaa tgggcgcaag cctgatgcag ccatgccgcg tgtgtgaaga

361 aggccttcgg gttgtaaagc actttcagtc gtgaggaagg tagtgtagtt aatagctgca

421 ttacttgacg ttagcgacag aagaagcacc ggctaactcc gtgccagcag ccgcggtaat

481 acggagggtg cgagcgttaa tcggaattac tgggcgtaaa gcgcatgcag gtggtttgtt

541 aagtcagatg tgaaagcccg gggctcaacc tcggaatagc atttgaaact ggcagactag

601 agtactgtag aggggggtag aatttcaggt gtagcggtga

//

LOCUS MK617701 640 bp DNA linear BCT 30-MAY-2019

DEFINITION Bacillus sp. (in: Bacteria) strain Q33 16S ribosomal RNA gene,

partial sequence.

ACCESSION MK617701

VERSION MK617701

KEYWORDS .

SOURCE Bacillus sp. (in: Bacteria)

ORGANISM Bacillus sp. (in: Bacteria)

Bacteria; Firmicutes; Bacilli; Bacillales; Bacillaceae; Bacillus.

REFERENCE 1 (bases 1 to 640)

AUTHORS Su,H., Huang,Q. and Yu,K.

TITLE Diversity of Cultivable Protease-Producing Bacteria

JOURNAL Unpublished

REFERENCE 2 (bases 1 to 640)

AUTHORS Su,H., Huang,Q. and Yu,K.

TITLE Direct Submission

JOURNAL Submitted (11-MAR-2019) Coral Reef Research Center of China, School

of Marine Sciences, No. 100 Daxue Road, Xixiangtang District,

Nanning, Guangxi Zhuang Autonomous Region 530004, China

COMMENT ##Assembly-Data-START##

Sequencing Technology :: Sanger dideoxy sequencing

##Assembly-Data-END##

FEATURES Location/Qualifiers

source 1..640

/organism="Bacillus sp. (in: Bacteria)"

/mol_type="genomic DNA"

/strain="Q33"

/isolation_source="coral"

/db_xref="taxon:1409"

rRNA <1..>640

/product="16S ribosomal RNA"

ORIGIN

1 cgagcggaca gatgggagct tgctccctga agtcagcggc ggacgggtga gtaacacgtg

61 ggcaacctgc ctgtaagact gggataactc cgggaaaccg gggctaatac cggataattc

121 tttccctcac atgagggaaa gctgaaagat ggtttcggct atcacttaca gatgggcccg

181 cggcgcatta gctagttggt gaggtaacgg ctcaccaagg caacgatgcg tagccgacct

241 gagagggtga tcggccacac tgggactgag acacggccca gactcctacg ggaggcagca

301 gtagggaatc ttccgcaatg gacgaaagtc tgacggagca acgccgcgtg agtgatgaag

361 gttttcggat cgtaaaactc tgttgttagg gaagaacaag taccggagta actgccggta

421 ccttgacggt acctaaccag aaagccacgg ctaactacgt gccagcagcc gcggtaatac

481 gtaggtggca agcgttgtcc ggaattattg ggcgtaaagc gcgcgcaggc ggttccttaa

541 gtctgatgtg aaagcccccg gctcaaccgg ggagggtcat tggaaactgg ggaacttgag

601 tgcagaagag aagagtggaa ttccacgtgt agcggtgaaa

//

LOCUS MK617702 640 bp DNA linear BCT 30-MAY-2019

DEFINITION Stenotrophomonas sp. strain Q35 16S ribosomal RNA gene, partial

sequence.

ACCESSION MK617702

VERSION MK617702

KEYWORDS .

SOURCE Stenotrophomonas sp.

ORGANISM Stenotrophomonas sp.

Bacteria; Proteobacteria; Gammaproteobacteria; Xanthomonadales;

Xanthomonadaceae; Stenotrophomonas.

REFERENCE 1 (bases 1 to 640)

AUTHORS Su,H., Huang,Q. and Yu,K.

TITLE Diversity of Cultivable Protease-Producing Bacteria

JOURNAL Unpublished

REFERENCE 2 (bases 1 to 640)

AUTHORS Su,H., Huang,Q. and Yu,K.

TITLE Direct Submission

JOURNAL Submitted (11-MAR-2019) Coral Reef Research Center of China, School

of Marine Sciences, No. 100 Daxue Road, Xixiangtang District,

Nanning, Guangxi Zhuang Autonomous Region 530004, China

COMMENT ##Assembly-Data-START##

Sequencing Technology :: Sanger dideoxy sequencing

##Assembly-Data-END##

FEATURES Location/Qualifiers

source 1..640

/organism="Stenotrophomonas sp."

/mol_type="genomic DNA"

/strain="Q35"

/isolation_source="coral"

/db_xref="taxon:69392"

rRNA <1..>640

/product="16S ribosomal RNA"

ORIGIN

1 tcgaacggca gcacaggaga gcttgctctc tgggtggcga gtggcggacg ggtgaggaat

61 acatcggaat ctactttttc gtgggggata acgtagggaa acttacgcta ataccgcata

121 cgacctacgg gtgaaagcag gggatcttcg gaccttgcgc gattgaatga gccgatgtcg

181 gattagctag ttggcggggt aaaggcccac caaggcgacg atccgtagct ggtctgagag

241 gatgatcagc cacactggaa ctgagacacg gtccagactc ctacgggagg cagcagtggg

301 gaatattgga caatgggcgc aagcctgatc cagccatacc gcgtgggtga agaaggcctt

361 cgggttgtaa agcccttttg ttgggaaaga aatccagctg gctaataccc ggttgggatg

421 acggtaccca aagaataagc accggctaac ttcgtgccag cagccgcggt aatacgaagg

481 gtgcaagcgt tactcggaat tactgggcgt aaagcgtgcg taggtggtcg tttaagtccg

541 ttgtgaaagc cctgggctca acctgggaac tgcagtggat actgggcgac tagagtgtgg

601 tagagggtag cggaattcct ggtgtagcag tgaaatgcgt

//

LOCUS MK617703 640 bp DNA linear BCT 30-MAY-2019

DEFINITION Bacillus sp. (in: Bacteria) strain Q36 16S ribosomal RNA gene,

partial sequence.

ACCESSION MK617703

VERSION MK617703

KEYWORDS .

SOURCE Bacillus sp. (in: Bacteria)

ORGANISM Bacillus sp. (in: Bacteria)

Bacteria; Firmicutes; Bacilli; Bacillales; Bacillaceae; Bacillus.

REFERENCE 1 (bases 1 to 640)

AUTHORS Su,H., Huang,Q. and Yu,K.

TITLE Diversity of Cultivable Protease-Producing Bacteria

JOURNAL Unpublished

REFERENCE 2 (bases 1 to 640)

AUTHORS Su,H., Huang,Q. and Yu,K.

TITLE Direct Submission

JOURNAL Submitted (11-MAR-2019) Coral Reef Research Center of China, School

of Marine Sciences, No. 100 Daxue Road, Xixiangtang District,

Nanning, Guangxi Zhuang Autonomous Region 530004, China

COMMENT ##Assembly-Data-START##

Sequencing Technology :: Sanger dideoxy sequencing

##Assembly-Data-END##

FEATURES Location/Qualifiers

source 1..640

/organism="Bacillus sp. (in: Bacteria)"

/mol_type="genomic DNA"

/strain="Q36"

/isolation_source="coral"

/db_xref="taxon:1409"

rRNA <1..>640

/product="16S ribosomal RNA"

ORIGIN

1 gtcgagcgga cagatgggag cttgctccct gaagtcagcg gcggacgggt gagtaacacg

61 tgggcaacct gcctgtaaga ctgggataac tccgggaaac cggggctaat accggataat

121 tctttccctc acatgaggga aagctgaaag atggtttcgg ctatcactta cagatgggcc

181 cgcggcgcat tagctagttg gtgaggtaac ggctcaccaa ggcaacgatg cgtagccgac

241 ctgagagggt gatcggccac actgggactg agacacggcc cagactccta cgggaggcag

301 cagtagggaa tcttccgcaa tggacgaaag tctgacggag caacgccgcg tgagtgatga

361 aggttttcgg atcgtaaaac tctgttgtta gggaagaaca agtaccggag taactgccgg

421 taccttgacg gtacctaacc agaaagccac ggctaactac gtgccagcag ccgcggtaat

481 acgtaggtgg caagcgttgt ccggaattat tgggcgtaaa gcgcgcgcag gcggttcctt

541 aagtctgatg tgaaagcccc cggctcaacc ggggagggtc attggaaact ggggaacttg

601 agtgcagaag agaagagtgg aattccacgt gtagcggtga

//

LOCUS MK617704 640 bp DNA linear BCT 30-MAY-2019

DEFINITION Bacillus sp. (in: Bacteria) strain sJ20 16S ribosomal RNA gene,

partial sequence.

ACCESSION MK617704

VERSION MK617704

KEYWORDS .

SOURCE Bacillus sp. (in: Bacteria)

ORGANISM Bacillus sp. (in: Bacteria)

Bacteria; Firmicutes; Bacilli; Bacillales; Bacillaceae; Bacillus.

REFERENCE 1 (bases 1 to 640)

AUTHORS Su,H., Huang,Q. and Yu,K.

TITLE Diversity of Cultivable Protease-Producing Bacteria

JOURNAL Unpublished

REFERENCE 2 (bases 1 to 640)

AUTHORS Su,H., Huang,Q. and Yu,K.

TITLE Direct Submission

JOURNAL Submitted (11-MAR-2019) Coral Reef Research Center of China, School

of Marine Sciences, No. 100 Daxue Road, Xixiangtang District,

Nanning, Guangxi Zhuang Autonomous Region 530004, China

COMMENT ##Assembly-Data-START##

Sequencing Technology :: Sanger dideoxy sequencing

##Assembly-Data-END##

FEATURES Location/Qualifiers

source 1..640

/organism="Bacillus sp. (in: Bacteria)"

/mol_type="genomic DNA"

/strain="sJ20"

/isolation_source="coral"

/db_xref="taxon:1409"

rRNA <1..>640

/product="16S ribosomal RNA"

ORIGIN

1 gagaggagct tgctcctctg atttagcggc ggacgggtga gtaacacgtg ggtaatctgc

61 ctgtaagacg gggataactc cgggaaaccg gggctaatac cggataataa gagaagaagc

121 atttcttctt tttgaaagtc ggtttcggct gacacttaca gatgagcccg cggcgcatta

181 gctagttggt gaggtaacgg ctcaccaagg cgacgatgcg tagccgacct gagagggtga

241 tcggccacac tgggactgaa acacggccca aactcctacg ggaggcagca gtagggaatc

301 ttcggcaatg ggcgaaagcc tgaccgagca acgccgcgtg agcgatgaag gccttcgggt

361 cgtaaagctc tgttgttaga gaagaacaag tacgagagta actgctcgta ccttgacggt

421 acctaaccag aaagccacgg ctaactacgt gccagcagcc gcggtaatac gtatgtggca

481 agtgttatcc ggaattattg ggcgtaaagc gcgcgcaggc ggtctcttaa gtctgatgtg

541 aaagcccacg gctcacccgt ggagggtcat tggaaactgg gagacttgag tgcaagagag

601 aaaagtggaa ttccacgtgt agcggtgaaa tgcgtagaga

//

LOCUS MK617705 640 bp DNA linear BCT 30-MAY-2019

DEFINITION Fictibacillus sp. strain sJ27 16S ribosomal RNA gene, partial

sequence.

ACCESSION MK617705

VERSION MK617705

KEYWORDS .

SOURCE Fictibacillus sp.

ORGANISM Fictibacillus sp.

Bacteria; Firmicutes; Bacilli; Bacillales; Bacillaceae;

Fictibacillus.

REFERENCE 1 (bases 1 to 640)

AUTHORS Su,H., Huang,Q. and Yu,K.

TITLE Diversity of Cultivable Protease-Producing Bacteria

JOURNAL Unpublished

REFERENCE 2 (bases 1 to 640)

AUTHORS Su,H., Huang,Q. and Yu,K.

TITLE Direct Submission

JOURNAL Submitted (11-MAR-2019) Coral Reef Research Center of China, School

of Marine Sciences, No. 100 Daxue Road, Xixiangtang District,

Nanning, Guangxi Zhuang Autonomous Region 530004, China

COMMENT ##Assembly-Data-START##

Sequencing Technology :: Sanger dideoxy sequencing

##Assembly-Data-END##

FEATURES Location/Qualifiers

source 1..640

/organism="Fictibacillus sp."

/mol_type="genomic DNA"

/strain="sJ27"

/isolation_source="coral"

/db_xref="taxon:1871617"

rRNA <1..>640

/product="16S ribosomal RNA"

ORIGIN

1 tgaagaggag cttgctcctc tgatttagcg gcggacgggt gagtaacacg tgggtaatct

61 gcctgtaaga cggggataac tccgggaaac cggggctaat accggataat aagaagaaac

121 gcatgtttct tttttgaaag tcggtttcgg ctgacactta cagatgagcc cgcggcgcat

181 tagctagttg gtgaggtaac ggctcaccaa ggcgacgatg cgtagccgac ctgagagggt

241 gatcggccac actgggactg agacacggcc cagactccta cgggaggcag cagtagggaa

301 tcttcggcaa tgggcgaaag cctgaccgag caacgccgcg tgagcgatga aggccttcgg

361 gtcgtaaagc tctgttgtta gagaagaaca agtacgagag taactgctcg taccttgacg

421 gtacctaacc agaaagccac ggctaactac gtgccagcag ccgcggtaat acgtaggtgg

481 caagcgttat ccggaattat tgggcgtaaa gcgcgcgcag gcggtctctt aagtctgatg

541 tgaaagccca cggctcaacc gtggagggtc attggaaact gggagacttg agtgcaggag

601 agaaaagtgg aattccacgt gtagcggtga aatgcgtaga

//

LOCUS MK617706 640 bp DNA linear BCT 30-MAY-2019

DEFINITION Bacillus sp. (in: Bacteria) strain sJ36 16S ribosomal RNA gene,

partial sequence.

ACCESSION MK617706

VERSION MK617706

KEYWORDS .

SOURCE Bacillus sp. (in: Bacteria)

ORGANISM Bacillus sp. (in: Bacteria)

Bacteria; Firmicutes; Bacilli; Bacillales; Bacillaceae; Bacillus.

REFERENCE 1 (bases 1 to 640)

AUTHORS Su,H., Huang,Q. and Yu,K.

TITLE Diversity of Cultivable Protease-Producing Bacteria

JOURNAL Unpublished

REFERENCE 2 (bases 1 to 640)

AUTHORS Su,H., Huang,Q. and Yu,K.

TITLE Direct Submission

JOURNAL Submitted (11-MAR-2019) Coral Reef Research Center of China, School

of Marine Sciences, No. 100 Daxue Road, Xixiangtang District,

Nanning, Guangxi Zhuang Autonomous Region 530004, China

COMMENT ##Assembly-Data-START##

Sequencing Technology :: Sanger dideoxy sequencing

##Assembly-Data-END##

FEATURES Location/Qualifiers

source 1..640

/organism="Bacillus sp. (in: Bacteria)"

/mol_type="genomic DNA"

/strain="sJ36"

/isolation_source="coral"

/db_xref="taxon:1409"

rRNA <1..>640

/product="16S ribosomal RNA"

ORIGIN

1 gaaccaatga ggagcttgct cgctgaagtt agcggcggac gcgtgagtaa cacgtgcgta

61 acctgcctgt aagattggca taactccggc aatccggagc taataccgga taatattttg

121 aaccgcatgg ttcttatttg aaagatggtt tcggctgtca cttacagatg gacccgcggc

181 gcattagcta gttggtgagg taacggctca ccaaggcgac gatgcgtagc cgacctgaga

241 gggtgatcgg ccacactggg actgagacac ggcccagact cctacgggag gcagcagtac

301 ggaatctacc gcaatggacg aaagcctgac cgagcaacgc cgcgtgaacg atgaaggcct

361 tcgggtcgta aagttctgtt gttagagaag aacaagtacc agagtatctg ctggtacctt

421 gaccgtacct aaccagaaag ccacgcctaa ctacgtgcca gcagccgccg taatacgtag

481 gtgccaagcg taatccggat atattgggcg ttaagcgggc gcaggccgtc tcttaagtct

541 gatgtgtaag cccacggctc aaccgtggac ggtcattgga aactggcaga ctagagtgca

601 ggagagaaaa gtggaattcc acgtgtagcg gtgaaatgcg

//

LOCUS MK617707 640 bp DNA linear BCT 30-MAY-2019

DEFINITION Roseobacter sp. strain sJ37 16S ribosomal RNA gene, partial

sequence.

ACCESSION MK617707

VERSION MK617707

KEYWORDS .

SOURCE Roseobacter sp.

ORGANISM Roseobacter sp.

Bacteria; Proteobacteria; Alphaproteobacteria; Rhodobacterales;

Rhodobacteraceae; Roseobacter.

REFERENCE 1 (bases 1 to 640)

AUTHORS Su,H., Huang,Q. and Yu,K.

TITLE Diversity of Cultivable Protease-Producing Bacteria

JOURNAL Unpublished

REFERENCE 2 (bases 1 to 640)

AUTHORS Su,H., Huang,Q. and Yu,K.

TITLE Direct Submission

JOURNAL Submitted (11-MAR-2019) Coral Reef Research Center of China, School

of Marine Sciences, No. 100 Daxue Road, Xixiangtang District,

Nanning, Guangxi Zhuang Autonomous Region 530004, China

COMMENT ##Assembly-Data-START##

Sequencing Technology :: Sanger dideoxy sequencing

##Assembly-Data-END##

FEATURES Location/Qualifiers

source 1..640

/organism="Roseobacter sp."

/mol_type="genomic DNA"

/strain="sJ37"

/isolation_source="coral"

/db_xref="taxon:1907202"

rRNA <1..>640

/product="16S ribosomal RNA"

ORIGIN

1 gcagctacca tgcaagtcga gcgagatctt cggatctagc ggcggacggg tgagtaacgc

61 gtgggaacgt gcccttctct acggaatagt cccgggaaac tgggtttaat accgtatacg

121 cccttcgggg gaaagattta tcggagaagg atcggcccgc gttggattag gtagttggtg

181 gggtaatggc ctaccaagcc gacgatccat agctggtttg agaggatgat cagccacact

241 gggactgaga cacggcccag actcctacgg gaggcagcag tggggaatct tagacaatgg

301 gggcaaccct gatctagcca tgccgcgtga gtgatgaagg ccttagggtc gtaaagctct

361 ttcgccaggg atgataatga cagtacctgg taaagaaacc ccggctaact ccgtgccagc

421 agccgcggta atacggaggg ggttagcgtt gttcggaatt actgggcgta aagcgcacgt

481 aggcggatta gtcagtcaga ggtgaaatcc cagggctcaa ccctggaact gcctttgata

541 ctgctagtct tgagttcgag agaggtgagt ggaattccga gtgtagaggt gaaattcgta

601 gatattcgga ggaacaccag tggcgaaggc ggctcactgg

//

LOCUS MK617708 640 bp DNA linear BCT 30-MAY-2019

DEFINITION Microbulbifer sp. strain sJ50 16S ribosomal RNA gene, partial

sequence.

ACCESSION MK617708

VERSION MK617708

KEYWORDS .

SOURCE Microbulbifer sp.

ORGANISM Microbulbifer sp.

Bacteria; Proteobacteria; Gammaproteobacteria; Cellvibrionales;

Microbulbiferaceae; Microbulbifer.

REFERENCE 1 (bases 1 to 640)

AUTHORS Su,H., Huang,Q. and Yu,K.

TITLE Diversity of Cultivable Protease-Producing Bacteria

JOURNAL Unpublished

REFERENCE 2 (bases 1 to 640)

AUTHORS Su,H., Huang,Q. and Yu,K.

TITLE Direct Submission

JOURNAL Submitted (11-MAR-2019) Coral Reef Research Center of China, School

of Marine Sciences, No. 100 Daxue Road, Xixiangtang District,

Nanning, Guangxi Zhuang Autonomous Region 530004, China

COMMENT ##Assembly-Data-START##

Sequencing Technology :: Sanger dideoxy sequencing

##Assembly-Data-END##

FEATURES Location/Qualifiers

source 1..640

/organism="Microbulbifer sp."

/mol_type="genomic DNA"

/strain="sJ50"

/isolation_source="coral"

/db_xref="taxon:1908541"

rRNA <1..>640

/product="16S ribosomal RNA"

ORIGIN

1 acgttccttc gggaacaagt agagcggcgg acgggtgagt aacgcgtggg aaattgccca

61 gtagtggggg acaacattcg gaaacggatg ctaataccgc atacgcccta cgggggaaag

121 caggggatct tcggaccttg cgctattgga tatgcccgcg tcggattagc tagttggtga

181 ggtaatggct caccaaggca acgatccgta gctggtctga gaggatgatc agccacactg

241 ggactgagac acggcccaga ctcctacggg aggcagcagt ggggaatatt ggacaatggg

301 cgcaagcctg atccagccat gccgcgtgtg tgaagaaggc cctagggttg taaagcactt

361 tcagtaggga ggaaggcctt aaagttaata cctttgagga ttgacgttac ctacagaaga

421 agcaccggct aactccgtgc cagcagccgc ggtaatacgg agggtgcgag cgttaatcgg

481 aattactggg cgtaaagcgc gcgtaggcgg ttagttaagc tggatgtgaa agccccgggc

541 tcaacctggg aactgcattc agaactggct ggctagagta cgagagaggg tagtggaatt

601 tcctgtgtag cggtgaaatg cgtagatata ggaaggaaca

//

LOCUS MK617709 640 bp DNA linear BCT 30-MAY-2019

DEFINITION Bacillus sp. (in: Bacteria) strain sJ51 16S ribosomal RNA gene,

partial sequence.

ACCESSION MK617709

VERSION MK617709

KEYWORDS .

SOURCE Bacillus sp. (in: Bacteria)

ORGANISM Bacillus sp. (in: Bacteria)

Bacteria; Firmicutes; Bacilli; Bacillales; Bacillaceae; Bacillus.

REFERENCE 1 (bases 1 to 640)

AUTHORS Su,H., Huang,Q. and Yu,K.

TITLE Diversity of Cultivable Protease-Producing Bacteria

JOURNAL Unpublished

REFERENCE 2 (bases 1 to 640)

AUTHORS Su,H., Huang,Q. and Yu,K.

TITLE Direct Submission

JOURNAL Submitted (11-MAR-2019) Coral Reef Research Center of China, School

of Marine Sciences, No. 100 Daxue Road, Xixiangtang District,

Nanning, Guangxi Zhuang Autonomous Region 530004, China

COMMENT ##Assembly-Data-START##

Sequencing Technology :: Sanger dideoxy sequencing

##Assembly-Data-END##

FEATURES Location/Qualifiers

source 1..640

/organism="Bacillus sp. (in: Bacteria)"

/mol_type="genomic DNA"

/strain="sJ51"

/isolation_source="coral"

/db_xref="taxon:1409"

rRNA <1..>640

/product="16S ribosomal RNA"

ORIGIN

1 ggattaagag cttgctctta tgaagttagc ggcggacggg tgagtaacac gtgggtaacc

61 tgcccataag actgggataa ctccgggaaa ccggggctaa taccggataa cattttgaac

121 cgcatggttc gaaattgaaa ggcggcttcg gctgtcactt atggatggac ccgcgtcgca

181 ttagctagtt ggtgaggtaa cggctcacca aggcaacgat gcgtagccga cctgagaggg

241 tgatcggcca cactgggact gagacacggc ccagactcct acgggaggca gcagtaggga

301 atcttccgca atggacgaaa gtctgacgga gcaacgccgc gtgagtgatg aaggctttcg

361 ggtcgtaaaa ctctgttgtt agggaagaac aagtgctagt tgaataagct ggcaccttga

421 cggtacctaa ccagaaagcc acggctaact acgtgccagc agccgcggta atacgtaggt

481 ggcaagcgtt atccggaatt attgggcgta aagcgcgcgc aggtggtttc ttaagtctga

541 tgtgaaagcc cacggctcaa ccgtggaggg tcattggaaa ctgggagact tgagtgcaga

601 agaggaaagt ggaattccat gtgtagcggt gaaatgcgta

//

LOCUS MK617710 640 bp DNA linear BCT 30-MAY-2019

DEFINITION Aquimarina sp. strain sJ58 16S ribosomal RNA gene, partial

sequence.

ACCESSION MK617710

VERSION MK617710

KEYWORDS .

SOURCE Aquimarina sp.

ORGANISM Aquimarina sp.

Bacteria; Bacteroidetes; Flavobacteriia; Flavobacteriales;

Flavobacteriaceae; Aquimarina.

REFERENCE 1 (bases 1 to 640)

AUTHORS Su,H., Huang,Q. and Yu,K.

TITLE Diversity of Cultivable Protease-Producing Bacteria

JOURNAL Unpublished

REFERENCE 2 (bases 1 to 640)

AUTHORS Su,H., Huang,Q. and Yu,K.

TITLE Direct Submission

JOURNAL Submitted (11-MAR-2019) Coral Reef Research Center of China, School

of Marine Sciences, No. 100 Daxue Road, Xixiangtang District,

Nanning, Guangxi Zhuang Autonomous Region 530004, China

COMMENT ##Assembly-Data-START##

Sequencing Technology :: Sanger dideoxy sequencing

##Assembly-Data-END##

FEATURES Location/Qualifiers

source 1..640

/organism="Aquimarina sp."

/mol_type="genomic DNA"

/strain="sJ58"

/isolation_source="coral"

/db_xref="taxon:1872586"

rRNA <1..>640

/product="16S ribosomal RNA"

ORIGIN

1 ggtaacaggg ttgcttgcaa ccgctgacga ccggcgcacg ggtgcgtaac gcgtatagaa

61 cctacctttt actggagaat agcccagaga aatttggatt aatgctccat agtattattt

121 agtggcatca cttagtaatt aaagatttat tggtaaaaga tggctatgcg ttctattagc

181 tagttggtat ggtaacggca taccaaggct acgatagata ggggtcctga gagggagatc

241 ccccacactg gtactgagac acggaccaga ctcctacggg aggcagcagt gaggaatatt

301 ggacaatgga ggcaactctg atccagccat gccgcgtgta ggaagactgc cctatgggtt

361 gtaaactact tttatagagg aagaaacccc ggtatgtata ccggtctgac ggtactctac

421 gaataaggat cggctaactc cgtgccagca gccgcggtaa tacggaggat ccaagcgtta

481 tccggaatca ttgggtttaa agggtccgta ggcgggtcag taagtcagtg gtgaaagttt

541 tcggctcaac cggaaaattg ccattgatac tgctggtctt gaattattgt gaagtggtta

601 gaatgtgtag tgtagcggtg aaatgcatag atattacaca

//

LOCUS MK617711 640 bp DNA linear BCT 30-MAY-2019

DEFINITION Vibrio sp. strain sL1 16S ribosomal RNA gene, partial sequence.

ACCESSION MK617711

VERSION MK617711

KEYWORDS .

SOURCE Vibrio sp.

ORGANISM Vibrio sp.

Bacteria; Proteobacteria; Gammaproteobacteria; Vibrionales;

Vibrionaceae; Vibrio.

REFERENCE 1 (bases 1 to 640)

AUTHORS Su,H., Huang,Q. and Yu,K.

TITLE Diversity of Cultivable Protease-Producing Bacteria

JOURNAL Unpublished

REFERENCE 2 (bases 1 to 640)

AUTHORS Su,H., Huang,Q. and Yu,K.

TITLE Direct Submission

JOURNAL Submitted (11-MAR-2019) Coral Reef Research Center of China, School

of Marine Sciences, No. 100 Daxue Road, Xixiangtang District,

Nanning, Guangxi Zhuang Autonomous Region 530004, China

COMMENT ##Assembly-Data-START##

Sequencing Technology :: Sanger dideoxy sequencing

##Assembly-Data-END##

FEATURES Location/Qualifiers

source 1..640

/organism="Vibrio sp."

/mol_type="genomic DNA"

/strain="sL1"

/isolation_source="coral"

/db_xref="taxon:678"

rRNA <1..>640

/product="16S ribosomal RNA"

ORIGIN

1 ggaacgagtt atctgaacct tcggggaacg ataacggcgt cgagcggcgg acgggtgagt

61 aatgcctagg aaattgccct gatgtggggg ataaccattg gaaacgatgg ctaataccgc

121 ataatacctt cgggtcaaag agggggacct tcgggcctct cgcgtcagga tatgcctagg

181 tgggattagc tagttggtga ggtaatggct caccaaggcg acgatcccta gctggtctga

241 gaggatgatc agccacactg gaactgagac acggtccaga ctcctacggg aggcagcagt

301 ggggaatatt gcacaatggg cgcaagcctg atgcagccat gccgcgtgtg tgaagaaggc

361 cttcgggttg taaagcactt tcagtcgtga ggaaggtggt agtgttaata gcactatcat

421 ttgacgttag cgacagaaga agcaccggct aactccgtgc cagcagccgc ggtaatacgg

481 agggtgcgag cgttaatcgg aattactggg cgtaaagcgc atgcaggtgg tttgttaagt

541 cagatgtgaa agcccggggc tcaacctcgg aatagcattt gaaactggca gactagagta

601 ctgtagaggg gggtagaatt tcaggtgtag cggtgaaatg

//

LOCUS MK617712 640 bp DNA linear BCT 30-MAY-2019

DEFINITION Vibrio sp. strain sL3 16S ribosomal RNA gene, partial sequence.

ACCESSION MK617712

VERSION MK617712

KEYWORDS .

SOURCE Vibrio sp.

ORGANISM Vibrio sp.

Bacteria; Proteobacteria; Gammaproteobacteria; Vibrionales;

Vibrionaceae; Vibrio.

REFERENCE 1 (bases 1 to 640)

AUTHORS Su,H., Huang,Q. and Yu,K.

TITLE Diversity of Cultivable Protease-Producing Bacteria

JOURNAL Unpublished

REFERENCE 2 (bases 1 to 640)

AUTHORS Su,H., Huang,Q. and Yu,K.

TITLE Direct Submission

JOURNAL Submitted (11-MAR-2019) Coral Reef Research Center of China, School

of Marine Sciences, No. 100 Daxue Road, Xixiangtang District,

Nanning, Guangxi Zhuang Autonomous Region 530004, China

COMMENT ##Assembly-Data-START##

Sequencing Technology :: Sanger dideoxy sequencing

##Assembly-Data-END##

FEATURES Location/Qualifiers

source 1..640

/organism="Vibrio sp."

/mol_type="genomic DNA"

/strain="sL3"

/isolation_source="coral"

/db_xref="taxon:678"

rRNA <1..>640

/product="16S ribosomal RNA"

ORIGIN

1 gaaacgagtt atctgaacct tcagggaagg ataacggcgg cgagcggcgg acgggtgagt

61 aatgcctagg aaattgccct ggtgtggggg ataaccattg gaaacgatgg ctaataccgc

121 ataatacctt cgggtcaaag agggggacct tcgggcctct cgcgtcagga tatgcctagg

181 tgggattagc tagttggaga ggtaatggct caccaaggcg acgatcccta gctggtctga

241 gaggatgatc agccacactg gaactgagac acggtccaga ctcctacggg aggcagcagt

301 ggggaatatt gcacaatggg cgcaagcctg atgcacccat gccgcgtgtg tgaagaaggc

361 cttcgggttg taaagcactt tcagtcgtga ggaaggtggt agtgttaata gcactatcat

421 ttgacgttag cgacagaaga agcaccggct aactccgtgc cagcagccgc ggtaatacgg

481 agggtgcgag cgttaatcgg aattactggg cgtaaagcgc atgcaggtgg tttgttaagt

541 cagatgtgaa agcccggggc tcaacctcgg aatagcattt gaaactggca gactagagta

601 ctgtagaggg gggtagaatt tcaggtgtag cggagaaatg

//

LOCUS MK617713 640 bp DNA linear BCT 30-MAY-2019

DEFINITION Microbulbifer sp. strain sL24 16S ribosomal RNA gene, partial

sequence.

ACCESSION MK617713

VERSION MK617713

KEYWORDS .

SOURCE Microbulbifer sp.

ORGANISM Microbulbifer sp.

Bacteria; Proteobacteria; Gammaproteobacteria; Cellvibrionales;

Microbulbiferaceae; Microbulbifer.

REFERENCE 1 (bases 1 to 640)

AUTHORS Su,H., Huang,Q. and Yu,K.

TITLE Diversity of Cultivable Protease-Producing Bacteria

JOURNAL Unpublished

REFERENCE 2 (bases 1 to 640)

AUTHORS Su,H., Huang,Q. and Yu,K.

TITLE Direct Submission

JOURNAL Submitted (11-MAR-2019) Coral Reef Research Center of China, School

of Marine Sciences, No. 100 Daxue Road, Xixiangtang District,

Nanning, Guangxi Zhuang Autonomous Region 530004, China

COMMENT ##Assembly-Data-START##

Sequencing Technology :: Sanger dideoxy sequencing

##Assembly-Data-END##

FEATURES Location/Qualifiers

source 1..640

/organism="Microbulbifer sp."

/mol_type="genomic DNA"

/strain="sL24"

/isolation_source="coral"

/db_xref="taxon:1908541"

rRNA <1..>640

/product="16S ribosomal RNA"

ORIGIN

1 acgttccttc gggaacaagt agagcggcgg acgggtgagt aacgcgtggg aaattgccca

61 gtagtggggg acaacattcg gaaacggatg ctaataccgc atacgcccta cgggggaaag

121 caggggatct tcggaccttg cgctattgga tatgcccgcg tcggattagc tagttggtga

181 ggtaatggct caccaaggca acgatccgta gctggtctga gaggatgatc agccacactg

241 ggactgagac acggcccaga ctcctacggg aggcagcagt ggggaatatt ggacaatggg

301 cgcaagcctg atccagccat gccgcgtgtg tgaagaaggc cctagggttg taaagcactt

361 tcagtaggga ggaaggcctt aaagttaata cctttgagga ttgacgttac ctacagaaga

421 agcaccggct aactccgtgc cagcagccgc ggtaatacgg agggtgcgag cgttaatcgg

481 aattactggg cgtaaagcgc gcgtaggcgg ttagttaagc tggatgtgaa agccccgggc

541 tcaacctggg aactgcattc agaactggct ggctagagta cgagagaggg tagtggaatt

601 tcctgtgtag cggtgaaatg cgtagatata ggaaggaaca

//

LOCUS MK617714 640 bp DNA linear BCT 30-MAY-2019

DEFINITION Paenisporosarcina sp. strain sL25 16S ribosomal RNA gene, partial

sequence.

ACCESSION MK617714

VERSION MK617714

KEYWORDS .

SOURCE Paenisporosarcina sp.

ORGANISM Paenisporosarcina sp.

Bacteria; Firmicutes; Bacilli; Bacillales; Planococcaceae;

Paenisporosarcina.

REFERENCE 1 (bases 1 to 640)

AUTHORS Su,H., Huang,Q. and Yu,K.

TITLE Diversity of Cultivable Protease-Producing Bacteria

JOURNAL Unpublished

REFERENCE 2 (bases 1 to 640)

AUTHORS Su,H., Huang,Q. and Yu,K.

TITLE Direct Submission

JOURNAL Submitted (11-MAR-2019) Coral Reef Research Center of China, School

of Marine Sciences, No. 100 Daxue Road, Xixiangtang District,

Nanning, Guangxi Zhuang Autonomous Region 530004, China

COMMENT ##Assembly-Data-START##

Sequencing Technology :: Sanger dideoxy sequencing

##Assembly-Data-END##

FEATURES Location/Qualifiers

source 1..640

/organism="Paenisporosarcina sp."

/mol_type="genomic DNA"

/strain="sL25"

/isolation_source="coral"

/db_xref="taxon:1932001"

rRNA <1..>640

/product="16S ribosomal RNA"

ORIGIN

1 gaatgatgaa gaagcttgct tcttctgatt ttagcggcgg acgggtgagt aacacgtggg

61 caacctacct tgtagattgg gataactccg ggaaaccggg gctaatacca aataatccat

121 tttgcttcat ggcgaaatgt taaaaggcgg cttcggctgt cactacgaga tgggcccgcg

181 gcgcattagc tagttggtag ggtaacggcc taccaaggcg acgatgcgta gccgacctga

241 gagggtgatc ggccacactg ggactgagac acggcccaga ctcctacggg aggcagcagt

301 agggaatctt ccacaatgga cgaaagtctg atggagcaac gccgcgtgag tgaagaaggt

361 tttcggatcg taaaactctg ttgtaaggga agaacacgta cgagagtaac tgctcgtacc

421 ttgacggtac cttattagaa agccacggct aactacgtgc cagcagccgc ggtaatacgt

481 aggtggcaag cgttgtccgg aattattggg cgtaaagcgc gcgcaggcgg tcctttaagt

541 ctgatgtgaa agcccacggc tcaaccgtgg agggtcattg gaaactgggg gacttgagta

601 cagaagagga aagcggaatt ccaagtgtag cggtgaaatg

//

LOCUS MK617715 640 bp DNA linear BCT 30-MAY-2019

DEFINITION Vibrio sp. strain sL26 16S ribosomal RNA gene, partial sequence.

ACCESSION MK617715

VERSION MK617715

KEYWORDS .

SOURCE Vibrio sp.

ORGANISM Vibrio sp.

Bacteria; Proteobacteria; Gammaproteobacteria; Vibrionales;

Vibrionaceae; Vibrio.

REFERENCE 1 (bases 1 to 640)

AUTHORS Su,H., Huang,Q. and Yu,K.

TITLE Diversity of Cultivable Protease-Producing Bacteria

JOURNAL Unpublished

REFERENCE 2 (bases 1 to 640)

AUTHORS Su,H., Huang,Q. and Yu,K.

TITLE Direct Submission

JOURNAL Submitted (11-MAR-2019) Coral Reef Research Center of China, School

of Marine Sciences, No. 100 Daxue Road, Xixiangtang District,

Nanning, Guangxi Zhuang Autonomous Region 530004, China

COMMENT ##Assembly-Data-START##

Sequencing Technology :: Sanger dideoxy sequencing

##Assembly-Data-END##

FEATURES Location/Qualifiers

source 1..640

/organism="Vibrio sp."

/mol_type="genomic DNA"

/strain="sL26"

/isolation_source="coral"

/db_xref="taxon:678"

rRNA <1..>640

/product="16S ribosomal RNA"

ORIGIN

1 cgagttatct gaaccttcgg ggaacgataa cggcgtcgag cggcggacgg gtgagtaatg

61 cctaggaaat tgccctgatg tgggggataa ccattggaaa cgatggctaa taccgcataa

121 tgcctacggg ccaaagaggg ggaccttcgg gcctctcgcg tcaggatatg cctaggtggg

181 attagctagt tggtgaggta atggctcacc aaggcgacga tccctagctg gtctgagagg

241 atgatcagcc acactggaac tgagacacgg tccagactcc tacgggaggc agcagtgggg

301 aatattgcac aatgggcgca agcctgatgc agccatgccg cgtgtgtgaa gaaggccttc

361 gggttgtaaa gcactttcag tcgtgaggaa ggtggtgtag ttaatagctg cattacttga

421 cgttagcgac agaagaagca ccggctaact ccgtgccagc agccgcggta atacggaggg

481 tgcgagcgtt aatcggaatt actgggcgta aagcgcatgc aggtggtttg ttaagtcaga

541 tgtgaaagcc cggggctcaa cctcggaata gcatttgaaa ctggcagact agagtactgt

601 agaggggggt agaatttcag gtgtagcggt gaaatgcgta

//

LOCUS MK617716 640 bp DNA linear BCT 30-MAY-2019

DEFINITION Microbulbifer sp. strain sL27 16S ribosomal RNA gene, partial

sequence.

ACCESSION MK617716

VERSION MK617716

KEYWORDS .

SOURCE Microbulbifer sp.

ORGANISM Microbulbifer sp.

Bacteria; Proteobacteria; Gammaproteobacteria; Cellvibrionales;

Microbulbiferaceae; Microbulbifer.

REFERENCE 1 (bases 1 to 640)

AUTHORS Su,H., Huang,Q. and Yu,K.

TITLE Diversity of Cultivable Protease-Producing Bacteria

JOURNAL Unpublished

REFERENCE 2 (bases 1 to 640)

AUTHORS Su,H., Huang,Q. and Yu,K.

TITLE Direct Submission

JOURNAL Submitted (11-MAR-2019) Coral Reef Research Center of China, School

of Marine Sciences, No. 100 Daxue Road, Xixiangtang District,

Nanning, Guangxi Zhuang Autonomous Region 530004, China

COMMENT ##Assembly-Data-START##

Sequencing Technology :: Sanger dideoxy sequencing

##Assembly-Data-END##

FEATURES Location/Qualifiers

source 1..640

/organism="Microbulbifer sp."

/mol_type="genomic DNA"

/strain="sL27"

/isolation_source="coral"

/db_xref="taxon:1908541"

rRNA <1..>640

/product="16S ribosomal RNA"

ORIGIN

1 gttccttcgg gaacaagtag agcggcggac gggtgagtaa cgcgtgggaa attgcccagt

61 agtgggggac aacattcgga aacggatgct aataccgcat acgccctacg ggggaaagca

121 ggggatcttc ggaccttgcg ctattggata tgcccgcgtc ggattagcta gttggtgagg

181 taatggctca ccaaggcaac gatccgtagc tggtctgaga ggatgatcag ccacactggg

241 actgagacac ggcccagact cctacgggag gcagcagtgg ggaatattgg acaatgggcg

301 caagcctgat ccagccatgc cgcgtgtgtg aagaaggccc tagggttgta aagcactttc

361 agtagggagg aaggccttaa agttaatacc tttgaggatt gacgttacct acagaagaag

421 caccggctaa ctccgtgcca gcagccgcgg taatacggag ggtgcgagcg ttaatcggaa

481 ttactgggcg taaagcgcgc gtaggcggtt agttaagctg gatgtgaaag ccccgggctc

541 aacctgggaa ctgcattcag aactggctgg ctagagtacg agagagggta gtggaatttc

601 ctgtgtagcg gtgaaatgcg tagatatagg aaggaacatc

//

LOCUS MK617717 640 bp DNA linear BCT 30-MAY-2019

DEFINITION Microbulbifer sp. strain sL30 16S ribosomal RNA gene, partial

sequence.

ACCESSION MK617717

VERSION MK617717

KEYWORDS .

SOURCE Microbulbifer sp.

ORGANISM Microbulbifer sp.

Bacteria; Proteobacteria; Gammaproteobacteria; Cellvibrionales;

Microbulbiferaceae; Microbulbifer.

REFERENCE 1 (bases 1 to 640)

AUTHORS Su,H., Huang,Q. and Yu,K.

TITLE Diversity of Cultivable Protease-Producing Bacteria

JOURNAL Unpublished

REFERENCE 2 (bases 1 to 640)

AUTHORS Su,H., Huang,Q. and Yu,K.

TITLE Direct Submission

JOURNAL Submitted (11-MAR-2019) Coral Reef Research Center of China, School

of Marine Sciences, No. 100 Daxue Road, Xixiangtang District,

Nanning, Guangxi Zhuang Autonomous Region 530004, China

COMMENT ##Assembly-Data-START##

Sequencing Technology :: Sanger dideoxy sequencing

##Assembly-Data-END##

FEATURES Location/Qualifiers

source 1..640

/organism="Microbulbifer sp."

/mol_type="genomic DNA"

/strain="sL30"

/isolation_source="coral"

/db_xref="taxon:1908541"

rRNA <1..>640

/product="16S ribosomal RNA"

ORIGIN

1 aagttcttcg gaacgagtag agcggcggac gggtgagtaa cgcgtgggaa attgcccagt

61 agtgggggac aacattcgga aacggatgct aataccgcat acgccctacg ggggaaagca

121 ggggatcttc ggaccttgtg ctattggata tgcccgcgtc ggattagcta gttggtgagg

181 taatggctca ccaaggcaac gatccgtagc tggtctgaga ggatgatcag ccacactggg

241 actgagacac ggcccagact cctacgggag gcagcagtgg ggaatattgg acaatgggcg

301 caagcctgat ccagccatgc cgcgtgtgtg aagaaggctc tagggttgta aagcactttc

361 agtagggagg aaggccttaa agttaatacc tttgaggatt gacgttacct acagaagaag

421 caccggctaa ctccgtgcca gcagccgcgg taatacggag ggtgcgagcg ttaatcggaa

481 ttactgggcg taaagcgcgc gtaggcggtt agttaagctg gatgtgaaag ccccgggctc

541 aacctgggaa ctgcattcag aactggctgg ctagagtacg agagagggta gtggaatttc

601 ctgtgtagcg gtgaaatgcg tagatatagg aaggaacatc

//

LOCUS MK617718 640 bp DNA linear BCT 30-MAY-2019

DEFINITION Pseudovibrio sp. strain sL34 16S ribosomal RNA gene, partial

sequence.

ACCESSION MK617718

VERSION MK617718

KEYWORDS .

SOURCE Pseudovibrio sp.

ORGANISM Pseudovibrio sp.

Bacteria; Proteobacteria; Alphaproteobacteria; Rhodobacterales;

Rhodobacteraceae; Pseudovibrio.

REFERENCE 1 (bases 1 to 640)

AUTHORS Su,H., Huang,Q. and Yu,K.

TITLE Diversity of Cultivable Protease-Producing Bacteria

JOURNAL Unpublished

REFERENCE 2 (bases 1 to 640)

AUTHORS Su,H., Huang,Q. and Yu,K.

TITLE Direct Submission

JOURNAL Submitted (11-MAR-2019) Coral Reef Research Center of China, School

of Marine Sciences, No. 100 Daxue Road, Xixiangtang District,

Nanning, Guangxi Zhuang Autonomous Region 530004, China

COMMENT ##Assembly-Data-START##

Sequencing Technology :: Sanger dideoxy sequencing

##Assembly-Data-END##

FEATURES Location/Qualifiers

source 1..640

/organism="Pseudovibrio sp."

/mol_type="genomic DNA"

/strain="sL34"

/isolation_source="coral"

/db_xref="taxon:1909297"

rRNA <1..>640

/product="16S ribosomal RNA"

ORIGIN

1 tccttcggga ttagtggcag acgggtgagt aacgcgtggg aagctacctt gtggtaggga

61 acaacagttg gaaacgactg ctaataccct atgagcccta tgggggaaag atttatcgcc

121 atgagatgtg cccgcgttag attagctagt tggtaaggta atggcttacc aaggcgacga

181 tctatagctg gtctgagagg atgatcagcc acactgggac tgagacacgg cccagactcc

241 tacgggaggc agcagtgggg aatattggac aatgggggca accctgatcc agccatgccg

301 cgtgagtgat gacggcctta gggttgtaaa gctctttcag cagtgaagat aatgacatta

361 actgcagaag aagccccggc taacttcgtg ccagcagccg cggtaatacg aagggggcta

421 gcgttgttcg gaatcactgg gcgtaaagcg tacgtaggcg gactgatcag tcaggggtga

481 aatcccgggg ctcaaccccg gaactgcctt tgatactgtc agtcttgaga tcgagagagg

541 tgagtggaac tccgagtgta gaggtgaaat tcgtagatat tcggaagaac accagtggcg

601 aaggcggctc actggctcga tactgacgct gaggtacgaa

//

LOCUS MK617719 640 bp DNA linear BCT 30-MAY-2019

DEFINITION Pseudovibrio sp. strain sL37 16S ribosomal RNA gene, partial

sequence.

ACCESSION MK617719

VERSION MK617719

KEYWORDS .

SOURCE Pseudovibrio sp.

ORGANISM Pseudovibrio sp.

Bacteria; Proteobacteria; Alphaproteobacteria; Rhodobacterales;

Rhodobacteraceae; Pseudovibrio.

REFERENCE 1 (bases 1 to 640)

AUTHORS Su,H., Huang,Q. and Yu,K.

TITLE Diversity of Cultivable Protease-Producing Bacteria

JOURNAL Unpublished

REFERENCE 2 (bases 1 to 640)

AUTHORS Su,H., Huang,Q. and Yu,K.

TITLE Direct Submission

JOURNAL Submitted (11-MAR-2019) Coral Reef Research Center of China, School

of Marine Sciences, No. 100 Daxue Road, Xixiangtang District,

Nanning, Guangxi Zhuang Autonomous Region 530004, China

COMMENT ##Assembly-Data-START##

Sequencing Technology :: Sanger dideoxy sequencing

##Assembly-Data-END##

FEATURES Location/Qualifiers

source 1..640

/organism="Pseudovibrio sp."

/mol_type="genomic DNA"

/strain="sL37"

/isolation_source="coral"

/db_xref="taxon:1909297"

rRNA <1..>640

/product="16S ribosomal RNA"

ORIGIN

1 tccttcggga ttagtggcag acgggtgagt aacgcgtggg aagctacctt gtggtaggga

61 acaacagttg gaaacgactg ctaataccct atgagcccta tgggggaaag atttatcgcc

121 atgagatgtg cccgcgttag attagctagt tggtaaggta atggcttacc aaggcgacga

181 tctatagctg gtctgagagg atgatcagcc acactgggac tgagacacgg cccagactcc

241 tacgggaggc agcagtgggg aatattggac aatgggggca accctgatcc agccatgccg

301 cgtgagtgat gacggcctta gggttgtaaa gctctttcag cagtgaagat aatgacatta

361 actgcagaag aagccccggc taacttcgtg ccagcagccg cggtaatacg aagggggcta

421 gcgttgttcg gaatcactgg gcgtaaagcg tacgtaggcg gactgatcag tcaggggtga

481 aatcccgggg ctcaaccccg gaactgcctt tgatactgtc agtcttgaga tcgagagagg

541 tgagtggaac tccgagtgta gaggtgaaat tcgtagatat tcggaagaac accagtggcg

601 aaggcggctc actggctcga tactgacgct gaggtacgaa

//

LOCUS MK617720 640 bp DNA linear BCT 30-MAY-2019

DEFINITION Pseudovibrio sp. strain sL47 16S ribosomal RNA gene, partial

sequence.

ACCESSION MK617720

VERSION MK617720

KEYWORDS .

SOURCE Pseudovibrio sp.

ORGANISM Pseudovibrio sp.

Bacteria; Proteobacteria; Alphaproteobacteria; Rhodobacterales;

Rhodobacteraceae; Pseudovibrio.

REFERENCE 1 (bases 1 to 640)

AUTHORS Su,H., Huang,Q. and Yu,K.

TITLE Diversity of Cultivable Protease-Producing Bacteria

JOURNAL Unpublished

REFERENCE 2 (bases 1 to 640)

AUTHORS Su,H., Huang,Q. and Yu,K.

TITLE Direct Submission

JOURNAL Submitted (11-MAR-2019) Coral Reef Research Center of China, School

of Marine Sciences, No. 100 Daxue Road, Xixiangtang District,

Nanning, Guangxi Zhuang Autonomous Region 530004, China

COMMENT ##Assembly-Data-START##

Sequencing Technology :: Sanger dideoxy sequencing

##Assembly-Data-END##

FEATURES Location/Qualifiers

source 1..640

/organism="Pseudovibrio sp."

/mol_type="genomic DNA"

/strain="sL47"

/isolation_source="coral"

/db_xref="taxon:1909297"

rRNA <1..>640

/product="16S ribosomal RNA"

ORIGIN

1 ggatccttcg ggattagtgg cagacgggtg agtaacgcgt gggaagctac cttgtggtag

61 ggaacaacag ttggaaacga ctgctaatac cctatgagcc ctatggggga aagatttatc

121 gccatgagat gtgcccgcgt tagattagct agttggtaag gtaatggctt accaaggcga

181 cgatctatag ctggtctgag aggatgatca gccacactgg gactgagaca cggcccagac

241 tcctacggga ggcagcagtg gggaatattg gacaatgggg gcaaccctga tccagccatg

301 ccgcgtgagt gatgacggcc ttagggttgt aaagctcttt cagcagtgaa gataatgaca

361 ttaactgcag aagaagcccc ggctaacttc gtgccagcag ccgcggtaat acgaaggggg

421 ctagcgttgt tcggaatcac tgggcgtaaa gcgtacgtag gcggactgat cagtcagggg

481 tgaaatcccg gggctcaacc ccggaactgc ctttgatact gtcagtcttg agatcgagag

541 aggtgagtgg aactccgagt gtagaggtga aattcgtaga tattcggaag aacaccagtg

601 gcgaaggcgg ctcactggct cgatactgac gctgaggtac

//

LOCUS MK617721 640 bp DNA linear BCT 30-MAY-2019

DEFINITION Fictibacillus sp. strain sL56 16S ribosomal RNA gene, partial

sequence.

ACCESSION MK617721

VERSION MK617721

KEYWORDS .

SOURCE Fictibacillus sp.

ORGANISM Fictibacillus sp.

Bacteria; Firmicutes; Bacilli; Bacillales; Bacillaceae;

Fictibacillus.

REFERENCE 1 (bases 1 to 640)

AUTHORS Su,H., Huang,Q. and Yu,K.

TITLE Diversity of Cultivable Protease-Producing Bacteria

JOURNAL Unpublished

REFERENCE 2 (bases 1 to 640)

AUTHORS Su,H., Huang,Q. and Yu,K.

TITLE Direct Submission

JOURNAL Submitted (11-MAR-2019) Coral Reef Research Center of China, School

of Marine Sciences, No. 100 Daxue Road, Xixiangtang District,

Nanning, Guangxi Zhuang Autonomous Region 530004, China

COMMENT ##Assembly-Data-START##

Sequencing Technology :: Sanger dideoxy sequencing

##Assembly-Data-END##

FEATURES Location/Qualifiers

source 1..640

/organism="Fictibacillus sp."

/mol_type="genomic DNA"

/strain="sL56"

/isolation_source="coral"

/db_xref="taxon:1871617"

rRNA <1..>640

/product="16S ribosomal RNA"

ORIGIN

1 aagaggagct tgctcctctg atttagcggc ggacgggtga gtaacacgtg ggtaatctgc

61 ctgtaagacg gggataactc cgggaaaccg gggctaatac cggataataa gagaagaagc

121 atttcttctt tttgaaagtc ggtttcggct gacacttaca gatgagcccg cggcgcatta

181 gctagttggt gaggtaacgg ctcaccaagg cgacgatgcg tagccgacct gagagggtga

241 tcggccacac tgggactgag acacggccca gactcctacg ggaggcagca gtagggaatc

301 ttcggcaatg ggcgaaagcc tgaccgagca acgccgcgtg agcgatgaag gccttcgggt

361 cgtaaagctc tgttgttaga gaagaacaag tacgagagta actgctcgta ccttgacggt

421 acctaaccag aaagccacgg ctaactacgt gccagcagcc gcggtaatac gtaggtggca

481 agcgttatcc ggaattattg ggcgtaaagc gcgcgcaggc ggtctcttaa gtctgatgtg

541 aaagcccacg gctcaaccgt ggagggtcat tggaaactgg gagacttgag tgcaggagag

601 aaaagtggaa ttccacgtgt agcggtgaaa tgcgtagaga

//

LOCUS MK617722 640 bp DNA linear BCT 30-MAY-2019

DEFINITION Vibrio sp. strain sQ3 16S ribosomal RNA gene, partial sequence.

ACCESSION MK617722

VERSION MK617722

KEYWORDS .

SOURCE Vibrio sp.

ORGANISM Vibrio sp.

Bacteria; Proteobacteria; Gammaproteobacteria; Vibrionales;

Vibrionaceae; Vibrio.

REFERENCE 1 (bases 1 to 640)

AUTHORS Su,H., Huang,Q. and Yu,K.

TITLE Diversity of Cultivable Protease-Producing Bacteria

JOURNAL Unpublished

REFERENCE 2 (bases 1 to 640)

AUTHORS Su,H., Huang,Q. and Yu,K.

TITLE Direct Submission

JOURNAL Submitted (11-MAR-2019) Coral Reef Research Center of China, School

of Marine Sciences, No. 100 Daxue Road, Xixiangtang District,

Nanning, Guangxi Zhuang Autonomous Region 530004, China

COMMENT ##Assembly-Data-START##

Sequencing Technology :: Sanger dideoxy sequencing

##Assembly-Data-END##

FEATURES Location/Qualifiers

source 1..640

/organism="Vibrio sp."

/mol_type="genomic DNA"

/strain="sQ3"

/isolation_source="coral"

/db_xref="taxon:678"

rRNA <1..>640

/product="16S ribosomal RNA"

ORIGIN

1 ttatctgaac cttcggggaa cgataacggc gtcgagcggc ggacgggtga gtaatgccta

61 ggaaattgcc ctgatgtggg ggataaccat tggaaacgat ggctaatacc gcatgatgcc

121 tacgggccaa agagggggac cttcgggcct ctcgcgtcag gatatgccta ggtgggatta

181 gctagttggt gaggtaaggg ctcaccaagg cgacgatccc tagctggtct gagaggatga

241 tcagccacac tggaactgag acacggtcca gactcctacg ggaggcagca gtggggaata

301 ttgcacaatg ggcgcaagcc tgatgcagcc atgccgcgtg tgtgaagaag gccttcgggt

361 tgtaaagcac tttcagtcgt gaggaaggtg gtgtagttaa tagctgcatt atttgacgtt

421 agcgacagaa gaagcaccgg ctaactccgt gccagcagcc gcggtaatac ggagggtgcg

481 agcgttaatc ggaattactg ggcgtaaagc gcatgcaggt ggtttgttaa gtcagatgtg

541 aaagcccggg gctcaacctc ggaatagcat ttgaaactgg cagactagag tactgtagag

601 gggggtagaa tttcaggtgt agcggtgaaa tgcgtagaga

//

LOCUS MK617723 640 bp DNA linear BCT 30-MAY-2019

DEFINITION Vibrio sp. strain sQ5 16S ribosomal RNA gene, partial sequence.

ACCESSION MK617723

VERSION MK617723

KEYWORDS .

SOURCE Vibrio sp.

ORGANISM Vibrio sp.

Bacteria; Proteobacteria; Gammaproteobacteria; Vibrionales;

Vibrionaceae; Vibrio.

REFERENCE 1 (bases 1 to 640)

AUTHORS Su,H., Huang,Q. and Yu,K.

TITLE Diversity of Cultivable Protease-Producing Bacteria

JOURNAL Unpublished

REFERENCE 2 (bases 1 to 640)

AUTHORS Su,H., Huang,Q. and Yu,K.

TITLE Direct Submission

JOURNAL Submitted (11-MAR-2019) Coral Reef Research Center of China, School

of Marine Sciences, No. 100 Daxue Road, Xixiangtang District,

Nanning, Guangxi Zhuang Autonomous Region 530004, China

COMMENT ##Assembly-Data-START##

Sequencing Technology :: Sanger dideoxy sequencing

##Assembly-Data-END##

FEATURES Location/Qualifiers

source 1..640

/organism="Vibrio sp."

/mol_type="genomic DNA"

/strain="sQ5"

/isolation_source="coral"

/db_xref="taxon:678"

rRNA <1..>640

/product="16S ribosomal RNA"

ORIGIN

1 ttatctgaac cttcggggaa cgataacggc gtcgagcggc ggacgggtga gtaatgccta

61 ggaaattgcc ctgatgtggg ggataaccat tggaaacgat ggctaatacc gcatgatgcc

121 tacgggccaa agagggggac cttcgggcct ctcgcgtcag gatatgccta ggtgggatta

181 gctagttggt gaggtaaggg ctcaccaagg cgacgatccc tagctggtct gagaggatga

241 tcagccacac tggaactgag acacggtcca gactcctacg ggaggcagca gtggggaata

301 ttgcacaatg ggcgcaagcc tgatgcagcc atgccgcgtg tgtgaagaag gccttcgggt

361 tgtaaagcac tttcagtcgt gaggaaggtg gtgtagttaa tagctgcatt atttgacgtt

421 agcgacagaa gaagcaccgg ctaactccgt gccagcagcc gcggtaatac ggagggtgcg

481 agcgttaatc ggaattactg ggcgtaaagc gcatgcaggt ggtttgttaa gtcagatgtg

541 aaagcccggg gctcaacctc ggaatagcat ttgaaactgg cagactagag tactgtagag

601 gggggtagaa tttcaggtgt agcggtgaaa tgcgtagaga

//

LOCUS MK617724 640 bp DNA linear BCT 30-MAY-2019

DEFINITION Vibrio sp. strain sQ11 16S ribosomal RNA gene, partial sequence.

ACCESSION MK617724

VERSION MK617724

KEYWORDS .

SOURCE Vibrio sp.

ORGANISM Vibrio sp.

Bacteria; Proteobacteria; Gammaproteobacteria; Vibrionales;

Vibrionaceae; Vibrio.

REFERENCE 1 (bases 1 to 640)

AUTHORS Su,H., Huang,Q. and Yu,K.

TITLE Diversity of Cultivable Protease-Producing Bacteria

JOURNAL Unpublished

REFERENCE 2 (bases 1 to 640)

AUTHORS Su,H., Huang,Q. and Yu,K.

TITLE Direct Submission

JOURNAL Submitted (11-MAR-2019) Coral Reef Research Center of China, School

of Marine Sciences, No. 100 Daxue Road, Xixiangtang District,

Nanning, Guangxi Zhuang Autonomous Region 530004, China

COMMENT ##Assembly-Data-START##

Sequencing Technology :: Sanger dideoxy sequencing

##Assembly-Data-END##

FEATURES Location/Qualifiers

source 1..640

/organism="Vibrio sp."

/mol_type="genomic DNA"

/strain="sQ11"

/isolation_source="coral"

/db_xref="taxon:678"

rRNA <1..>640

/product="16S ribosomal RNA"

ORIGIN

1 agttatctga accttcgggg aacgataacg gcgtcgagcg gcggacgggt gagtaatgcc

61 taggaaattg ccctgatgtg ggggataacc attggaaacg atggctaata ccgcatgatg

121 cctacgggcc aaagaggggg accttcgggc ctctcgcgtc aggatatgcc taggtgggat

181 tagctagttg gtgaggtaag ggctcaccaa ggcgacgatc cctagctggt ctgagaggat

241 gatcagccac actggaactg agacacggtc cagactccta cgggaggcag cagtggggaa

301 tattgcacaa tgggcgcaag cctgatgcag ccatgccgcg tgtgtgaaga aggccttcgg

361 gttgtaaagc actttcagtc gtgaggaagg tggtgtagtt aatagctgca ttatttgacg

421 ttagcgacag aagaagcacc ggctaactcc gtgccagcag ccgcggtaat acggagggtg

481 cgagcgttaa tcggaattac tgggcgtaaa gcgcatgcag gtggtttgtt aagtcagatg

541 tgaaagcccg gggctcaacc tcggaatagc atttgaaact ggcagactag agtactgtag

601 aggggggtag aatttcaggt gtagcggtga aatgcgtaga

//

LOCUS MK617725 640 bp DNA linear BCT 30-MAY-2019

DEFINITION Vibrio sp. strain sQ14 16S ribosomal RNA gene, partial sequence.

ACCESSION MK617725

VERSION MK617725

KEYWORDS .

SOURCE Vibrio sp.

ORGANISM Vibrio sp.

Bacteria; Proteobacteria; Gammaproteobacteria; Vibrionales;

Vibrionaceae; Vibrio.

REFERENCE 1 (bases 1 to 640)

AUTHORS Su,H., Huang,Q. and Yu,K.

TITLE Diversity of Cultivable Protease-Producing Bacteria

JOURNAL Unpublished

REFERENCE 2 (bases 1 to 640)

AUTHORS Su,H., Huang,Q. and Yu,K.

TITLE Direct Submission

JOURNAL Submitted (11-MAR-2019) Coral Reef Research Center of China, School

of Marine Sciences, No. 100 Daxue Road, Xixiangtang District,

Nanning, Guangxi Zhuang Autonomous Region 530004, China

COMMENT ##Assembly-Data-START##

Sequencing Technology :: Sanger dideoxy sequencing

##Assembly-Data-END##

FEATURES Location/Qualifiers

source 1..640

/organism="Vibrio sp."

/mol_type="genomic DNA"

/strain="sQ14"

/isolation_source="coral"

/db_xref="taxon:678"

rRNA <1..>640

/product="16S ribosomal RNA"

ORIGIN

1 agttatctga accttcgggg aacgataacg gcgtcgagcg gcggacgggt gagtaatgcc

61 taggaaattg ccctgatgtg ggggataacc attggaaacg atggctaata ccgcatgatg

121 cctacgggcc aaagaggggg accttcgggc ctctcgcgtc aggatatgcc taggtgggat

181 tagctagttg gtgaggtaag ggctcaccaa ggcgacgatc cctagctggt ctgagaggat

241 gatcagccac actggaactg agacacggtc cagactccta cgggaggcag cagtggggaa

301 tattgcacaa tgggcgcaag cctgatgcag ccatgccgcg tgtgtgaaga aggccttcgg

361 gttgtaaagc actttcagtc gtgaggaagg tggtgtagtt aatagctgca tcatttgacg

421 ttagcgacag aagaagcacc ggctaactcc gtgccagcag ccgcggtaat acggagggtg

481 cgagcgttaa tcggaattac tgggcgtaaa gcgcatgcag gtggtttgtt aagtcagatg

541 tgaaagcccg gggctcaacc tcggaatagc atttgaaact ggcagactag agtactgtag

601 aggggggtag aatttcaggt gtagcggtga aatgcgtaga

//

LOCUS MK617726 640 bp DNA linear BCT 30-MAY-2019

DEFINITION Bacillus sp. (in: Bacteria) strain sQ21 16S ribosomal RNA gene,

partial sequence.

ACCESSION MK617726

VERSION MK617726

KEYWORDS .

SOURCE Bacillus sp. (in: Bacteria)

ORGANISM Bacillus sp. (in: Bacteria)

Bacteria; Firmicutes; Bacilli; Bacillales; Bacillaceae; Bacillus.

REFERENCE 1 (bases 1 to 640)

AUTHORS Su,H., Huang,Q. and Yu,K.

TITLE Diversity of Cultivable Protease-Producing Bacteria

JOURNAL Unpublished

REFERENCE 2 (bases 1 to 640)

AUTHORS Su,H., Huang,Q. and Yu,K.

TITLE Direct Submission

JOURNAL Submitted (11-MAR-2019) Coral Reef Research Center of China, School

of Marine Sciences, No. 100 Daxue Road, Xixiangtang District,

Nanning, Guangxi Zhuang Autonomous Region 530004, China

COMMENT ##Assembly-Data-START##

Sequencing Technology :: Sanger dideoxy sequencing

##Assembly-Data-END##

FEATURES Location/Qualifiers

source 1..640

/organism="Bacillus sp. (in: Bacteria)"

/mol_type="genomic DNA"

/strain="sQ21"

/isolation_source="coral"

/db_xref="taxon:1409"

rRNA <1..>640

/product="16S ribosomal RNA"

ORIGIN

1 gatgggagct tgctccctga agtcagcggc ggacgggtga gtaacacgtg ggcaacctgc

61 ctgtaagact gggataactc cgggaaaccg gggctaatac cggataattc tttccctcac

121 atgagggaaa gctgaaagat ggtttcggct atcacttaca gatgggcccg cggcgcatta

181 gctagttggt gaggtaacgg ctcaccaagg caacgatgcg tagccgacct gagagggtga

241 tcggccacac tgggactgag acacggccca gactcctacg ggaggcagca gtagggaatc

301 ttccgcaatg gacgaaagtc tgacggagca acgccgcgtg agtgatgaag gttttcggat

361 cgtaaaactc tgttgttagg gaagaacaag taccggagta actgccggta ccttgacggt

421 acctaaccag aaagccacgg ctaactacgt gccagcagcc gcggtaatac gtaggtggca

481 agcgttgtcc ggaattattg ggcgtaaagc gcgcgcaggc ggttccttaa gtctgatgtg

541 aaagcccccg gctcaaccgg ggagggtcat tggaaactgg ggaacttgag tgcagaagag

601 aagagtggaa ttccacgtgt agcggtgaaa tgcgtagaga

//

LOCUS MK617727 640 bp DNA linear BCT 30-MAY-2019

DEFINITION Vibrio sp. strain sQ22 16S ribosomal RNA gene, partial sequence.

ACCESSION MK617727

VERSION MK617727

KEYWORDS .

SOURCE Vibrio sp.

ORGANISM Vibrio sp.

Bacteria; Proteobacteria; Gammaproteobacteria; Vibrionales;

Vibrionaceae; Vibrio.

REFERENCE 1 (bases 1 to 640)

AUTHORS Su,H., Huang,Q. and Yu,K.

TITLE Diversity of Cultivable Protease-Producing Bacteria

JOURNAL Unpublished

REFERENCE 2 (bases 1 to 640)

AUTHORS Su,H., Huang,Q. and Yu,K.

TITLE Direct Submission

JOURNAL Submitted (11-MAR-2019) Coral Reef Research Center of China, School

of Marine Sciences, No. 100 Daxue Road, Xixiangtang District,

Nanning, Guangxi Zhuang Autonomous Region 530004, China

COMMENT ##Assembly-Data-START##

Sequencing Technology :: Sanger dideoxy sequencing

##Assembly-Data-END##

FEATURES Location/Qualifiers

source 1..640

/organism="Vibrio sp."

/mol_type="genomic DNA"

/strain="sQ22"

/isolation_source="coral"

/db_xref="taxon:678"

rRNA <1..>640

/product="16S ribosomal RNA"

ORIGIN

1 agttatctga accttcgggg aacgataacg gcgtcgagcg gcggacgggt gagtaatgcc

61 taggaaattg ccctgatgtg ggggataacc attggaaacg atggctaata ccgcatgatg

121 cctacgggcc aaagaggggg accttcgggc ctctcgcgtc aggatatgcc taggtgggat

181 tagctagttg gtgaggtaag ggctcaccaa ggcgacgatc cctagctggt ctgagaggat

241 gatcagccac actggaactg agacacggtc cagactccta cgggaggcag cagtggggaa

301 tattgcacaa tgggcgcaag cctgatgcag ccatgccgcg tgtgtgaaga aggccttcgg

361 gttgtaaagc actttcagtc gtgaggaagg tggtgtagtt aatagctgca ttatttgacg

421 ttagcgacag aagaagcacc ggctaactcc gtgccagcag ccgcggtaat acggagggtg

481 cgagcgttaa tcggaattac tgggcgtaaa gcgcatgcag gtggtttgtt aagtcagatg

541 tgaaagcccg gggctcaacc tcggaatagc atttgaaact ggcagactag agtactgtag

601 aggggggtag aatttcaggt gtagcggtga aatgcgtaga

//

LOCUS MK617728 640 bp DNA linear BCT 30-MAY-2019

DEFINITION Vibrio sp. strain sQ23 16S ribosomal RNA gene, partial sequence.

ACCESSION MK617728

VERSION MK617728

KEYWORDS .

SOURCE Vibrio sp.

ORGANISM Vibrio sp.

Bacteria; Proteobacteria; Gammaproteobacteria; Vibrionales;

Vibrionaceae; Vibrio.

REFERENCE 1 (bases 1 to 640)

AUTHORS Su,H., Huang,Q. and Yu,K.

TITLE Diversity of Cultivable Protease-Producing Bacteria

JOURNAL Unpublished

REFERENCE 2 (bases 1 to 640)

AUTHORS Su,H., Huang,Q. and Yu,K.

TITLE Direct Submission

JOURNAL Submitted (11-MAR-2019) Coral Reef Research Center of China, School

of Marine Sciences, No. 100 Daxue Road, Xixiangtang District,

Nanning, Guangxi Zhuang Autonomous Region 530004, China

COMMENT ##Assembly-Data-START##

Sequencing Technology :: Sanger dideoxy sequencing

##Assembly-Data-END##

FEATURES Location/Qualifiers

source 1..640

/organism="Vibrio sp."

/mol_type="genomic DNA"

/strain="sQ23"

/isolation_source="coral"

/db_xref="taxon:678"

rRNA <1..>640

/product="16S ribosomal RNA"

ORIGIN

1 gagttatctg aaccttcggg gaacgataac ggcgtcgagc ggcggacggg tgagtaatgc

61 ctaggaaatt gccctgatgt gggggataac cattggaaac gatggctaat accgcatgat

121 gcctacgggc caaagagggg gaccttcggg cctctcgcgt caggatatgc ctaggtggga

181 ttagctagtt ggtgaggtaa gggctcacca aggcgacgat ccctagctgg tctgagagga

241 tgatcagcca cactggaact gagacacggt ccagactcct acgggaggca gcagtgggga

301 atattgcaca atgggcgcaa gcctgatgca gccatgccgc gtgtgtgaag aaggccttcg

361 ggttgtaaag cactttcagt cgtgaggaag gtggtgtagt taatagctgc attatttgac

421 gttagcgaca gaagaagcac cggctaactc cgtgccagca gccgcggtaa tacggagggt

481 gcgagcgtta atcggaatta ctgggcgtaa agcgcatgca ggtggtttgt taagtcagat

541 gtgaaagccc ggggctcaac ctcggaatag catttgaaac tggcagacta gagtactgta

601 gaggggggta gaatttcagg tgtagcggtg aaatgcgtag

//

LOCUS MK617729 640 bp DNA linear BCT 30-MAY-2019

DEFINITION Vibrio sp. strain sQ28 16S ribosomal RNA gene, partial sequence.

ACCESSION MK617729

VERSION MK617729

KEYWORDS .

SOURCE Vibrio sp.

ORGANISM Vibrio sp.

Bacteria; Proteobacteria; Gammaproteobacteria; Vibrionales;

Vibrionaceae; Vibrio.

REFERENCE 1 (bases 1 to 640)

AUTHORS Su,H., Huang,Q. and Yu,K.

TITLE Diversity of Cultivable Protease-Producing Bacteria

JOURNAL Unpublished

REFERENCE 2 (bases 1 to 640)

AUTHORS Su,H., Huang,Q. and Yu,K.

TITLE Direct Submission

JOURNAL Submitted (11-MAR-2019) Coral Reef Research Center of China, School

of Marine Sciences, No. 100 Daxue Road, Xixiangtang District,

Nanning, Guangxi Zhuang Autonomous Region 530004, China

COMMENT ##Assembly-Data-START##

Sequencing Technology :: Sanger dideoxy sequencing

##Assembly-Data-END##

FEATURES Location/Qualifiers

source 1..640

/organism="Vibrio sp."

/mol_type="genomic DNA"

/strain="sQ28"

/isolation_source="coral"

/db_xref="taxon:678"

rRNA <1..>640

/product="16S ribosomal RNA"

ORIGIN

1 gagttatctg aaccttcggg gaacgataac ggcgtcgagc ggcggacggg tgagtaatgc

61 ctaggaaatt gccctgatgt gggggataac cattggaaac gatggctaat accgcatgat

121 gcctacgggc caaagagggg gaccttcggg cctctcgcgt caggatatgc ctaggtggga

181 ttagctagtt ggtgaggtaa gggctcacca aggcgacgat ccctagctgg tctgagagga

241 tgatcagcca cactggaact gagacacggt ccagactcct acgggaggca gcagtgggga

301 atattgcaca atgggcgcaa gcctgatgca gccatgccgc gtgtgtgaag aaggccttcg

361 ggttgtaaag cactttcagt cgtgaggaag gtggtgtagt taatagctgc attatttgac

421 gttagcgaca gaagaagcac cggctaactc cgtgccagca gccgcggtaa tacggagggt

481 gcgagcgtta atcggaatta ctgggcgtaa agcgcatgca ggtggtttgt taagtcagat

541 gtgaaagccc ggggctcaac ctcggaatag catttgaaac tggcagacta gagtactgta

601 gaggggggta gaatttcagg tgtagcggtg aaatgcgtag

//

LOCUS MK617730 640 bp DNA linear BCT 30-MAY-2019

DEFINITION Vibrio sp. strain sQ34 16S ribosomal RNA gene, partial sequence.

ACCESSION MK617730

VERSION MK617730

KEYWORDS .

SOURCE Vibrio sp.

ORGANISM Vibrio sp.

Bacteria; Proteobacteria; Gammaproteobacteria; Vibrionales;

Vibrionaceae; Vibrio.

REFERENCE 1 (bases 1 to 640)

AUTHORS Su,H., Huang,Q. and Yu,K.

TITLE Diversity of Cultivable Protease-Producing Bacteria

JOURNAL Unpublished

REFERENCE 2 (bases 1 to 640)

AUTHORS Su,H., Huang,Q. and Yu,K.

TITLE Direct Submission

JOURNAL Submitted (11-MAR-2019) Coral Reef Research Center of China, School

of Marine Sciences, No. 100 Daxue Road, Xixiangtang District,

Nanning, Guangxi Zhuang Autonomous Region 530004, China

COMMENT ##Assembly-Data-START##

Sequencing Technology :: Sanger dideoxy sequencing

##Assembly-Data-END##

FEATURES Location/Qualifiers

source 1..640

/organism="Vibrio sp."

/mol_type="genomic DNA"

/strain="sQ34"

/isolation_source="coral"

/db_xref="taxon:678"

rRNA <1..>640

/product="16S ribosomal RNA"

ORIGIN

1 cgagttatct gaaccttcgg ggaacgataa cggcgtcgag cggcggacgg gtgagtaatg

61 cctaggaaat tgccctgatg tgggggataa ccattggaaa cgatggctaa taccgcatga

121 tgcctacggg ccaaagaggg ggaccttcgg gcctctcgcg tcaggatatg cctaggtggg

181 attagctagt tggtgaggta agggctcacc aaggcgacga tccctagctg gtctgagagg

241 atgatcagcc acactggaac tgagacacgg tccagactcc tacgggaggc agcagtgggg

301 aatattgcac aatgggcgca agcctgatgc agccatgccg cgtgtgtgaa gaaggccttc

361 gggttgtaaa gcactttcag tcgtgaggaa ggtggtgtag ttaatagctg cattatttga

421 cgttagcgac agaagaagca ccggctaact ccgtgccagc agccgcggta atacggaggg

481 tgcgagcgtt aatcggaatt actgggcgta aagcgcatgc aggtggtttg ttaagtcaga

541 tgtgaaagcc cggggctcaa cctcggaata gcatttgaaa ctggcagact agagtactgt

601 agaggggggt agaatttcag gtgtagcggt gaaatgcgta

//

LOCUS MK617731 640 bp DNA linear BCT 30-MAY-2019

DEFINITION Vibrio sp. strain sQ35 16S ribosomal RNA gene, partial sequence.

ACCESSION MK617731

VERSION MK617731

KEYWORDS .

SOURCE Vibrio sp.

ORGANISM Vibrio sp.

Bacteria; Proteobacteria; Gammaproteobacteria; Vibrionales;

Vibrionaceae; Vibrio.

REFERENCE 1 (bases 1 to 640)

AUTHORS Su,H., Huang,Q. and Yu,K.

TITLE Diversity of Cultivable Protease-Producing Bacteria

JOURNAL Unpublished

REFERENCE 2 (bases 1 to 640)

AUTHORS Su,H., Huang,Q. and Yu,K.

TITLE Direct Submission

JOURNAL Submitted (11-MAR-2019) Coral Reef Research Center of China, School

of Marine Sciences, No. 100 Daxue Road, Xixiangtang District,

Nanning, Guangxi Zhuang Autonomous Region 530004, China

COMMENT ##Assembly-Data-START##

Sequencing Technology :: Sanger dideoxy sequencing

##Assembly-Data-END##

FEATURES Location/Qualifiers

source 1..640

/organism="Vibrio sp."

/mol_type="genomic DNA"

/strain="sQ35"

/isolation_source="coral"

/db_xref="taxon:678"

rRNA <1..>640

/product="16S ribosomal RNA"

ORIGIN

1 gagttatctg aaccttcggg gaacgataac ggcgtcgagc ggcggacggg tgagtaatgc

61 ctaggaaatt gccctgatgt gggggataac cattggaaac gatggctaat accgcatgat

121 gcctacgggc caaagagggg gaccttcggg cctctcgcgt caggatatgc ctaggtggga

181 ttagctagtt ggtgaggtaa gggctcacca aggcgacgat ccctagctgg tctgagagga

241 tgatcagcca cactggaact gagacacggt ccagactcct acgggaggca gcagtgggga

301 atattgcaca atgggcgcaa gcctgatgca gccatgccgc gtgtgtgaag aaggccttcg

361 ggttgtaaag cactttcagt cgtgaggaag gtggtgtagt taatagctgc attatttgac

421 gttagcgaca gaagaagcac cggctaactc cgtgccagca gccgcggtaa tacggagggt

481 gcgagcgtta atcggaatta ctgggcgtaa agcgcatgca ggtggtttgt taagtcagat

541 gtgaaagccc ggggctcaac ctcggaatag catttgaaac tggcagacta gagtactgta

601 gaggggggta gaatttcagg tgtagcggtg aaatgcgtag

//

LOCUS MK617732 640 bp DNA linear BCT 30-MAY-2019

DEFINITION Vibrio sp. strain sQ39 16S ribosomal RNA gene, partial sequence.

ACCESSION MK617732

VERSION MK617732

KEYWORDS .

SOURCE Vibrio sp.

ORGANISM Vibrio sp.

Bacteria; Proteobacteria; Gammaproteobacteria; Vibrionales;

Vibrionaceae; Vibrio.

REFERENCE 1 (bases 1 to 640)

AUTHORS Su,H., Huang,Q. and Yu,K.

TITLE Diversity of Cultivable Protease-Producing Bacteria

JOURNAL Unpublished

REFERENCE 2 (bases 1 to 640)

AUTHORS Su,H., Huang,Q. and Yu,K.

TITLE Direct Submission

JOURNAL Submitted (11-MAR-2019) Coral Reef Research Center of China, School

of Marine Sciences, No. 100 Daxue Road, Xixiangtang District,

Nanning, Guangxi Zhuang Autonomous Region 530004, China

COMMENT ##Assembly-Data-START##

Sequencing Technology :: Sanger dideoxy sequencing

##Assembly-Data-END##

FEATURES Location/Qualifiers

source 1..640

/organism="Vibrio sp."

/mol_type="genomic DNA"

/strain="sQ39"

/isolation_source="coral"

/db_xref="taxon:678"

rRNA <1..>640

/product="16S ribosomal RNA"

ORIGIN

1 cgagttatct gaaccttcgg ggaacgataa cggcgtcgag cggcggacgg gtgagtaatg

61 cctaggaaat tgccctgatg tgggggataa ccattggaaa cgatggctaa taccgcatga

121 tgcctacggg ccaaagaggg ggaccttcgg gcctctcgcg tcaggatatg cctaggtggg

181 attagctagt tggtgaggta agggctcacc aaggcgacga tccctagctg gtctgagagg

241 atgatcagcc acactggaac tgagacacgg tccagactcc tacgggaggc agcagtgggg

301 aatattgcac aatgggcgca agcctgatgc agccatgccg cgtgtgtgaa gaaggccttc

361 gggttgtaaa gcactttcag tcgtgaggaa ggtggtgtag ttaatagctg cattatttga

421 cgttagcgac agaagaagca ccggctaact ccgtgccagc agccgcggta atacggaggg

481 tgcgagcgtt aatcggaatt actgggcgta aagcgcatgc aggtggtttg ttaagtcaga

541 tgtgaaagcc cggggctcaa cctcggaata gcatttgaaa ctggcagact agagtactgt

601 agaggggggt agaatttcag gtgtagcggt gaaatgcgta

//

LOCUS MK617733 640 bp DNA linear BCT 30-MAY-2019

DEFINITION Vibrio sp. strain sQ48 16S ribosomal RNA gene, partial sequence.

ACCESSION MK617733

VERSION MK617733

KEYWORDS .

SOURCE Vibrio sp.

ORGANISM Vibrio sp.

Bacteria; Proteobacteria; Gammaproteobacteria; Vibrionales;

Vibrionaceae; Vibrio.

REFERENCE 1 (bases 1 to 640)

AUTHORS Su,H., Huang,Q. and Yu,K.

TITLE Diversity of Cultivable Protease-Producing Bacteria

JOURNAL Unpublished

REFERENCE 2 (bases 1 to 640)

AUTHORS Su,H., Huang,Q. and Yu,K.

TITLE Direct Submission

JOURNAL Submitted (11-MAR-2019) Coral Reef Research Center of China, School

of Marine Sciences, No. 100 Daxue Road, Xixiangtang District,

Nanning, Guangxi Zhuang Autonomous Region 530004, China

COMMENT ##Assembly-Data-START##

Sequencing Technology :: Sanger dideoxy sequencing

##Assembly-Data-END##

FEATURES Location/Qualifiers

source 1..640

/organism="Vibrio sp."

/mol_type="genomic DNA"

/strain="sQ48"

/isolation_source="coral"

/db_xref="taxon:678"

rRNA <1..>640

/product="16S ribosomal RNA"

ORIGIN

1 agttgtctga tgcttctttg aacgataacg gcgtccagcg gaggacgggt gagtaatgcc

61 tactaaattg ccctgctgcg ggggataacc attggtaacg atggctaata ccgcatgagg

121 cctgggggcc acagaggggg accttcgggc ctctcgcgtc gggatatgcc taggggggat

181 tagctggttg gtgaggtaat ggctccccac ggggacgatc cctagctggt ctgagaggat

241 gatcagacac agtggaactg acacacggtc cagactccgg cgggaggcgg cagtggggaa

301 tattgcagaa tgggcgctag cctgatgcag acatgccgcg tgtgtgaaga aggccttcgg

361 gttgtaaagc actttcagtc gtgaggaagg tggtgtatgt aatagttgcg ttatttgaag

421 ttagcgacaa aacaagcacc ggctacctcc gtgccagcag ccgcggaaat acggagggag

481 ctagcgttaa tcggaattac tgggcgtaaa gcgcatgcag gtggtttgtt cagtcggatg

541 tgaaagcccg gggcactacc tcggaattga atttaaaact ggaagactag agtaatgtac

601 aggggggtag aatttcaggt gtaacatctg aaatgcatat

//

LOCUS MK617734 640 bp DNA linear BCT 30-MAY-2019

DEFINITION Bacillus sp. (in: Bacteria) strain sQ50 16S ribosomal RNA gene,

partial sequence.

ACCESSION MK617734

VERSION MK617734

KEYWORDS .

SOURCE Bacillus sp. (in: Bacteria)

ORGANISM Bacillus sp. (in: Bacteria)

Bacteria; Firmicutes; Bacilli; Bacillales; Bacillaceae; Bacillus.

REFERENCE 1 (bases 1 to 640)

AUTHORS Su,H., Huang,Q. and Yu,K.

TITLE Diversity of Cultivable Protease-Producing Bacteria

JOURNAL Unpublished

REFERENCE 2 (bases 1 to 640)

AUTHORS Su,H., Huang,Q. and Yu,K.

TITLE Direct Submission

JOURNAL Submitted (11-MAR-2019) Coral Reef Research Center of China, School

of Marine Sciences, No. 100 Daxue Road, Xixiangtang District,

Nanning, Guangxi Zhuang Autonomous Region 530004, China

COMMENT ##Assembly-Data-START##

Sequencing Technology :: Sanger dideoxy sequencing

##Assembly-Data-END##

FEATURES Location/Qualifiers

source 1..640

/organism="Bacillus sp. (in: Bacteria)"

/mol_type="genomic DNA"

/strain="sQ50"

/isolation_source="coral"

/db_xref="taxon:1409"

rRNA <1..>640

/product="16S ribosomal RNA"

ORIGIN

1 ggacagatgg gagcttgctc cctgaagtca gcggcggacg ggtgagtaac acgtgggcaa

61 cctgcctgta agactgggat aactccggga aaccggggct aataccggat aattctttcc

121 ctcacatgag ggaaagctga aagatggttt cggctatcac ttacagatgg gcccgcggcg

181 cattagctag ttggtgaggt aacggctcac caaggcaacg atgcgtagcc gacctgagag

241 ggtgatcggc cacactggga ctgagacacg gcccagactc ctacgggagg cagcagtagg

301 gaatcttccg caatggacga aagtctgacg gagcaacgcc gcgtgagtga tgaaggtttt

361 cggatcgtaa aactctgttg ttagggaaga acaagtaccg gagtaactgc cggtaccttg

421 acggtaccta accagaaagc cacggctaac tacgtgccag cagccgcggt aatacgtagg

481 tggcaagcgt tgtccggaat tattgggcgt aaagcgcgcg caggcggttc cttaagtctg

541 atgtgaaagc ccccggctca accggggagg gtcattggaa actggggaac ttgagtgcag

601 aagagaagag tggaattcca cgtgtagcgg tgaaatgcgt

//

LOCUS MK617735 640 bp DNA linear BCT 30-MAY-2019

DEFINITION Vibrio sp. strain sQ56 16S ribosomal RNA gene, partial sequence.

ACCESSION MK617735

VERSION MK617735

KEYWORDS .

SOURCE Vibrio sp.

ORGANISM Vibrio sp.

Bacteria; Proteobacteria; Gammaproteobacteria; Vibrionales;

Vibrionaceae; Vibrio.

REFERENCE 1 (bases 1 to 640)

AUTHORS Su,H., Huang,Q. and Yu,K.

TITLE Diversity of Cultivable Protease-Producing Bacteria

JOURNAL Unpublished

REFERENCE 2 (bases 1 to 640)

AUTHORS Su,H., Huang,Q. and Yu,K.

TITLE Direct Submission

JOURNAL Submitted (11-MAR-2019) Coral Reef Research Center of China, School

of Marine Sciences, No. 100 Daxue Road, Xixiangtang District,

Nanning, Guangxi Zhuang Autonomous Region 530004, China

COMMENT ##Assembly-Data-START##

Sequencing Technology :: Sanger dideoxy sequencing

##Assembly-Data-END##

FEATURES Location/Qualifiers

source 1..640

/organism="Vibrio sp."

/mol_type="genomic DNA"

/strain="sQ56"

/isolation_source="coral"

/db_xref="taxon:678"

rRNA <1..>640

/product="16S ribosomal RNA"

ORIGIN

1 cgagttatct gaaccttcgg ggaacgataa cggcgtcgag cggcggacgg gtgagtaatg

61 cctaggaaat tgccctgatg tgggggataa ccattggaaa cgatggctaa taccgcatga

121 tgcctacggg ccaaagaggg ggaccttcgg gcctctcgcg tcaggatatg cctaggtggg

181 attagctagt tggtgaggta agggctcacc aaggcgacga tccctagctg gtctgagagg

241 atgatcagcc acactggaac tgagacacgg tccagactcc tacgggaggc agcagtgggg

301 aatattgcac aatgggcgca agcctgatgc agccatgccg cgtgtgtgaa gaaggccttc

361 gggttgtaaa gcactttcag tcgtgaggaa ggtggtgtag ttaatagctg cattatttga

421 cgttagcgac agaagaagca ccggctaact ccgtgccagc agccgcggta atacggaggg

481 tgcgagcgtt aatcggaatt actgggcgta aagcgcatgc aggtggtttg ttaagtcaga

541 tgtgaaagcc cggggctcaa cctcggaata gcatttgaaa ctggcagact agagtactgt

601 agaggggggt agaatttcag gtgtagcggt gaaatgcgta

//

LOCUS MK617736 640 bp DNA linear BCT 30-MAY-2019

DEFINITION Vibrio sp. strain sQ57 16S ribosomal RNA gene, partial sequence.

ACCESSION MK617736

VERSION MK617736

KEYWORDS .

SOURCE Vibrio sp.

ORGANISM Vibrio sp.

Bacteria; Proteobacteria; Gammaproteobacteria; Vibrionales;

Vibrionaceae; Vibrio.

REFERENCE 1 (bases 1 to 640)

AUTHORS Su,H., Huang,Q. and Yu,K.

TITLE Diversity of Cultivable Protease-Producing Bacteria

JOURNAL Unpublished

REFERENCE 2 (bases 1 to 640)

AUTHORS Su,H., Huang,Q. and Yu,K.

TITLE Direct Submission

JOURNAL Submitted (11-MAR-2019) Coral Reef Research Center of China, School

of Marine Sciences, No. 100 Daxue Road, Xixiangtang District,

Nanning, Guangxi Zhuang Autonomous Region 530004, China

COMMENT ##Assembly-Data-START##

Sequencing Technology :: Sanger dideoxy sequencing

##Assembly-Data-END##

FEATURES Location/Qualifiers

source 1..640

/organism="Vibrio sp."

/mol_type="genomic DNA"

/strain="sQ57"

/isolation_source="coral"

/db_xref="taxon:678"

rRNA <1..>640

/product="16S ribosomal RNA"

ORIGIN

1 gagttatctg aaccttcggg gaacgataac ggcgtcgagc ggcggacggg tgagtaatgc

61 ctaggaaatt gccctgatgt gggggataac cattggaaac gatggctaat accgcatgat

121 gcctacgggc caaagagggg gaccttcggg cctctcgcgt caggatatgc ctaggtggga

181 ttagctagtt ggtgaggtaa gggctcacca aggcgacgat ccctagctgg tctgagagga

241 tgatcagcca cactggaact gagacacggt ccagactcct acgggaggca gcagtgggga

301 atattgcaca atgggcgcaa gcctgatgca gccatgccgc gtgtgtgaag aaggccttcg

361 ggttgtaaag cactttcagt cgtgaggaag gtggtgtagt taatagctgc attatttgac

421 gttagcgaca gaagaagcac cggctaactc cgtgccagca gccgcggtaa tacggagggt

481 gcgagcgtta atcggaatta ctgggcgtaa agcgcatgca ggtggtttgt taagtcagat

541 gtgaaagccc ggggctcaac ctcggaatag catttgaaac tggcagacta gagtactgta

601 gaggggggta gaatttcagg tgtagcggtg aaatgcgtag

//

LOCUS MK617737 640 bp DNA linear BCT 30-MAY-2019

DEFINITION Pseudovibrio sp. strain yL4cb 16S ribosomal RNA gene, partial

sequence.

ACCESSION MK617737

VERSION MK617737

KEYWORDS .

SOURCE Pseudovibrio sp.

ORGANISM Pseudovibrio sp.

Bacteria; Proteobacteria; Alphaproteobacteria; Rhodobacterales;

Rhodobacteraceae; Pseudovibrio.

REFERENCE 1 (bases 1 to 640)

AUTHORS Su,H., Huang,Q. and Yu,K.

TITLE Diversity of Cultivable Protease-Producing Bacteria

JOURNAL Unpublished

REFERENCE 2 (bases 1 to 640)

AUTHORS Su,H., Huang,Q. and Yu,K.

TITLE Direct Submission

JOURNAL Submitted (11-MAR-2019) Coral Reef Research Center of China, School

of Marine Sciences, No. 100 Daxue Road, Xixiangtang District,

Nanning, Guangxi Zhuang Autonomous Region 530004, China

COMMENT ##Assembly-Data-START##

Sequencing Technology :: Sanger dideoxy sequencing

##Assembly-Data-END##

FEATURES Location/Qualifiers

source 1..640

/organism="Pseudovibrio sp."

/mol_type="genomic DNA"

/strain="yL4cb"

/isolation_source="coral"

/db_xref="taxon:1909297"

rRNA <1..>640

/product="16S ribosomal RNA"

ORIGIN

1 gaacggatcc ttcgggatta gtggcagacg ggtgagtaac gcgtgggaag ctaccttgtg

61 gtagggaaca acagttggaa acgactgcta ataccctatg agccctatgg gggaaagatt

121 tatcgccatg agatgtgccc gcgttagatt agctagttgg taaggtaatg gcttaccaag

181 gcgacgatct atagctggtc tgagaggatg atcagccaca ctgggactga gacacggccc

241 agactcctac gggaggcagc agtggggaat attggacaat gggggcaacc ctgatccagc

301 catgccgcgt gagtgatgac ggccttaggg ttgtaaagct ctttcagcag tgaagataat

361 gacattaact gcagaagaag ccccggctaa cttcgtgcca gcagccgcgg taatacgaag

421 ggggctagcg ttgttcggaa tcactgggcg taaagcgtac gtaggcggac tgatcagtca

481 ggggtgaaat cccggggctc aaccccggaa ctgcctttga tactgtcagt cttgagatcg

541 agagaggtga gtggaactcc gagtgtagag gtgaaattcg tagatattcg gaagaacacc

601 agtggcgaag gcggctcact ggctcgatac tgacgctgag

//

LOCUS MK617738 640 bp DNA linear BCT 30-MAY-2019

DEFINITION Vibrio sp. strain sQ61 16S ribosomal RNA gene, partial sequence.

ACCESSION MK617738

VERSION MK617738

KEYWORDS .

SOURCE Vibrio sp.

ORGANISM Vibrio sp.

Bacteria; Proteobacteria; Gammaproteobacteria; Vibrionales;

Vibrionaceae; Vibrio.

REFERENCE 1 (bases 1 to 640)

AUTHORS Su,H., Huang,Q. and Yu,K.

TITLE Diversity of Cultivable Protease-Producing Bacteria

JOURNAL Unpublished

REFERENCE 2 (bases 1 to 640)

AUTHORS Su,H., Huang,Q. and Yu,K.

TITLE Direct Submission

JOURNAL Submitted (11-MAR-2019) Coral Reef Research Center of China, School

of Marine Sciences, No. 100 Daxue Road, Xixiangtang District,

Nanning, Guangxi Zhuang Autonomous Region 530004, China

COMMENT ##Assembly-Data-START##

Sequencing Technology :: Sanger dideoxy sequencing

##Assembly-Data-END##

FEATURES Location/Qualifiers

source 1..640

/organism="Vibrio sp."

/mol_type="genomic DNA"

/strain="sQ61"

/isolation_source="coral"

/db_xref="taxon:678"

rRNA <1..>640

/product="16S ribosomal RNA"

ORIGIN

1 gagttatctg aaccttcggg gaacgataac ggcgtcgagc ggcggacggg tgagtaatgc

61 ctaggaaatt gccctgatgt gggggataac cattggaaac gatggctaat accgcatgat

121 gcctacgggc caaagagggg gaccttcggg cctctcgcgt caggatatgc ctaggtggga

181 ttagctagtt ggtgaggtaa gggctcacca aggcgacgat ccctagctgg tctgagagga

241 tgatcagcca cactggaact gagacacggt ccagactcct acgggaggca gcagtgggga

301 atattgcaca atgggcgcaa gcctgatgca gccatgccgc gtgtgtgaag aaggccttcg

361 ggttgtaaag cactttcagt cgtgaggaag gtggtgtagt taatagctgc attatttgac

421 gttagcgaca gaagaagcac cggctaactc cgtgccagca gccgcggtaa tacggagggt

481 gcgagcgtta atcggaatta ctgggcgtaa agcgcatgca ggtggtttgt taagtcagat

541 gtgaaagccc ggggctcaac ctcggaatag catttgaaac tggcagacta gagtactgta

601 gaggggggta gaatttcagg tgtagcggtg aaatgcgtag

//

LOCUS MK617739 640 bp DNA linear BCT 30-MAY-2019

DEFINITION Bacillus sp. (in: Bacteria) strain yA3 16S ribosomal RNA gene,

partial sequence.

ACCESSION MK617739

VERSION MK617739

KEYWORDS .

SOURCE Bacillus sp. (in: Bacteria)

ORGANISM Bacillus sp. (in: Bacteria)

Bacteria; Firmicutes; Bacilli; Bacillales; Bacillaceae; Bacillus.

REFERENCE 1 (bases 1 to 640)

AUTHORS Su,H., Huang,Q. and Yu,K.

TITLE Diversity of Cultivable Protease-Producing Bacteria

JOURNAL Unpublished

REFERENCE 2 (bases 1 to 640)

AUTHORS Su,H., Huang,Q. and Yu,K.

TITLE Direct Submission

JOURNAL Submitted (11-MAR-2019) Coral Reef Research Center of China, School

of Marine Sciences, No. 100 Daxue Road, Xixiangtang District,

Nanning, Guangxi Zhuang Autonomous Region 530004, China

COMMENT ##Assembly-Data-START##

Sequencing Technology :: Sanger dideoxy sequencing

##Assembly-Data-END##

FEATURES Location/Qualifiers

source 1..640

/organism="Bacillus sp. (in: Bacteria)"

/mol_type="genomic DNA"

/strain="yA3"

/isolation_source="coral"

/db_xref="taxon:1409"

rRNA <1..>640

/product="16S ribosomal RNA"

ORIGIN

1 acagatggga gcttgctccc tgaagtcagc ggcggacggg tgagtaacac gtgggcaacc

61 tgcctgtaag actgggataa ctccgggaaa ccggggctaa taccggataa ttctttccct

121 cacatgaggg aaagctgaaa gatggtttcg gctatcactt acagatgggc ccgcggcgca

181 ttagctagtt ggtgaggtaa cggctcacca aggcaacgat gcgtagccga cctgagaggg

241 tgatcggcca cactgggact gagacacggc ccagactcct acgggaggca gcagtaggga

301 atcttccgca atggacgaaa gtctgacgga gcaacgccgc gtgagtgatg aaggttttcg

361 gatcgtaaaa ctctgttgtt agggaagaac aagtaccgga gtaactgccg gtaccttgac

421 ggtacctaac cagaaagcca cggctaacta cgtgccagca gccgcggtaa tacgtaggtg

481 gcaagcgttg tccggaatta ttgggcgtaa agcgcgcgca ggcggttcct taagtctgat

541 gtgaaagccc ccggctcaac cggggagggt cattggaaac tggggaactt gagtgcagaa

601 gagaagagtg gaattccacg tgtagcggtg aaatgcgtag

//

LOCUS MK617740 640 bp DNA linear BCT 30-MAY-2019

DEFINITION Fictibacillus sp. strain yA27 16S ribosomal RNA gene, partial

sequence.

ACCESSION MK617740

VERSION MK617740

KEYWORDS .

SOURCE Fictibacillus sp.

ORGANISM Fictibacillus sp.

Bacteria; Firmicutes; Bacilli; Bacillales; Bacillaceae;

Fictibacillus.

REFERENCE 1 (bases 1 to 640)

AUTHORS Su,H., Huang,Q. and Yu,K.

TITLE Diversity of Cultivable Protease-Producing Bacteria

JOURNAL Unpublished

REFERENCE 2 (bases 1 to 640)

AUTHORS Su,H., Huang,Q. and Yu,K.

TITLE Direct Submission

JOURNAL Submitted (11-MAR-2019) Coral Reef Research Center of China, School

of Marine Sciences, No. 100 Daxue Road, Xixiangtang District,

Nanning, Guangxi Zhuang Autonomous Region 530004, China

COMMENT ##Assembly-Data-START##

Sequencing Technology :: Sanger dideoxy sequencing

##Assembly-Data-END##

FEATURES Location/Qualifiers

source 1..640

/organism="Fictibacillus sp."

/mol_type="genomic DNA"

/strain="yA27"

/isolation_source="coral"

/db_xref="taxon:1871617"

rRNA <1..>640

/product="16S ribosomal RNA"

ORIGIN

1 tgatgaggag cttgctcctc tgatttagcg gcggacgggt gagtaacacg tgggtaatct

61 gcctgtaaga cggggataac tccgggaaac cggggctaat accggataat aagagaagaa

121 gcatttcttc tttttgaaag tcggtttcgg ctgacactta cagatgagcc cgcggcgcat

181 tagctagttg gtgaggtaac ggctcaccaa ggcgacgatg cgtagccgac ctgagagggt

241 gatcggccac actgggactg agacacggcc cagactccta cgggaggcag cagtagggaa

301 tcttcggcaa tgggcgaaag cctgaccgag caacgccgcg tgagcgatga aggccttcgg

361 gtcgtaaagc tctgttgtta gagaagaaca agtacgagag taactgctcg taccttgacg

421 gtacctaacc agaaagccac ggctaactac gtgccagcag ccgcggtaat acgtaggtgg

481 caagcgttat ccggaattat tgggcgtaaa gcgcgcgcag gcggtctctt aagtctgatg

541 tgaaagccca cggctcaacc gtggagggtc attggaaact gggagacttg agtgcaggag

601 agaaaagtgg aattccacgt gtagcggtga aatgcgtaga

//

LOCUS MK617741 640 bp DNA linear BCT 30-MAY-2019

DEFINITION Exiguobacterium sp. strain yB14b 16S ribosomal RNA gene, partial

sequence.

ACCESSION MK617741

VERSION MK617741

KEYWORDS .

SOURCE Exiguobacterium sp.

ORGANISM Exiguobacterium sp.

Bacteria; Firmicutes; Bacilli; Bacillales; Bacillales Family XII.

Incertae Sedis; Exiguobacterium.

REFERENCE 1 (bases 1 to 640)

AUTHORS Su,H., Huang,Q. and Yu,K.

TITLE Diversity of Cultivable Protease-Producing Bacteria

JOURNAL Unpublished

REFERENCE 2 (bases 1 to 640)

AUTHORS Su,H., Huang,Q. and Yu,K.

TITLE Direct Submission

JOURNAL Submitted (11-MAR-2019) Coral Reef Research Center of China, School

of Marine Sciences, No. 100 Daxue Road, Xixiangtang District,

Nanning, Guangxi Zhuang Autonomous Region 530004, China

COMMENT ##Assembly-Data-START##

Sequencing Technology :: Sanger dideoxy sequencing

##Assembly-Data-END##

FEATURES Location/Qualifiers

source 1..640

/organism="Exiguobacterium sp."

/mol_type="genomic DNA"

/strain="yB14b"

/isolation_source="coral"

/db_xref="taxon:44751"

rRNA <1..>640

/product="16S ribosomal RNA"

ORIGIN

1 aggaagtcga cggaaccctt cggggggaag tcgacggaat gagcggcgga cgggtgagta

61 acacgtaaag aacctgccct caggtctggg ataaccacga gaaatcgggg ctaataccgg

121 atgggtcatc ggaccgcatg gtccgaggat gaaaggcgct ccggcgtcgc ctggggatgg

181 ctttgcggtg cattagctag ttggtggggt aatggcccac caaggcgacg atgcatagcc

241 gacctgagag ggtgatcggc cacactggga ctgagacacg gcccagactc ctacgggagg

301 cagcagtagg gaatcttcca caatggacga aagtctgatg gagcaacgcc gcgtgaacga

361 tgaaggcctt cgggtcgtaa agttctgttg taagggaaga acaagtgccg caggcaatgg

421 cggcaccttg acggtacctt gcgagaaagc cacggctaac tacgtgccag cagccgcggt

481 aatacgtagg tggcaagcgt tgtccggaat tattgggcgt aaagcgcgcg caggcggcct

541 cttaagtctg atgtgaaagc ccccggctca accggggagg gccattggaa actgggaggc

601 ttgagtatag gagagaagag tggaattcca cgtgtagcgg

//

LOCUS MK617742 640 bp DNA linear BCT 30-MAY-2019

DEFINITION Microbacterium sp. strain yB18 16S ribosomal RNA gene, partial

sequence.

ACCESSION MK617742

VERSION MK617742

KEYWORDS .

SOURCE Microbacterium sp.

ORGANISM Microbacterium sp.

Bacteria; Actinobacteria; Micrococcales; Microbacteriaceae;

Microbacterium.

REFERENCE 1 (bases 1 to 640)

AUTHORS Su,H., Huang,Q. and Yu,K.

TITLE Diversity of Cultivable Protease-Producing Bacteria

JOURNAL Unpublished

REFERENCE 2 (bases 1 to 640)

AUTHORS Su,H., Huang,Q. and Yu,K.

TITLE Direct Submission

JOURNAL Submitted (11-MAR-2019) Coral Reef Research Center of China, School

of Marine Sciences, No. 100 Daxue Road, Xixiangtang District,

Nanning, Guangxi Zhuang Autonomous Region 530004, China

COMMENT ##Assembly-Data-START##

Sequencing Technology :: Sanger dideoxy sequencing

##Assembly-Data-END##

FEATURES Location/Qualifiers

source 1..640

/organism="Microbacterium sp."

/mol_type="genomic DNA"

/strain="yB18"

/isolation_source="coral"

/db_xref="taxon:51671"

rRNA <1..>640

/product="16S ribosomal RNA"

ORIGIN

1 agcccagctt gctgggtgga ttagtggcga acgggtgagt aacacgtgag caacctgccc

61 ctgactctgg gataagcgct ggaaacggcg tctaatactg gatatgtccc gtcaccgcat

121 ggtgtgcggg tggaaagatt tttcggttgg ggatgggctc gcggcctatc agcttgttgg

181 tgaggtaatg gctcaccaag gcgtcgacgg gtagccggcc tgagagggtg accggccaca

241 ctgggactga gacacggccc agactcctac gggaggcagc agtggggaat attgcacaat

301 gggcgcaagc ctgatgcagc aacgccgcgt gagggatgac ggccttcggg ttgtaaacct

361 cttttagcag ggaagaagcg agagtgacgg tacctgcaga aaaagcaccg gctaactacg

421 tgccagcagc cgcggtaata cgtagggtgc aagcgttatc cggaattatt gggcgtaaag

481 agctcgtagg cggtctgtcg cgtctgctgt gaaatcccga ggctcaacct cgggcttgca

541 gtgggtacgg gcagactaga gtgcggtagg ggagattgga attcctggtg tagcggtgga

601 atgcgcagat atcaggagga acaccgatgg cgaaggcaga

//

LOCUS MK617743 640 bp DNA linear BCT 30-MAY-2019

DEFINITION Fictibacillus sp. strain yC8 16S ribosomal RNA gene, partial

sequence.

ACCESSION MK617743

VERSION MK617743

KEYWORDS .

SOURCE Fictibacillus sp.

ORGANISM Fictibacillus sp.

Bacteria; Firmicutes; Bacilli; Bacillales; Bacillaceae;

Fictibacillus.

REFERENCE 1 (bases 1 to 640)

AUTHORS Su,H., Huang,Q. and Yu,K.

TITLE Diversity of Cultivable Protease-Producing Bacteria

JOURNAL Unpublished

REFERENCE 2 (bases 1 to 640)

AUTHORS Su,H., Huang,Q. and Yu,K.

TITLE Direct Submission

JOURNAL Submitted (11-MAR-2019) Coral Reef Research Center of China, School

of Marine Sciences, No. 100 Daxue Road, Xixiangtang District,

Nanning, Guangxi Zhuang Autonomous Region 530004, China

COMMENT ##Assembly-Data-START##

Sequencing Technology :: Sanger dideoxy sequencing

##Assembly-Data-END##

FEATURES Location/Qualifiers

source 1..640

/organism="Fictibacillus sp."

/mol_type="genomic DNA"

/strain="yC8"

/isolation_source="coral"

/db_xref="taxon:1871617"

rRNA <1..>640

/product="16S ribosomal RNA"

ORIGIN

1 tgaagaggag cttgctcctc tgatttagcg gcggacgggt gagtaacacg tgggtaatct

61 gcctgtaaga cggggataac tccgggaaac cggggctaat accggataat aagaagaaac

121 gcatgtttct tttttgaaag tcggtttcgg ctgacactta cagatgagcc cgcggcgcat

181 tagctagttg gtgaggtaac ggctcaccaa ggcgacgatg cgtagccgac ctgagagggt

241 gatcggccac actgggactg agacacggcc cagactccta cgggaggcag cagtagggaa

301 tcttcggcaa tgggcgaaag cctgaccgag caacgccgcg tgagcgatga aggccttcgg

361 gtcgtaaagc tctgttgtta gagaagaaca agtacgagag taactgctcg taccttgacg

421 gtacctaacc agaaagccac ggctaactac gtgccagcag ccgcggtaat acgtaggtgg

481 caagcgttat ccggaattat tgggcgtaaa gcgcgcgcag gcggtctctt aagtctgatg

541 tgaaagccca cggctcaacc gtggagggtc attggaaact gggagacttg agtgcaggag

601 agaaaagtgg aattccacgt gtagcggtga aatgcgtaga

//

LOCUS MK617744 640 bp DNA linear BCT 30-MAY-2019

DEFINITION Bacillus sp. (in: Bacteria) strain yD3 16S ribosomal RNA gene,

partial sequence.

ACCESSION MK617744

VERSION MK617744

KEYWORDS .

SOURCE Bacillus sp. (in: Bacteria)

ORGANISM Bacillus sp. (in: Bacteria)

Bacteria; Firmicutes; Bacilli; Bacillales; Bacillaceae; Bacillus.

REFERENCE 1 (bases 1 to 640)

AUTHORS Su,H., Huang,Q. and Yu,K.

TITLE Diversity of Cultivable Protease-Producing Bacteria

JOURNAL Unpublished

REFERENCE 2 (bases 1 to 640)

AUTHORS Su,H., Huang,Q. and Yu,K.

TITLE Direct Submission

JOURNAL Submitted (11-MAR-2019) Coral Reef Research Center of China, School

of Marine Sciences, No. 100 Daxue Road, Xixiangtang District,

Nanning, Guangxi Zhuang Autonomous Region 530004, China

COMMENT ##Assembly-Data-START##

Sequencing Technology :: Sanger dideoxy sequencing

##Assembly-Data-END##

FEATURES Location/Qualifiers

source 1..640

/organism="Bacillus sp. (in: Bacteria)"

/mol_type="genomic DNA"

/strain="yD3"

/isolation_source="coral"

/db_xref="taxon:1409"

rRNA <1..>640

/product="16S ribosomal RNA"

ORIGIN

1 acagatggga gcttgctccc tgaagtcagc ggcggacggg tgagtaacac gtgggcaacc

61 tgcctgtaag actgggataa ctccgggaaa ccggggctaa taccggataa ttctttccct

121 cacatgaggg aaagctgaaa gatggtttcg gctatcactt acagatgggc ccgcggcgca

181 ttagctagtt ggtgaggtaa cggctcacca aggcaacgat gcgtagccga cctgagaggg

241 tgatcggcca cactgggact gagacacggc ccagactcct acgggaggca gcagtaggga

301 atcttccgca atggacgaaa gtctgacgga gcaacgccgc gtgagtgatg aaggttttcg

361 gatcgtaaaa ctctgttgtt agggaagaac aagtaccgga gtaactgccg gtaccttgac

421 ggtacctaac cagaaagcca cggctaacta cgtgccagca gccgcggtaa tacgtaggtg

481 gcaagcgttg tccggaatta ttgggcgtaa agcgcgcgca ggcggttcct taagtctgat

541 gtgaaagccc ccggctcaac cggggagggt cattggaaac tggggaactt gagtgcagaa

601 gagaagagtg gaattccacg tgtagcggtg aaatgcgtag

//

LOCUS MK617745 640 bp DNA linear BCT 30-MAY-2019

DEFINITION Bacillus sp. (in: Bacteria) strain yD5 16S ribosomal RNA gene,

partial sequence.

ACCESSION MK617745

VERSION MK617745

KEYWORDS .

SOURCE Bacillus sp. (in: Bacteria)

ORGANISM Bacillus sp. (in: Bacteria)

Bacteria; Firmicutes; Bacilli; Bacillales; Bacillaceae; Bacillus.

REFERENCE 1 (bases 1 to 640)

AUTHORS Su,H., Huang,Q. and Yu,K.

TITLE Diversity of Cultivable Protease-Producing Bacteria

JOURNAL Unpublished

REFERENCE 2 (bases 1 to 640)

AUTHORS Su,H., Huang,Q. and Yu,K.

TITLE Direct Submission

JOURNAL Submitted (11-MAR-2019) Coral Reef Research Center of China, School

of Marine Sciences, No. 100 Daxue Road, Xixiangtang District,

Nanning, Guangxi Zhuang Autonomous Region 530004, China

COMMENT ##Assembly-Data-START##

Sequencing Technology :: Sanger dideoxy sequencing

##Assembly-Data-END##

FEATURES Location/Qualifiers

source 1..640

/organism="Bacillus sp. (in: Bacteria)"

/mol_type="genomic DNA"

/strain="yD5"

/isolation_source="coral"

/db_xref="taxon:1409"

rRNA <1..>640

/product="16S ribosomal RNA"

ORIGIN

1 acagatggga gcttgctccc tgaagtcagc ggcggacggg tgagtaacac gtgggcaacc

61 tgcctgtaag actgggataa ctccgggaaa ccggggctaa taccggataa ttctttccct

121 cacatgaggg aaagctgaaa gatggtttcg gctatcactt acagatgggc ccgcggcgca

181 ttagctagtt ggtgaggtaa cggctcacca aggcaacgat gcgtagccga cctgagaggg

241 tgatcggcca cactgggact gagacacggc ccagactcct acgggaggca gcagtaggga

301 atcttccgca atggacgaaa gtctgacgga gcaacgccgc gtgagtgatg aaggttttcg

361 gatcgtaaaa ctctgttgtt agggaagaac aagtaccgga gtaactgccg gtaccttgac

421 ggtacctaac cagaaagcca cggctaacta cgtgccagca gccgcggtaa tacgtaggtg

481 gcaagcgttg tccggaatta ttgggcgtaa agcgcgcgca ggcggttcct taagtctgat

541 gtgaaagccc ccggctcaac cggggagggt cattggaaac tggggaactt gagtgcagaa

601 gagaagagtg gaattccacg tgtagcggtg aaatgcgtag

//

LOCUS MK617746 640 bp DNA linear BCT 30-MAY-2019

DEFINITION Bacillus sp. (in: Bacteria) strain yD6 16S ribosomal RNA gene,

partial sequence.

ACCESSION MK617746

VERSION MK617746

KEYWORDS .

SOURCE Bacillus sp. (in: Bacteria)

ORGANISM Bacillus sp. (in: Bacteria)

Bacteria; Firmicutes; Bacilli; Bacillales; Bacillaceae; Bacillus.

REFERENCE 1 (bases 1 to 640)

AUTHORS Su,H., Huang,Q. and Yu,K.

TITLE Diversity of Cultivable Protease-Producing Bacteria

JOURNAL Unpublished

REFERENCE 2 (bases 1 to 640)

AUTHORS Su,H., Huang,Q. and Yu,K.

TITLE Direct Submission

JOURNAL Submitted (11-MAR-2019) Coral Reef Research Center of China, School

of Marine Sciences, No. 100 Daxue Road, Xixiangtang District,

Nanning, Guangxi Zhuang Autonomous Region 530004, China

COMMENT ##Assembly-Data-START##

Sequencing Technology :: Sanger dideoxy sequencing

##Assembly-Data-END##

FEATURES Location/Qualifiers

source 1..640

/organism="Bacillus sp. (in: Bacteria)"

/mol_type="genomic DNA"

/strain="yD6"

/isolation_source="coral"

/db_xref="taxon:1409"

rRNA <1..>640

/product="16S ribosomal RNA"

ORIGIN

1 acagatggga gcttgctccc tgaagtcagc ggcggacggg tgagtaacac gtgggcaacc

61 tgcctgtaag actgggataa ctccgggaaa ccggggctaa taccggataa ttctttccct

121 cacatgaggg aaagctgaaa gatggtttcg gctatcactt acagatgggc ccgcggcgca

181 ttagctagtt ggtgaggtaa cggctcacca aggcaacgat gcgtagccga cctgagaggg

241 tgatcggcca cactgggact gagacacggc ccagactcct acgggaggca gcagtaggga

301 atcttccgca atggacgaaa gtctgacgga gcaacgccgc gtgagtgatg aaggttttcg

361 gatcgtaaaa ctctgttgtt agggaagaac aagtaccgga gtaactgccg gtaccttgac

421 ggtacctaac cagaaagcca cggctaacta cgtgccagca gccgcggtaa tacgtaggtg

481 gcaagcgttg tccggaatta ttgggcgtaa agcgcgcgca ggcggttcct taagtctgat

541 gtgaaagccc ccggctcaac cggggagggt cattggaaac tggggaactt gagtgcagaa

601 gagaagagtg gaattccacg tgtagcggtg aaatgcgtag

//

LOCUS MK617747 640 bp DNA linear BCT 30-MAY-2019

DEFINITION Exiguobacterium sp. strain yD8 16S ribosomal RNA gene, partial

sequence.

ACCESSION MK617747

VERSION MK617747

KEYWORDS .

SOURCE Exiguobacterium sp.

ORGANISM Exiguobacterium sp.

Bacteria; Firmicutes; Bacilli; Bacillales; Bacillales Family XII.

Incertae Sedis; Exiguobacterium.

REFERENCE 1 (bases 1 to 640)

AUTHORS Su,H., Huang,Q. and Yu,K.

TITLE Diversity of Cultivable Protease-Producing Bacteria

JOURNAL Unpublished

REFERENCE 2 (bases 1 to 640)

AUTHORS Su,H., Huang,Q. and Yu,K.

TITLE Direct Submission

JOURNAL Submitted (11-MAR-2019) Coral Reef Research Center of China, School

of Marine Sciences, No. 100 Daxue Road, Xixiangtang District,

Nanning, Guangxi Zhuang Autonomous Region 530004, China

COMMENT ##Assembly-Data-START##

Sequencing Technology :: Sanger dideoxy sequencing

##Assembly-Data-END##

FEATURES Location/Qualifiers

source 1..640

/organism="Exiguobacterium sp."

/mol_type="genomic DNA"

/strain="yD8"

/isolation_source="coral"

/db_xref="taxon:44751"

rRNA <1..>640

/product="16S ribosomal RNA"

ORIGIN

1 aggaagtcga cggaaccctt cggggggaag tcgacggaat gagcggcgga cgggtgagta

61 acacgtaaag aacctgccct caggtctggg ataaccacga gaaatcgggg ctaataccgg

121 atgggtcatc ggaccgcatg gtccgaggat gaaaggcgct ccggcgtcgc ctggggatgg

181 ctttgcggtg cattagctag ttggtggggt aatggcccac caaggcgacg atgcatagcc

241 gacctgagag ggtgatcggc cacactggga ctgagacacg gcccagactc ctacgggagg

301 cagcagtagg gaatcttcca caatggacga aagtctgatg gagcaacgcc gcgtgaacga

361 tgaaggcctt cgggtcgtaa agttctgttg taagggaaga acaagtgccg caggcaatgg

421 cggcaccttg acggtacctt gcgagaaagc cacggctaac tacgtgccag cagccgcggt

481 aatacgtagg tggcaagcgt tgtccggaat tattgggcgt aaagcgcgcg caggcggcct

541 cttaagtctg atgtgaaagc ccccggctca accggggagg gccattggaa actgggaggc

601 ttgagtatag gagagaagag tggaattcca cgtgtagcgg

//

LOCUS MK617748 640 bp DNA linear BCT 30-MAY-2019

DEFINITION Exiguobacterium sp. strain yF2 16S ribosomal RNA gene, partial

sequence.

ACCESSION MK617748

VERSION MK617748

KEYWORDS .

SOURCE Exiguobacterium sp.

ORGANISM Exiguobacterium sp.

Bacteria; Firmicutes; Bacilli; Bacillales; Bacillales Family XII.

Incertae Sedis; Exiguobacterium.

REFERENCE 1 (bases 1 to 640)

AUTHORS Su,H., Huang,Q. and Yu,K.

TITLE Diversity of Cultivable Protease-Producing Bacteria

JOURNAL Unpublished

REFERENCE 2 (bases 1 to 640)

AUTHORS Su,H., Huang,Q. and Yu,K.

TITLE Direct Submission

JOURNAL Submitted (11-MAR-2019) Coral Reef Research Center of China, School

of Marine Sciences, No. 100 Daxue Road, Xixiangtang District,

Nanning, Guangxi Zhuang Autonomous Region 530004, China

COMMENT ##Assembly-Data-START##

Sequencing Technology :: Sanger dideoxy sequencing

##Assembly-Data-END##

FEATURES Location/Qualifiers

source 1..640

/organism="Exiguobacterium sp."

/mol_type="genomic DNA"

/strain="yF2"

/isolation_source="coral"

/db_xref="taxon:44751"

rRNA <1..>640

/product="16S ribosomal RNA"

ORIGIN

1 ggaagtcgac ggaacccttc ggggggaagt cgacggaatg agcggcggac gggtgagtaa

61 cacgtaaaga acctgccctc aggtctggga taaccacgag aaatcggggc taataccgga

121 tgggtcatcg gaccgcatgg tccgaggatg aaaggcgctc cggcgtcgcc tggggatggc

181 tttgcggtgc attagctagt tggtggggta atggcccacc aaggcgacga tgcatagccg

241 acctgagagg gtgatcggcc acactgggac tgagacacgg cccagactcc tacgggaggc

301 agcagtaggg aatcttccac aatggacgaa agtctgatgg agcaacgccg cgtgaacgat

361 gaaggccttc gggtcgtaaa gttctgttgt aagggaagaa caagtgccgc aggcaatggc

421 ggcaccttga cggtaccttg cgagaaagcc acggctaact acgtgccagc agccgcggta

481 atacgtaggt ggcaagcgtt gtccggaatt attgggcgta aagcgcgcgc aggcggcctc

541 ttaagtctga tgtgaaagcc cccggctcaa ccggggaggg ccattggaaa ctgggaggct

601 tgagtatagg agagaagagt ggaattccac gtgtagcggt

//

LOCUS MK617749 640 bp DNA linear BCT 30-MAY-2019

DEFINITION Bacillus sp. (in: Bacteria) strain yF12B 16S ribosomal RNA gene,

partial sequence.

ACCESSION MK617749

VERSION MK617749

KEYWORDS .

SOURCE Bacillus sp. (in: Bacteria)

ORGANISM Bacillus sp. (in: Bacteria)

Bacteria; Firmicutes; Bacilli; Bacillales; Bacillaceae; Bacillus.

REFERENCE 1 (bases 1 to 640)

AUTHORS Su,H., Huang,Q. and Yu,K.

TITLE Diversity of Cultivable Protease-Producing Bacteria

JOURNAL Unpublished

REFERENCE 2 (bases 1 to 640)

AUTHORS Su,H., Huang,Q. and Yu,K.

TITLE Direct Submission

JOURNAL Submitted (11-MAR-2019) Coral Reef Research Center of China, School

of Marine Sciences, No. 100 Daxue Road, Xixiangtang District,

Nanning, Guangxi Zhuang Autonomous Region 530004, China

COMMENT ##Assembly-Data-START##

Sequencing Technology :: Sanger dideoxy sequencing

##Assembly-Data-END##

FEATURES Location/Qualifiers

source 1..640

/organism="Bacillus sp. (in: Bacteria)"

/mol_type="genomic DNA"

/strain="yF12B"

/isolation_source="coral"

/db_xref="taxon:1409"

rRNA <1..>640

/product="16S ribosomal RNA"

ORIGIN

1 caatgggagc ttgctccctg aggttagcgg cggacgggtg agtaacacgt gggtaacctg

61 cctgtaagat tgggataact ccgggaaacc ggagctaata ccggataaca ttttgaaccg

121 catggttcga aattgaaaga tggtttcggc tatcacttac agatggaccc gcggcgcatt

181 agctagttgg tgaggtaacg gctcaccaag gcgacgatgc gtagccgacc tgagagggtg

241 atcggccaca ctgggactga gacacggccc agactcctac gggaggcagc agtagggaat

301 cttccgcaat ggacgaaagt ctgacggagc aacgccgcgt gaacgatgaa ggccttcggg

361 tcgtaaagtt ctgttgttag ggaagaacaa gtaccagagt aactgctggt accttgacgg

421 tacctaacca gaaagccacg gctaactacg tgccagcagc cgcggtaata cgtaggtggc

481 aagcgttgtc cggaattatt gggcgtaaag cgcgcgcagg cggtttctta agtctgatgt

541 gaaagcccac ggctcaaccg tggagggtca ttggaaactg gggaacttga gtgcagaaga

601 ggagagtgga attccacgtg tagcggtgaa atgcgtagag

//

LOCUS MK617750 640 bp DNA linear BCT 30-MAY-2019

DEFINITION Brevibacterium sp. strain yF15 16S ribosomal RNA gene, partial

sequence.

ACCESSION MK617750

VERSION MK617750

KEYWORDS .

SOURCE Brevibacterium sp.

ORGANISM Brevibacterium sp.

Bacteria; Actinobacteria; Micrococcales; Brevibacteriaceae;

Brevibacterium.

REFERENCE 1 (bases 1 to 640)

AUTHORS Su,H., Huang,Q. and Yu,K.

TITLE Diversity of Cultivable Protease-Producing Bacteria

JOURNAL Unpublished

REFERENCE 2 (bases 1 to 640)

AUTHORS Su,H., Huang,Q. and Yu,K.

TITLE Direct Submission

JOURNAL Submitted (11-MAR-2019) Coral Reef Research Center of China, School

of Marine Sciences, No. 100 Daxue Road, Xixiangtang District,

Nanning, Guangxi Zhuang Autonomous Region 530004, China

COMMENT ##Assembly-Data-START##

Sequencing Technology :: Sanger dideoxy sequencing

##Assembly-Data-END##

FEATURES Location/Qualifiers

source 1..640

/organism="Brevibacterium sp."

/mol_type="genomic DNA"

/strain="yF15"

/isolation_source="coral"

/db_xref="taxon:1701"

rRNA <1..>640

/product="16S ribosomal RNA"

ORIGIN

1 agcctggtgc ttgcaccggg tggatgagtg gcgaacgggt gagtaacacg tgagtaacct

61 gcccctgact tcgggataag cccgggaaac tgggtctaat accggatacg actgccggac

121 gcatgtctgg tggtggaaag ttttttcggt tggggatggg ctcgcggcct atcagtttgt

181 tggtgaggta atggctcacc aagacgacga cgggtagccg gcctgagagg gcgaccggcc

241 acactgggac tgagacacgg cccagactcc tacgggaggc agcagtgggg aatattgcac

301 aatgggggaa accctgatgc agcgacgcag cgtgcgggat gacggccttc gggttgtaaa

361 ccgctttcag cagggaagaa gcgccagtga ccgtacctgc gtaaaaagta ccggctaact

421 acatgccccc agccgcggta atacgtaggg gacaagcgtt gtccggaatt attgggcgta

481 aagagctctc atgtggttgg tcacgtctgc tgtggaaacg caacgcttaa cgttgcgcgt

541 gcgctgcgta cgggctgact agagtgcact aggggagtct ggaattcctg gtgtatcggt

601 gaaatgcgca catatctcga ggaacaccgg tggcgaaagc

//

LOCUS MK617751 640 bp DNA linear BCT 30-MAY-2019

DEFINITION Idiomarina sp. strain yL5c 16S ribosomal RNA gene, partial

sequence.

ACCESSION MK617751

VERSION MK617751

KEYWORDS .

SOURCE Idiomarina sp.

ORGANISM Idiomarina sp.

Bacteria; Proteobacteria; Gammaproteobacteria; Alteromonadales;

Idiomarinaceae; Idiomarina.

REFERENCE 1 (bases 1 to 640)

AUTHORS Su,H., Huang,Q. and Yu,K.

TITLE Diversity of Cultivable Protease-Producing Bacteria

JOURNAL Unpublished

REFERENCE 2 (bases 1 to 640)

AUTHORS Su,H., Huang,Q. and Yu,K.

TITLE Direct Submission

JOURNAL Submitted (11-MAR-2019) Coral Reef Research Center of China, School

of Marine Sciences, No. 100 Daxue Road, Xixiangtang District,

Nanning, Guangxi Zhuang Autonomous Region 530004, China

COMMENT ##Assembly-Data-START##

Sequencing Technology :: Sanger dideoxy sequencing

##Assembly-Data-END##

FEATURES Location/Qualifiers

source 1..640

/organism="Idiomarina sp."

/mol_type="genomic DNA"

/strain="yL5c"

/isolation_source="coral"

/db_xref="taxon:1874361"

rRNA <1..>640

/product="16S ribosomal RNA"

ORIGIN

1 cagagagaag cttgcttctt tgctgacgag cggcggacgg gtgagtaata cttgggaact

61 tgcctctagg cgggggacaa ccactggaaa cggtggctaa taccgcataa tgtctacgga

121 ccaaagtggg ggaccttcgg gcctcacacc tagagatggg cccaagtggg attagctagt

181 tggtgaggta aaggctcacc aaggcgacga tccctagctg ttctgagagg atgatcagcc

241 acactgggac tgagacacgg cccagactcc tacgggaggc agcagtgggg aatattgcac

301 aatgggcgca agcctgatgc agccatgccg cgtgtgtgaa gaaggccttc gggttgtaaa

361 gcactttcag tggtgaggaa ggtcagtaag ttaatagctt gctgaattga cgttagccac

421 agaagaagca ccggctaact ccgtgccagc agccgcggta atacggaggg tgcaagcgtt

481 aatcggaatt actgggcgta aagcgtacgt aggcggtgtg ttaagcaaga tgtgaaagcc

541 ccgggctcaa cctgggaatt gcattttgaa ctggcacgct agagtcctga agagggtggt

601 agaatttcca gtgtagcggt gaaatgcgta gatattggaa

//

LOCUS MK617752 640 bp DNA linear BCT 30-MAY-2019

DEFINITION Vibrio sp. strain yL6D 16S ribosomal RNA gene, partial sequence.

ACCESSION MK617752

VERSION MK617752

KEYWORDS .

SOURCE Vibrio sp.

ORGANISM Vibrio sp.

Bacteria; Proteobacteria; Gammaproteobacteria; Vibrionales;

Vibrionaceae; Vibrio.

REFERENCE 1 (bases 1 to 640)

AUTHORS Su,H., Huang,Q. and Yu,K.

TITLE Diversity of Cultivable Protease-Producing Bacteria

JOURNAL Unpublished

REFERENCE 2 (bases 1 to 640)

AUTHORS Su,H., Huang,Q. and Yu,K.

TITLE Direct Submission

JOURNAL Submitted (11-MAR-2019) Coral Reef Research Center of China, School

of Marine Sciences, No. 100 Daxue Road, Xixiangtang District,

Nanning, Guangxi Zhuang Autonomous Region 530004, China

COMMENT ##Assembly-Data-START##

Sequencing Technology :: Sanger dideoxy sequencing

##Assembly-Data-END##

FEATURES Location/Qualifiers

source 1..640

/organism="Vibrio sp."

/mol_type="genomic DNA"

/strain="yL6D"

/isolation_source="coral"

/db_xref="taxon:678"

rRNA <1..>640

/product="16S ribosomal RNA"

ORIGIN

1 gaacgagtta tctgaacctt cgggggacga taacggcgtc gagcggcgga cgggtgagta

61 atgcctagga aattgccctg atgtggggga taaccattgg aaacgatggc taataccgca

121 taatgcctac gggccaaaga gggggacctt cgggcctctc gcgtcaggat atgcctaggt

181 gggattagct agttggtgag gtaatggctc accaaggcga cgatccctag ctggtctgag

241 aggatgatca gccacactgg aactgagaca cggtccagac tcctacggga ggcagcagtg

301 gggaatattg cacaatgggc gcaagcctga tgcagccatg ccgcgtgtgt gaagaaggcc

361 ttcgggttgt aaagcacttt cagtcgtgag gaaggtagtg tagttaatag ctgcattatt

421 tgacgttagc gacagaagaa gcaccggcta actccgtgcc agcagccgcg gtaatacgga

481 gggtgcgagc gttaatcgga attactgggc gtaaagcgca tgcaggtggt ttgttaagtc

541 agatgtgaaa gcccggggct caacctcgga attgcatttg aaactggcag actagagtac

601 tgtagagggg ggtagaattt caggtgtagc ggtgaaatgc

//

LOCUS MK617753 640 bp DNA linear BCT 30-MAY-2019

DEFINITION Vibrio sp. strain yL19a 16S ribosomal RNA gene, partial sequence.

ACCESSION MK617753

VERSION MK617753

KEYWORDS .

SOURCE Vibrio sp.

ORGANISM Vibrio sp.

Bacteria; Proteobacteria; Gammaproteobacteria; Vibrionales;

Vibrionaceae; Vibrio.

REFERENCE 1 (bases 1 to 640)

AUTHORS Su,H., Huang,Q. and Yu,K.

TITLE Diversity of Cultivable Protease-Producing Bacteria

JOURNAL Unpublished

REFERENCE 2 (bases 1 to 640)

AUTHORS Su,H., Huang,Q. and Yu,K.

TITLE Direct Submission

JOURNAL Submitted (11-MAR-2019) Coral Reef Research Center of China, School

of Marine Sciences, No. 100 Daxue Road, Xixiangtang District,

Nanning, Guangxi Zhuang Autonomous Region 530004, China

COMMENT ##Assembly-Data-START##

Sequencing Technology :: Sanger dideoxy sequencing

##Assembly-Data-END##

FEATURES Location/Qualifiers

source 1..640

/organism="Vibrio sp."

/mol_type="genomic DNA"

/strain="yL19a"

/isolation_source="coral"

/db_xref="taxon:678"

rRNA <1..>640

/product="16S ribosomal RNA"

ORIGIN

1 acgagttatc tgaaccttcg gggaacgata acggcgtcga gcggcggacg ggtgagtaat

61 gcctaggaaa ttgccctgat gtgggggata accattggaa acgatggcta ataccgcata

121 atgcctacgg gccaaagagg gggaccttcg ggcctctcgc gtcaggatat gcctaggtgg

181 gattagctag ttggtgaggt aatggctcac caaggcgacg atccctagct ggtctgagag

241 gatgatcagc cacactggaa ctgagacacg gtccagactc ctacgggagg cagcagtggg

301 gaatattgca caatgggcgc aagcctgatg cagccatgcc gcgtgtgtga agaaggcctt

361 cgggttgtaa agcactttca gtcgtgagga aggtagtgta gttaatagct gcattatttg

421 acgttagcga cagaagaagc accggctaac tccgtgccag cagccgcggt aatacggagg

481 gtgcgagcgt taatcggaat tactgggcgt aaagcgcatg caggtggttt gttaagtcag

541 atgtgaaagc ccggggctca acctcggaat tgcatttgaa actggcagac tagagtactg

601 tagagggggg tagaatttca ggtgtagcgg tgaaatgcgt

//

LOCUS MK617754 640 bp DNA linear BCT 30-MAY-2019

DEFINITION Vibrio sp. strain yL8b 16S ribosomal RNA gene, partial sequence.

ACCESSION MK617754

VERSION MK617754

KEYWORDS .

SOURCE Vibrio sp.

ORGANISM Vibrio sp.

Bacteria; Proteobacteria; Gammaproteobacteria; Vibrionales;

Vibrionaceae; Vibrio.

REFERENCE 1 (bases 1 to 640)

AUTHORS Su,H., Huang,Q. and Yu,K.

TITLE Diversity of Cultivable Protease-Producing Bacteria

JOURNAL Unpublished

REFERENCE 2 (bases 1 to 640)

AUTHORS Su,H., Huang,Q. and Yu,K.

TITLE Direct Submission

JOURNAL Submitted (11-MAR-2019) Coral Reef Research Center of China, School

of Marine Sciences, No. 100 Daxue Road, Xixiangtang District,

Nanning, Guangxi Zhuang Autonomous Region 530004, China

COMMENT ##Assembly-Data-START##

Sequencing Technology :: Sanger dideoxy sequencing

##Assembly-Data-END##

FEATURES Location/Qualifiers

source 1..640

/organism="Vibrio sp."

/mol_type="genomic DNA"

/strain="yL8b"

/isolation_source="coral"

/db_xref="taxon:678"

rRNA <1..>640

/product="16S ribosomal RNA"

ORIGIN

1 acgagttatc tgaaccttcg gggaacgata acggcgtcga gcggcggacg ggtgagtaat

61 gcctaggaaa ttgccctgat gtgggggata accattggaa acgatggcta ataccgcata

121 atgcctacgg gccaaagagg gggaccttcg ggcctctcgc gtcaggatat gcctaggtgg

181 gattagctag ttggtgaggt aatggctcac caaggcgacg atccctagct ggtctgagag

241 gatgatcagc cacactggaa ctgagacacg gtccagactc ctacgggagg cagcagtggg

301 gaatattgca caatgggcgc aagcctgatg cagccatgcc gcgtgtgtga agaaggcctt

361 cgggttgtaa agcactttca gtcgtgagga aggtagtgta gttaatagct gcattatttg

421 acgttagcga cagaagaagc accggctaac tccgtgccag cagccgcggt aatacggagg

481 gtgcgagcgt taatcggaat tactgggcgt aaagcgcatg caggtggttt gttaagtcag

541 atgtgaaagc ccggggctca acctcggaat tgcatttgaa actggcagac tagagtactg

601 tagagggggg tagaatttca ggtgtagcgg tgaaatgcgt

//

LOCUS MK617755 640 bp DNA linear BCT 30-MAY-2019

DEFINITION Bacillus sp. (in: Bacteria) strain yL12 16S ribosomal RNA gene,

partial sequence.

ACCESSION MK617755

VERSION MK617755

KEYWORDS .

SOURCE Bacillus sp. (in: Bacteria)

ORGANISM Bacillus sp. (in: Bacteria)

Bacteria; Firmicutes; Bacilli; Bacillales; Bacillaceae; Bacillus.

REFERENCE 1 (bases 1 to 640)

AUTHORS Su,H., Huang,Q. and Yu,K.

TITLE Diversity of Cultivable Protease-Producing Bacteria

JOURNAL Unpublished

REFERENCE 2 (bases 1 to 640)

AUTHORS Su,H., Huang,Q. and Yu,K.

TITLE Direct Submission

JOURNAL Submitted (11-MAR-2019) Coral Reef Research Center of China, School

of Marine Sciences, No. 100 Daxue Road, Xixiangtang District,

Nanning, Guangxi Zhuang Autonomous Region 530004, China

COMMENT ##Assembly-Data-START##

Sequencing Technology :: Sanger dideoxy sequencing

##Assembly-Data-END##

FEATURES Location/Qualifiers

source 1..640

/organism="Bacillus sp. (in: Bacteria)"

/mol_type="genomic DNA"

/strain="yL12"

/isolation_source="coral"

/db_xref="taxon:1409"

rRNA <1..>640

/product="16S ribosomal RNA"

ORIGIN

1 aatcgatggg agcttgctcc ctgagattag cggcggacgg gtgagtaaca cgtgggcaac

61 ctgcctataa gactgggata acttcgggaa accggagcta ataccggata cgttcttttc

121 tcgcatgaga gaagatggaa agacggttta cgctgtcact tatagatggg cccgcggcgc

181 attagctagt tggtgaggta atggctcacc aaggcgacga tgcgtagccg acctgagagg

241 gtgatcggcc acactgggac tgagacacgg cccagactcc tacgggaggc agcagtaggg

301 aatcttccgc aatggacgaa agtctgacgg agcaacgccg cgtgaacgaa gaaggccttc

361 gggtcgtaaa gttctgttgt tagggaagaa caagtaccag agtaactgct ggtaccttga

421 cggtacctaa ccagaaagcc acggctaact acgtgccagc agccgcggta atacgtaggt

481 ggcaagcgtt gtccggaatt attgggcgta aagcgcgcgc aggtggttcc ttaagtctga

541 tgtgaaagcc cacggctcaa ccgtggaggg tcattggaaa ctggggaact tgagtgcaga

601 agaggaaagt ggaattccaa gtgtagcggt gaaatgcgta

//

LOCUS MK617756 640 bp DNA linear BCT 30-MAY-2019

DEFINITION Bacillus sp. (in: Bacteria) strain yL8 16S ribosomal RNA gene,

partial sequence.

ACCESSION MK617756

VERSION MK617756

KEYWORDS .

SOURCE Bacillus sp. (in: Bacteria)

ORGANISM Bacillus sp. (in: Bacteria)

Bacteria; Firmicutes; Bacilli; Bacillales; Bacillaceae; Bacillus.

REFERENCE 1 (bases 1 to 640)

AUTHORS Su,H., Huang,Q. and Yu,K.

TITLE Diversity of Cultivable Protease-Producing Bacteria

JOURNAL Unpublished

REFERENCE 2 (bases 1 to 640)

AUTHORS Su,H., Huang,Q. and Yu,K.

TITLE Direct Submission

JOURNAL Submitted (11-MAR-2019) Coral Reef Research Center of China, School

of Marine Sciences, No. 100 Daxue Road, Xixiangtang District,

Nanning, Guangxi Zhuang Autonomous Region 530004, China

COMMENT ##Assembly-Data-START##

Sequencing Technology :: Sanger dideoxy sequencing

##Assembly-Data-END##

FEATURES Location/Qualifiers

source 1..640

/organism="Bacillus sp. (in: Bacteria)"

/mol_type="genomic DNA"

/strain="yL8"

/isolation_source="coral"

/db_xref="taxon:1409"

rRNA <1..>640

/product="16S ribosomal RNA"

ORIGIN

1 cgtttttgaa gcttgcttca aaaacgttag cggcggacgg gtgagtaaca cgtgggcaac

61 ctaccttatc gactgggata actccgggaa accggggcta ataccggata acatctagca

121 cctcctggtg ccggattaaa agagggcttc ttgctctcac gatgagatgg gcccgcggcg

181 cattagctag ttggagaggt aacggctccc caaggcgacg atgcgtagcc gacctgagag

241 ggtgatcggc cacactggga ctgagacacg gcccagactc ctacgggagg cagcagtagg

301 gaatcttccg caatggacga aagtctgacg gagcaacgcc gcgtgagtga tgaagggttt

361 cggctcgtaa agctctgtta tgagggaaga acacgtaccg ttcgaatagg gcggtacctt

421 gacggtacct catcagaaag ccacggctaa ctacgtgcca gcagccgcgg taatacgtag

481 gtggcaagcg ttgtccggaa ttattgggcg taaagcgcgc gcaggcggcc ttttaagtct

541 gatgtgaaat cttgcggctc aaccgcaagc ggccattgga aactgggagg cttgagtaca

601 gaagaggaga gtggaattcc acgtgtagcg gtgaaatgcg

//

LOCUS MK617757 640 bp DNA linear BCT 30-MAY-2019

DEFINITION Bacillus sp. (in: Bacteria) strain yQ5 16S ribosomal RNA gene,

partial sequence.

ACCESSION MK617757

VERSION MK617757

KEYWORDS .

SOURCE Bacillus sp. (in: Bacteria)

ORGANISM Bacillus sp. (in: Bacteria)

Bacteria; Firmicutes; Bacilli; Bacillales; Bacillaceae; Bacillus.

REFERENCE 1 (bases 1 to 640)

AUTHORS Su,H., Huang,Q. and Yu,K.

TITLE Diversity of Cultivable Protease-Producing Bacteria

JOURNAL Unpublished

REFERENCE 2 (bases 1 to 640)

AUTHORS Su,H., Huang,Q. and Yu,K.

TITLE Direct Submission

JOURNAL Submitted (11-MAR-2019) Coral Reef Research Center of China, School

of Marine Sciences, No. 100 Daxue Road, Xixiangtang District,

Nanning, Guangxi Zhuang Autonomous Region 530004, China

COMMENT ##Assembly-Data-START##

Sequencing Technology :: Sanger dideoxy sequencing

##Assembly-Data-END##

FEATURES Location/Qualifiers

source 1..640

/organism="Bacillus sp. (in: Bacteria)"

/mol_type="genomic DNA"

/strain="yQ5"

/isolation_source="coral"

/db_xref="taxon:1409"

rRNA <1..>640

/product="16S ribosomal RNA"

ORIGIN

1 acagatggga gcttgctccc tgaagtcagc ggcggacggg tgagtaacac gtgggcaacc

61 tgcctgtaag actgggataa ctccgggaaa ccggggctaa taccggataa ttctttccct

121 cacatgaggg aaagctgaaa gatggtttcg gctatcactt acagatgggc ccgcggcgca

181 ttagctagtt ggtgaggtaa cggctcacca aggcaacgat gcgtagccga cctgagaggg

241 tgatcggcca cactgggact gagacacggc ccagactcct acgggaggca gcagtaggga

301 atcttccgca atggacgaaa gtctgacgga gcaacgccgc gtgagtgatg aaggttttcg

361 gatcgtaaaa ctctgttgtt agggaagaac aagtaccgga gtaactgccg gtaccttgac

421 ggtacctaac cagaaagcca cggctaacta cgtgccagca gccgcggtaa tacgtaggtg

481 gcaagcgttg tccggaatta ttgggcgtaa agcgcgcgca ggcggttcct taagtctgat

541 gtgaaagccc ccggctcaac cggggagggt cattggaaac tggggaactt gagtgcagaa

601 gagaagagtg gaattccacg tgtagcggtg aaatgcgtag

//

LOCUS MK617758 640 bp DNA linear BCT 30-MAY-2019

DEFINITION Fictibacillus sp. strain yQ11 16S ribosomal RNA gene, partial

sequence.

ACCESSION MK617758

VERSION MK617758

KEYWORDS .

SOURCE Fictibacillus sp.

ORGANISM Fictibacillus sp.

Bacteria; Firmicutes; Bacilli; Bacillales; Bacillaceae;

Fictibacillus.

REFERENCE 1 (bases 1 to 640)

AUTHORS Su,H., Huang,Q. and Yu,K.

TITLE Diversity of Cultivable Protease-Producing Bacteria

JOURNAL Unpublished

REFERENCE 2 (bases 1 to 640)

AUTHORS Su,H., Huang,Q. and Yu,K.

TITLE Direct Submission

JOURNAL Submitted (11-MAR-2019) Coral Reef Research Center of China, School

of Marine Sciences, No. 100 Daxue Road, Xixiangtang District,

Nanning, Guangxi Zhuang Autonomous Region 530004, China

COMMENT ##Assembly-Data-START##

Sequencing Technology :: Sanger dideoxy sequencing

##Assembly-Data-END##

FEATURES Location/Qualifiers

source 1..640

/organism="Fictibacillus sp."

/mol_type="genomic DNA"

/strain="yQ11"

/isolation_source="coral"

/db_xref="taxon:1871617"

rRNA <1..>640

/product="16S ribosomal RNA"

ORIGIN

1 aatgatgagg agcttgctcc tctgatttag cggcggacgg gtgagtaaca cgtgggtaat

61 ctgcctgtaa gacggggata actccgggaa accggggcta ataccggata ataagagaag

121 aagcatttct tctttttgaa agtcggtttc ggctgacact tacagatgag cccgcggcgc

181 attagctagt tggtgaggta acggctcacc aaggcgacga tgcgtagccg acctgagagg

241 gtgatcggcc acactgggac tgagacacgg cccagactcc tacgggaggc agcagtaggg

301 aatcttcggc aatgggcgaa agcctgaccg agcaacgccg cgtgagcgat gaaggccttc

361 gggtcgtaaa gctctgttgt tagagaagaa caagtacgag agtaactgct cgtaccttga

421 cggtacctaa ccagaaagcc acggctaact acgtgccagc agccgcggta atacgtaggt

481 ggcaagcgtt atccggaatt attgggcgta aagcgcgcgc aggcggtctc ttaagtctga

541 tgtgaaagcc cacggctcaa ccgtggaggg tcattggaaa ctgggagact tgagtgcagg

601 agagaaaagt ggaattccac gtgtagcggt gaaatgcgta

//

LOCUS MK617759 640 bp DNA linear BCT 30-MAY-2019

DEFINITION Pseudomonas sp. strain yQ15 16S ribosomal RNA gene, partial

sequence.

ACCESSION MK617759

VERSION MK617759

KEYWORDS .

SOURCE Pseudomonas sp.

ORGANISM Pseudomonas sp.

Bacteria; Proteobacteria; Gammaproteobacteria; Pseudomonadales;

Pseudomonadaceae; Pseudomonas.

REFERENCE 1 (bases 1 to 640)

AUTHORS Su,H., Huang,Q. and Yu,K.

TITLE Diversity of Cultivable Protease-Producing Bacteria

JOURNAL Unpublished

REFERENCE 2 (bases 1 to 640)

AUTHORS Su,H., Huang,Q. and Yu,K.

TITLE Direct Submission

JOURNAL Submitted (11-MAR-2019) Coral Reef Research Center of China, School

of Marine Sciences, No. 100 Daxue Road, Xixiangtang District,

Nanning, Guangxi Zhuang Autonomous Region 530004, China

COMMENT ##Assembly-Data-START##

Sequencing Technology :: Sanger dideoxy sequencing

##Assembly-Data-END##

FEATURES Location/Qualifiers

source 1..640

/organism="Pseudomonas sp."

/mol_type="genomic DNA"

/strain="yQ15"

/isolation_source="coral"

/db_xref="taxon:306"

rRNA <1..>640

/product="16S ribosomal RNA"

ORIGIN

1 tgagtggagc ttgctccatg attcagcggc ggacgggtga gtaatgccta ggaatctgcc

61 tggtagtggg ggacaacgtt tcgaaaggaa cgctaatacc gcatacgtcc tacgggagaa

121 agtgggggat cttcggacct cacgctatca gatgagccta ggtcggatta gctagttggt

181 ggggtaaagg ctcaccaagg cgacgatccg taactggtct gagaggatga tcagtcacac

241 tggaactgag acacggtcca gactcctacg ggaggcagca gtggggaata ttggacaatg

301 ggcgaaagcc tgatccagcc atgccgcgtg tgtgaagaag gtcttcggat tgtaaagcac

361 tttaagttgg gaggaagggc agtaagttaa taccttgctg ttttgacgtt accgacagaa

421 taagcaccgg ctaacttcgt gccagcagcc gcggtaatac gaagggtgca agcgttaatc

481 ggaattactg ggcgtaaagc gcgcgtaggt ggttcgttaa gttggatgtg aaagccccgg

541 gctcaacctg ggaactgcat ccaaaactgg cgagctagag tatggcagag ggtggtggaa

601 tttcctgtgt agcggtgaaa tgcgtagata taggaaggaa

//

LOCUS MK617760 640 bp DNA linear BCT 30-MAY-2019

DEFINITION Bacillus sp. (in: Bacteria) strain yQ16 16S ribosomal RNA gene,

partial sequence.

ACCESSION MK617760

VERSION MK617760

KEYWORDS .

SOURCE Bacillus sp. (in: Bacteria)

ORGANISM Bacillus sp. (in: Bacteria)

Bacteria; Firmicutes; Bacilli; Bacillales; Bacillaceae; Bacillus.

REFERENCE 1 (bases 1 to 640)

AUTHORS Su,H., Huang,Q. and Yu,K.

TITLE Diversity of Cultivable Protease-Producing Bacteria

JOURNAL Unpublished

REFERENCE 2 (bases 1 to 640)

AUTHORS Su,H., Huang,Q. and Yu,K.

TITLE Direct Submission

JOURNAL Submitted (11-MAR-2019) Coral Reef Research Center of China, School

of Marine Sciences, No. 100 Daxue Road, Xixiangtang District,

Nanning, Guangxi Zhuang Autonomous Region 530004, China

COMMENT ##Assembly-Data-START##

Sequencing Technology :: Sanger dideoxy sequencing

##Assembly-Data-END##

FEATURES Location/Qualifiers

source 1..640

/organism="Bacillus sp. (in: Bacteria)"

/mol_type="genomic DNA"

/strain="yQ16"

/isolation_source="coral"

/db_xref="taxon:1409"

rRNA <1..>640

/product="16S ribosomal RNA"

ORIGIN

1 tttactgaga gtttgatcct ggctcaggac gaacgctggc ggcgtgccta atacatgcaa

61 gtcgtgcgga ccttataaat gcttgctttt aaaaggttag cggcggacgg gtgagtaaca

121 cgtgggcaac ctgcctgtaa gaccgggata acgccgggaa accggggcta ataccggata

181 gttatttcct ccgcatggaa gaaaaaggaa aggcggcttc ggctgccact tacagatggg

241 cccgctgcgc attagctagt tggcggggta acggcccacc aaggcaacga tgcgtagccg

301 acctgagagg gtgatcggcc acattgggac tgagacacgg cccaaactcc tacgggaggc

361 agcagtaggg aatcttccgc aatggacgaa agtctgacgg agcaacgccg cgtgagtgaa

421 gaaggccttc gggtcgtaaa actctgttgc cggggaagaa caagtgccgt tcgaacaggg

481 cggcaccttg acggtacgcg gccagaaagc cacggctaac tacgtgccag cagccgcggt

541 aatacgtagg tggcaagcgt tgtccggaat tattgggcgt aaagcgcgcg caggcggctt

601 cttaagtctg atgtgaaatc ttgcggctca accgcaagcg

//

LOCUS MK617761 640 bp DNA linear BCT 30-MAY-2019

DEFINITION Fictibacillus sp. strain yQ18 16S ribosomal RNA gene, partial

sequence.

ACCESSION MK617761

VERSION MK617761

KEYWORDS .

SOURCE Fictibacillus sp.

ORGANISM Fictibacillus sp.

Bacteria; Firmicutes; Bacilli; Bacillales; Bacillaceae;

Fictibacillus.

REFERENCE 1 (bases 1 to 640)

AUTHORS Su,H., Huang,Q. and Yu,K.

TITLE Diversity of Cultivable Protease-Producing Bacteria

JOURNAL Unpublished

REFERENCE 2 (bases 1 to 640)

AUTHORS Su,H., Huang,Q. and Yu,K.

TITLE Direct Submission

JOURNAL Submitted (11-MAR-2019) Coral Reef Research Center of China, School

of Marine Sciences, No. 100 Daxue Road, Xixiangtang District,

Nanning, Guangxi Zhuang Autonomous Region 530004, China

COMMENT ##Assembly-Data-START##

Sequencing Technology :: Sanger dideoxy sequencing

##Assembly-Data-END##

FEATURES Location/Qualifiers

source 1..640

/organism="Fictibacillus sp."

/mol_type="genomic DNA"

/strain="yQ18"

/isolation_source="coral"

/db_xref="taxon:1871617"

rRNA <1..>640

/product="16S ribosomal RNA"

ORIGIN

1 gaatgatgag gagcttgctc ctctgattta gcggcggacg ggtgagtaac acgtgggtaa

61 tctgcctgta agacggggat aactccggga aaccggggct aataccggat aataagagaa

121 gaagcatttc ttctttttga aagtcggttt cggctgacac ttacagatga gcccgcggcg

181 cattagctag ttggtgaggt aacggctcac caaggcgacg atgcgtagcc gacctgagag

241 ggtgatcggc cacactggga ctgagacacg gcccagactc ctacgggagg cagcagtagg

301 gaatcttcgg caatgggcga aagcctgacc gagcaacgcc gcgtgagcga tgaaggcctt

361 cgggtcgtaa agctctgttg ttagagaaga acaagtacga gagtaactgc tcgtaccttg

421 acggtaccta accagaaagc cacggctaac tacgtgccag cagccgcggt aatacgtagg

481 tggcaagcgt tatccggaat tattgggcgt aaagcgcgcg caggcggtct cttaagtctg

541 atgtgaaagc ccacggctca accgtggagg gtcattggaa actgggagac ttgagtgcag

601 gagagaaaag tggaattcca cgtgtagcgg tgaaatgcgt

//

LOCUS MK617762 645 bp DNA linear BCT 30-MAY-2019

DEFINITION Lysinibacillus sp. strain B21 16S ribosomal RNA gene, partial

sequence.

ACCESSION MK617762

VERSION MK617762

KEYWORDS .

SOURCE Lysinibacillus sp.

ORGANISM Lysinibacillus sp.

Bacteria; Firmicutes; Bacilli; Bacillales; Bacillaceae;

Lysinibacillus.

REFERENCE 1 (bases 1 to 645)

AUTHORS Su,H., Huang,Q. and Yu,K.

TITLE Diversity of Cultivable Protease-Producing Bacteria

JOURNAL Unpublished

REFERENCE 2 (bases 1 to 645)

AUTHORS Su,H., Huang,Q. and Yu,K.

TITLE Direct Submission

JOURNAL Submitted (11-MAR-2019) Coral Reef Research Center of China, School

of Marine Sciences, No. 100 Daxue Road, Xixiangtang District,

Nanning, Guangxi Zhuang Autonomous Region 530004, China

COMMENT ##Assembly-Data-START##

Sequencing Technology :: Sanger dideoxy sequencing

##Assembly-Data-END##

FEATURES Location/Qualifiers

source 1..645

/organism="Lysinibacillus sp."

/mol_type="genomic DNA"

/strain="B21"

/isolation_source="coral"

/db_xref="taxon:1869345"

rRNA <1..>645

/product="16S ribosomal RNA"

ORIGIN

1 tgtatctgca gtctagcgaa cagaaaagga gcttgctcct ttgacgttag cggcggacgg

61 gtgagtaaca cgtgggcaac ctaccctata gtttgggata actccgggaa accggggcta

121 ataccgaata atctctttta cttcatggtg aaagactgaa agacggtttc ggctgtcgct

181 ataggatggg cccgcggcgc attagctagt tggtgaggta acggctcacc aaggccacca

241 tgcgtagccg acctgagagg gtgatcggcc accctgggac tgagacacgg cccaaactcc

301 tacgggaggc agcagtaggg aatcttccac aatgggcgaa agcctgatgg agcaacgccg

361 cgtgagtgaa gaaggttttc ggatcgtaaa actctgttgt aagggaagaa caagtacagt

421 agtaactggc tgtaccttga cggtacctta ttagaaagcc acggctaact acgtgccagc

481 agccgcggta atacgtaggt ggcaagcgtt gtccggaatt attgggcgta aagcgcgcgc

541 aggcggtcct ttaagtctga tgtgaaagcc cacggctcaa ccgtggaggg tcattggaaa

601 ctgggggact tgagtgcaga agaggaaagt ggaattccaa gactg

//

LOCUS MK617763 640 bp DNA linear BCT 30-MAY-2019

DEFINITION Lysinibacillus sp. strain B24 16S ribosomal RNA gene, partial

sequence.

ACCESSION MK617763

VERSION MK617763

KEYWORDS .

SOURCE Lysinibacillus sp.

ORGANISM Lysinibacillus sp.

Bacteria; Firmicutes; Bacilli; Bacillales; Bacillaceae;

Lysinibacillus.

REFERENCE 1 (bases 1 to 640)

AUTHORS Su,H., Huang,Q. and Yu,K.

TITLE Diversity of Cultivable Protease-Producing Bacteria

JOURNAL Unpublished

REFERENCE 2 (bases 1 to 640)

AUTHORS Su,H., Huang,Q. and Yu,K.

TITLE Direct Submission

JOURNAL Submitted (11-MAR-2019) Coral Reef Research Center of China, School

of Marine Sciences, No. 100 Daxue Road, Xixiangtang District,

Nanning, Guangxi Zhuang Autonomous Region 530004, China

COMMENT ##Assembly-Data-START##

Sequencing Technology :: Sanger dideoxy sequencing

##Assembly-Data-END##

FEATURES Location/Qualifiers

source 1..640

/organism="Lysinibacillus sp."

/mol_type="genomic DNA"

/strain="B24"

/isolation_source="coral"

/db_xref="taxon:1869345"

rRNA <1..>640

/product="16S ribosomal RNA"

ORIGIN

1 ttttatggag agtttgatcc tggctcagga cgaacgctgg cggcgtgcct aatacatgca

61 agtcgagcga acagagaagg agcttgctcc ttcgacgtta gcggcggacg ggtgagtaac

121 acgtgggcaa cctaccttat agtttgggat aactccggga aaccggggct aataccgaat

181 aatctgtttc acctcatggt gaaacactga aagacggttt cggctgtcgc tataggatgg

241 gcccgcggcg cattagctag ttggtgaggt aacggctcac caaggcgacg atgcgtagcc

301 gacctgagag ggtgatcggc cacactggga ctgagacacg gcccagactc ctacgggagg

361 cagcagtagg gaatcttcca caatgggcga aagcctgatg gagcaacgcc gcgtgagtga

421 agaaggattt cggttcgtaa aactctgttg taagggaaga acaagtacag tagtaactgg

481 ctgtaccttg acggtacctt attagaaagc cacggctaac tacgtgccag cagccgcggt

541 aatacgtagg tggcaagcgt tgtccggaat tattgggcgt aaagcgcgcg caggtggttt

601 cttaagtctg atgtgaaagc ccacggctca accgtggagg

//

LOCUS MK617764 640 bp DNA linear BCT 30-MAY-2019

DEFINITION Vibrio sp. strain D38 16S ribosomal RNA gene, partial sequence.

ACCESSION MK617764

VERSION MK617764

KEYWORDS .

SOURCE Vibrio sp.

ORGANISM Vibrio sp.

Bacteria; Proteobacteria; Gammaproteobacteria; Vibrionales;

Vibrionaceae; Vibrio.

REFERENCE 1 (bases 1 to 640)

AUTHORS Su,H., Huang,Q. and Yu,K.

TITLE Diversity of Cultivable Protease-Producing Bacteria

JOURNAL Unpublished

REFERENCE 2 (bases 1 to 640)

AUTHORS Su,H., Huang,Q. and Yu,K.

TITLE Direct Submission

JOURNAL Submitted (11-MAR-2019) Coral Reef Research Center of China, School

of Marine Sciences, No. 100 Daxue Road, Xixiangtang District,

Nanning, Guangxi Zhuang Autonomous Region 530004, China

COMMENT ##Assembly-Data-START##

Sequencing Technology :: Sanger dideoxy sequencing

##Assembly-Data-END##

FEATURES Location/Qualifiers

source 1..640

/organism="Vibrio sp."

/mol_type="genomic DNA"

/strain="D38"

/isolation_source="coral"

/db_xref="taxon:678"

rRNA <1..>640

/product="16S ribosomal RNA"

ORIGIN

1 cttaactgaa ccttcggggg acgttaaggg cgtcgagcgg cggacgggtg agtaatgcct

61 gggaatatgc cttgatgtgg gggataacca ttggaaacga tggctaatac cgcataatct

121 cttcggagca aagaggggga ccttcgggcc tctcgcgtca agattagccc aggtgggatt

181 agctagttgg tgaggtaatg gctcaccaag gcgacgatcc ctagctggtc tgagaggatg

241 atcagccaca ctggaactga gacacggtcc agactcctac gggaggcagc agtggggaat

301 attgcacaat gggcgcaagc ctgatgcagc catgccgcgt gtgtgaagaa ggccttcggg

361 ttgtaaagca ctttcagcag tgaggaaggt ggtgtcgtta atagcggcat catttgacgt

421 tagctgcaga agaagcaccg gctaactccg tgccagcagc cgcggtaata cggagggtgc

481 gagcgttaat cggaattact gggcgtaaag cgcatgcagg tggtttgtta agtcagatgt

541 gaaagcccgg ggctcaacct cggaaccgca tttgaaactg gcaggctaga gtactgtaga

601 ggggggtaga atttcaggtg tagcggtgaa atgcgtagag

//

LOCUS MK617765 640 bp DNA linear BCT 30-MAY-2019

DEFINITION Pseudoalteromonas sp. strain yL11 16S ribosomal RNA gene, partial

sequence.

ACCESSION MK617765

VERSION MK617765

KEYWORDS .

SOURCE Pseudoalteromonas sp.

ORGANISM Pseudoalteromonas sp.

Bacteria; Proteobacteria; Gammaproteobacteria; Alteromonadales;

Pseudoalteromonadaceae; Pseudoalteromonas.

REFERENCE 1 (bases 1 to 640)

AUTHORS Su,H., Huang,Q. and Yu,K.

TITLE Diversity of Cultivable Protease-Producing Bacteria

JOURNAL Unpublished

REFERENCE 2 (bases 1 to 640)

AUTHORS Su,H., Huang,Q. and Yu,K.

TITLE Direct Submission

JOURNAL Submitted (11-MAR-2019) Coral Reef Research Center of China, School

of Marine Sciences, No. 100 Daxue Road, Xixiangtang District,

Nanning, Guangxi Zhuang Autonomous Region 530004, China

COMMENT ##Assembly-Data-START##

Sequencing Technology :: Sanger dideoxy sequencing

##Assembly-Data-END##

FEATURES Location/Qualifiers

source 1..640

/organism="Pseudoalteromonas sp."

/mol_type="genomic DNA"

/strain="yL11"

/isolation_source="coral"

/db_xref="taxon:53249"

rRNA <1..>640

/product="16S ribosomal RNA"

ORIGIN

1 gaacgaagag gagcttgctc ctttggcgtc gagcggcgga cgggtgagta atgcttggga

61 atgtgcctta tggtggggga caacagttgg aaacgactgc taataccgca taatgtcttc

121 ggaccaaagt gggggacctt cgggcctcac gccataagat cagcccaagt gggattagct

181 agttggtaag gtaatggctt accaaggcga cgatccctag ctggtttgag aggatgatca

241 gccacactgg gactgagaca cggcccagac tcctacggga ggcagcagtg gggaatattg

301 cacaatgggc gcaagcctga tgcagccatg ccgcgtgtgt gaagaaggcc ttcgggttgt

361 aaagcacttt cagtaaggag gaaaggttaa gtgttaatag cacttagctg tgacgttact

421 tacagaagaa gcaccggcta actccgtgcc agcagccgcg gtaatacgga gggtgcgagc

481 gttaatcgga attactgggc gtaaagcgta cgcaggcggt ttgttaagcg agatgtgaaa

541 gccccgggct caacctggga actgcatttc gaactggcaa actagagtgt gatagagggt

601 ggtagaattt caggtgtagc ggtgaaatgc gtagagatct

//

LOCUS MK617766 640 bp DNA linear BCT 30-MAY-2019

DEFINITION Shewanella sp. strain yL13 16S ribosomal RNA gene, partial

sequence.

ACCESSION MK617766

VERSION MK617766

KEYWORDS .

SOURCE Shewanella sp.

ORGANISM Shewanella sp.

Bacteria; Proteobacteria; Gammaproteobacteria; Alteromonadales;

Shewanellaceae; Shewanella.

REFERENCE 1 (bases 1 to 640)

AUTHORS Su,H., Huang,Q. and Yu,K.

TITLE Diversity of Cultivable Protease-Producing Bacteria

JOURNAL Unpublished

REFERENCE 2 (bases 1 to 640)

AUTHORS Su,H., Huang,Q. and Yu,K.

TITLE Direct Submission

JOURNAL Submitted (11-MAR-2019) Coral Reef Research Center of China, School

of Marine Sciences, No. 100 Daxue Road, Xixiangtang District,

Nanning, Guangxi Zhuang Autonomous Region 530004, China

COMMENT ##Assembly-Data-START##

Sequencing Technology :: Sanger dideoxy sequencing

##Assembly-Data-END##

FEATURES Location/Qualifiers

source 1..640

/organism="Shewanella sp."

/mol_type="genomic DNA"

/strain="yL13"

/isolation_source="coral"

/db_xref="taxon:50422"

rRNA <1..>640

/product="16S ribosomal RNA"

ORIGIN

1 aacagaaagt agcttgctac tttgctgacg agcggcggac gggtgagtaa tgcctaggga

61 tctgcccagt cgagggggat aacagttgga aacgactgct aataccgcat acgccctacg

121 ggggaaagga ggggaccttc gggcctttcg cgattggatg aacctaggtg ggattagcta

181 gttggtaagg taatggctta ccaaggcgac gatccctagc tgttctgaga ggatgatcag

241 ccacactggg actgagacac ggcccagact cctacgggag gcagcagtgg ggaatattgc

301 acaatggggg aaaccctgat gcagccatgc cgcgtgtgtg aagaaggcct tcgggttgta

361 aagcactttc agtagggagg aaaggtagca gcttaatacg ttgttgctgt gacgttacct

421 acagaagaag gaccggctaa cttcgtgcca gcagccgcgg taatacgagg ggtccaagcg

481 ttaatcggaa ttactgggcg taaagcgtac gcaggcggtt cattaagcca gatgtgaaat

541 ccccgggctc aacctgggaa ttgcatttgg aactggtgaa ctagagtctt gtagaggggg

601 gtagaatttc aggtgtagcg gtgaaatgcg tagagatctg

//
